# Difluoromethylation of (hetero)aryl chlorides with chlorodifluoromethane catalyzed by nickel

**DOI:** 10.1038/s41467-018-03532-1

**Published:** 2018-03-21

**Authors:** Chang Xu, Wen-Hao Guo, Xu He, Yin-Long Guo, Xue-Ying Zhang, Xingang Zhang

**Affiliations:** Key Laboratory of Organofluorine Chemistry, Center for Excellence in Molecular Synthesis, Shanghai Institute of Organic Chemistry, University of Chinese Academy of Sciences, Chinese Academy of Sciences, 345 Lingling Road, 200032 Shanghai, China

## Abstract

Relatively low reactivity hinders using chlorodifluoromethane (ClCF_2_H) for general difluoromethylation with organic molecules, despite its availability as an inexpensive industrial chemical. To date, transformations of ClCF_2_H are very limited and most of them involve difluorocarbene intermediate. Here, we describe a strategy for difluoromethylation of aromatics through nickel-catalyzed cross-coupling of ClCF_2_H with readily accessible (hetero)aryl chlorides. The reaction proceeds under mild reaction conditions with high efficiency and features synthetic simplicity without preformation of arylmetals and broad substrate scope, including a variety of heteroaromatics and commercially available pharmaceuticals. The reliable practicability and scalability of the current nickel-catalyzed process has also been demonstrated by several 10-g scale reactions without loss of reaction efficiency. Preliminary mechanistic studies reveal that the reaction starts from the oxidative addition of aryl chlorides to Ni(0) and a difluoromethyl radical is involved in the reaction, providing a route for applications of ClCF_2_H in organic synthesis and related chemistry.

## Abstract (web summary)

Transformations with ClCF_2_H are very limited and normally involve a difluorocarbene intermediate. Here, the authors report a nickel-catalyzed difluoromethylation of aryl chlorides with chlorodifluoromethane via a difluoromethyl radical intermediate and apply the method to the synthesis of marketed pharmaceuticals.

## Introduction

**Fig. 1:**
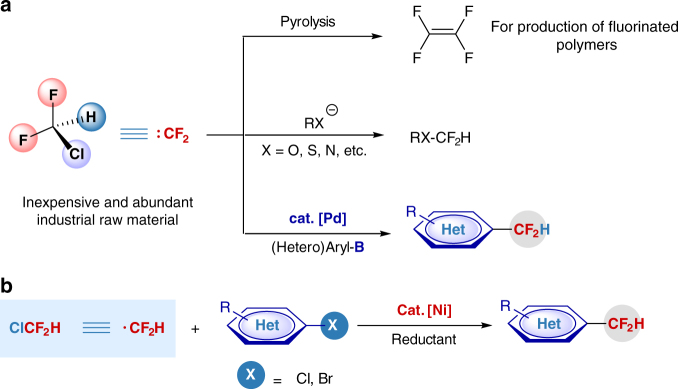
Strategies in activation of ClCF_2_H. **a** Previous work, activation of ClCF_2_H via a difluorocarbene pathway. **b** This work, a new activation of ClCF_2_H through a difluoromethyl radical pathway

Difluoromethylation of organic molecules using chlorodifluoromethane (ClCF_2_H), an inexpensive industrial raw material used for production of fluorinated polymers^1^, represents a cost-efficient and straightforward route to the synthesis of paramount important fluorinated compounds^2–12^. Its activation and transformation, however, is still of great challenge, due to the strong C–Cl bonding in this gaseous compound. Thus far, most transformation paths of ClCF_2_H involve the difluorocarbene intermediate. The difluorocarbene species formed through pyrolysis at high temperature or through dehydrochlorination under strong basic conditions has very limited synthetic applications: the former is only applied to produce tetrafluoroethylene (TFE) and related fluorinated polymers (e.g., Teflon)^1^, and the latter is used to prepare heteroatom-substituted difluoromethylated compounds^13–16^. Very recently, we have developed a palladium-catalyzed difluoromethylation of arylborons with ClCF_2_H, also via a difluorocarbene pathway (Fig. 1a)^17^. This study has also demonstrated that the activation of ClCF_2_H by transition metal can be conducted under mild reaction conditions and thus has wide application potential.

For both the practical application and the fundamental research, replacing the palladium catalyst by a first-row-based transition metal catalyst would pave a new and more cost-efficient way for applications of ClCF_2_H in organic synthesis and medicinal chemistry. Instead of ClCF_2_H activation, only rare examples of nickel catalyzed difluoroalkylation have been reported for the cross-coupling of difluoroalkyl chloride (ClCF_2_CO_2_Et) with nucleophilic arylboronic acids, in which the C–Cl bond is activated by an ester group (CO_2_Et) adjacent to the difluorocarbon^18–20^. As an inert substrate, the direct cleavage of C–Cl bond in ClCF_2_H remains a great challenge. Herein, we report a nickel-catalyzed reductive cross-coupling between ClCF_2_H and aryl chlorides^21–27^, representing an alternative strategy for the fluoroalkylation reactions. The reaction proceeds under mild reaction conditions with high efficiency and enables difluoromethylation of a variety of inexpensive and readily accessible aryl chlorides, including heteroaromatics and commercially available pharmaceuticals. The reaction can also extend to aryl bromides. In contrast to the difluorocarbene intermediate involved in the previous palladium-catalyzed process, the current Ni-catalyzed difluoromethylations undergoes a difluoromethyl radical pathway through the direct cleavage of C–Cl bond in ClCF_2_H.

## Results

### Optimization of the Ni-catalyzed cross-coupling

We began our studies on nickel-catalyzed reductive cross-coupling between ClCF_2_H and aryl chlorides (Fig. 1b). The use of aryl chlorides is because of their cheapness, ready availability, and more importantly, we proposed a direct transformation that can avoid the need for preformed arylmetals, such as arylborons and arylzincs. A suitable nickel catalytic system is the key to realize this synthesis route. To date, however, such a nickel catalyzed reductive cross-coupling between organohalides and fluoroalkylated electrophiles has not been reported and remains a challenge, because of the difficulties in selectively controlling the catalytic cycle to suppress the side reactions, such as the formation of hydrodehalogenated and dimerized fluorinated by-products. Although important progresses have been achieved in nickel-catalyzed reductive cross-coupling between aryl halides and unactivated alkyl halides^28,29^, specific challenges still exist in similar reactions of inert aryl chlorides without substitution by electron-withdrawing groups^30^, which is subject to the coupling reaction in the current study.

Accordingly, 1-(*tert*-butyl)-4-chlorobenzene **2a** was chosen as the model substrate for this nickel-catalyzed difluoromethylation reaction (Table 1). Without additives, either no reaction or low yields of difluoromethylated arene **3a** were observed in most of the experiments. The addition of MgCl_2_^31^ (1.5 equiv.) benefited the reaction with 16% yield of **3a** obtained when the reaction was carried out with NiCl_2_·DME (10 mol%, DME, dimethoxyethane), bpy **L1** (10 mol%, bpy, 2,2′-bipyridine) and Zn (2.0 equiv.) in DMA (dimethylacetamide) at 80 °C (entry 1). But other additives, such as HCl, TMSCl (trimethyl chlorosilane), or DIBAL-H (diisobutyl aluminum hydride) showed no reactivities (Supplementary Table 13). Encouraged by this result, a survey of the reaction parameters, such as ligands, nickel sources, and solvents, was conducted. Unfortunately, no significant improvement of the reaction efficiency was observed (entries 2–4 and Supplementary Tables 1–2). Since a more electron-rich nickel center can benefit the oxidative addition to C–Cl bond, the combination of two electron rich ligands **L4** and DMAP (4-dimethylaminopyridine)^32,33^ provided **3a** in 46% yield (entry 5). Other pyridine-based ligands were also examined, but were inferior to DMAP (entries 6–8). Decreasing the reaction temperature to 60 °C with 10 mol% of NiCl_2_ could improve the yield of **3a** to 59% (entry 9). Further optimization of the reaction conditions (Supplementary Tables 4–12) revealed that the addition of 3 Å molecular sieves (MS) in conjunction with NiCl_2_ (15 mol%) and **L4** (10 mol%) could afford **3a** in 79% yield (entry 10). In parallel, NiBr_2_ as an alternative catalyst under the same reaction conditions could also lead to **3a** in a comparable yield (entry 11). It should be mentioned that the use of 10 mol% of NiCl_2_ and 10 mol% of **L4** with 3 Å MS could also lead to **3a** in a comparable yield (76%) sometimes. But in most of the cases, we obtained the yields of **3a** in a range of 46% to 76%. We supposed that the use of excessive NiCl_2_ vs **L4** was probably because a comproportionation occurred between [Ni^II^] and in situ generated [Ni^0^]. Switching Zn with organic reductant tetrakis(dimethylamino)ethylene (TDAE) led to no **3a** (Supplementary Table 14). The absence of nickel or **L4** failed to provide **3a** either (entries 12 and 13). Thus, these findings demonstrate that Ni/**L4** and Zn play an essential role in promotion of the reaction.

**Table 1:** Representative results for the optimization of Ni-catalyzed difluoromethylation of **2a** with C1CF_2_H^a^


Entry	**L** (mol%)	Additive (x)	yield^b^ (%), **3a**
1	**L1**	MgCl_2_ (1.5)	16
2	**L2**	MgCl_2_ (1.5)	21
3	**L3**	MgCl_2_ (1.5)	10
4	**L4**	MgCl_2_ (1.5)	33
5	**L4** + DMAP (20)	MgCl_2_ (1.5)	46
6	**L4** + Py (20)	MgCl_2_ (1.5)	35
7	**L4** + 4-MeOPy (20)	MgCl_2_ (1.5)	38
8	**L4** + 4-CF_3_Py (20)	MgCl_2_ (1.5)	31
9^c^	**L4** + DMAP (20)	MgCl_2_ (4.0)	59
10^d^	**L4** + DMAP (20)	MgCl_2_ (4.0)	79
11^e^	**L4** + DMAP (20)	MgCl_2_ (4.0)	77
12^f^	**L4** + DMAP (20)	MgCl_2_ (4.0)	NR
13^g^	DMAP (20)	MgCl_2_ (4.0)	NR

*NR* no reaction

^a^Reaction conditions (unless otherwise specified): **1** (2.6  M in DMA, 6.5 equiv.), **2a** (0.2 mmol, 1.0 equiv.), DMA (2  mL)

^b^Determined by ^19^F NMR using fluorobenzene as an internal standard

^c^NiCl_2_ (10 mol%) and Zn (3.0 equiv.) were used and reaction run at 60 °C

^d^NiCl_2_ (15 mol %), **L4** (10 mol%), Zn (3.0 equiv.), and 3 Å MS were used and reaction run at 60 °C

^e^NiBr_2_ (15 mol%), **L4** (10 mol%), Zn (3.0 equiv.), and 3 Å MS were used and reaction run at 60 °C

^f^Reaction run in the absence of nickel catalyst

^g^Reaction run in the absence of **L4**

### Scope of the Ni-catalyzed cross-coupling

With the viable reaction conditions in hand, a variety of aryl chlorides were examined (Table 2). For the electron-neutral aryl chlorides, the use of 10 mol% of NiCl_2_ and **L4** in a low amount as 5 mol% still provided corresponding difluoromethylated arenes in high yields (**2b**–**2d**, **2x**, and **2y**). Although the *ortho* substituted substrates, such as *ortho* methyl, fluoride, vinyl and ester substituted phenyl chlorides furnished the difluoromethylated products in lower yields (**2e**–**2g** and **2n**), they are still synthetically useful for medicinal chemistry to access otherwise unavailable compounds. Aryl chlorides bearing electron-donating substituents were also amenable to the reaction, leading to **3h–3k** in moderate to good yields (**2h**–**2k**), in which methoxyl group at *meta* position provided higher yield than that at *para* position (**2i** and **2h**). Thus, the current nickel-catalyzed process shows the much larger substrate scope than the previous nickel-catalyzed reductive cross-coupling, where the electron-rich and -neutral aryl chlorides have no reaction or only lead to poor yields^30^. Electron-deficient aryl chlorides were also competent coupling partners, highlighting the generality of this approach (**2l**–**2r**). The reaction exhibited good tolerance to functional groups including base or nucleophile-sensitive moieties, such as alkoxycarbonyl and enolizable ketone, and other groups such as vinyl, methylsulfonyl, nitrile, and substituted piperazine (**2g**, **2l–2t**). Remarkably, alcohol and arylboronate did not interfere with the reaction efficiency, and led to compounds **2u–2w** in good yields, featuring the advantages of this approach. Furthermore, the reaction can also extend to aryl bromides, both electron-rich and electron-deficient aryl bromides were suitable substrates (**2c′**, **2i′**, and **2l′**).

**Table 2:** Scope of the nickel-catalyzed reductive cross-coupling of ClCF_2_H with aryl chlorides^a^

^a^Reaction conditions (unless otherwise specified): (hetero)aryl chloride (0.2 mmol, 1.0 equiv.), **1** (2.6 M in DMA, 6.5 equiv.), DMA (2 mL), 60 °C, 20 h. Average isolated yields from two runs

^b^Yield determined by ^19^F NMR using fluorobenzene or trifluorotoluene as an internal standard

^c^15 mol% of NiCl_2_ and 10 mol% of **L4** were used

^d^2.0 equiv. of ClCF_2_H was used and the reaction was conducted on 3 mmol scale

^e^20 mol% of NiBr_2_ and 10 mol% of **L4** with or without 3 Å MS were used

^f^20 mol% of NiCl_2_ and 10 mol% of **L4** were used

Synthesis of fluorinated heteroaromatic compounds is highly relevant to medicinal chemistry. To our delight, heteroaryl chlorides were also amenable to the current nickel-catalyzed reductive cross-coupling (Table 2). Pyridine-, quinoline-, and benzooxazole-containing substrates all underwent the reaction smoothly, leading to corresponding difluoromethylated heteroarenes in moderate to good yields (**4a-4i**).

We also examined this protocol for direct difluoromethylation of aryl chloride containing pharmaceuticals, since difluoromethyl group is considered as a bioisostere of hydroxyl and thio groups, and also as a lipophilic hydrogen bond donor^34,35^ (Table 3). Commercially available drugs such as fenofibrate, clofibrate, chlorodiphenhydramine and sibutramine underwent the current nickel-catalyzed process smoothly and afforded corresponding difluoromethylated products in good yields (**6a**–**6d**). The good tolerance of trialkyl amines of this coupling provides a useful route for the modulation of biologically active molecules. *N*-Heterocycles containing drugs were also viable in the reaction. For instance, clomipramine and buclizine furnished the desired products in good yields (**6e** and **6f**). Although lorcaserin bearing a free amine afforded low yield, the protected *N*-Boc-lorcaserin led to difluoromethylated arene efficiently (**6g**). Furthermore, *N*-heteroaryl containing drug loratadine was also applicable to the cross-coupling and provided **7h** in 82% yield (**6h**). Most importantly, the acetyl protected empagliflozin, a drug used for the treatment of type II diabetes could also provide difluoromethylated product (**6i)**. Although only 25% yield of **7i** was obtained, this strategy can trade off the yield for fast synthesis of various interesting new biologically active molecules in the late stage without the need for multi-step parallel synthesis. The rebamipide derivative with unprotected amide bond was also a competent coupling partner (**6j**). This finding encouraged us to highlight the utility of this protocol further. As shown in **6k**, the direct difluoromethylation of protic groups containing drug tolvaptan without protection of hydroxyl and amide bond produced corresponding difluoromethylated product in a yield as high as 92%. Thus, this protocol provides a synthetic simplicity route for the applications in drug discovery and development. Most remarkably, decreasing the loading amount of ClCF_2_H to 2 equiv. still provided difluoromethylated arenes with high efficiency as demonstrated by the synthesis of compounds **2a**, **2h**, **2l**, **4f** (Table 2), and **6k** (Table 3), thus demonstrating the advantages of this approach.

**Table 3:** Late-stage difluoromethylation of pharmaceuticals^a^

^a^Reaction conditions (unless otherwise specified): (hetero)aryl chloride (0.2 mmol, 1.0 equiv.), **1** (2.6 M in DMA, 6.5 equiv.), DMA (2 mL), 60 °C, 20 h. Average isolated yields from two runs

^b^20 mol% of NiBr_2_ and 10 mol% of **L4** with or without 3 Å MS were used

^c^15 mol% of NiCl_2_ and 10 mol% of **L4** were used

^d^Yield determined by ^19^F NMR using fluorobenzene as an internal standard

^e^20 mol% of NiCl_2_ and 10 mol% of **L4** were used

^f^2.0 equiv. of ClCF_2_H was used and the reaction was conducted on 3 mmol scale

**Fig. 2:**
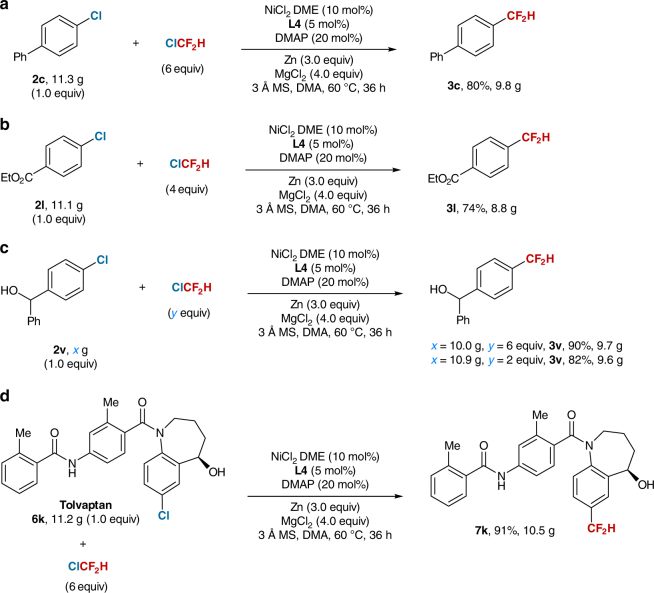
Ten-gram scale reaction of aryl chlorides with ClCF_2_H. **a** Reaction of ClCF_2_H with **2c**. **b** Reaction of ClCF_2_H with **2l**. **c** Reaction of ClCF_2_H with **2v**. **d** Reaction of ClCF_2_H with **6k**

To demonstrate the scalability of the current nickel-catalyzed process, several 10-g scale reactions of aryl chlorides were conducted. As shown in Fig. 2a, reaction of ClCF_2_H with 11.3 g of 4-chloro-1,1′-biphenyl **2c** proceeded smoothly under standard reaction conditions, providing **3c** in 80% yield. The electron-deficient aryl chloride **2l** (11.1g) was also applicable to the reaction and afforded **3l** even in a higher yield (74%) (Fig. 2b). Notably, substrate bearing a hydroxyl group (**2v**) could also furnish its corresponding difluoromethylated product **3v** in a much higher yield (90%) (Fig. 2c). Most remarkably, even 10-g scale late stage difluoromethylation of pharmaceutical tolvaptan **6k**, a high yield (91%) was still obtained (Fig. 2d). It is also worthy to note that decreasing the loading amount of ClCF_2_H to 2 equiv. could also lead to difluoromethylated arene without loss of reaction efficiency as shown by 10-g scale reaction of **2v** (Fig. 2c), thus demonstrating the good scalability and reliability of this reaction. In light of the wide existence of aryl chloride structural motif in pharmaceuticals and biologically active molecules, this approach would be useful in medicinal chemistry.

## Discussion

**Fig. 3:**
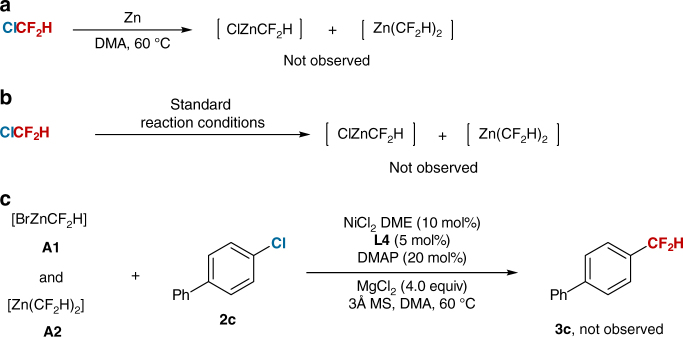
Reaction of ClCF_2_H with zinc. **a** Reaction of ClCF_2_H with zinc in DMA. **b** Reaction of ClCF_2_H with zinc under standard reaction conditions. **c** Reaction of arylchloride **2c** with difluoromethyl zinc species

In the mechanism study of this reaction, we conducted several experiments. Firstly, to rule out the possible generation of difluoromethyl zinc species^10,11^ in situ between ClCF_2_H and Zn, we performed control experiments. No difluoromethyl zinc species were observed in the reaction of ClCF_2_H with Zn in DMA at 60 °C, or even treatment of ClCF_2_H under standard reaction conditions in the absence of aryl chlorides (Fig. 3a, b). Instead, only starting material ClCF_2_H was observed after the reaction. We also prepared difluoromethyl zinc species (**A1** and **A2**) by reaction of BrCF_2_H with Zn in DMA at 60 °C (Supplementary Methods)^36^; however, no desired product **3c** was obtained when these difluoromethyl zinc species were treated with aryl chloride **2c** under standard reaction conditions (Fig. 3c). Thus, these results exclude the pathway that the formation of difluoromethylated arenes is derived from the cross-coupling between difluoromethyl zinc species and aryl chlorides. On the basis of the previous reports^28,29^, we suggest that a nickel-based, reductive cross-coupling catalytic cycle is involved in the reaction.

**Fig. 4:**
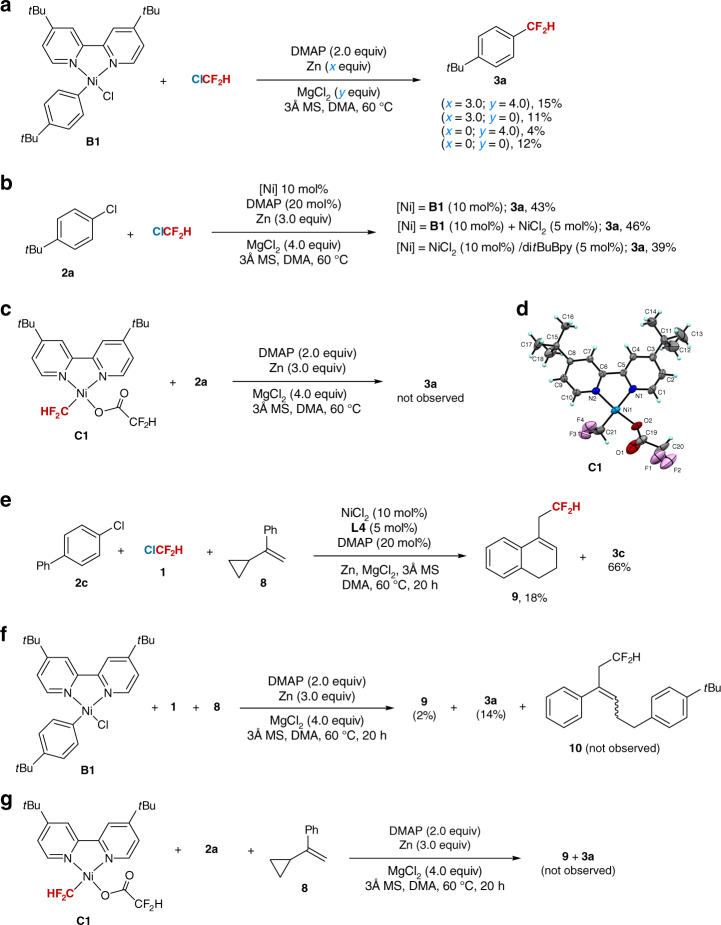
Mechanistic studies. **a** Reaction of nickel complex **B1** with ClCF_2_H. **b B1** or NiCl_2_/di*t*BuBpy catalyzed reaction of **2a** with ClCF_2_H. **c** Reaction of **C1** with **2a**. **d** X-ray crystal structure of **C1**. **e** Experiments to trap the difluoromethyl radical by reaction of **2c** and **8** with ClCF_2_H. **f** Reaction of **B1** and **8** with ClCF_2_H. **g** Reaction of **2a** and **8** with **C1**

Secondly, to identify the initiation of current reaction from aryl chloride or ClCF_2_H, we prepared aryl nickel complex [*p*-*t*Bu-PhNi(di*t*BuBpy)Cl] (**B1**)^37^ and difluoromethyl nickel complex [HCF_2_Ni(di*t*BuBpy)HCF_2_CO_2_] (**C1**)^38,39^. The structure of **C1** was confirmed by X-ray crystallographic analysis (Fig. 4d). To the best of our knowledge, the preparation of difluoromethyl nickel(II) complex has not been reported so far. The use of 4,4′-di*t*Bu-Bpy (**L2**) instead of 4,4′-diNH_2_-Bpy (**L4)** is because of the difficulties in isolation of [Ar-Ni(**L4**)-Cl] and [CF_2_H-Ni(**L4**)-HCF_2_CO_2_]. In addition, **L2** could also promote the reaction under standard reaction conditions and provided **3a** in 39% yield (Fig. 4b). A 15% yield of **3a** was provided when **B1** was treated with ClCF_2_H under standard reaction conditions (Fig. 4a). Complex **B1** could also serve as a precatalyst and provided **3a** in 43% yield, which is comparable with the yield obtained by using NiCl_2_/di*t*BuBpy catalytic system (Fig. 4b). However, no **3a** was obtained by reaction of difluoromethyl nickel complex **C1** with aryl chloride **2a** (Fig. 4c), demonstrating that the current reaction is initiated from aryl chloride and the possibility that the reaction starts from the oxidative addition of ClCF_2_H to Ni(0) is unlikely.

We also performed control experiments to gain some mechanistic insights into the reaction further (Fig. 4a). The omission of Zn led to **3a** in only 4% yield, implying that an active, low-valent nickel species is needed to promote the catalytic cycle by reduction of Ar-Ni^II^ (**B1**) with Zn. Inspired by the previous report, in which a reduction of Ar-Ni^II^ to Ar-Ni^I^ by reducing metals was proposed in the nickel-catalyzed coupling of aryl chlorides^40^, we envisioned that similar pathway may be involved in the reaction. Furthermore, a lower yield (11%) of **3a** was provided without MgCl_2_ (Fig. 4a), indicating that the presence of MgCl_2_ in current nickel-catalyzed process is probably to facilitate the reduction of nickel(II) complex by Zn to generate active nickel species. However, the exact role of MgCl_2_ remains elusive. On the other hand, the omission of Zn/MgCl_2_ could provide **3a** in 12% yield (Fig. 4a), suggesting that intermediate **B1** reduction is not needed without MgCl_2_.

Thirdly, to probe whether a difluoromethyl radical existed in the reaction, several radical trapping experiments were conducted. Radical inhibition experiments showed that the reaction could be readily inhibited by addition of electron transfer scavenger 1,4-dinitrobenzene^22^ or a radical scavenger 2,2,6,6-tetramethyl-1-piperidinyloxy (TEMPO) (Supplementary Table 16). In addition, radical clock experiment showed that a ring-expanded product **9** was formed in 18% yield when ClCF_2_H was treated with α-cyclopropylstyrene **8** in the presence of aryl chloride **2c** under standard reaction conditions (Fig. 4e). However, when a radical scavenger TEMPO was added to the reaction, the reaction was totally inhibited without observation of compounds **9** and **3c** (Supplementary Methods). Compound **9** could also be obtained by a stoichiometric reaction of nickel complex **B1** with ClCF_2_H and **8** under standard reaction conditions (Fig. 4f). But difluoromethyl nickel complex **C1** failed to provide compound **9** (Fig. 4g), thus ruling out the possible formation of compound **9** from **C1** through the Ni-concerted insertion mechanism (Supplementary Fig. 142b). Furthermore, the possibility of formation of compound **9** from aryldifluoromethyl nickel complex Ar-[Ni]-CF_2_H generated in situ between **B1** and ClCF_2_H is also unlikely, as no ring-opening product **10** was observed by treatment of **B1** with ClCF_2_H and **8** (Fig. 4f and Supplementary Fig. 142c). Therefore, these results suggest that the formation of **9** via a radical pathway (Supplementary Fig. 142a) is reasonable and a difluoromethyl radical species is involved in current catalytic cycle.

**Fig. 5:**
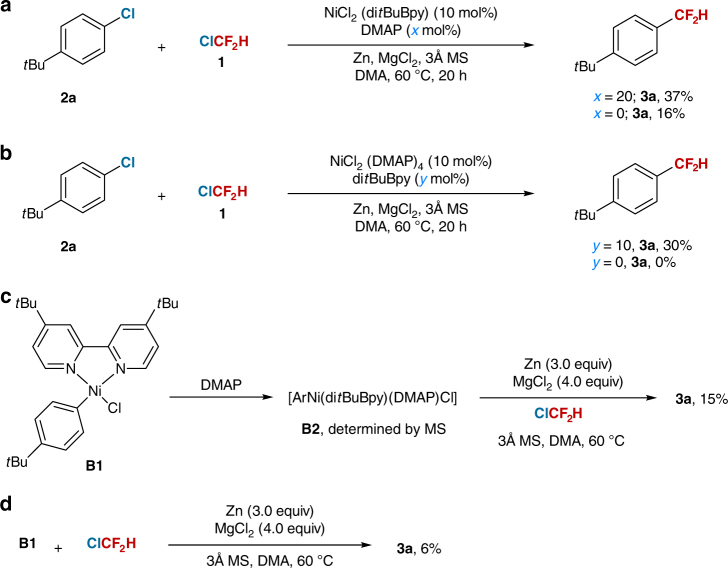
The role of DMAP. **a** [NiCl_2_(di*t*BuBpy)] (**D1**) catalyzed reaction between **2a** and **1** with or without DMAP. **b** [NiCl_2_(DMAP)_4_] (**D2**) catalyzed reaction between **2a** and **1** with or without di*t*BuBpy. **c** Reaction of **B1** with ClCF_2_H in the presence of DMAP. **d** Reaction of **B1** with ClCF_2_H without DMAP

Finally, to establish the role of DMAP in the reaction, we prepared nickel complexes [NiCl_2_(di*t*BuBpy)] (**D1**) and [NiCl_2_(DMAP)_4_] (**D2**)^27^. Both of them could serve as precatalysts and provided **3a** in comparable yields (Fig. 5a, b). However, **D1** provided **3a** in a lower yield (16%) without DMAP (Fig. 5a) and no **3a** was observed by using **D2** in the absence of di*t*BuBpy (Fig. 5b). Additionally, a DMAP coordinated nickel complex **B2** was observed by reaction of **B1** with DMAP, which could also produce **3a** in a 15% yield (Fig. 5c). But only 6% yield of **3a** was obtained without DMAP (Fig. 5d). These results demonstrate that DMAP can function as a co-ligand to coordinate to the nickel center and thus facilitate the catalytic cycle.

**Fig. 6:**
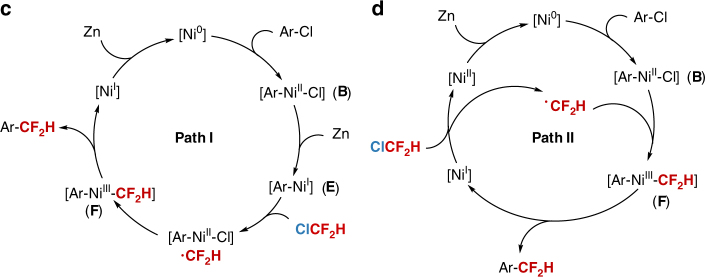
Proposed reaction mechanism. **a** Proposed mechanism via a radical-cage-rebound process. **b** Proposed mechanism via a radical chain process

We also performed a Hammett-type analysis of the reaction (Supplementary Fig. 137 and 138). Plots of log(*k*_rel_) versus *σ* and *σ*(−) were linear with moderate quality (*R*^2^ ∼ 0.93), and the slope (*ρ*) was between 1.9 and 2.0^30,41,42^. The *ρ* values are smaller than those reports on stoichiometric studies of the oxidative addition of aryl halides to Ni (4.4–8.8)^43,44^, indicating that the oxidative addition of aryl chlorides to Ni(0) is not rate-determining step^45^. On the basis of these results and previous reports^28,29^, we propose that the reaction starts from aryl chloride via a radical-cage-rebound process^29^ (Fig. 6a). An oxidative addition of aryl chloride to Ni(0) initiates the reaction. Subsequently, the resulting nickel(II) complex [Ar-Ni^II^-Cl] (**B**) is reduced by Zn to generate [Ar-Ni^I^] (**E**). **E** undergoes the second oxidative addition with ClCF_2_H to produce [Ar-Ni^III^-CF_2_H] (**F**) through a cage rebound process, in which a difluoromethyl radical ·CF_2_H is produced via a single-electron-transfer pathway, subsequently, the resulting ·CF_2_H rapidly recombines with [Ar-Ni^II^-Cl] to give **F**. Finally, **F** undergoes reductive elimination to deliver difluoromethylated arenes and [Ni^I^]. [Ni^I^] is further reduced by Zn to regenerate [Ni^0^]. Alternatively, a radical chain mechanism^28,46^ is also possible (Fig. 6b) as the intermediate **B1** could also lead to difluoromethylated product without reduction by Zn (Fig. 4a). In this pathway, the ·CF_2_H generated by reaction of [Ni^I^] with ClCF_2_H diffuses to the solution to combine with [Ar-Ni^II^-Cl] **B** to produce **F**, which undergoes reductive elimination to give difluoromethylated product. Finally, the resulting [Ni^II^] is reduced by Zn to regenerate [Ni^0^] (Supplementary Fig. 144).

In conclusion, we have developed a practical nickel-catalyzed difluoromethylation of (hetero)aryl chlorides and bromides with abundant and inexpensive ClCF_2_H, representing a new strategy for fluoroalkylation reactions. The reaction proceeds under mild reaction conditions and can efficiently access a wide range of difluoromethylated (hetero)aromatics, including pharmaceuticals. Comparing to the previous difluoromethylation methods^3–12,17^, the current nickel-catalyzed process features several advantages, inexpensive ClCF_2_H and low-cost nickel catalyst; more accessible and cheaper aryl chlorides as well as no need for preformed arylmetals; broad substrate scope including a variety of heteroaromatics and pharmaceuticals; synthetic simplicity and convenience without prefunctionalization of drugs and biologically active molecules. Particularly, the ability of direct modulation of pharmaceuticals by using ClCF_2_H provides good opportunities to discover new medicinal agents. The additives MgCl_2_ and DMAP are critical to the reaction efficiency and DMAP can serve as a co-ligand to facilitate the catalytic cycle. Preliminary mechanistic studies reveal that the reaction starts from the oxidative addition of aryl halides to Ni(0) and a difluoromethyl radical is involved in the reaction, which is in contrast to the previous difluorocarbene pathway, thus paving a new way for applications of ClCF_2_H in organic synthesis and related chemistry.

## Methods

### General procedure for the nickel catalyzed cross-coupling

To a 25 mL of Schlenk tube were added aryl chloride **2**,** 4**, or **6** (0.2 mmol, 1.0 equiv.), NiCl_2_ (10 mol%), **L4** (5 mol%), zinc dust (3.0 equiv.), MgCl_2_ (4.0 equiv.), 3 Å MS (100 mg) and DMAP (20 mol%). The mixture was evacuated and backfilled with argon for three times, DMA (2 mL) and ClCF_2_H **1** (2.6 M in DMA, 1.3 mmol, 6.5 equiv.) were then added. The Schlenk tube was screw capped and put into a preheated oil bath (60 °C). After stirring for 20 h, the reaction mixture was cooled to room temperature and diluted with ethyl acetate (2 mL). The yield was determined by ^19^F NMR using fluorobenzene as an internal standard before working up. Then the reaction mixture was filtered with a pad of cellite. The filtrate was washed with brine, extracted with EtOAc for three times. Then the organic layer was dried over Na_2_SO_4_ and concentrated. The residue was purified with silica gel chromatography to give product **3**, **5**, or **7**. Isolated yield is based on the average of two runs under identical conditions.

### Data availability

The authors declare that all the data supporting the findings of this study are available within the paper and its supplementary information files. CCDC 1572871 contains the supplementary crystallographic data for **C1**. These data can be obtained free of charge from the Cambridge Crystallographic Data Centre via www.ccdc.cam.ac.uk/data_request/cif.

## Electronic supplementary material

Supplementary Information(PDF 9012 kb)

Description of Additional Supplementary Files(PDF 165 kb)

Peer Review File(PDF 641 kb)

Supplementary Data 1(CIF 595 kb)

Description for the revised Supplementary Information(PDF 147 kb)
