# Supplementary material for: Difluoromethylation of (hetero)aryl chlorides with chlorodifluoromethane catalyzed by nickel
Source: Nat Commun. 2018 Mar 21;9:1170. doi: 10.1038/s41467-018-03532-1 (PMC5862906; doi:10.1038/s41467-018-03532-1)
Supplement: Supplementary file 1 — Supplementary Information(PDF 9012 kb) [file 41467_2018_3532_MOESM1_ESM.pdf]

Difluoromethylation of (Hetero)Aryl Chlorides with Chlorodifluoromethane Catalyzed by Nickel

Xu *et al.*

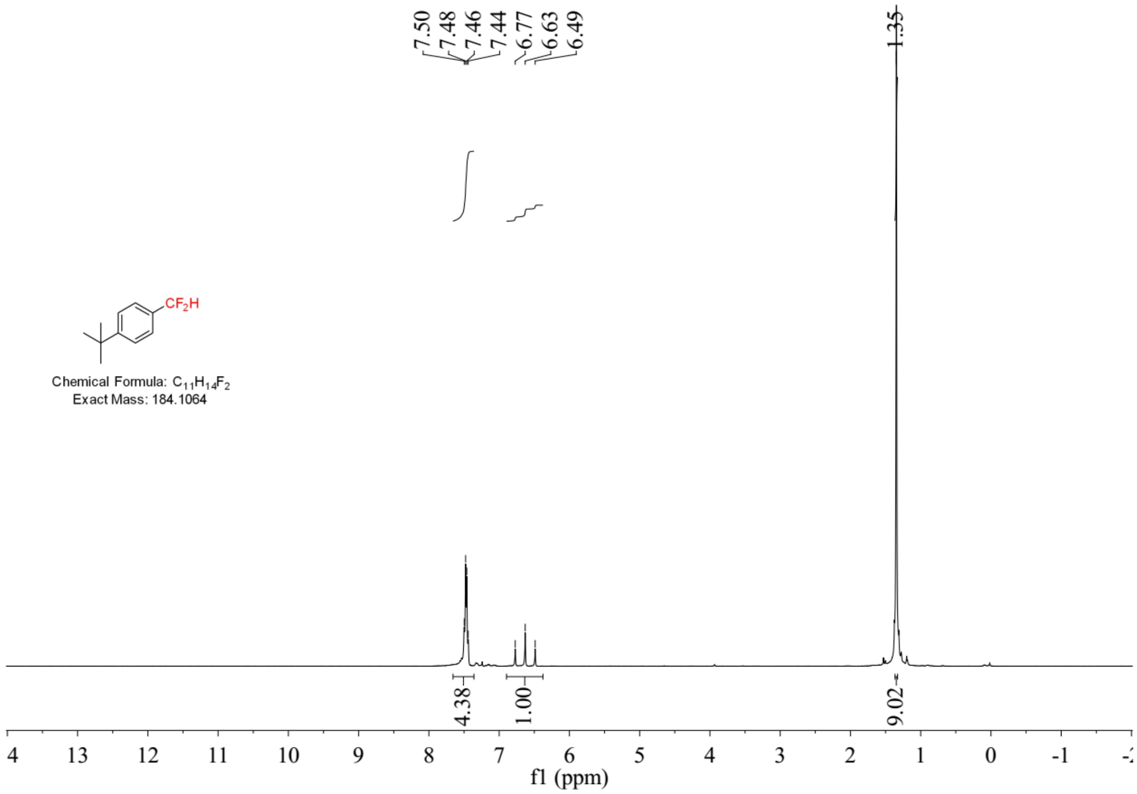

Supplementary Figure 1. ^{1}**H NMR of 1-(*tert*-butyl)-4-(difluoromethyl)benzene (3a)**

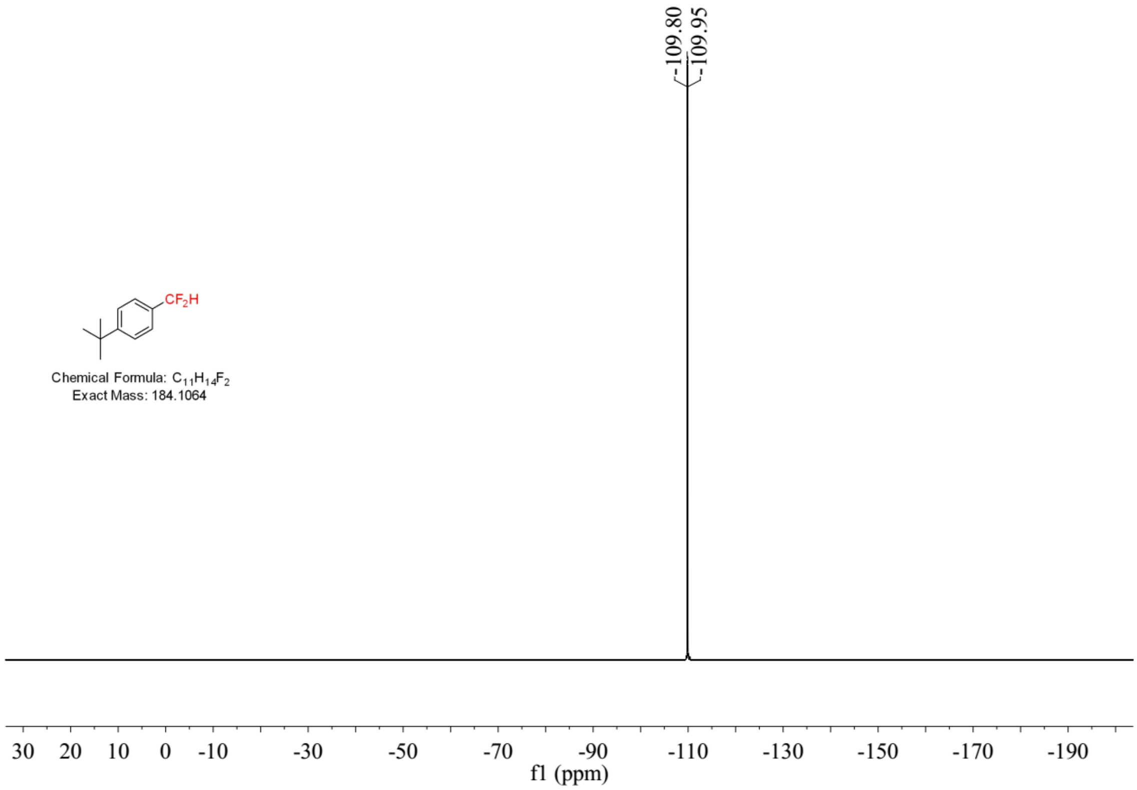

Supplementary Figure 2. ^{19}**F NMR of 1-(*tert*-butyl)-4-(difluoromethyl)benzene (3a)**

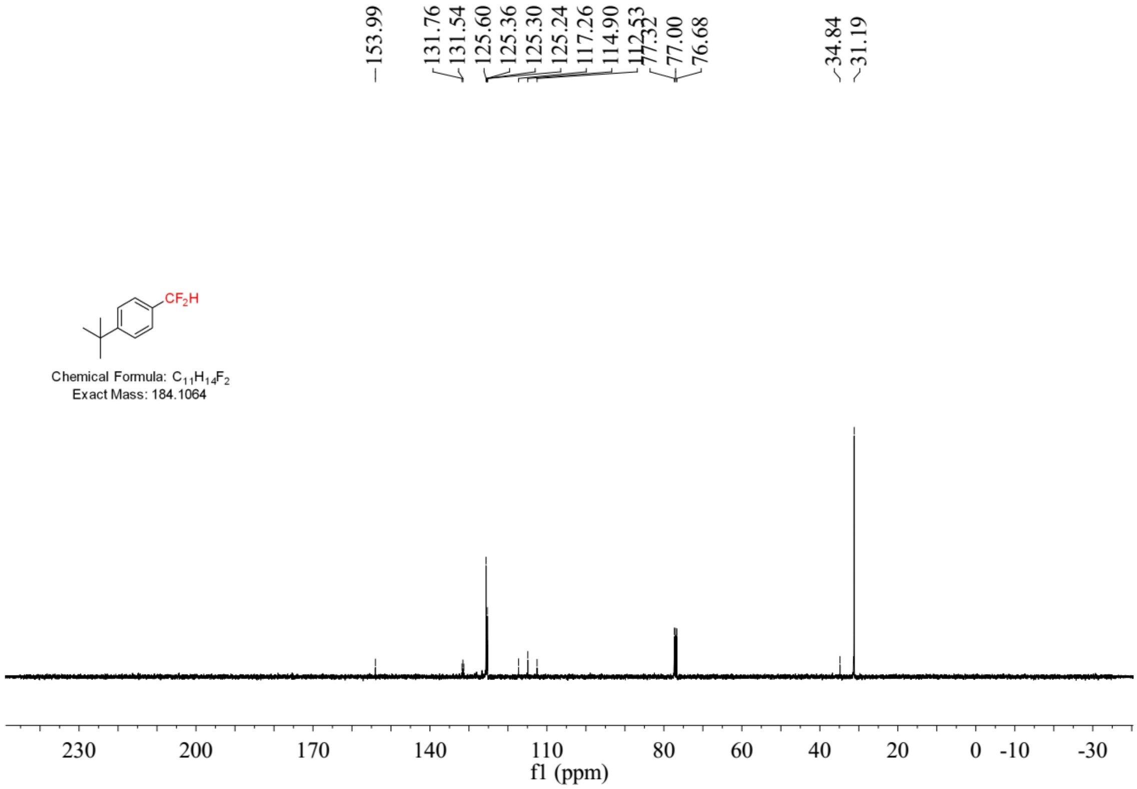

Supplementary Figure 3. ^{13}C NMR of 1-(*tert*-butyl)-4-(difluoromethyl)benzene (3a)

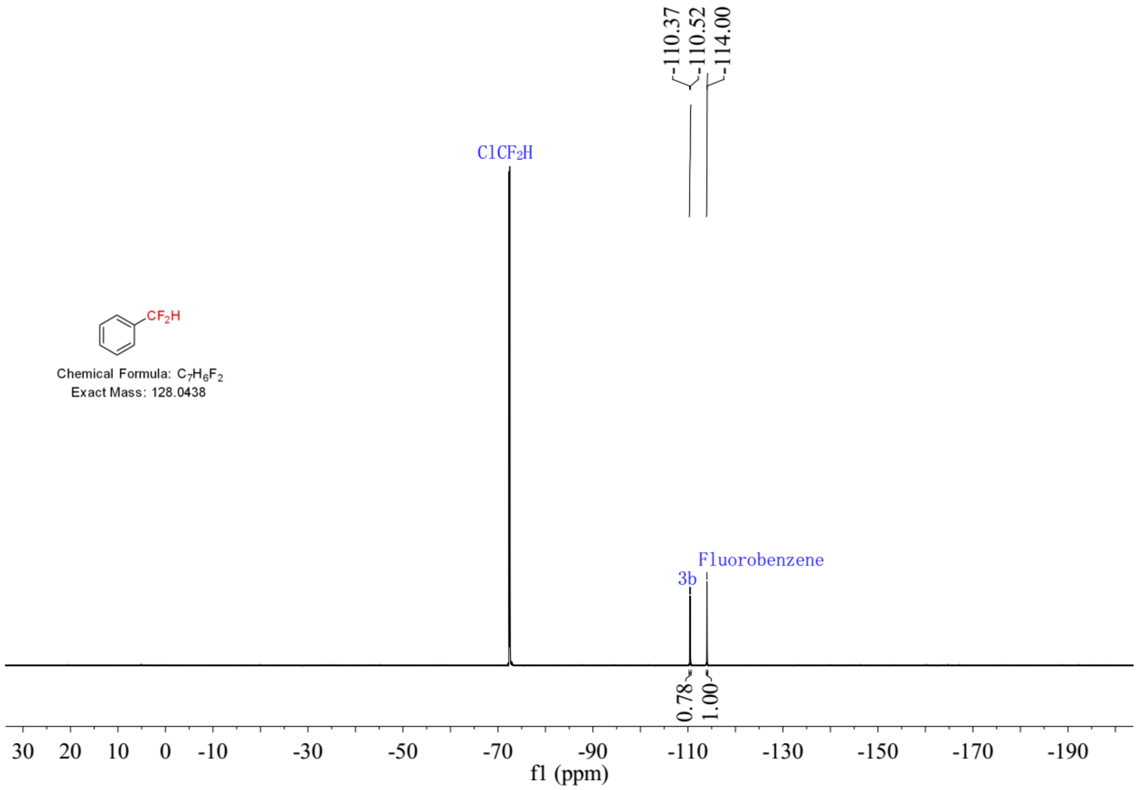

Supplementary Figure 4. Crude ^{19}F NMR of (difluoromethyl)benzene (3b)

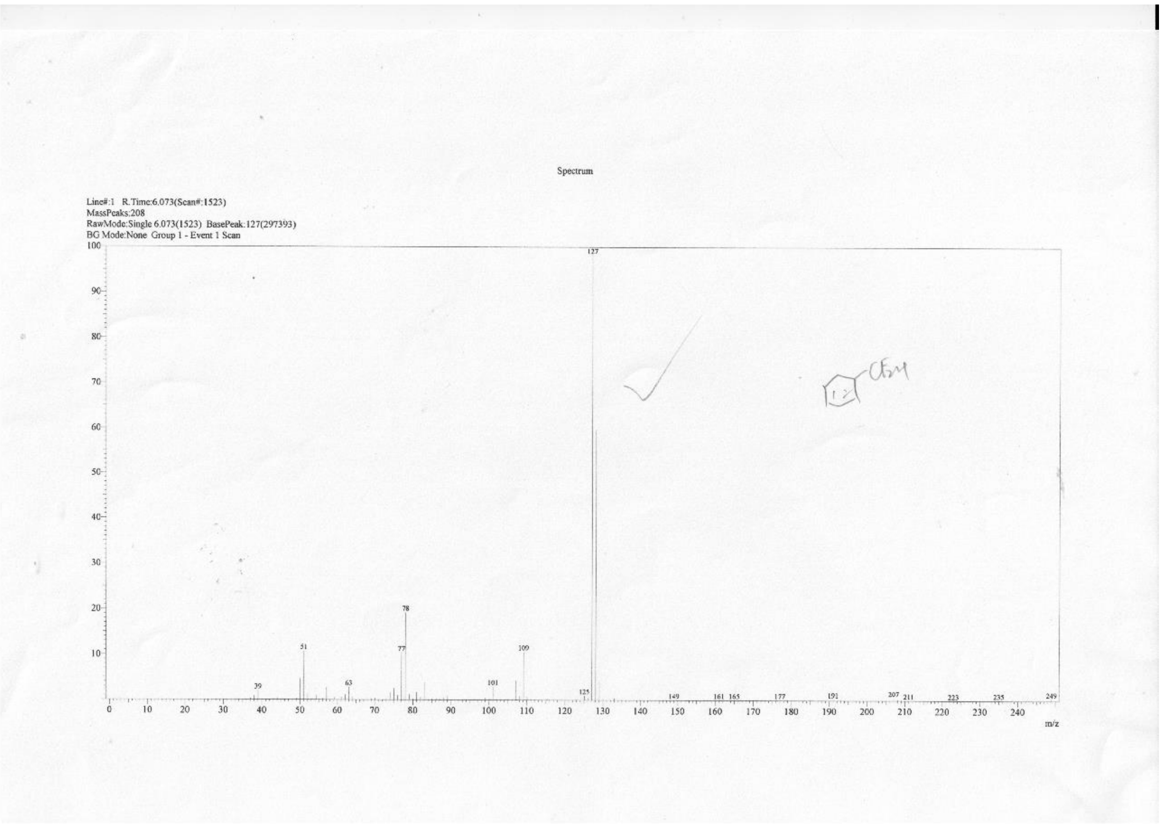

Supplementary Figure 5. MS(EI) of (difluoromethyl)benzene (3b)

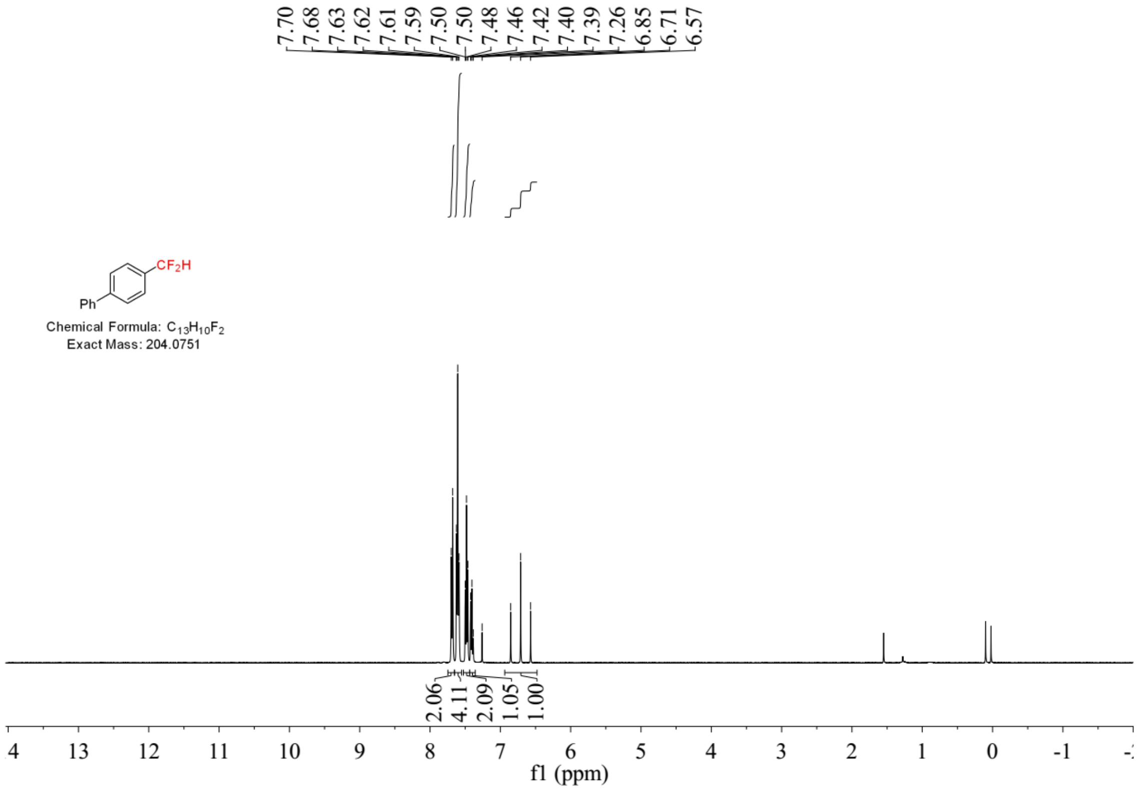

Supplementary Figure 6. ^{1}H-NMR of 4-(difluoromethyl)-1,1'-biphenyl (3c)

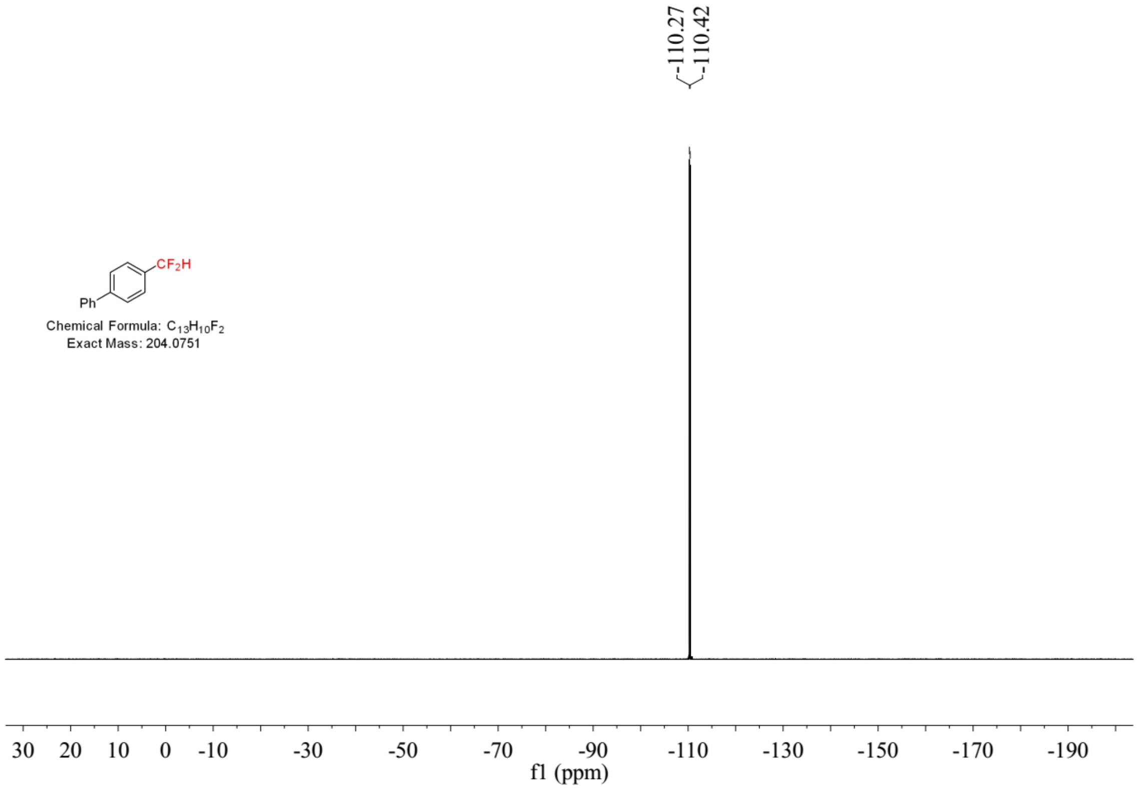

Supplementary Figure 7. ^{19}F-NMR of 4-(difluoromethyl)-1,1'-biphenyl (3c)

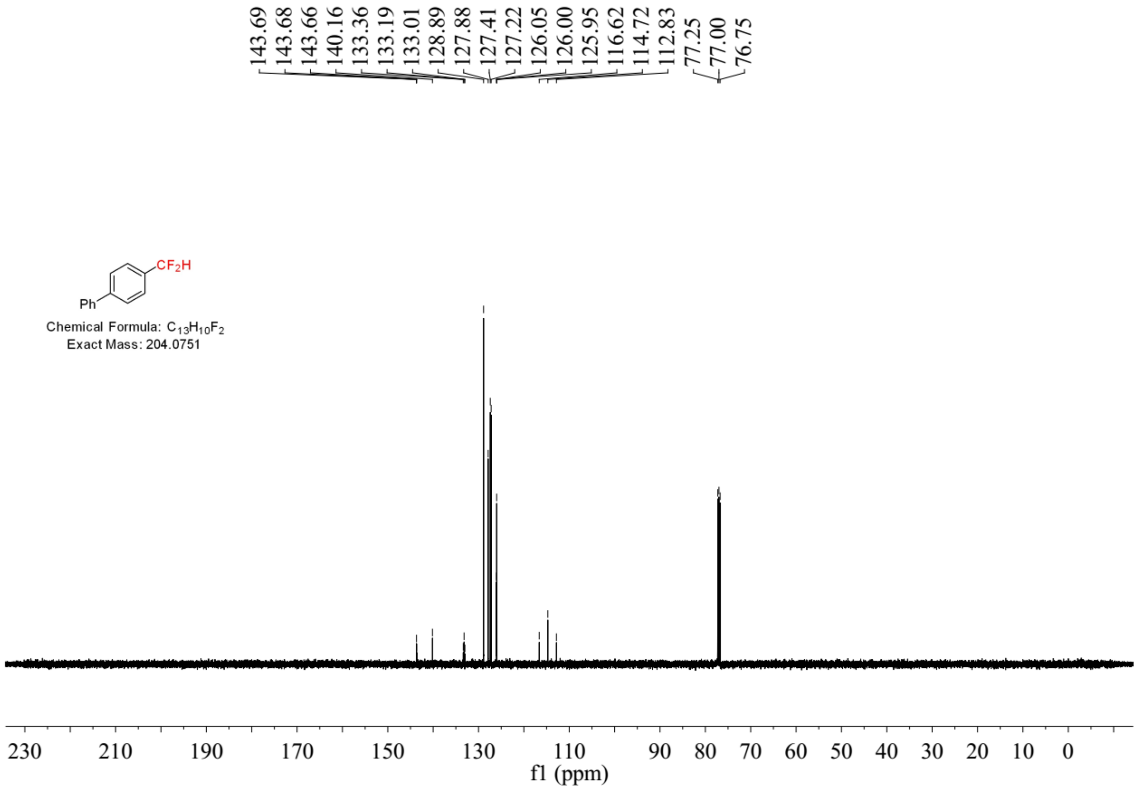

Supplementary Figure 8. ^{13}C-NMR of 4-(difluoromethyl)-1,1'-biphenyl (3c)

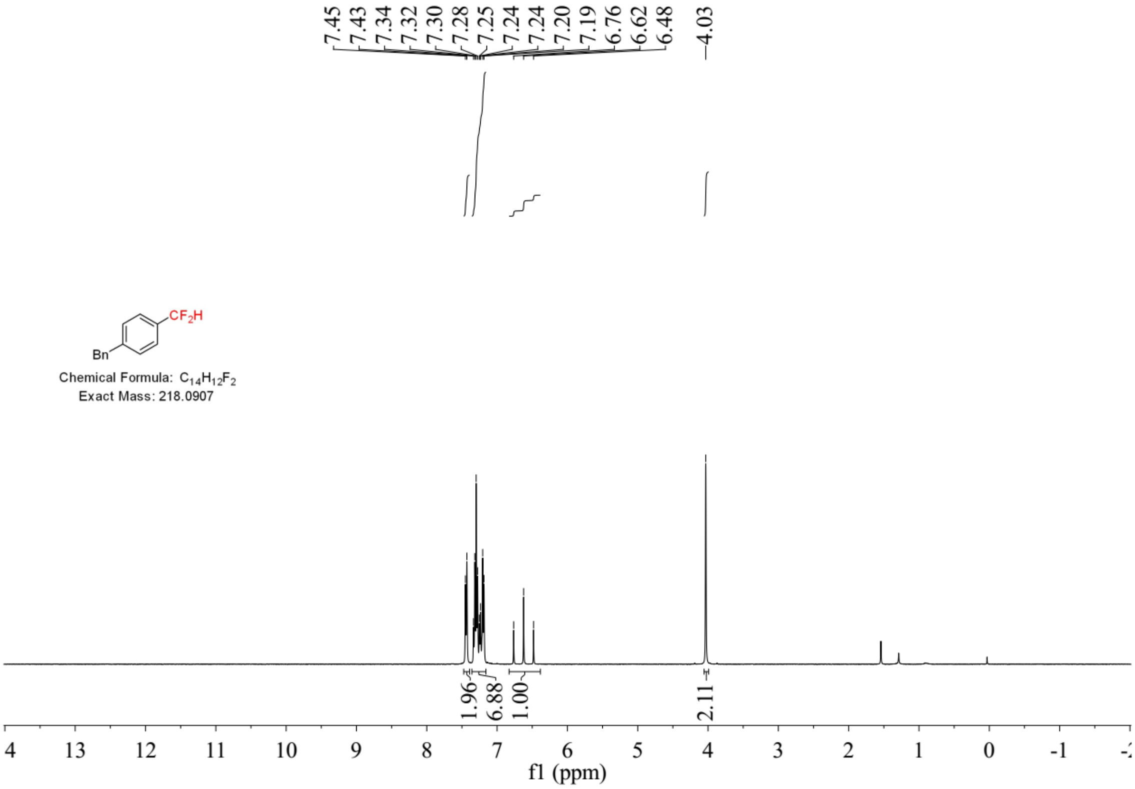

Supplementary Figure 9. ^{1}H-NMR of 1-benzyl-4-(difluoromethyl)benzene (3d)

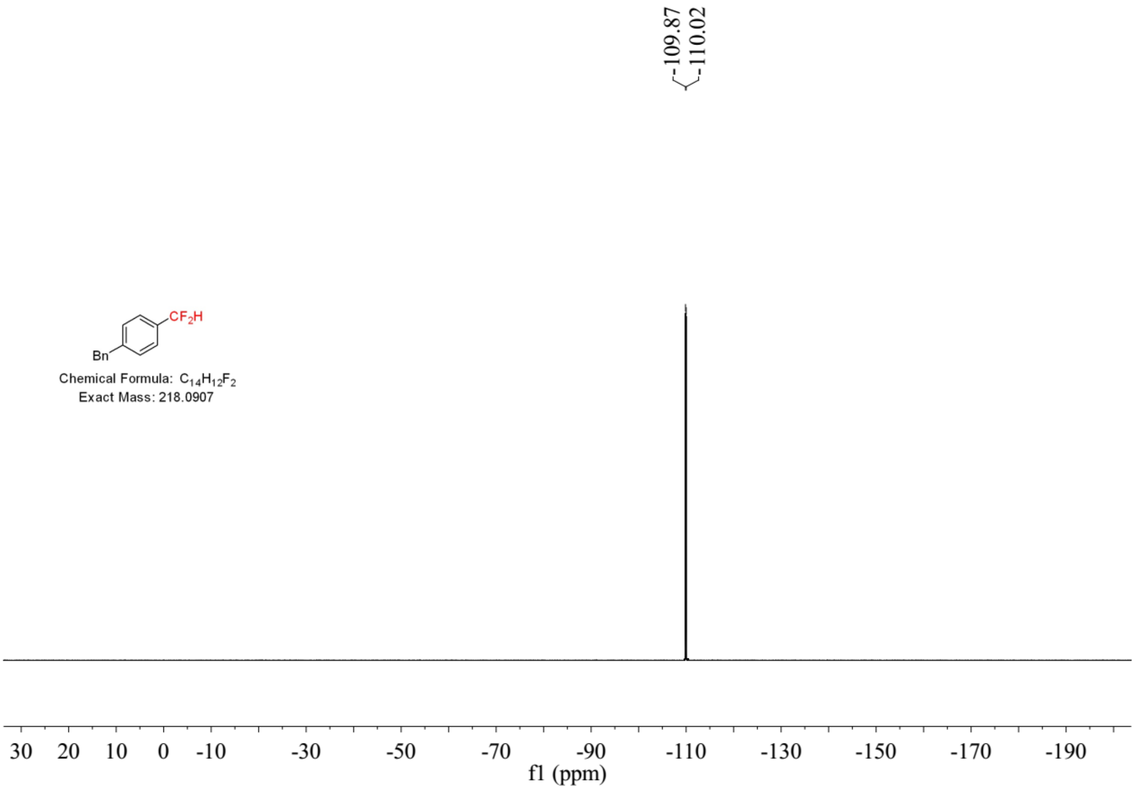

Supplementary Figure 10. ^{19}F-NMR of 1-benzyl-4-(difluoromethyl)benzene (3d)

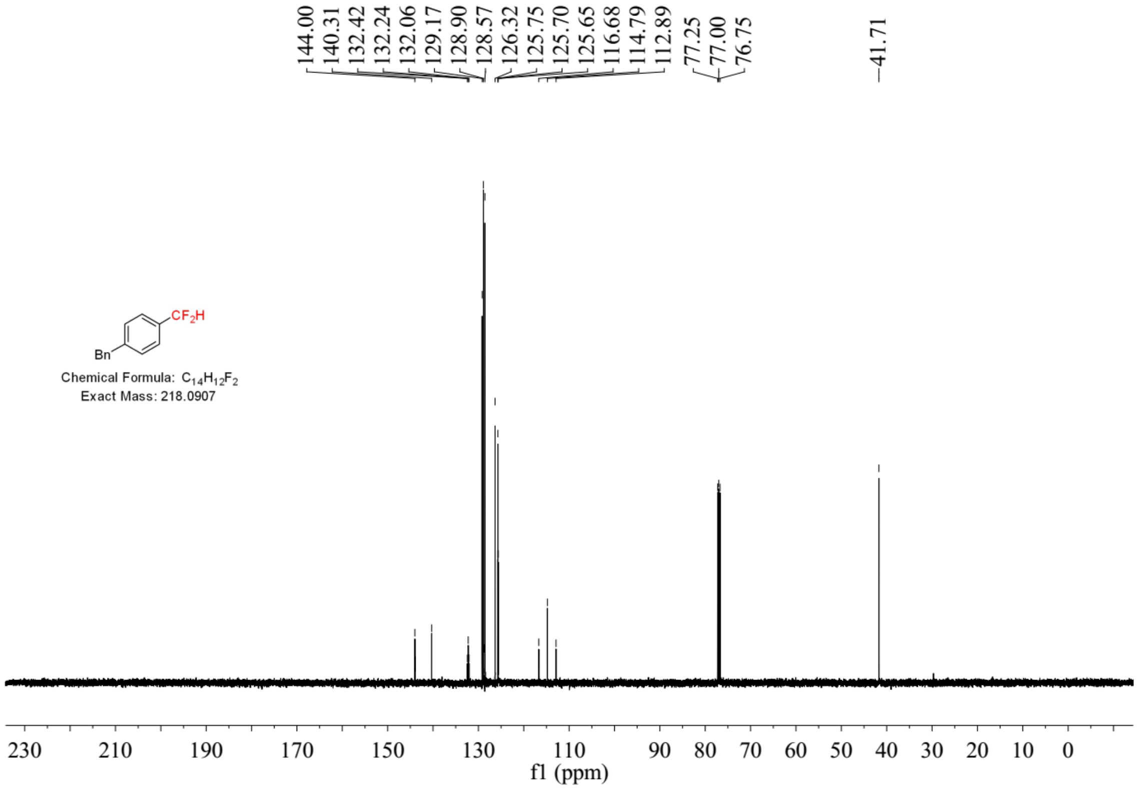

Supplementary Figure 11. ^{13}**C-NMR of 1-benzyl-4-(difluoromethyl)benzene (3d)**

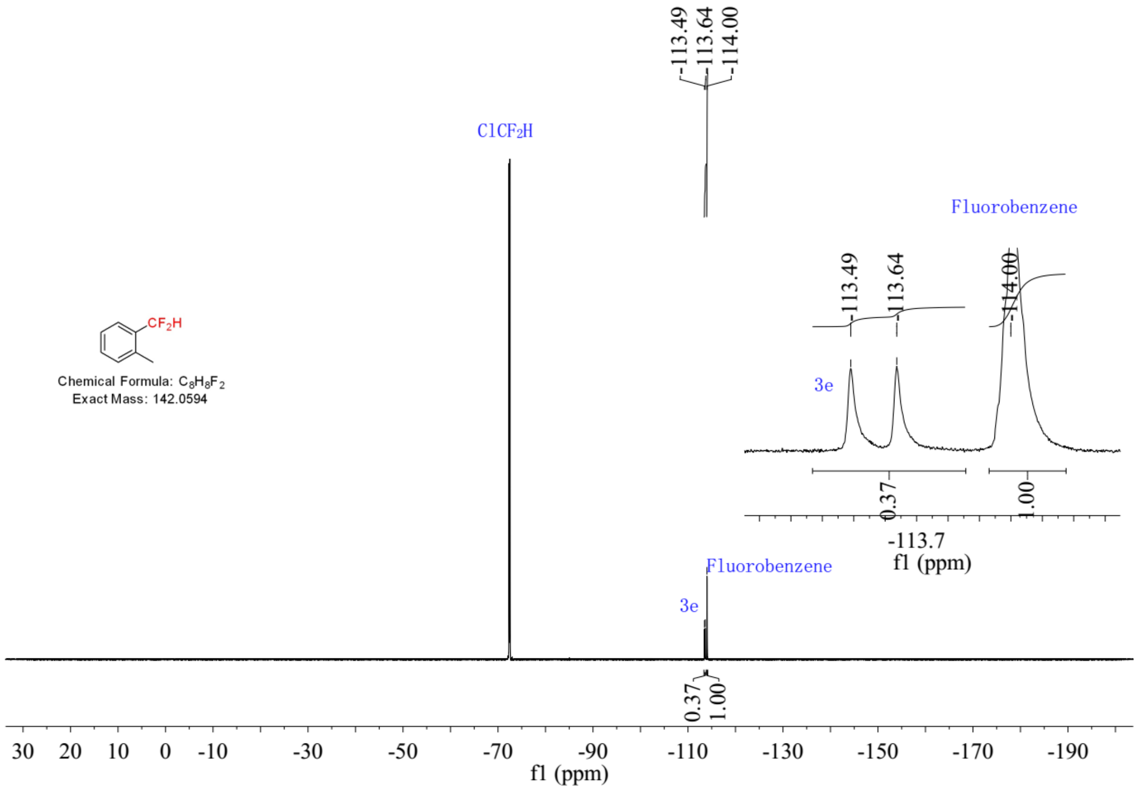

Supplementary Figure 12. Crude ^{19}**F NMR of 1-(difluoromethyl)-2-methylbenzene (3e)**

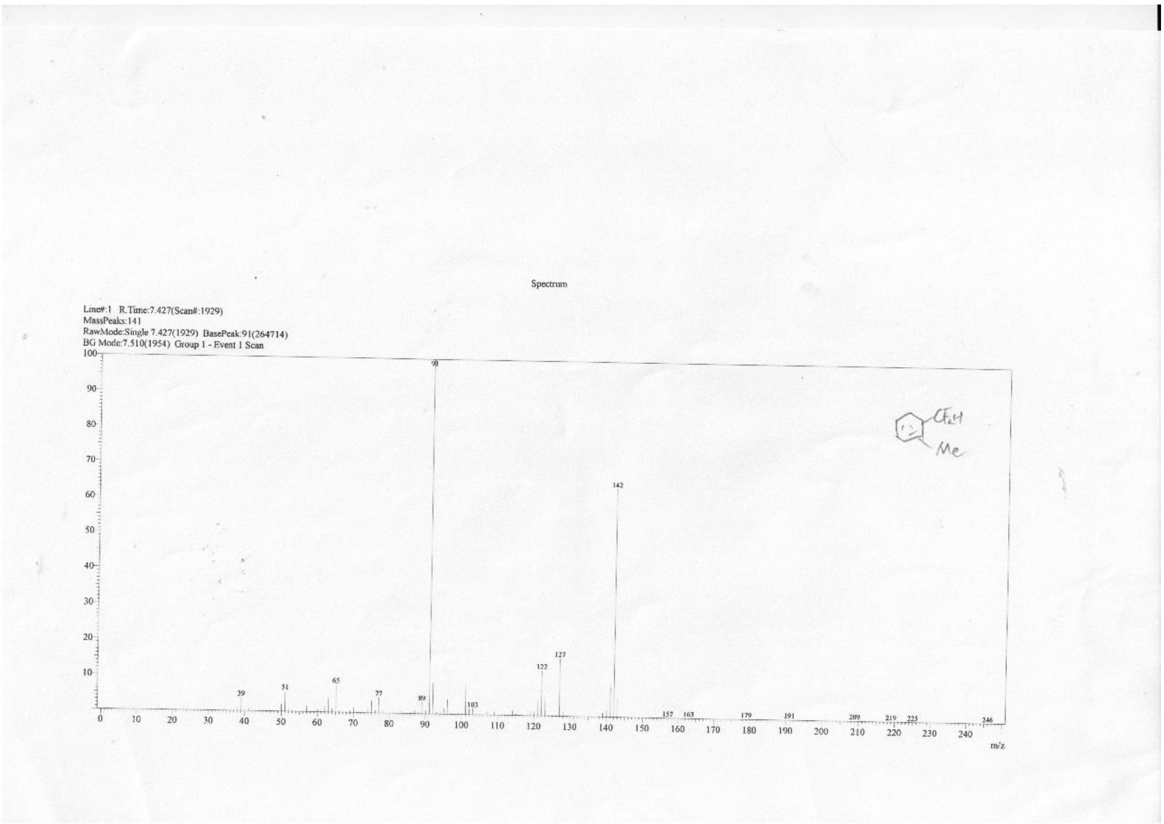

Supplementary Figure 13. MS(EI) of 1-(difluoromethyl)-2-methylbenzene (3e)

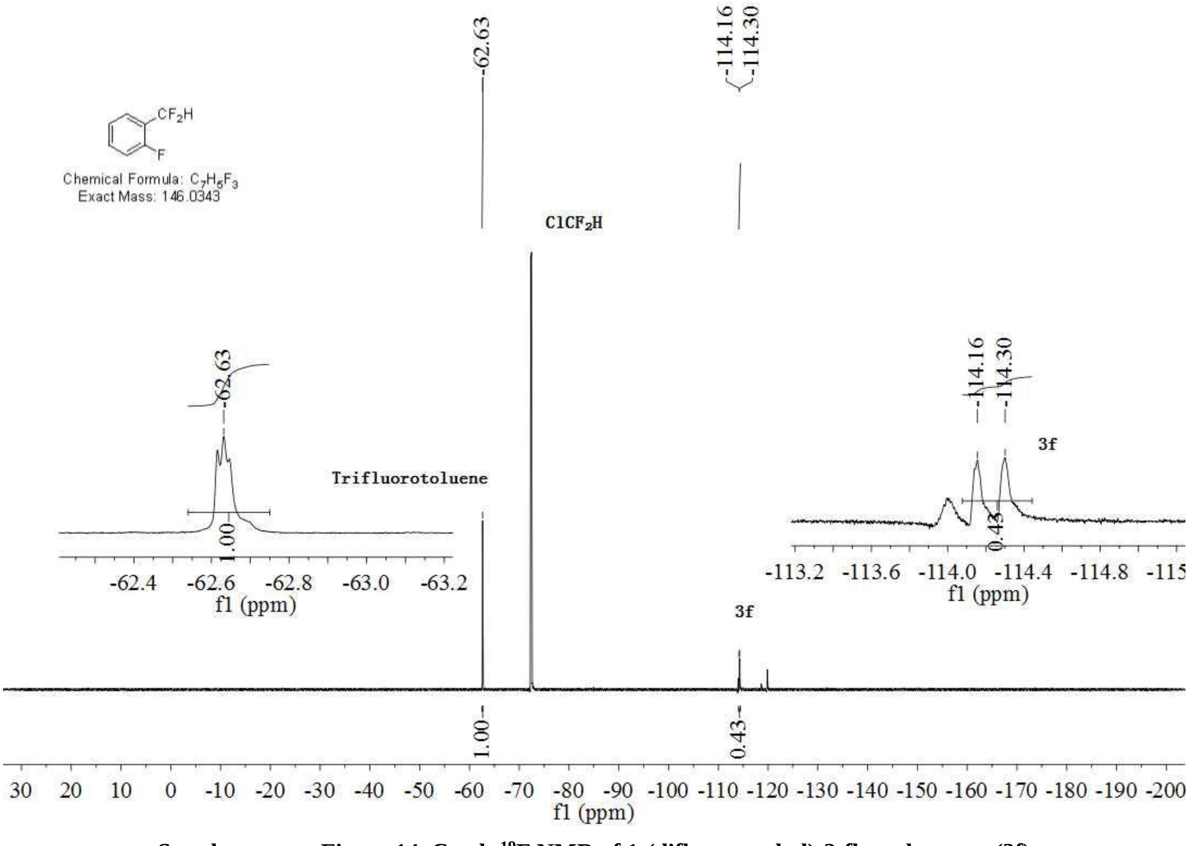

Supplementary Figure 14. Crude ^{19}F NMR of 1-(difluoromethyl)-2-fluorobenzene (3f)

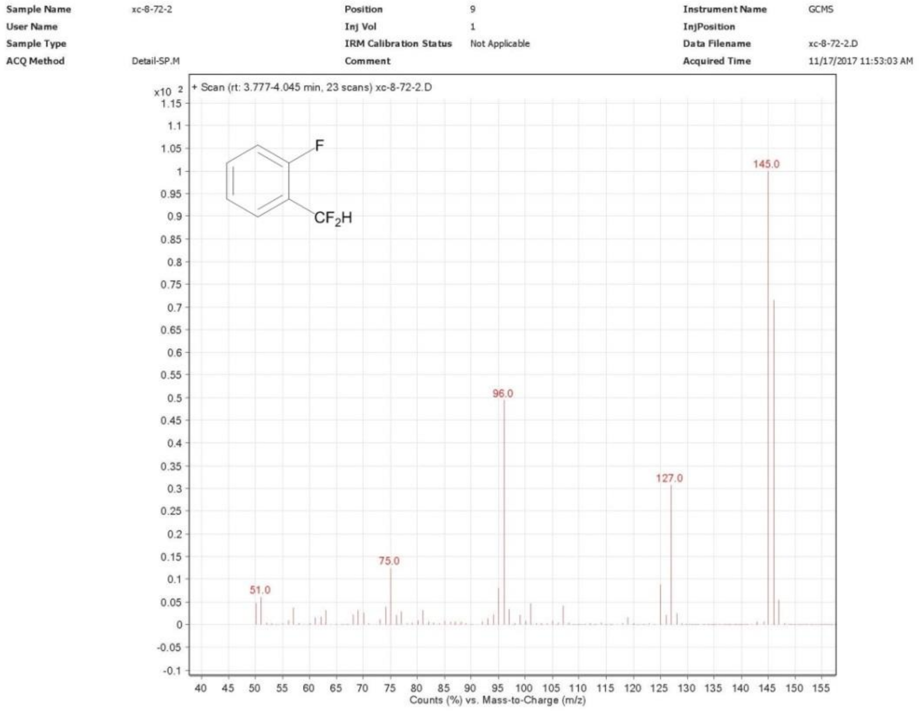

Supplementary Figure 15. MS(EI) of 1-(difluoromethyl)-2-fluorobenzene (3f)

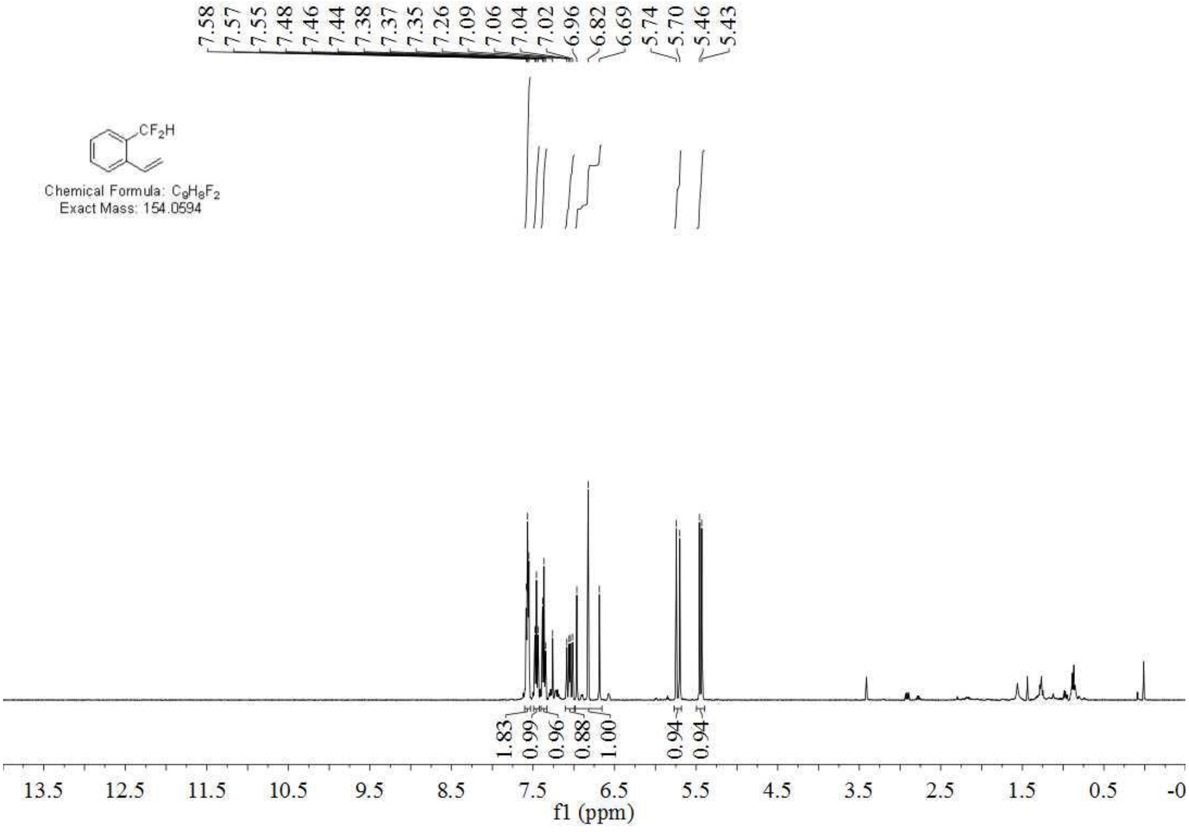

Supplementary Figure 16. ^{1}H-NMR of 1-(difluoromethyl)-2-vinylbenzene (3g)

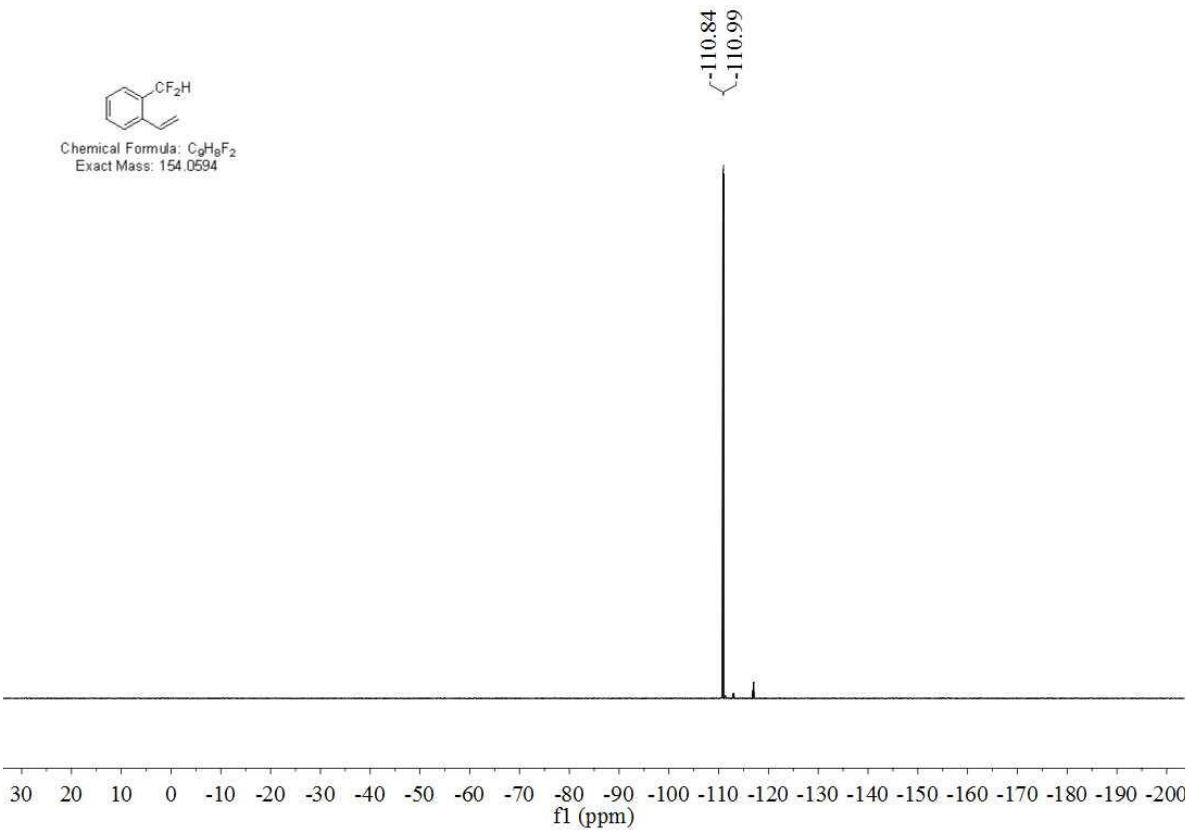

Supplementary Figure 17. ^{19}F-NMR of 1-(difluoromethyl)-2-vinylbenzene (3g)

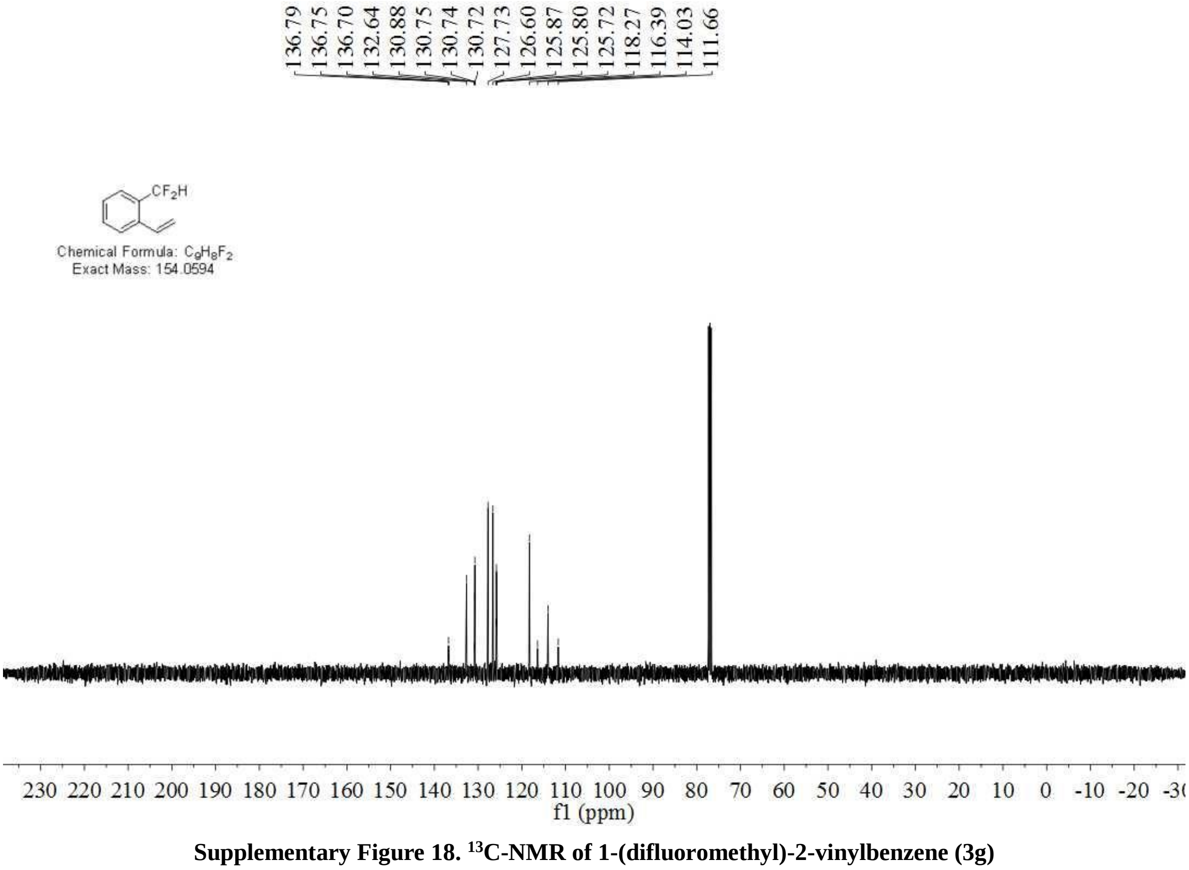

Supplementary Figure 18. ^{13}C-NMR of 1-(difluoromethyl)-2-vinylbenzene (3g)

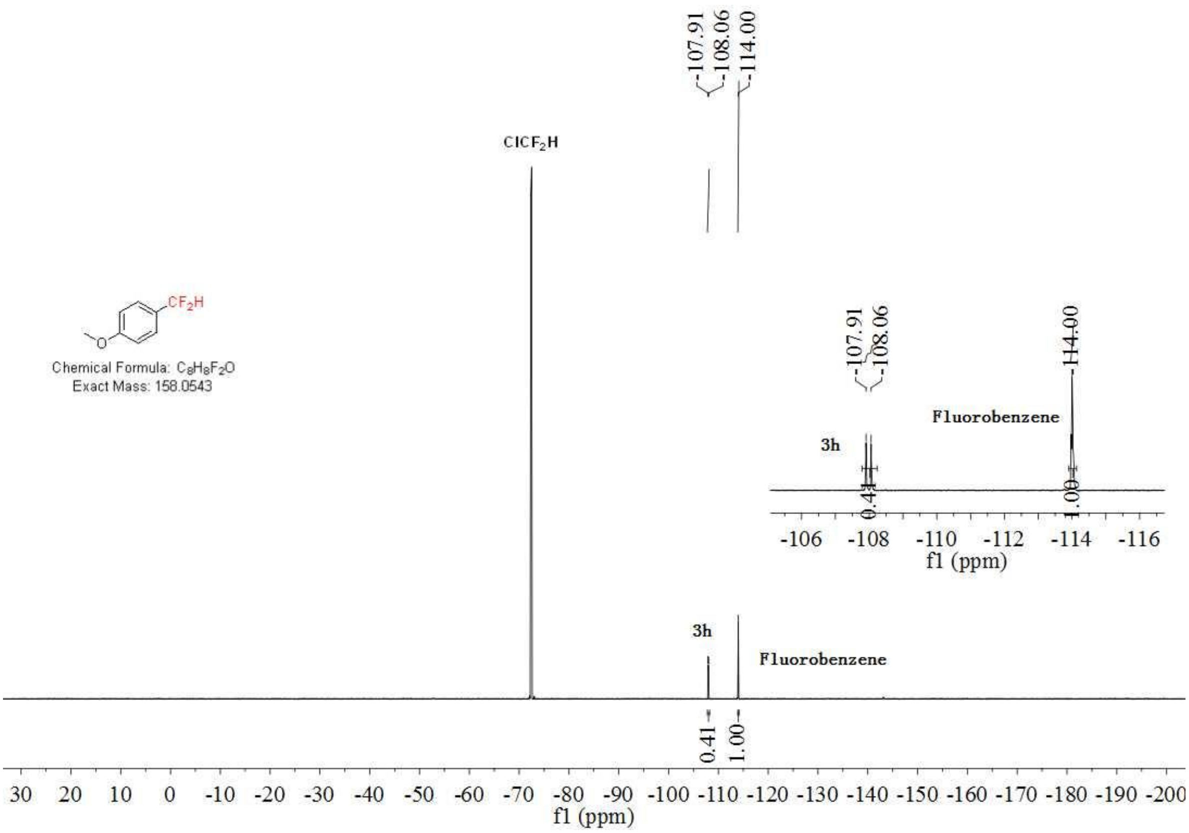

Supplementary Figure 19. Crude ^{19}F NMR of 1-(difluoromethyl)-4-methoxybenzene (3h)

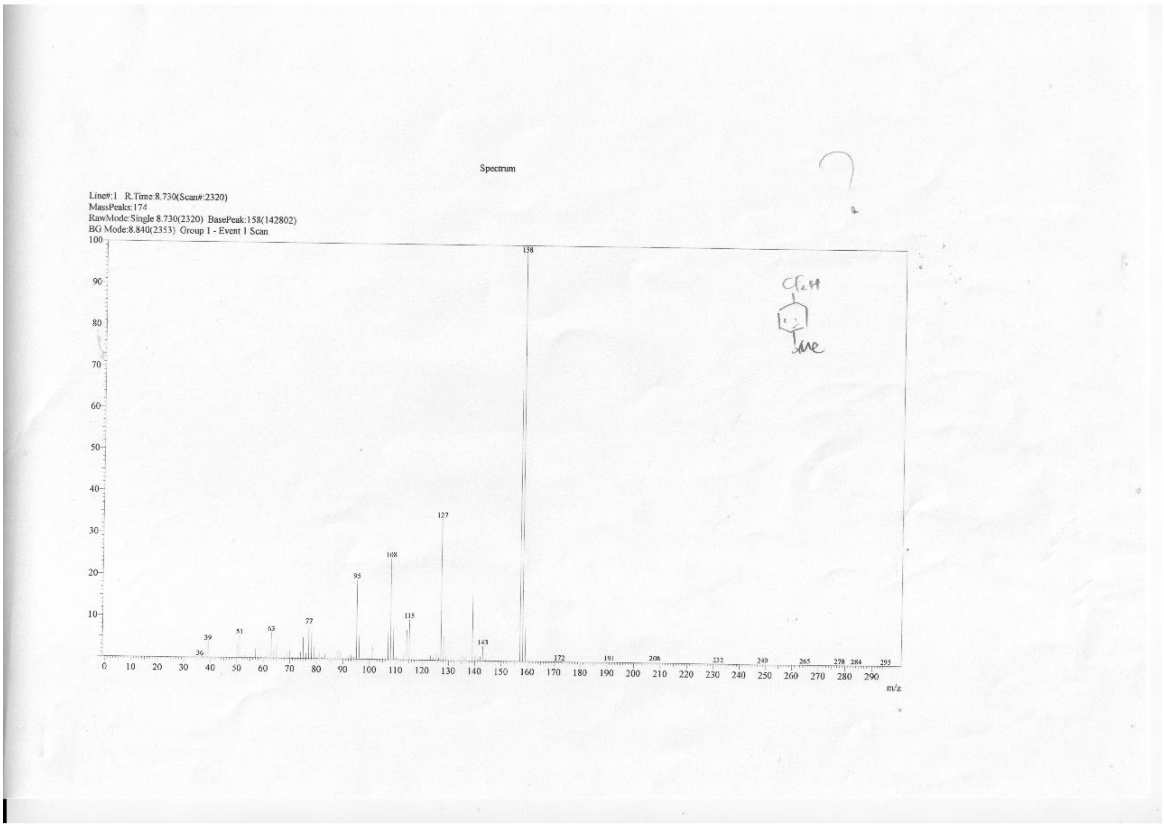

Supplementary Figure 20. MS(EI) of 1-(difluoromethyl)-4-methoxybenzene (3h)

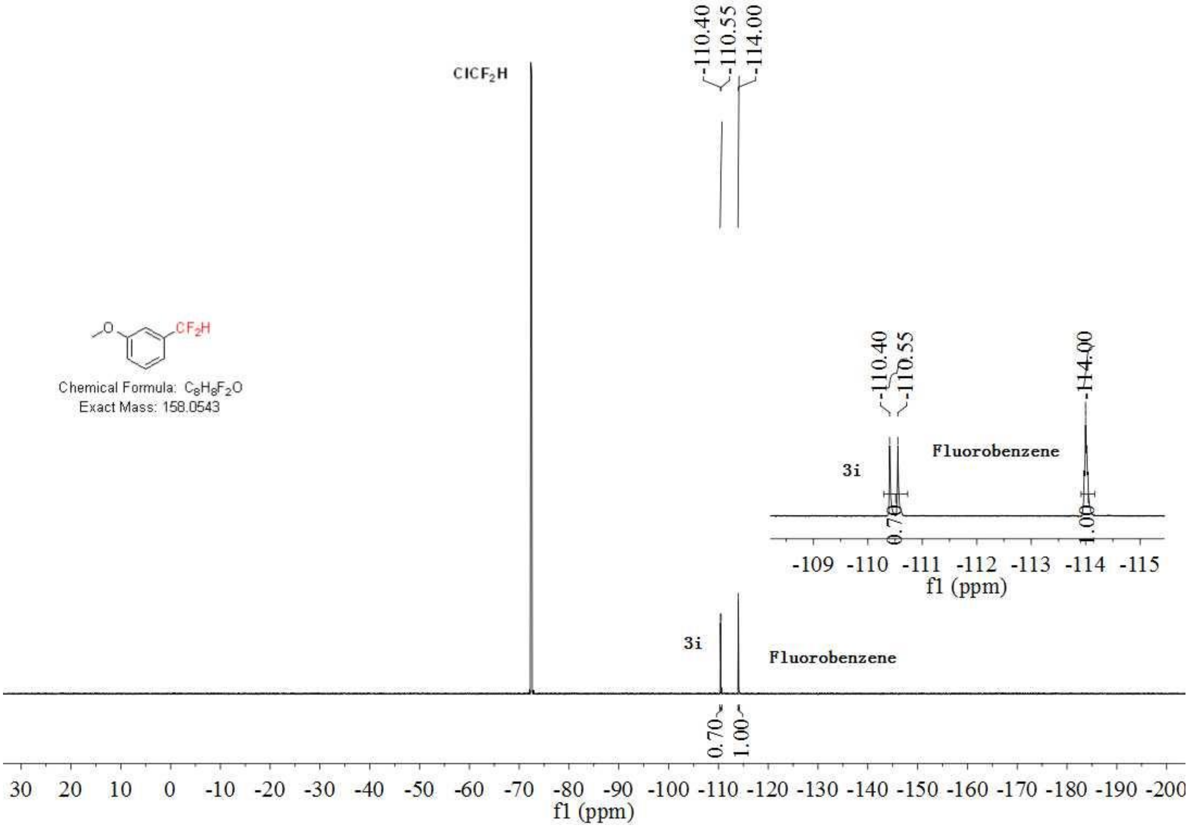

Supplementary Figure 21. Crude ^{19}F NMR of 1-(difluoromethyl)-3-methoxybenzene (3i)

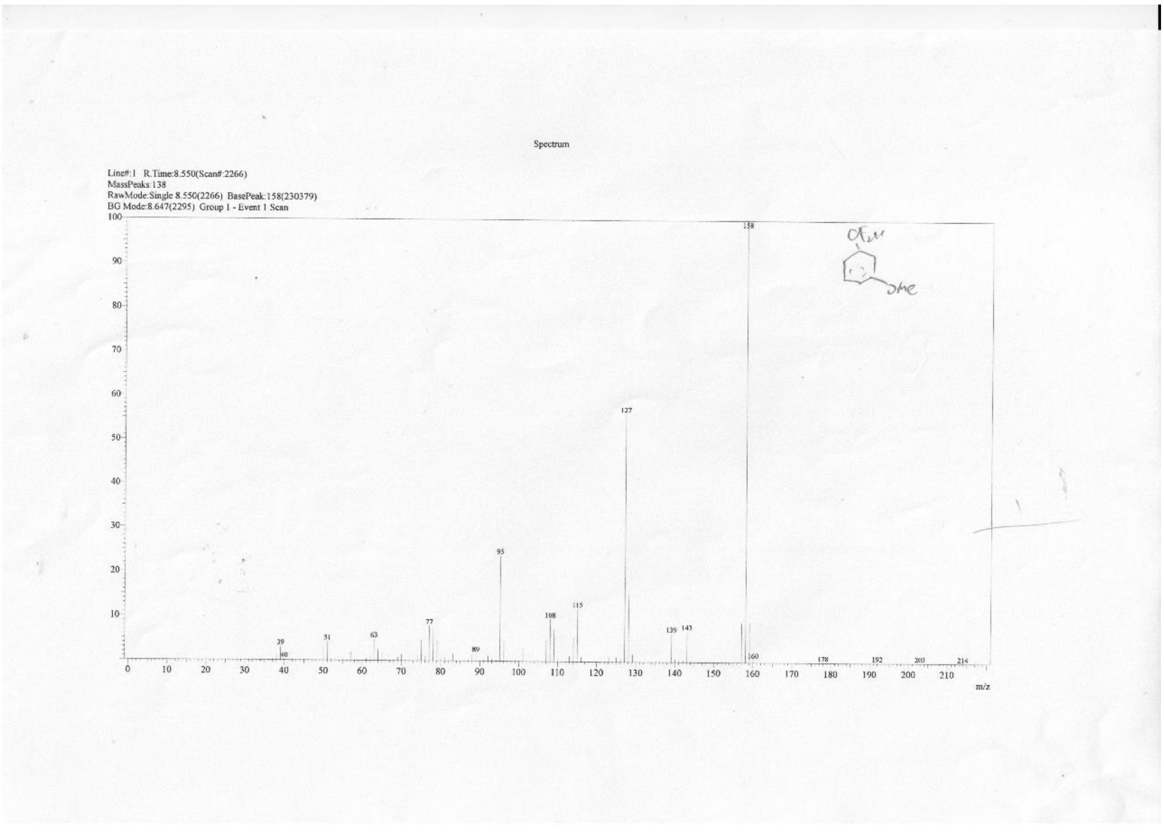

Supplementary Figure 22. MS(EI) of 1-(difluoromethyl)-3-methoxybenzene (3i)

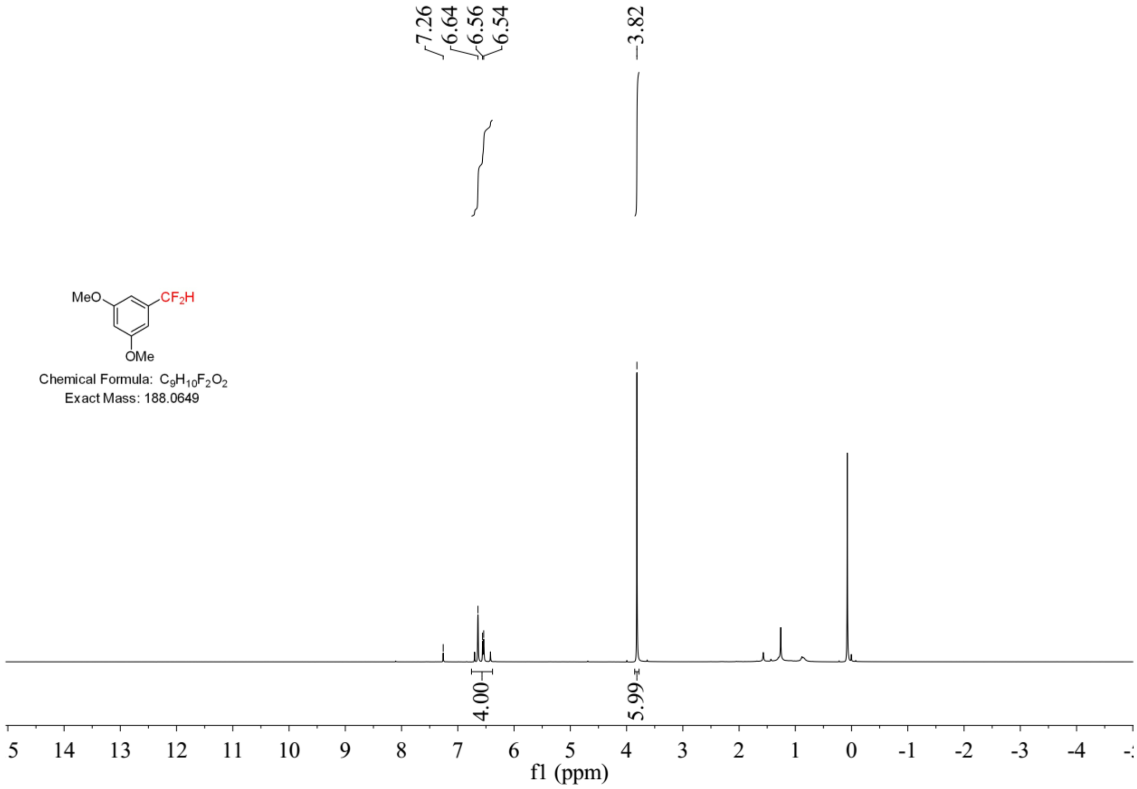

Supplementary Figure 23. ^{1}H-NMR of 1-(difluoromethyl)-3,5-dimethoxybenzene (3j)

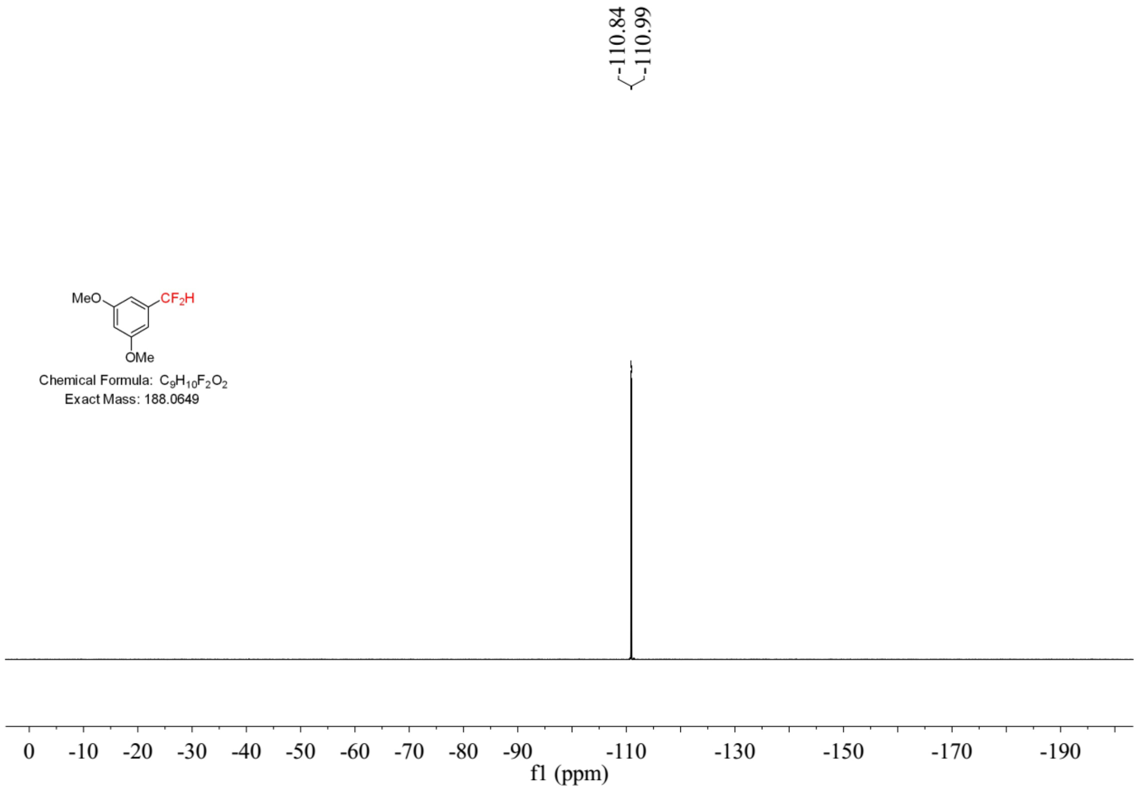

Supplementary Figure 24. ^{19}F-NMR of 1-(difluoromethyl)-3,5-dimethoxybenzene (3j)

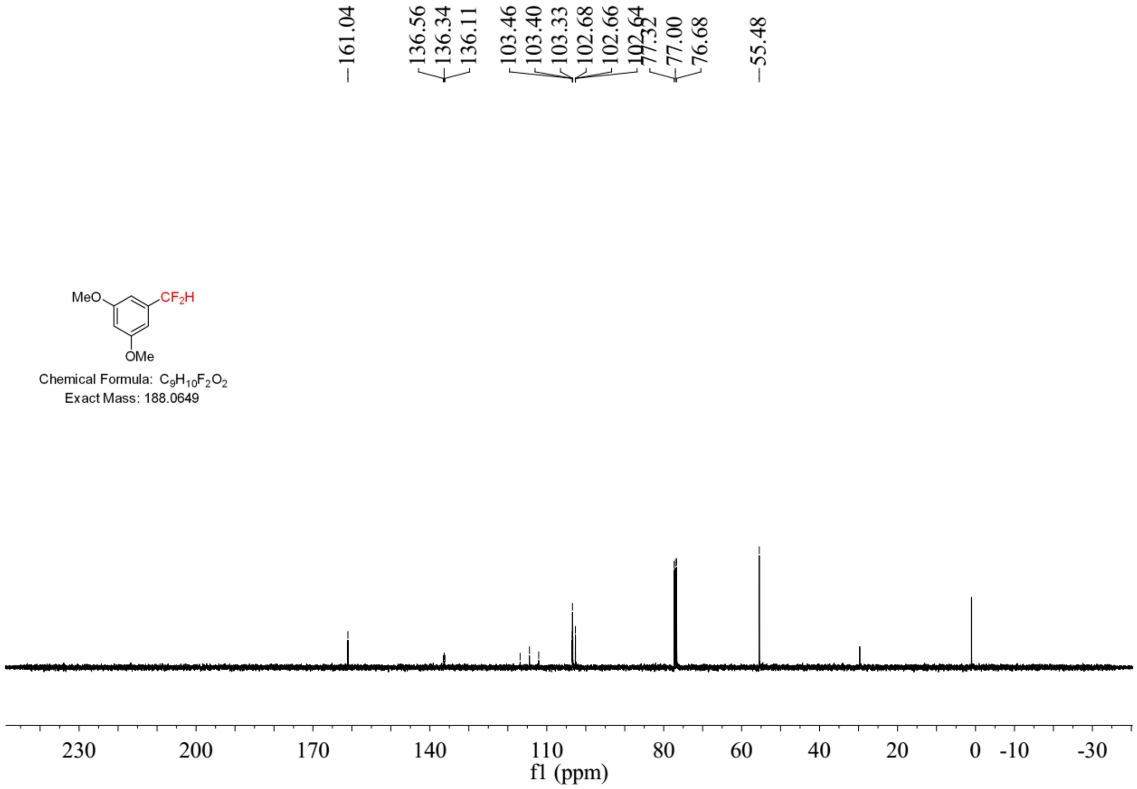

Supplementary Figure 25. **^{13}C-NMR of 1-(difluoromethyl)-3,5-dimethoxybenzene (3j)**

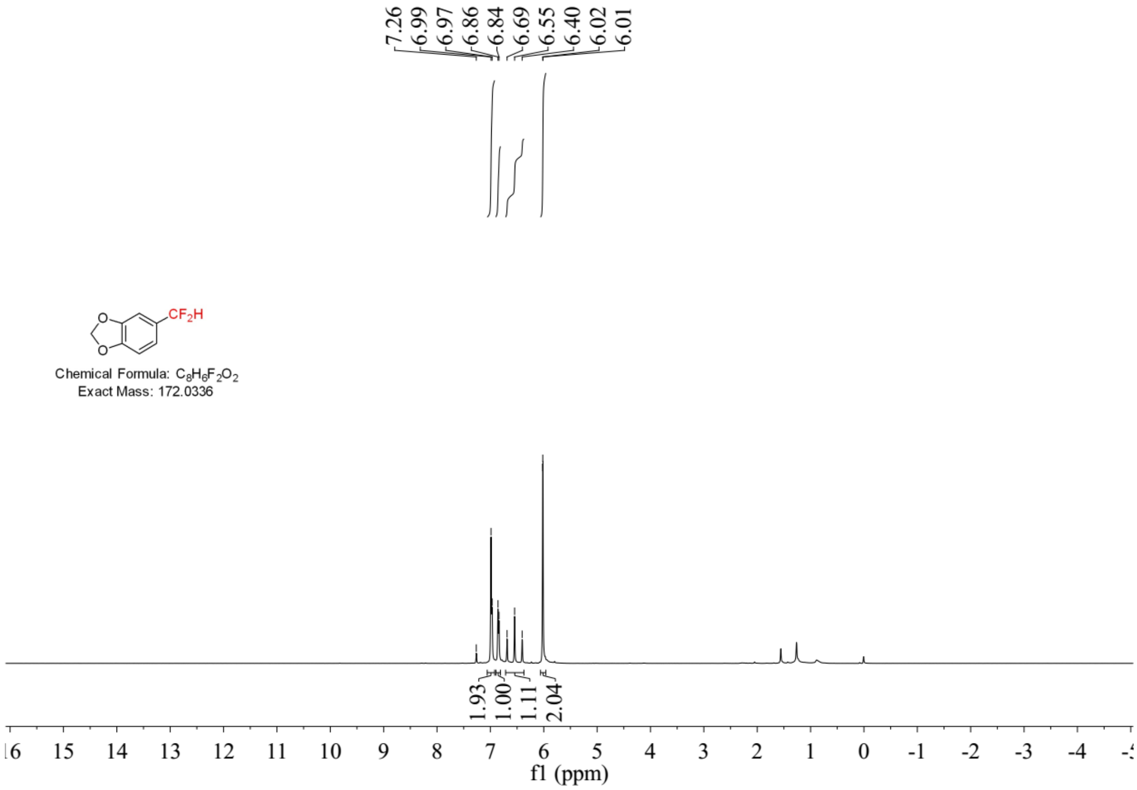

Supplementary Figure 26. **^{1}H-NMR of 5-(difluoromethyl)benzo[d][1,3]dioxole (3k)**

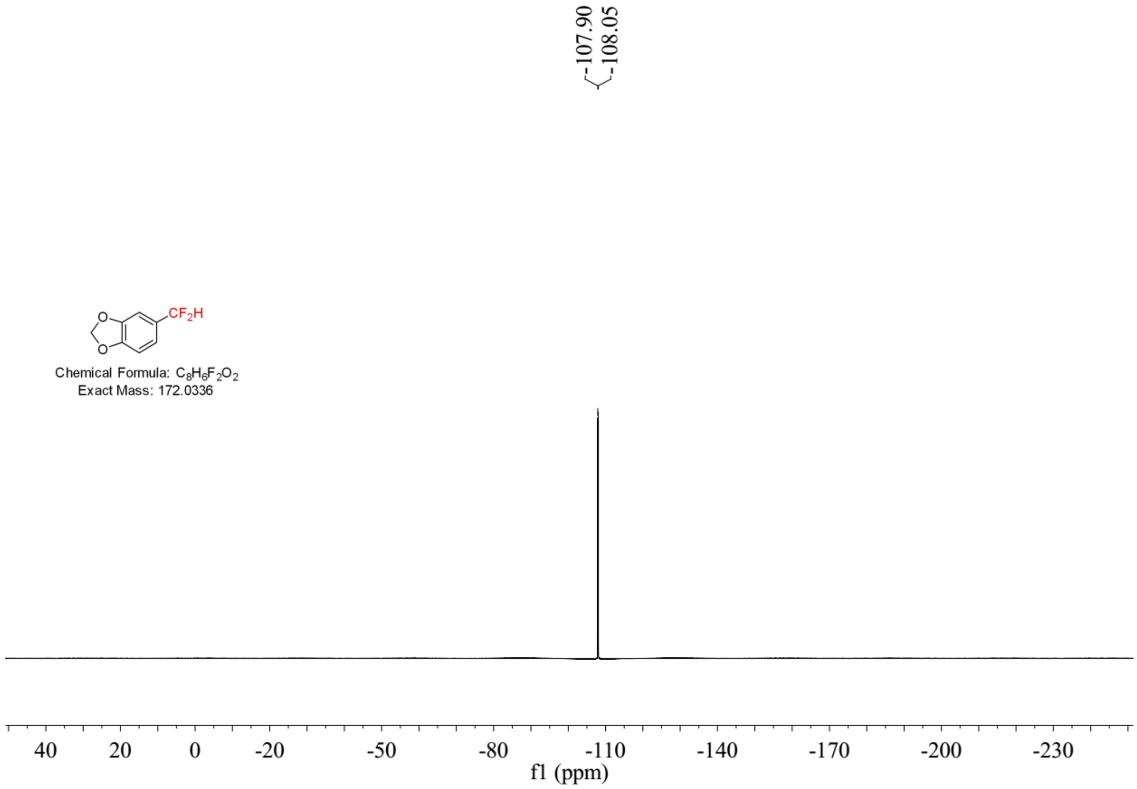

Supplementary Figure 27. **^{19}F-NMR of 5-(difluoromethyl)benzo[d][1,3]dioxole (3k)**

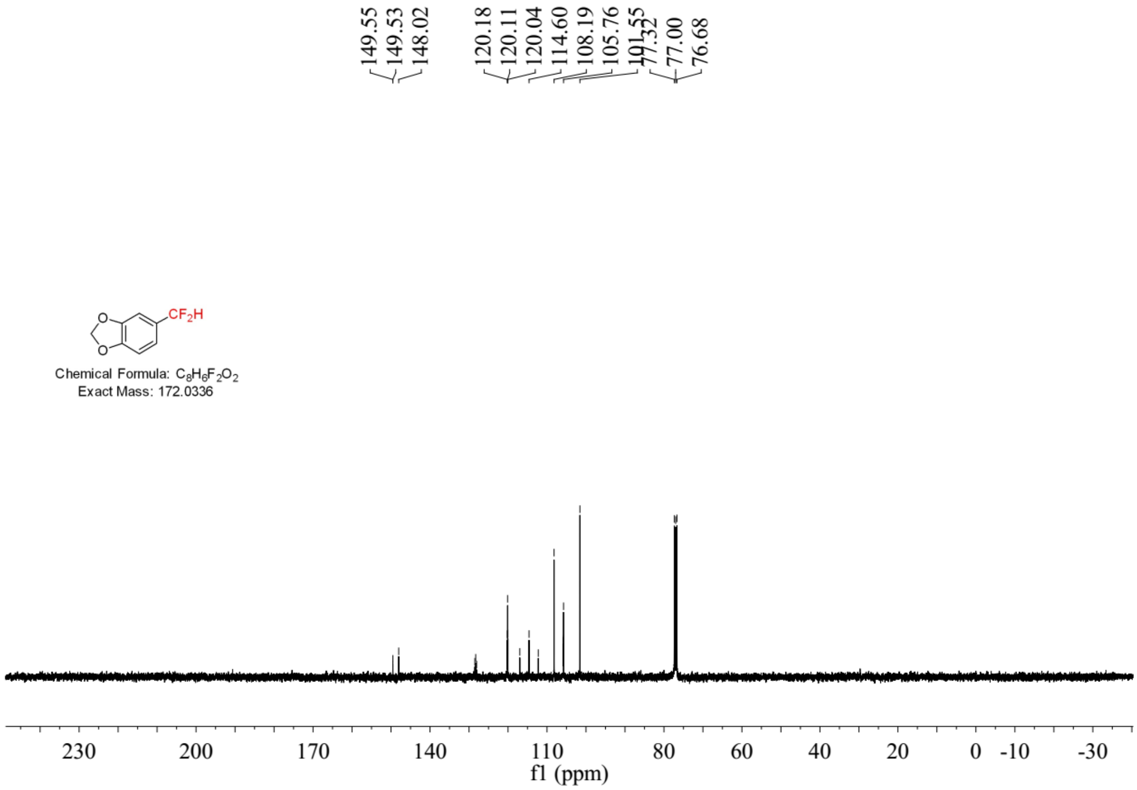

Supplementary Figure 28. **^{13}C-NMR of 5-(difluoromethyl)benzo[d][1,3]dioxole (3k)**

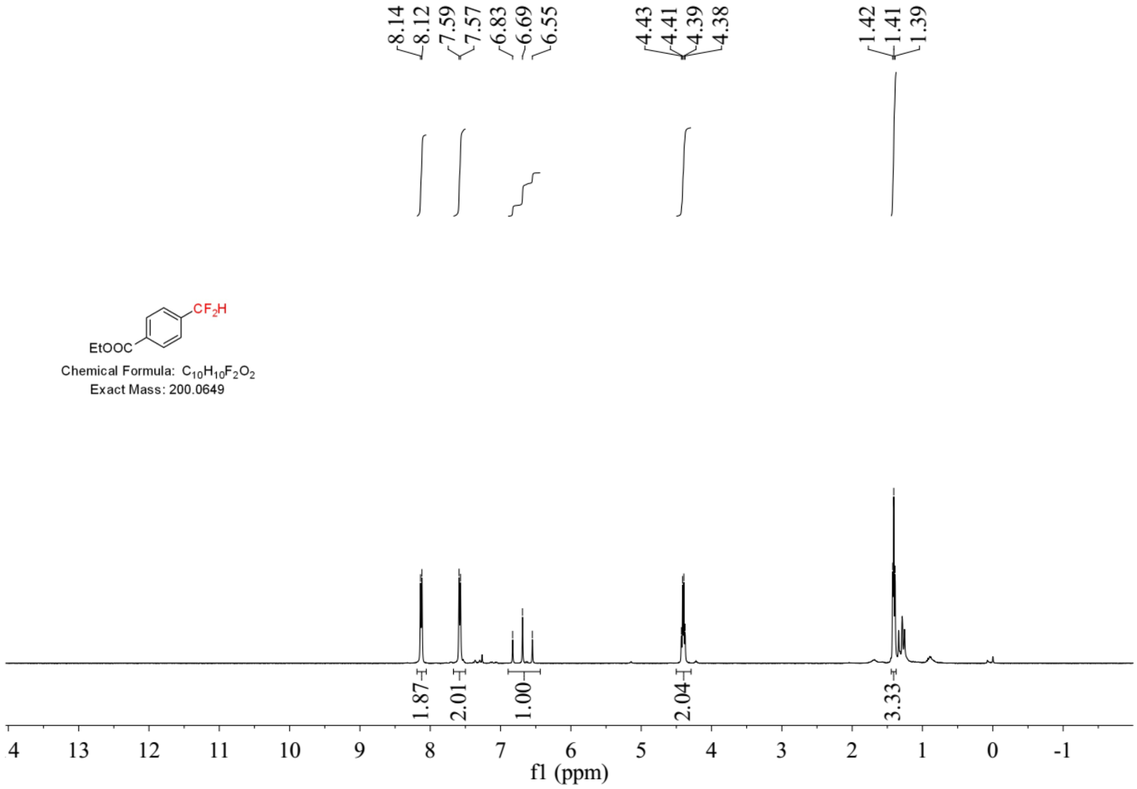

Supplementary Figure 29. ^{1}H-NMR of ethyl 4-(difluoromethyl)benzoate (3l)

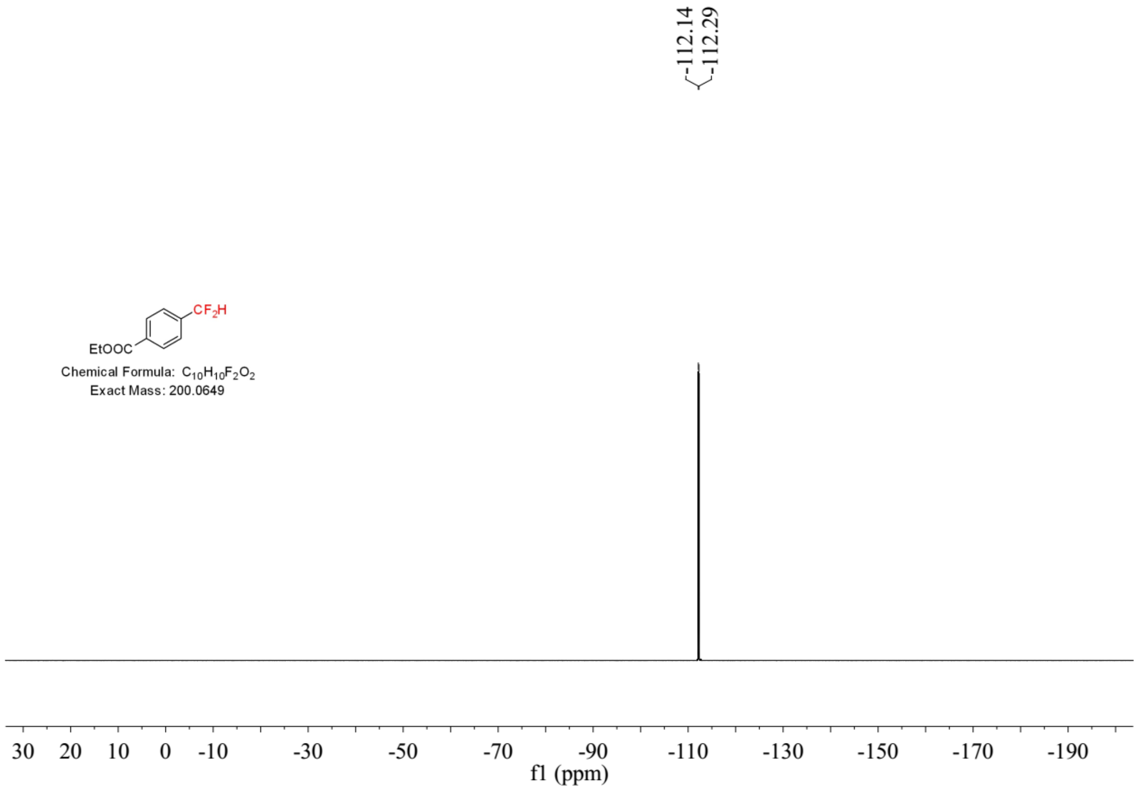

Supplementary Figure 30. ^{19}F-NMR of ethyl 4-(difluoromethyl)benzoate (3l)

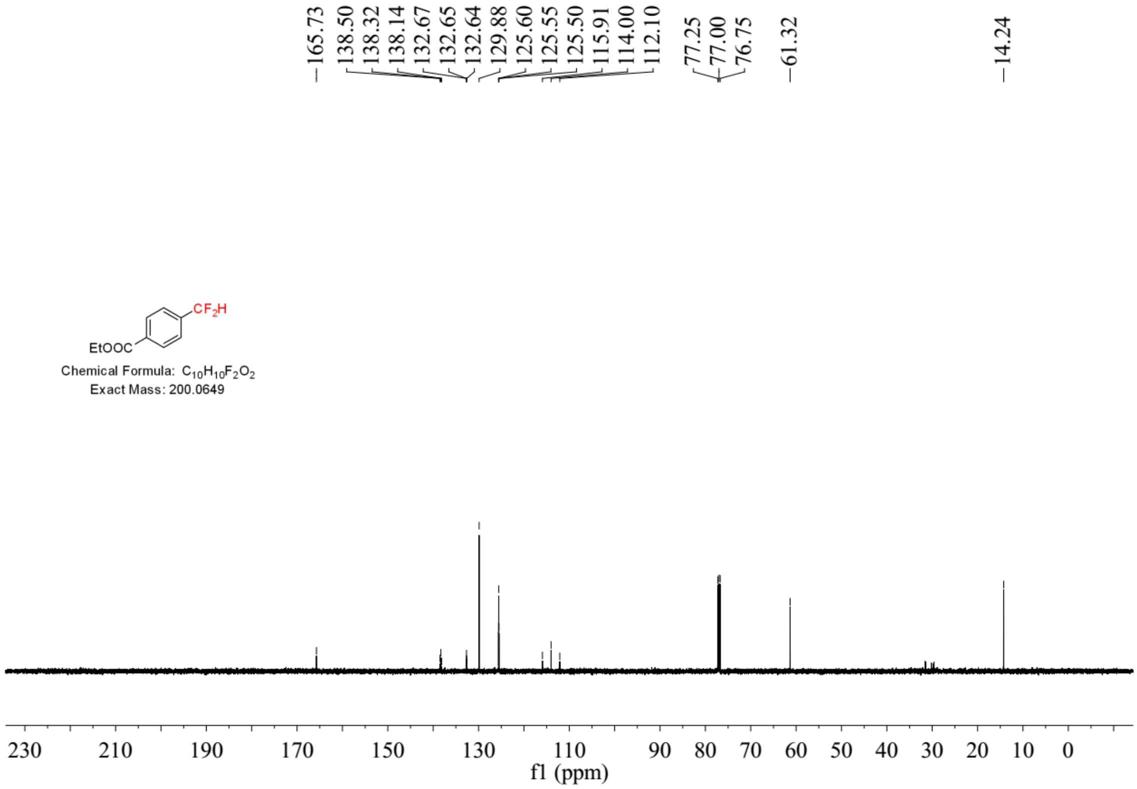

Supplementary Figure 31. ^{13}C-NMR of ethyl 4-(difluoromethyl)benzoate (3l)

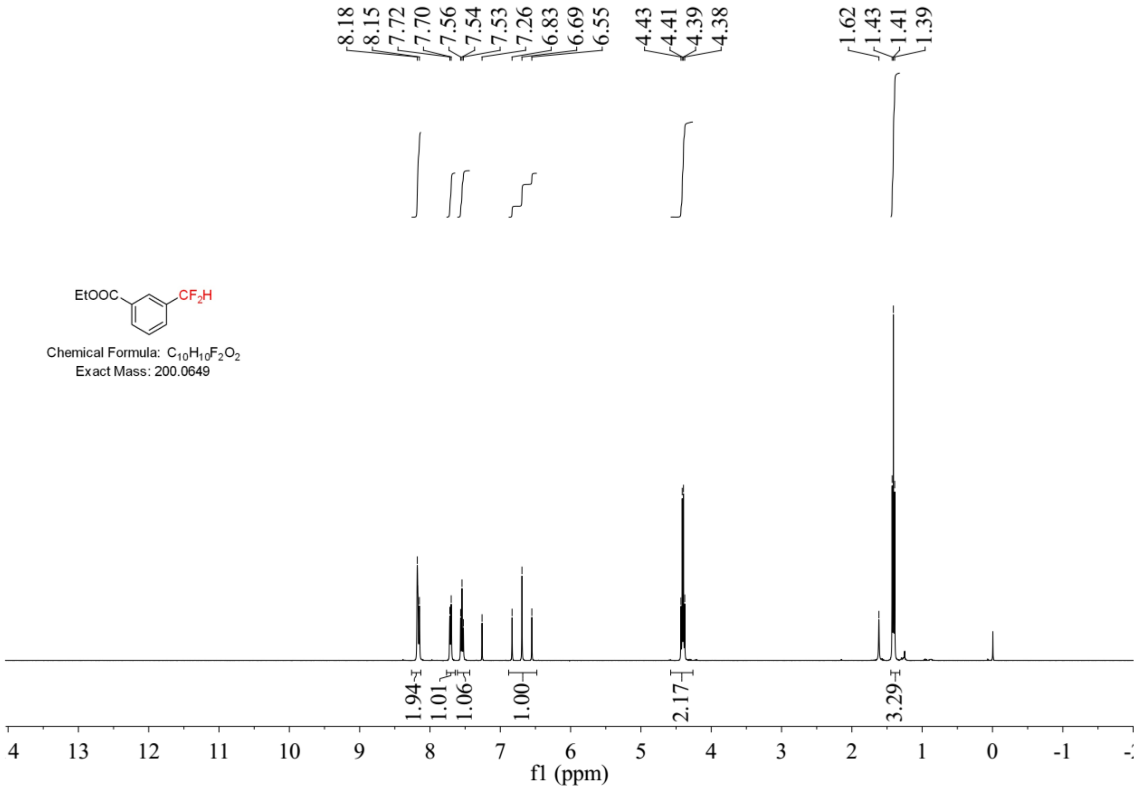

Supplementary Figure 32. ^{1}H-NMR of ethyl 3-(difluoromethyl)benzoate (3m)

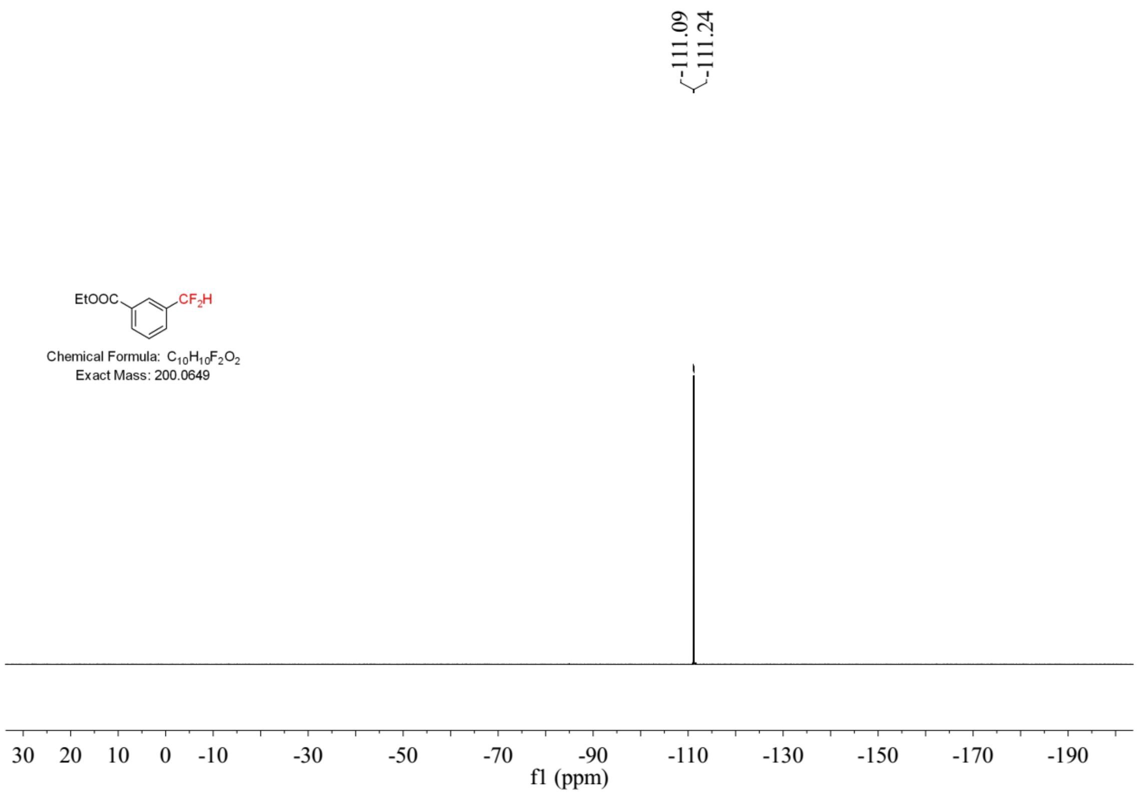

Supplementary Figure 33. ^{19}F-NMR of ethyl 3-(difluoromethyl)benzoate (3m)

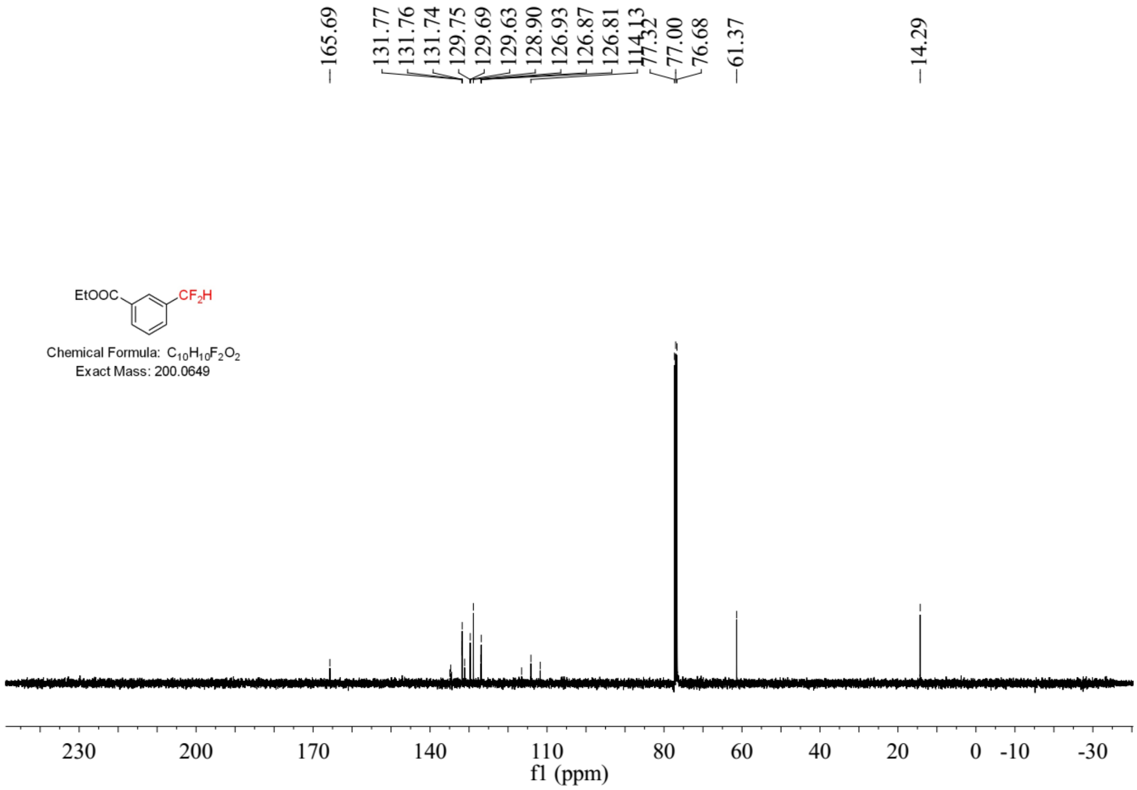

Supplementary Figure 34. ^{13}C-NMR of ethyl 3-(difluoromethyl)benzoate (3m)

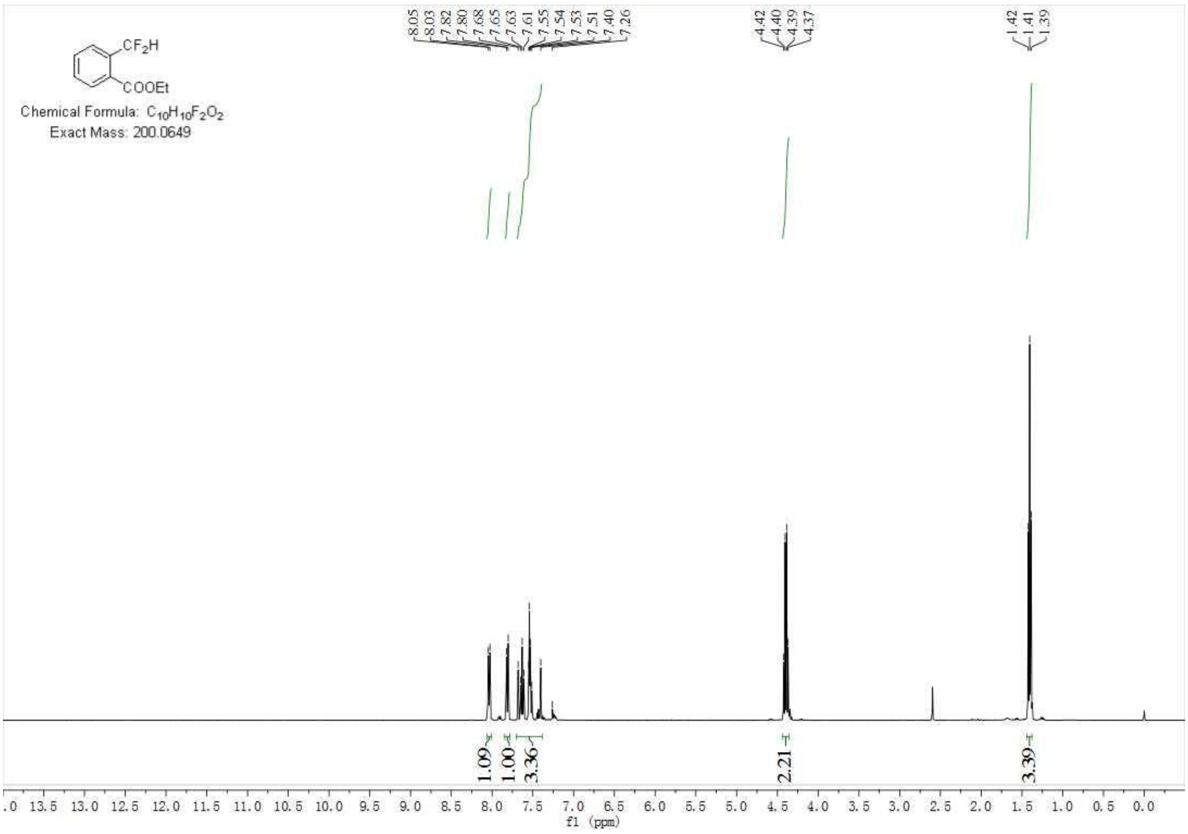

Supplementary Figure 35. ^{1}H-NMR of ethyl 2-(difluoromethyl)benzoate (3n)

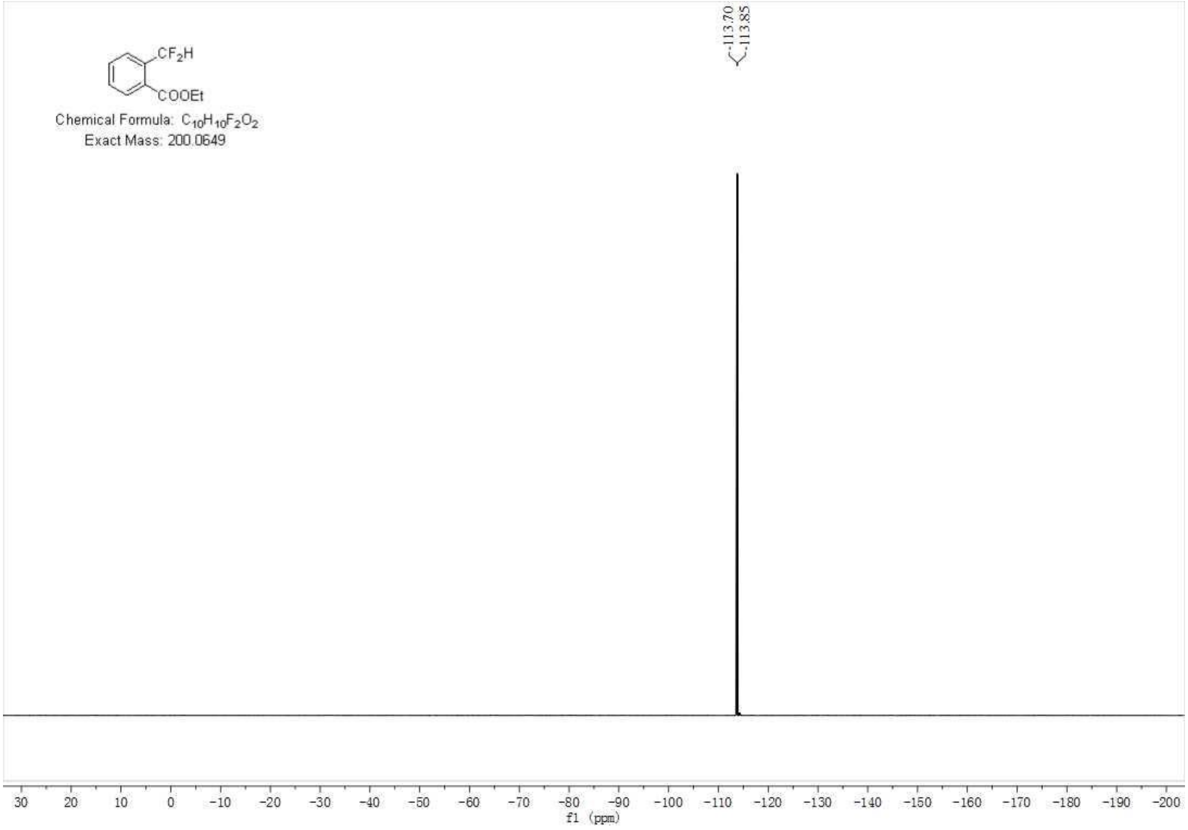

Supplementary Figure 36. ^{19}F-NMR of ethyl 2-(difluoromethyl)benzoate (3n)

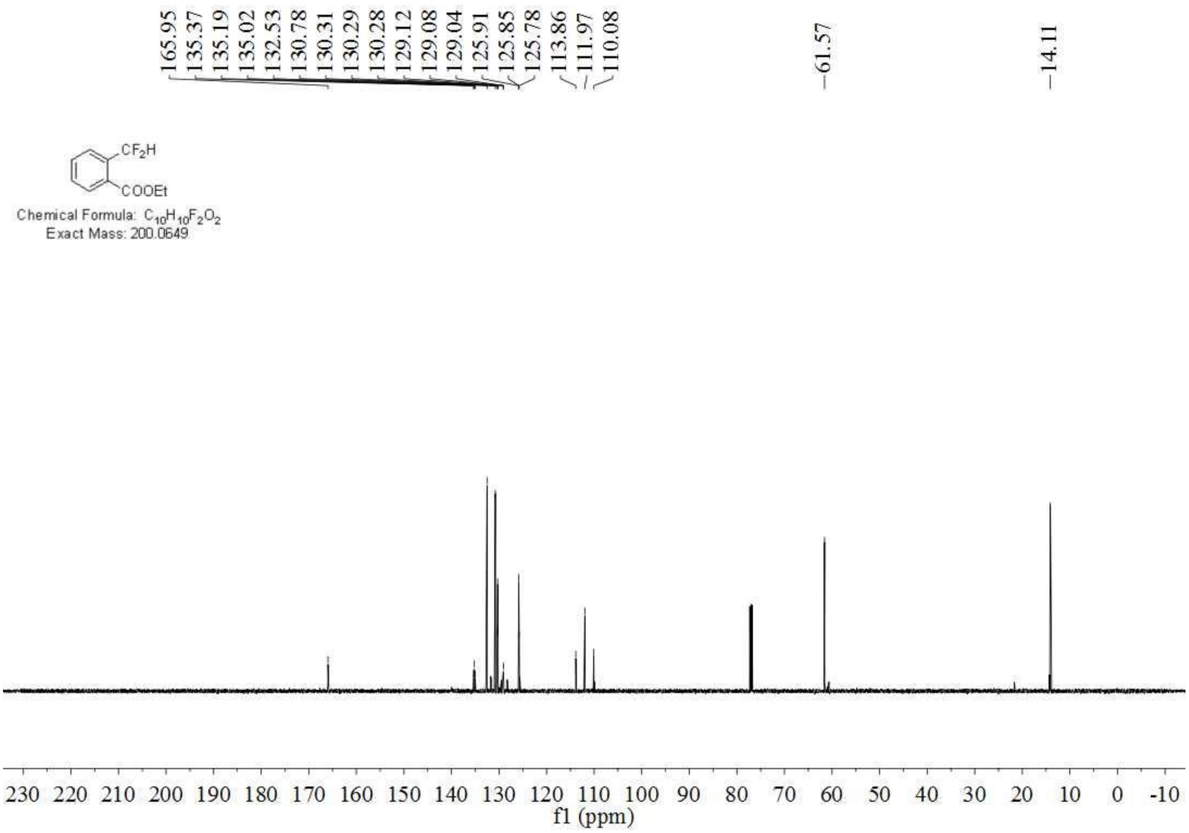

Supplementary Figure 37. ^{13}C-NMR of ethyl 2-(difluoromethyl)benzoate (3n)

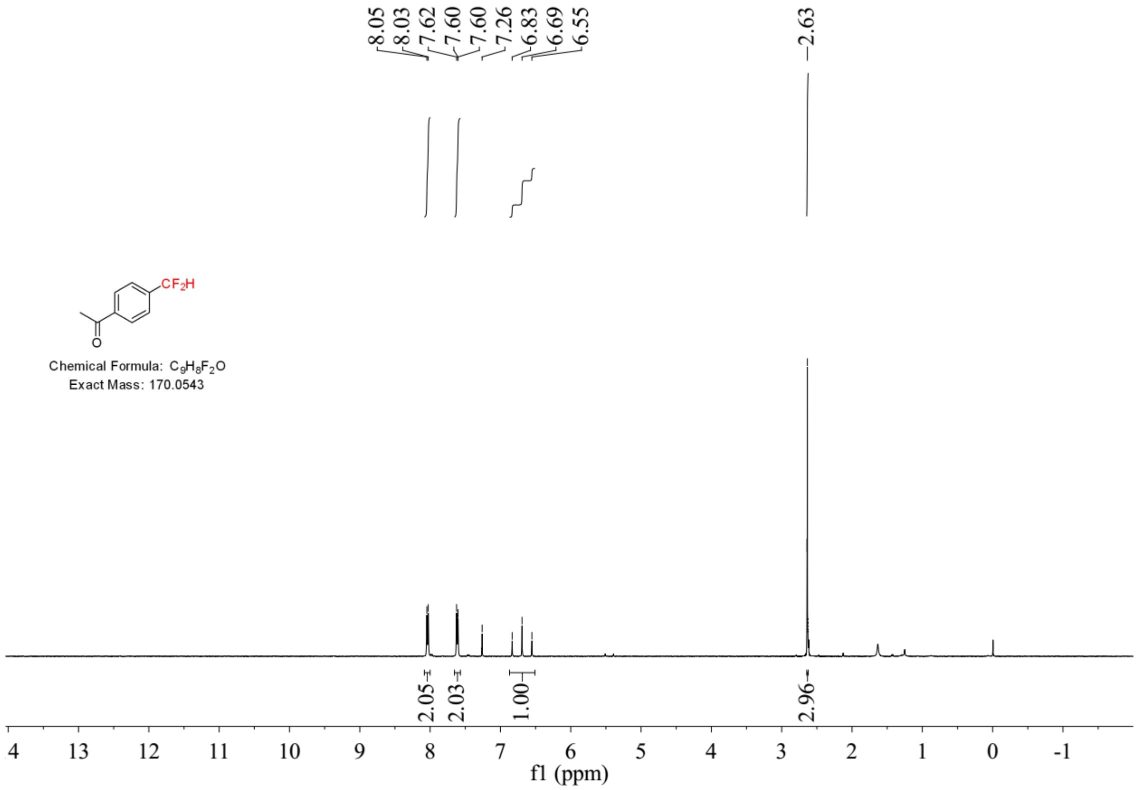

Supplementary Figure 38. ^{1}H-NMR of 1-(4-(difluoromethyl)phenyl)ethan-1-one (3o)

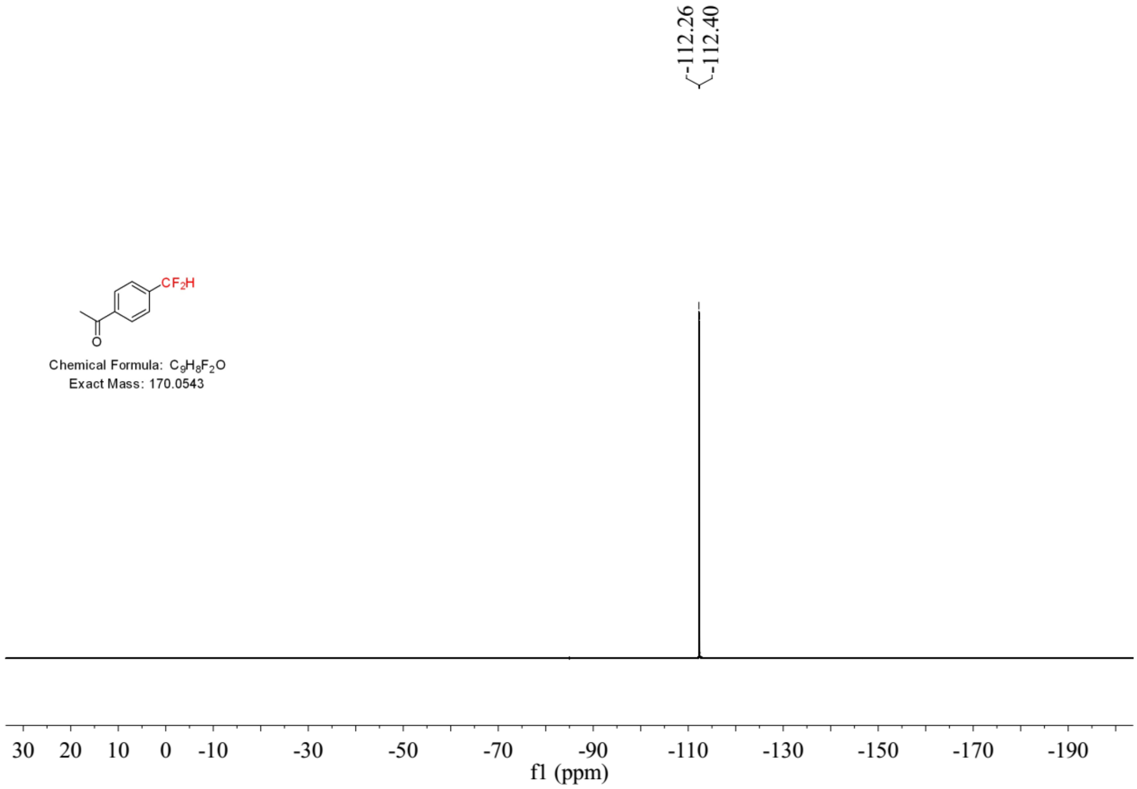

Supplementary Figure 39. ^{19}F-NMR of 1-(4-(difluoromethyl)phenyl)ethan-1-one (3o)

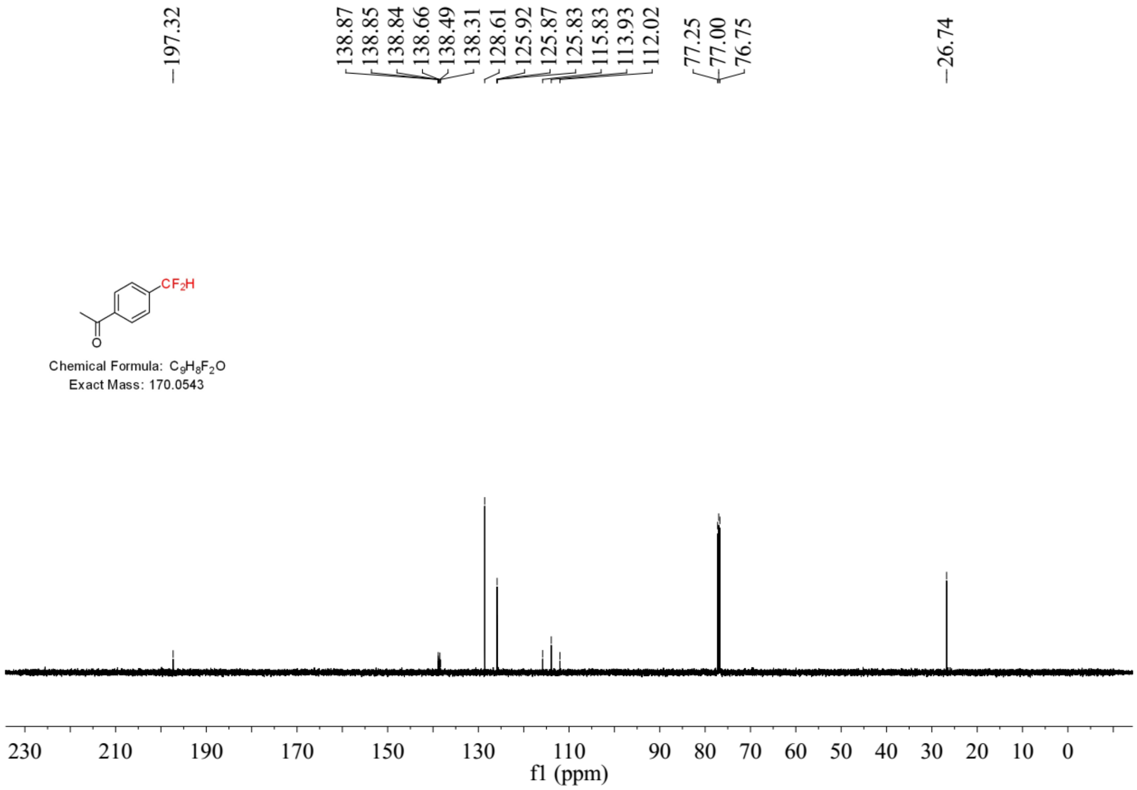

Supplementary Figure 40. ^{13}C-NMR of 1-(4-(difluoromethyl)phenyl)ethan-1-one (3o)

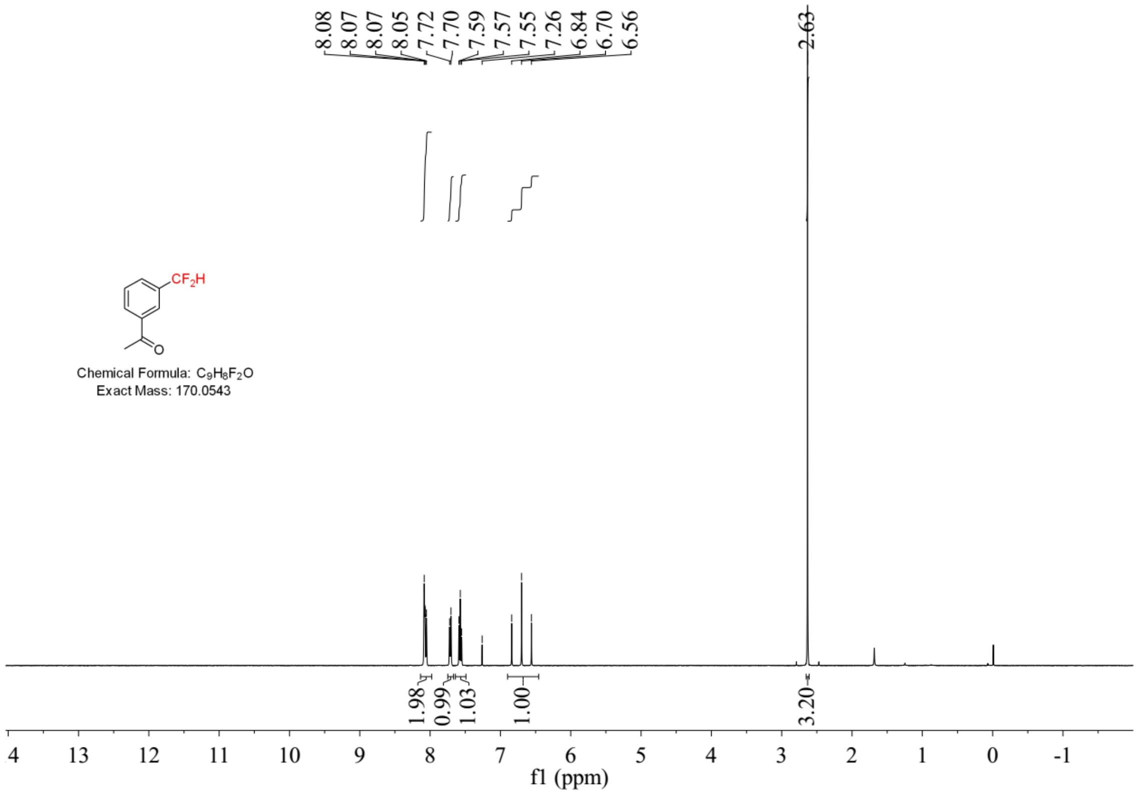

Supplementary Figure 41. ^{1}H-NMR of 1-(3-(difluoromethyl)phenyl)ethan-1-one (3p)

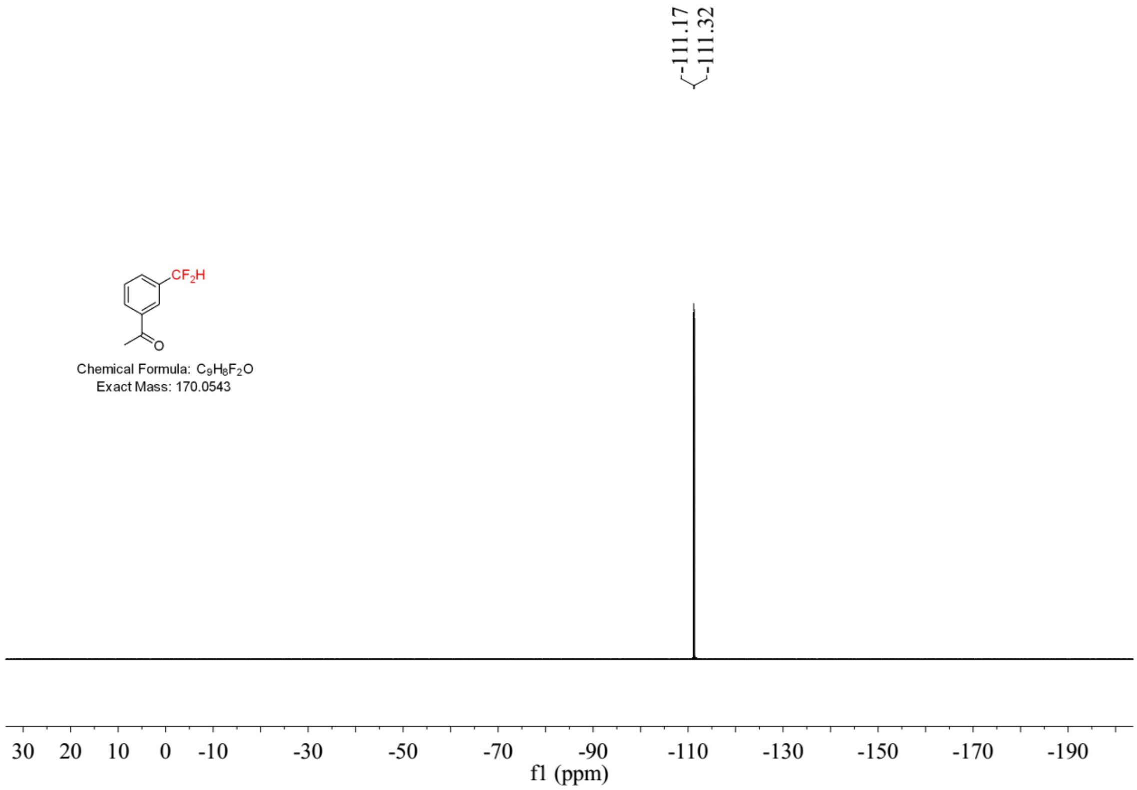

Supplementary Figure 42. ^{19}F-NMR of 1-(3-(difluoromethyl)phenyl)ethan-1-one (3p)

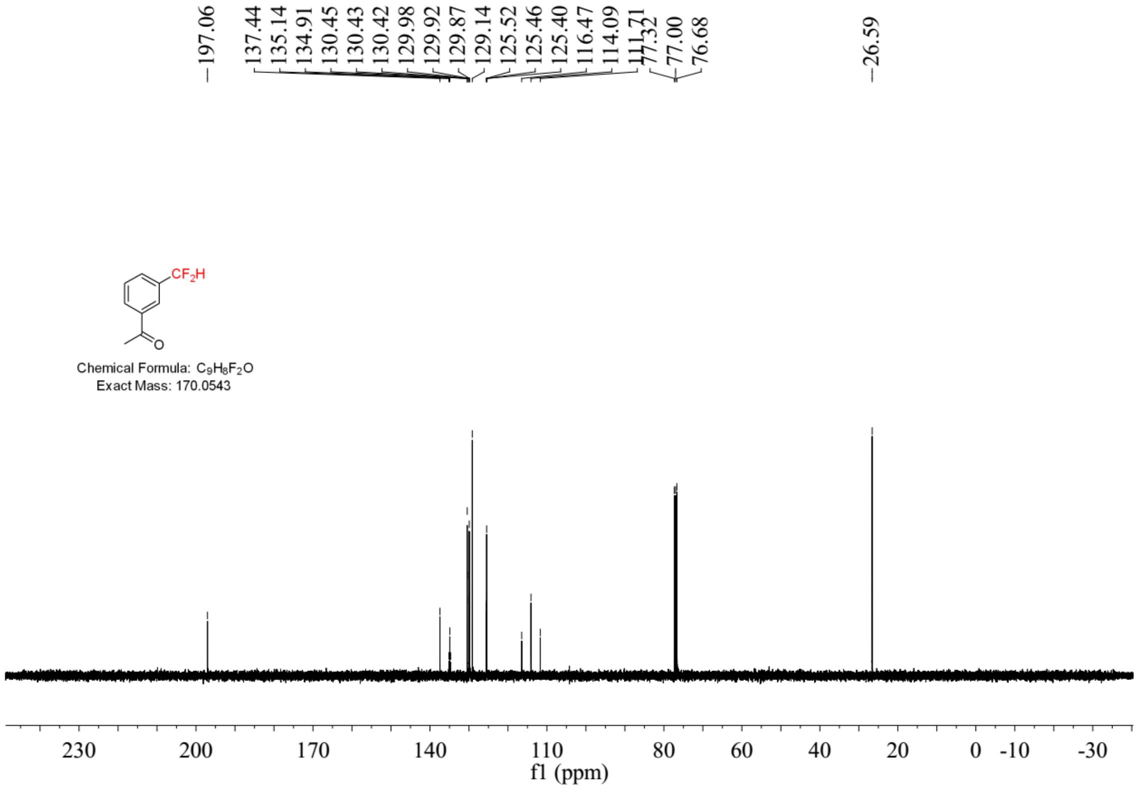

Supplementary Figure 43. ^{13}C-NMR of 1-(3-(difluoromethyl)phenyl)ethan-1-one (3p)

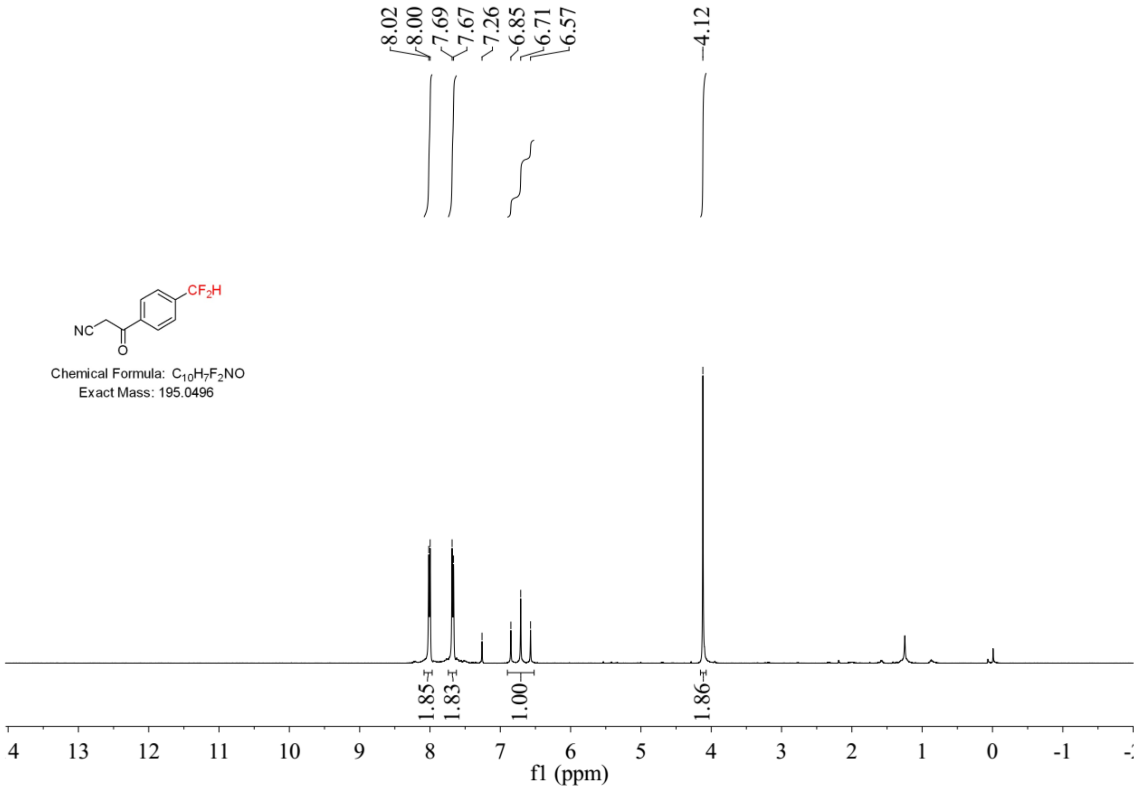

Supplementary Figure 44. ^{1}H-NMR of 3-(4-(difluoromethyl)phenyl)-3-oxopropanenitrile (3q)

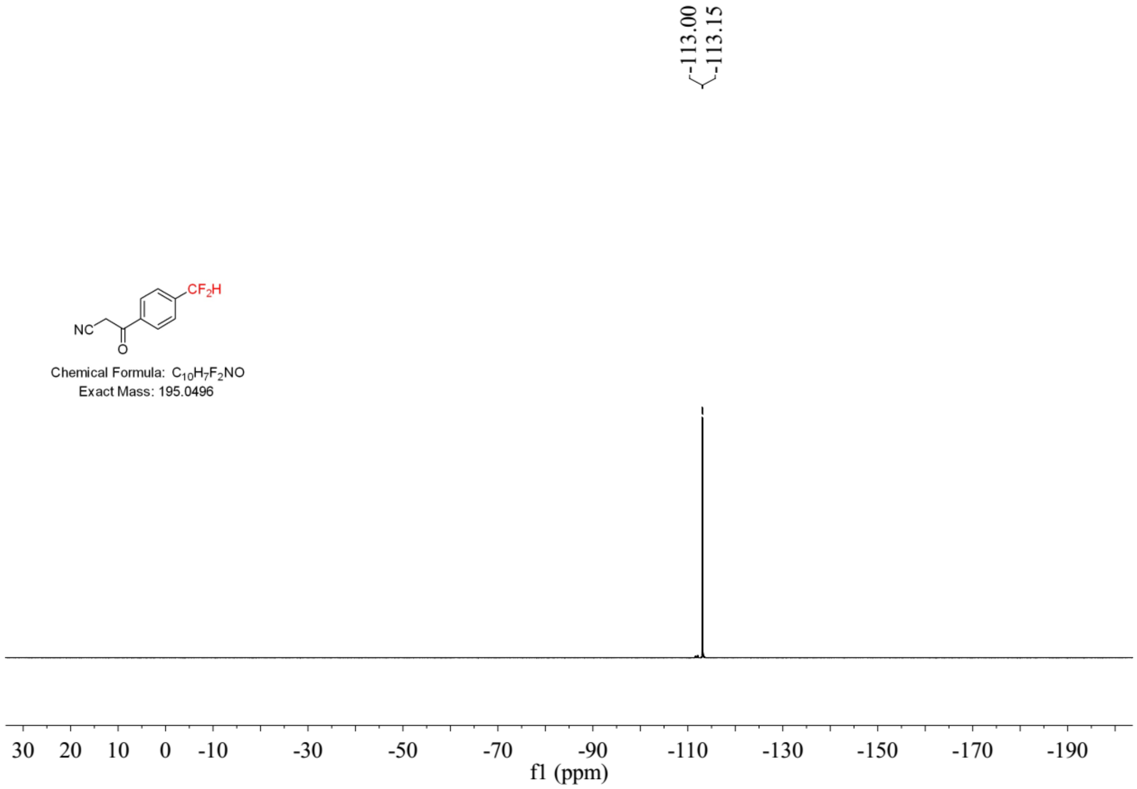

Supplementary Figure 45. ^{19}**F-NMR of 3-(4-(difluoromethyl)phenyl)-3-oxopropanenitrile (3q)**

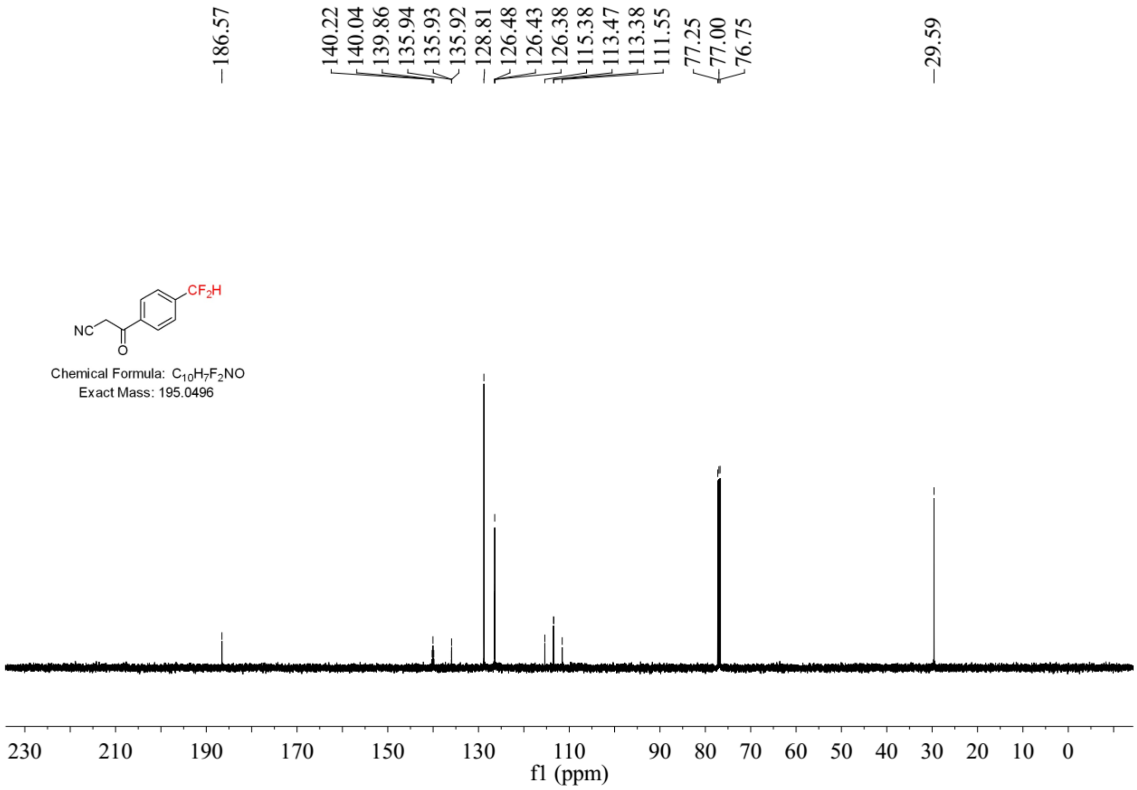

Supplementary Figure 46. ^{13}**C-NMR of 3-(4-(difluoromethyl)phenyl)-3-oxopropanenitrile (3q)**

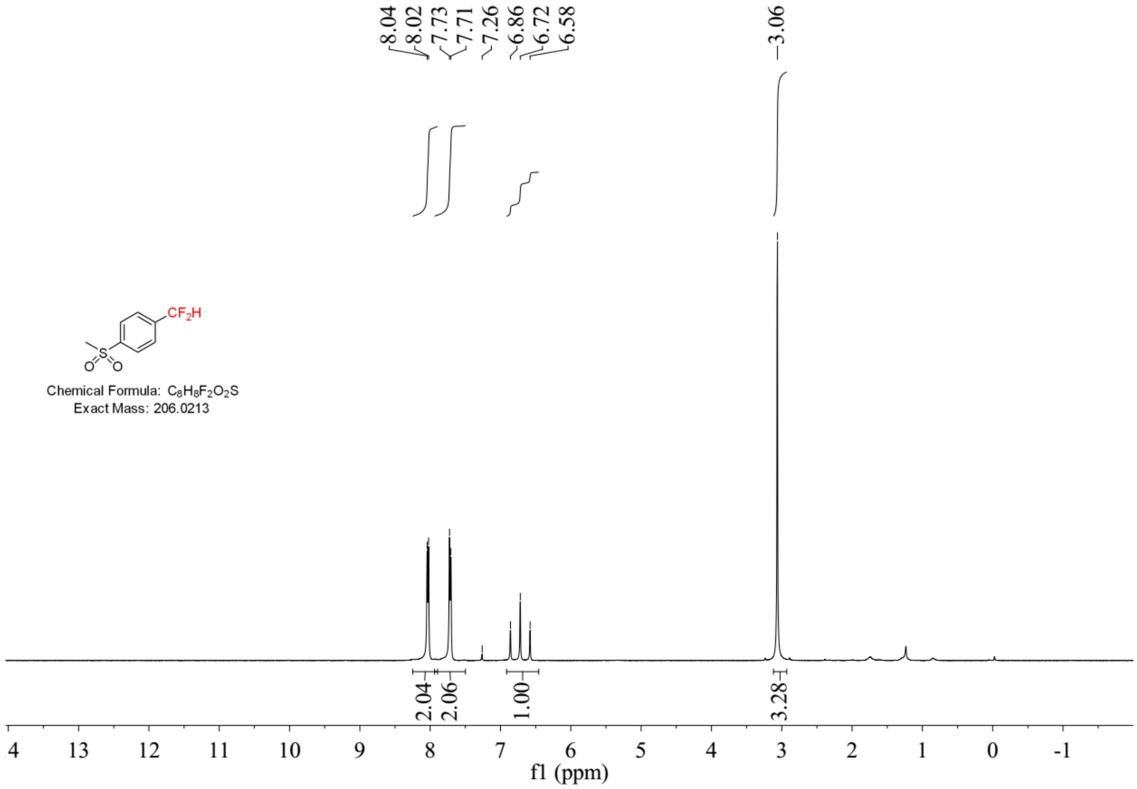

Supplementary Figure 47. ^{1}H-NMR of 1-(difluoromethyl)-4-(methylsulfonyl)benzene (3r)

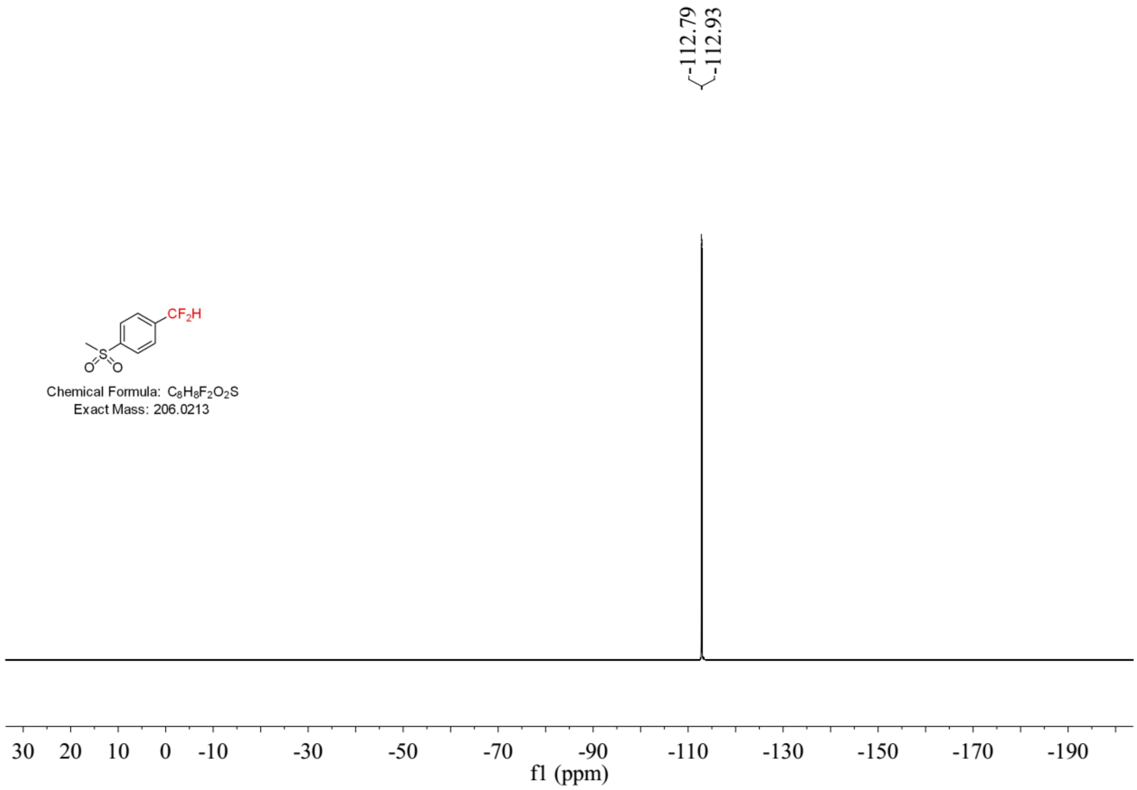

Supplementary Figure 48. ^{19}F-NMR of 1-(difluoromethyl)-4-(methylsulfonyl)benzene (3r)

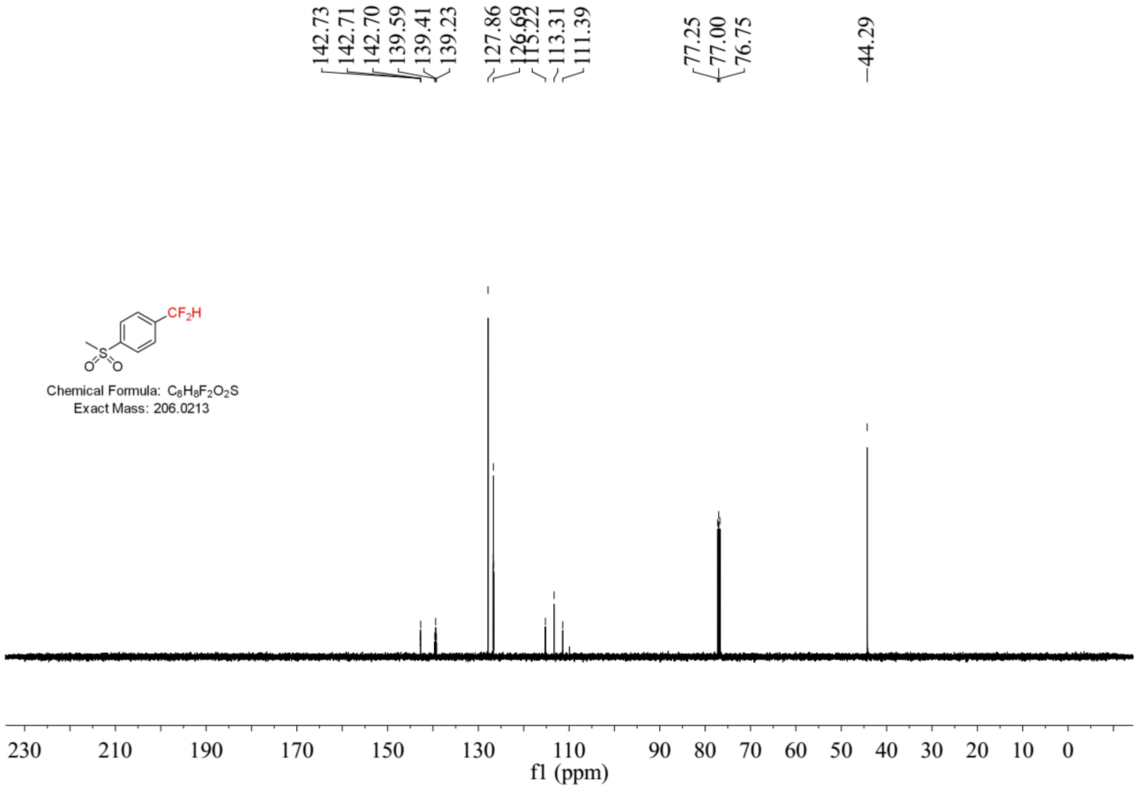

Supplementary Figure 49. ^{13}C-NMR of 1-(difluoromethyl)-4-(methylsulfonyl)benzene (3r)

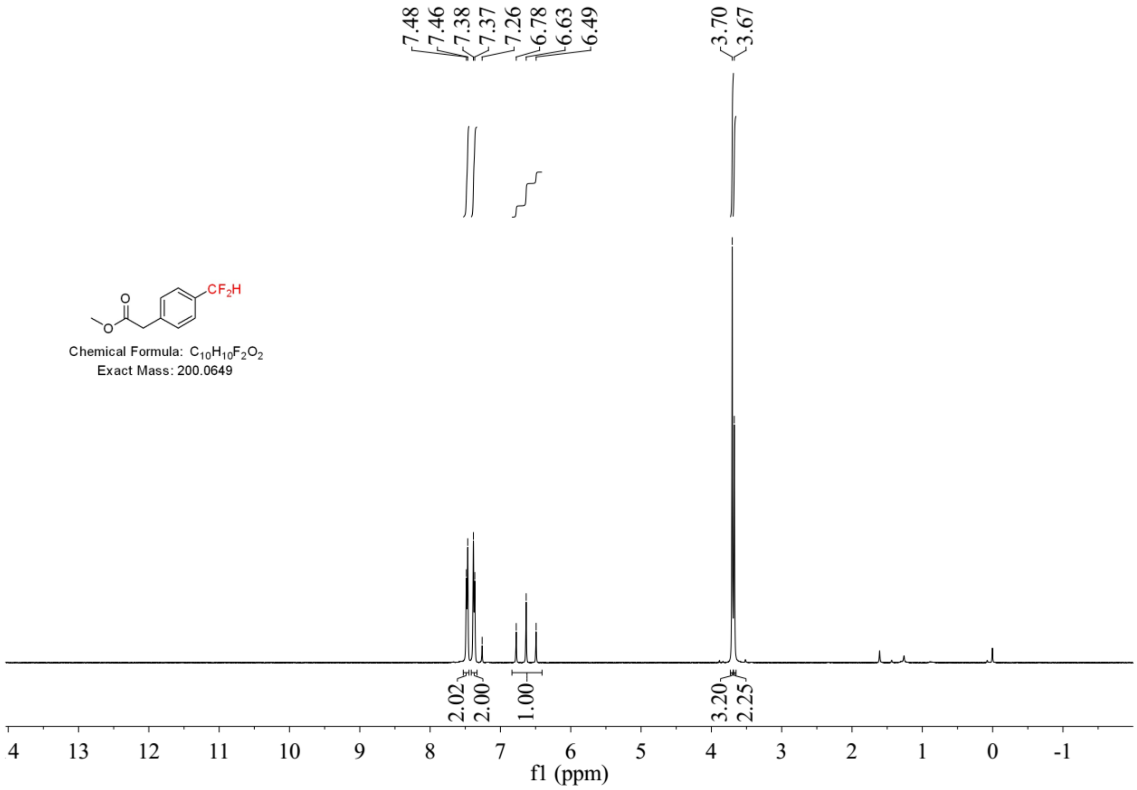

Supplementary Figure 50. ^{1}H-NMR of methyl 2-(4-(difluoromethyl)phenyl)acetate (3s)

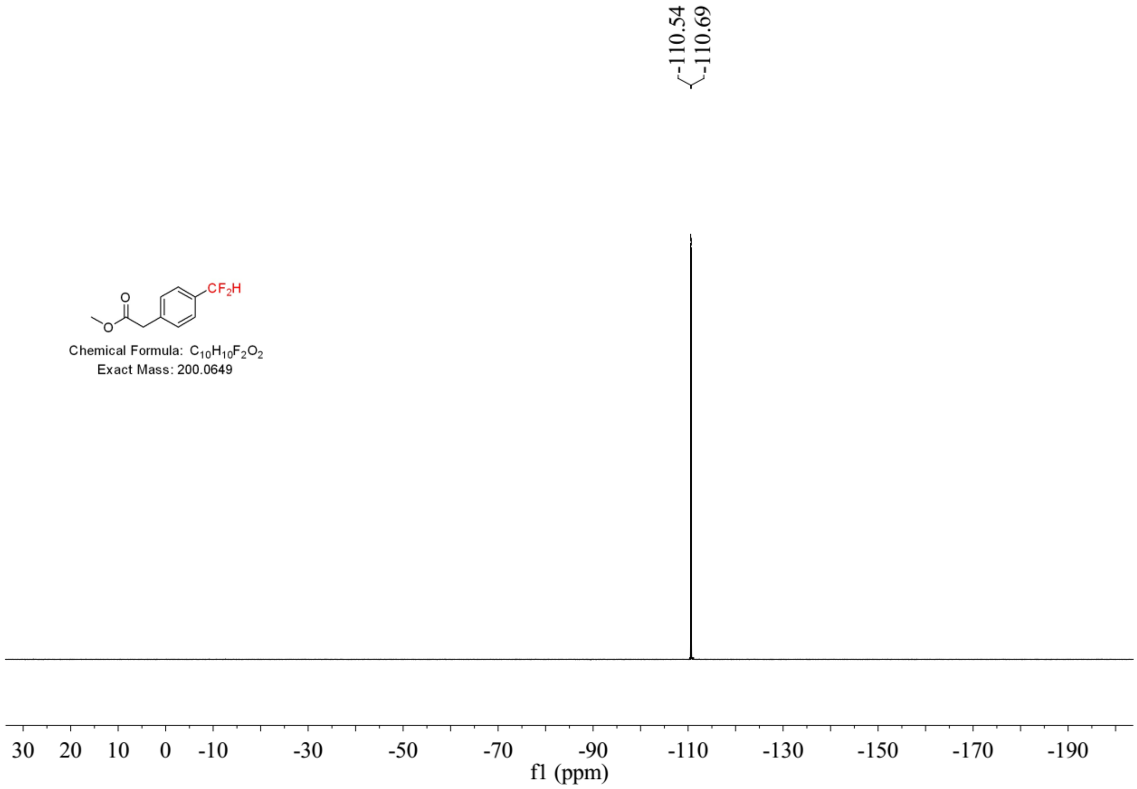

Supplementary Figure 51. ^{19}F-NMR of methyl 2-(4-(difluoromethyl)phenyl)acetate (3s)

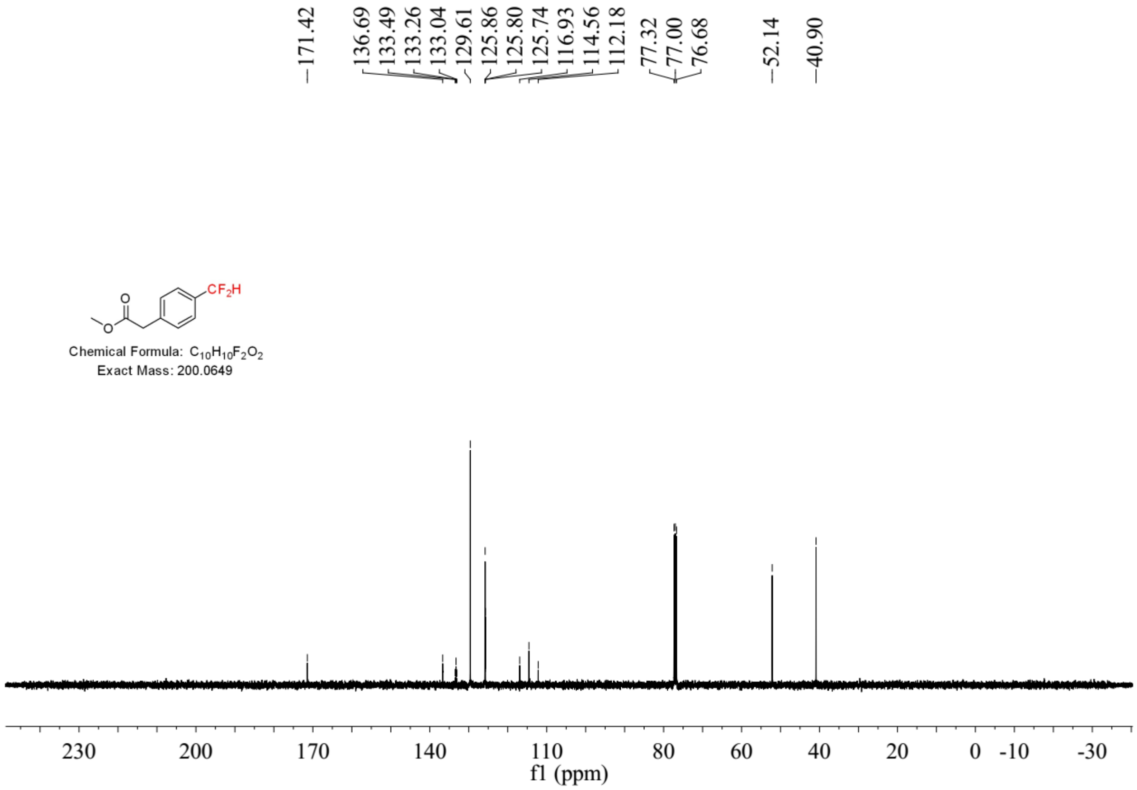

Supplementary Figure 52. ^{13}C-NMR of methyl 2-(4-(difluoromethyl)phenyl)acetate (3s)

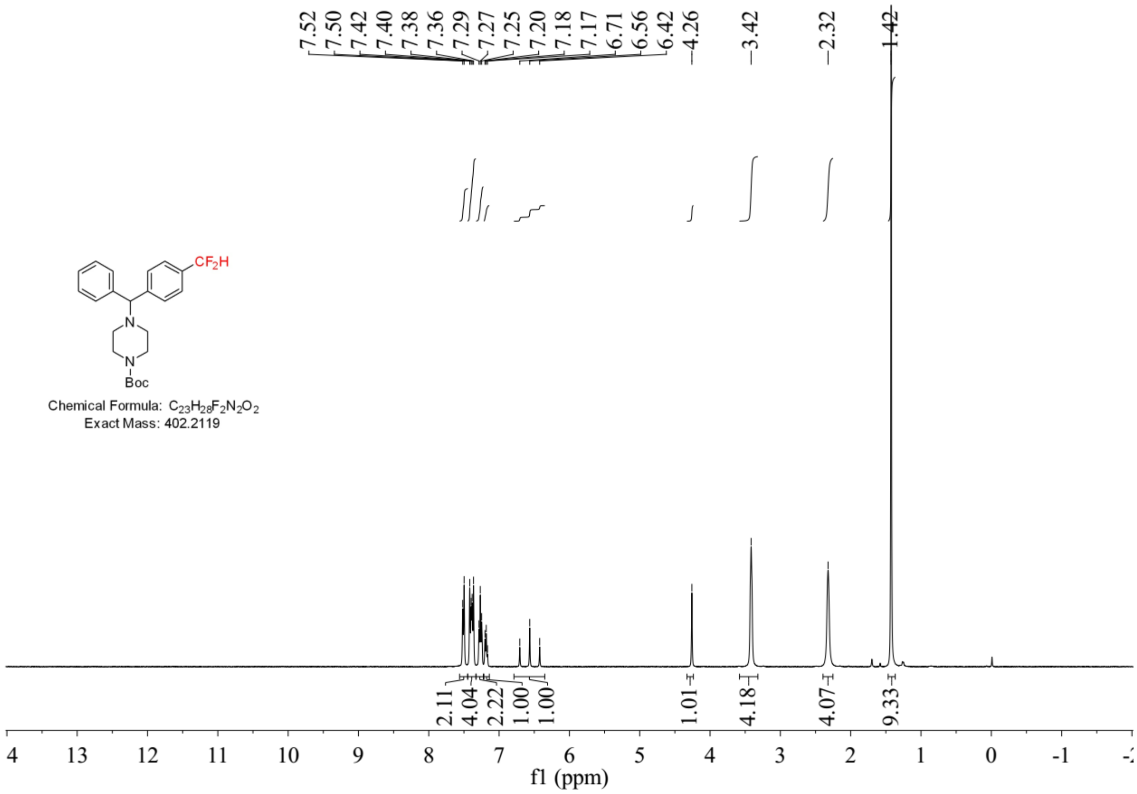

Supplementary Figure 53. ^{1}H-NMR of *tert*-butyl 4-((4-(difluoromethyl)phenyl)(phenyl)methyl)piperazine-1-carboxylate (3t)

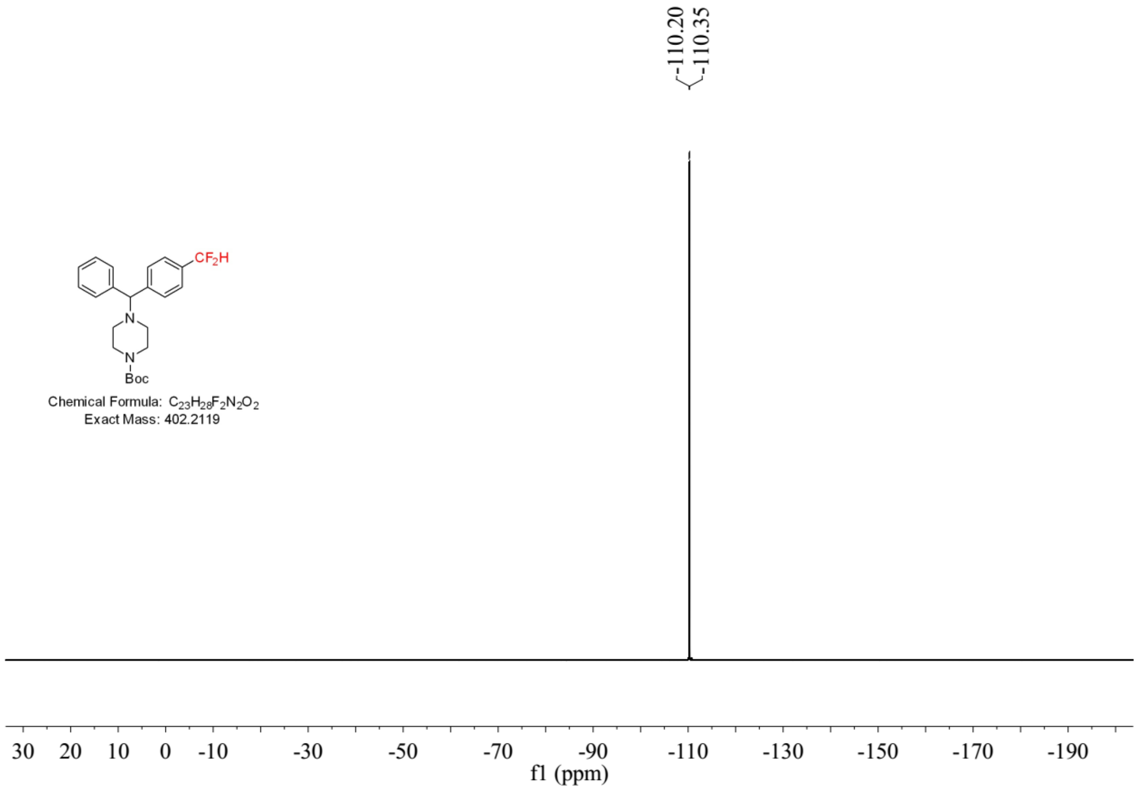

Supplementary Figure 54. ^{19}F-NMR of *tert*-butyl 4-((4-(difluoromethyl)phenyl)(phenyl)methyl)piperazine-1-carboxylate (3t)

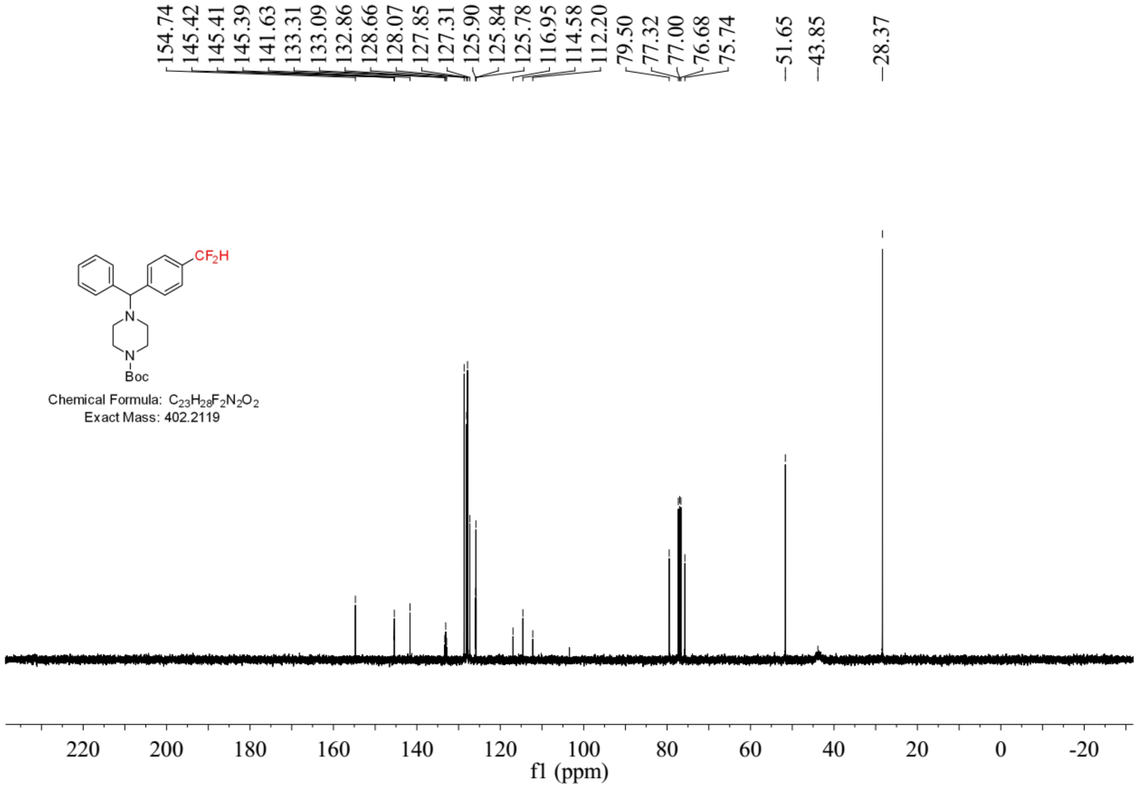

Supplementary Figure 55. ^{13}**C-NMR of** ***tert*****-nutyl 4-((4-(difluoromethyl)phenyl)(phenyl)methyl)piperazine-1-carboxylate (3t)**

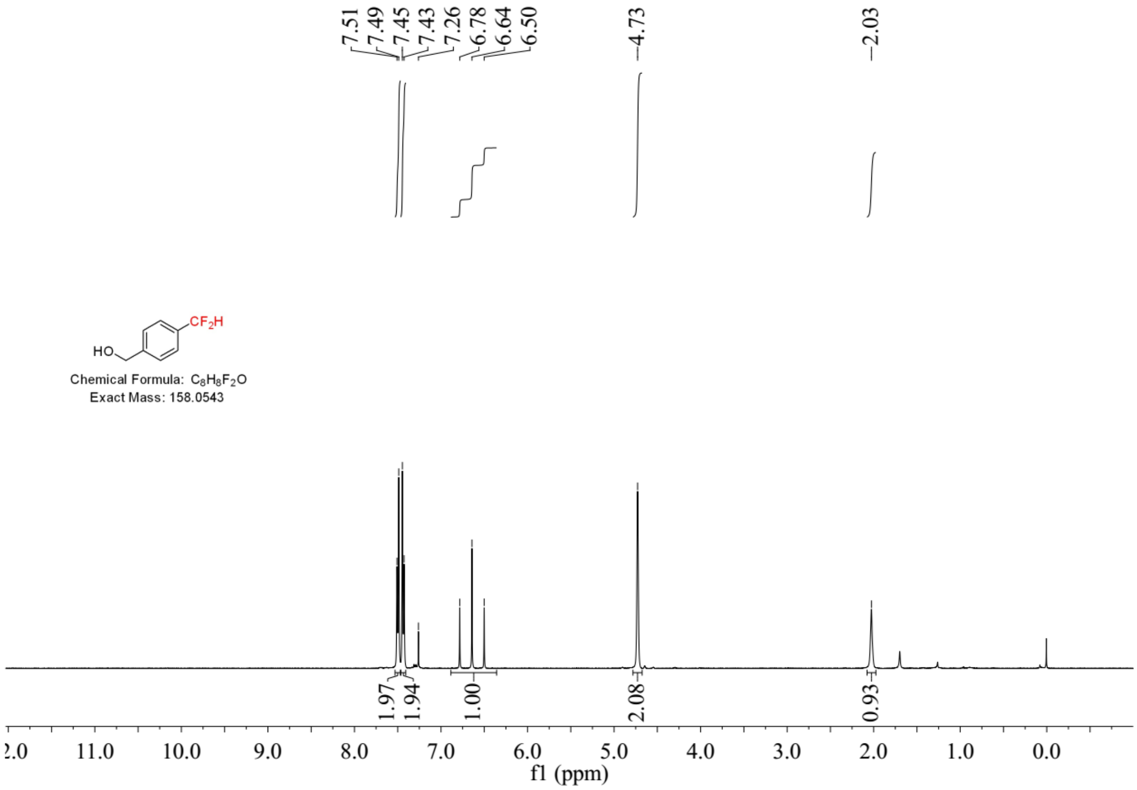

Supplementary Figure 56. ^{1}**H-NMR of (4-(difluoromethyl)phenyl)methanol (3u)**

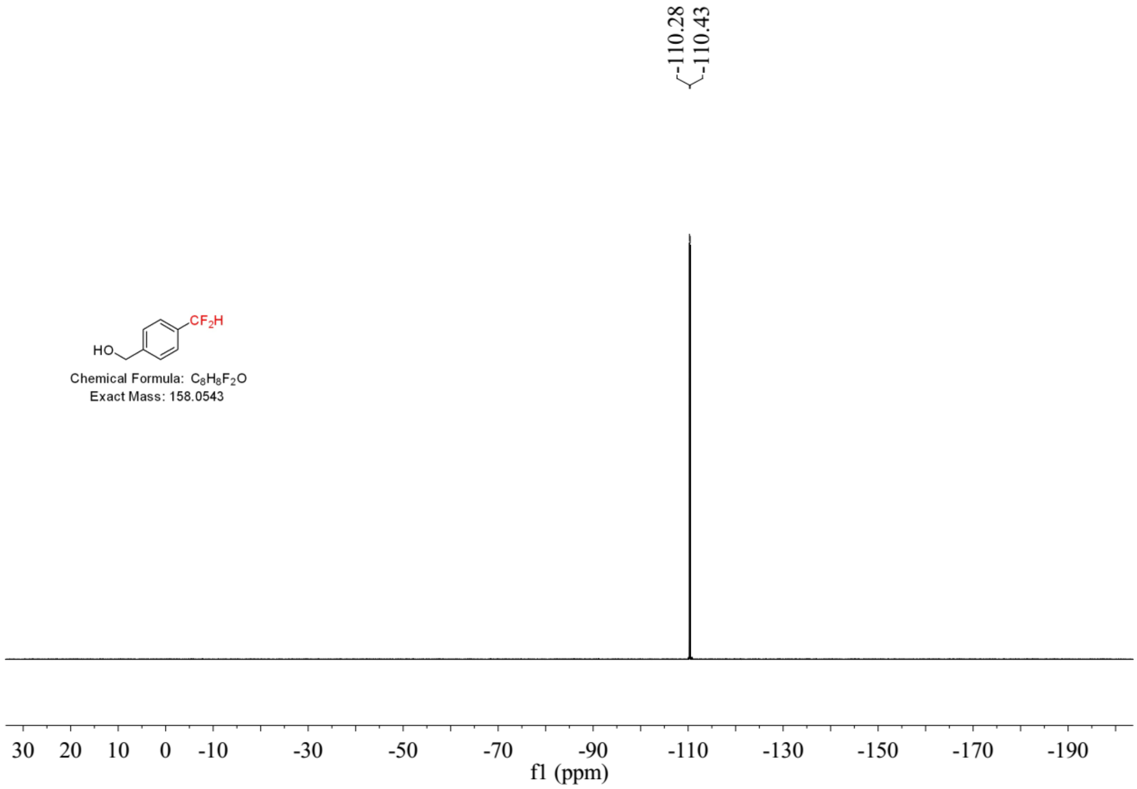

Supplementary Figure 57. ^{19}F-NMR of (4-(difluoromethyl)phenyl)methanol (3u)

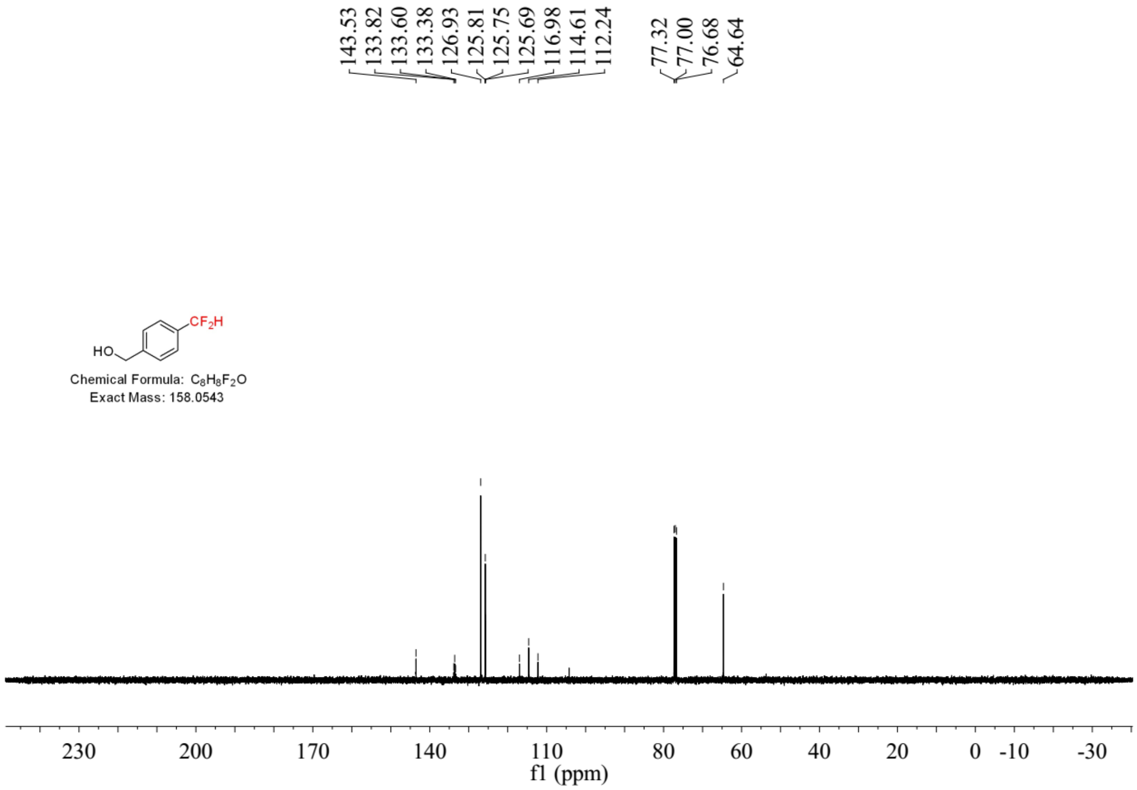

Supplementary Figure 58. ^{13}C-NMR of (4-(difluoromethyl)phenyl)methanol (3u)

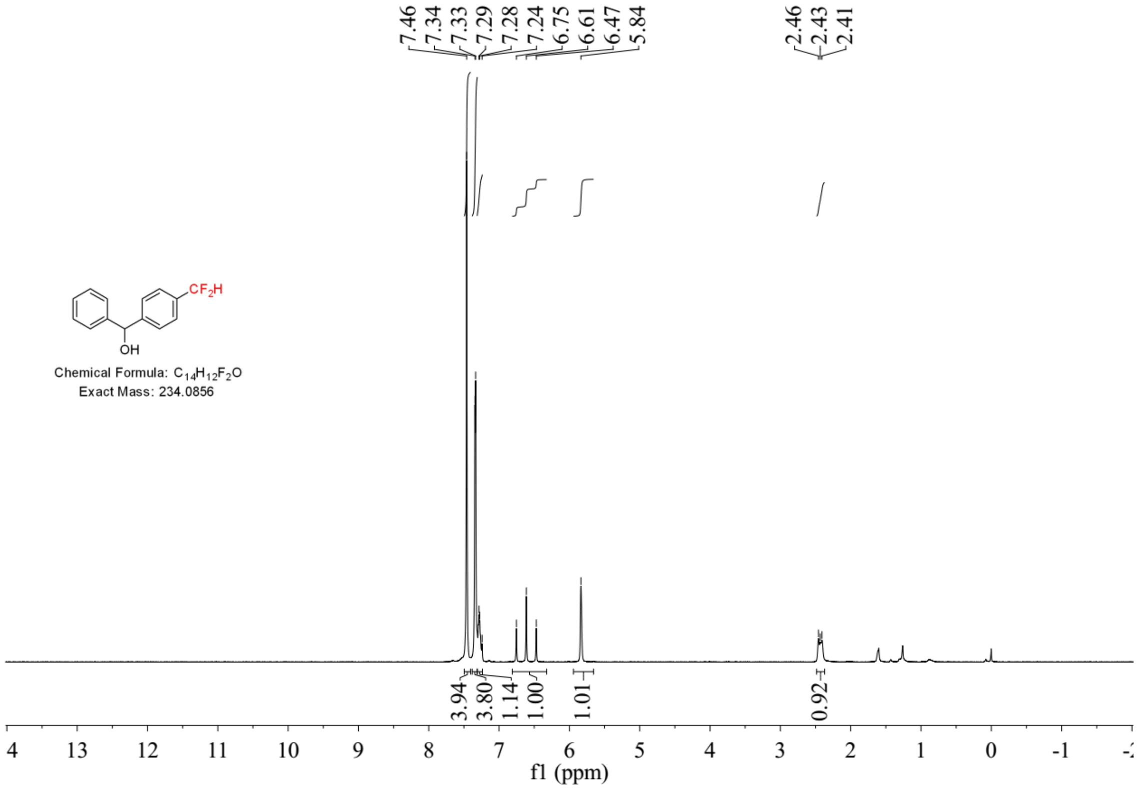

Supplementary Figure 59. ^{1}H-NMR of (4-(difluoromethyl)phenyl)(phenyl)methanol (3v)

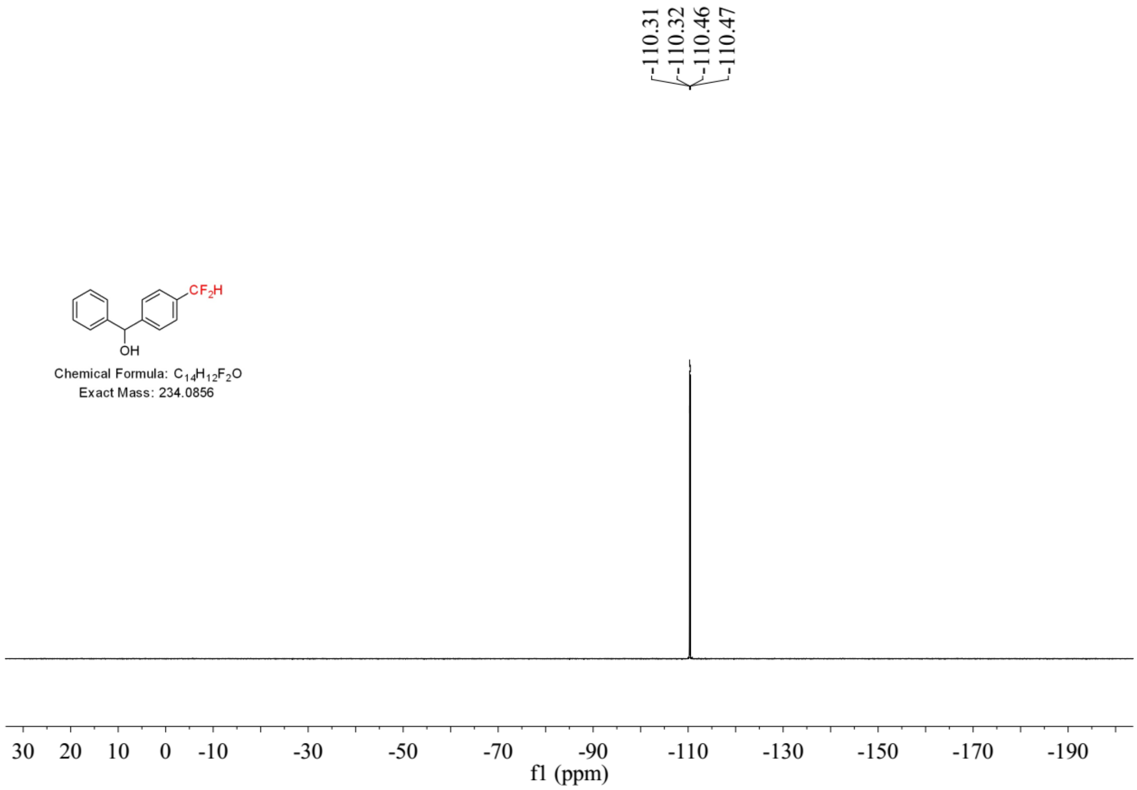

Supplementary Figure 60. ^{19}F-NMR of (4-(difluoromethyl)phenyl)(phenyl)methanol (3v)

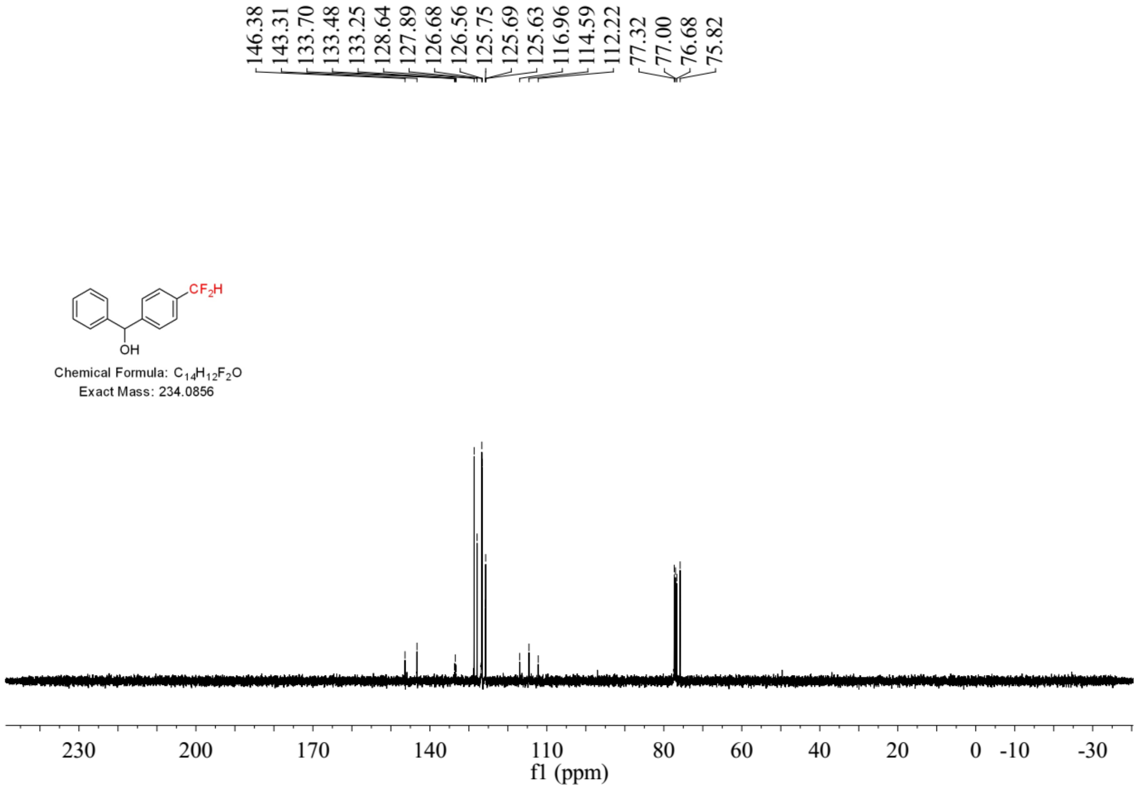

Supplementary Figure 61. **^{13}C-NMR of (4-(difluoromethyl)phenyl)(phenyl)methanol (3v)**

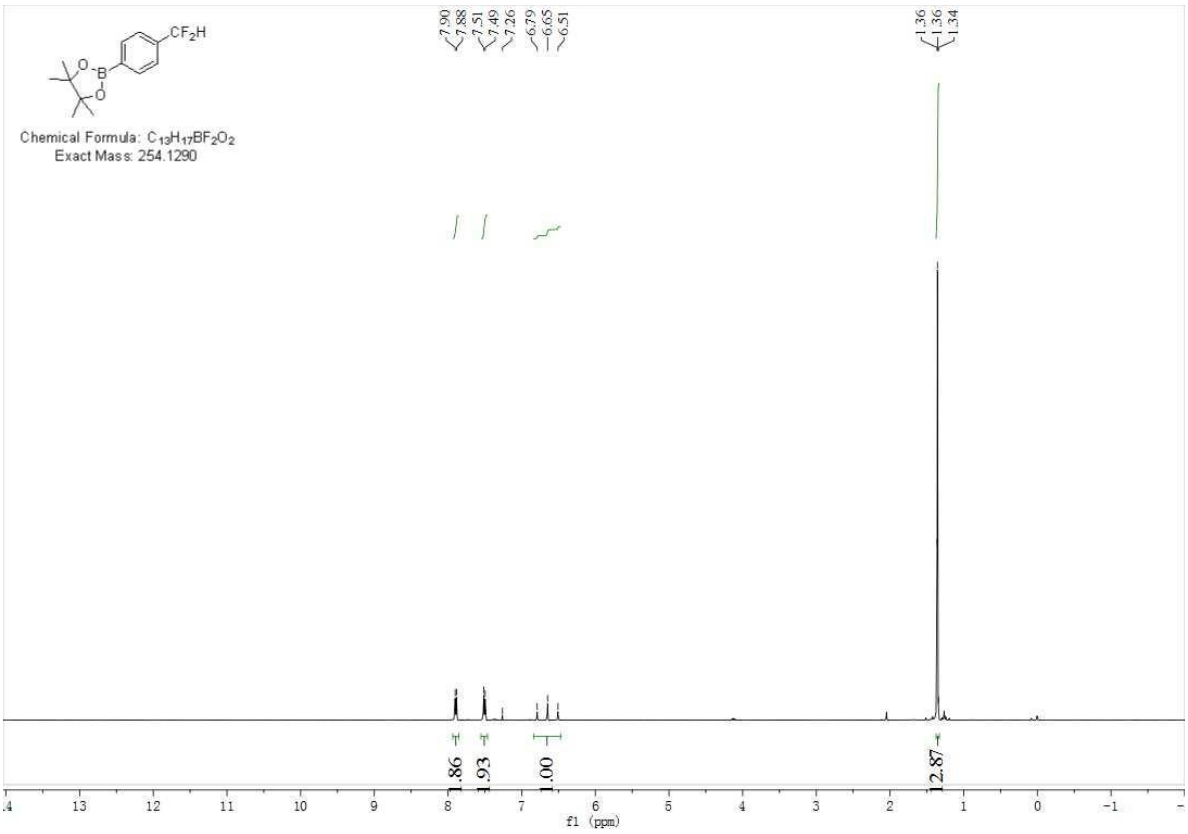

Supplementary Figure 62. **^{1}H-NMR of 2-(4-(difluoromethyl)phenyl)-4,4,5,5-tetramethyl-1,3,2-dioxaborolane (3w)**

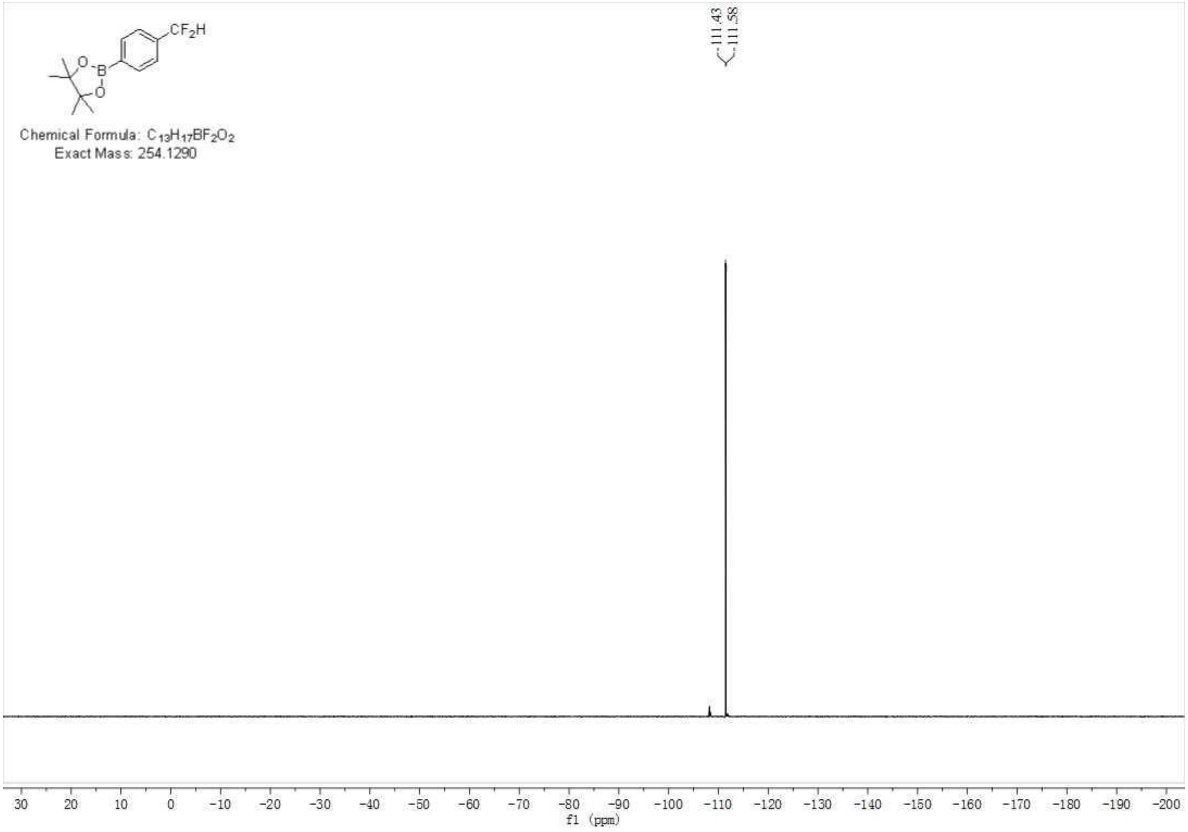

Supplementary Figure 63. **^{19}F-NMR of 2-(4-(difluoromethyl)phenyl)-4,4,5,5-tetramethyl-1,3,2-dioxaborolane (3w)**

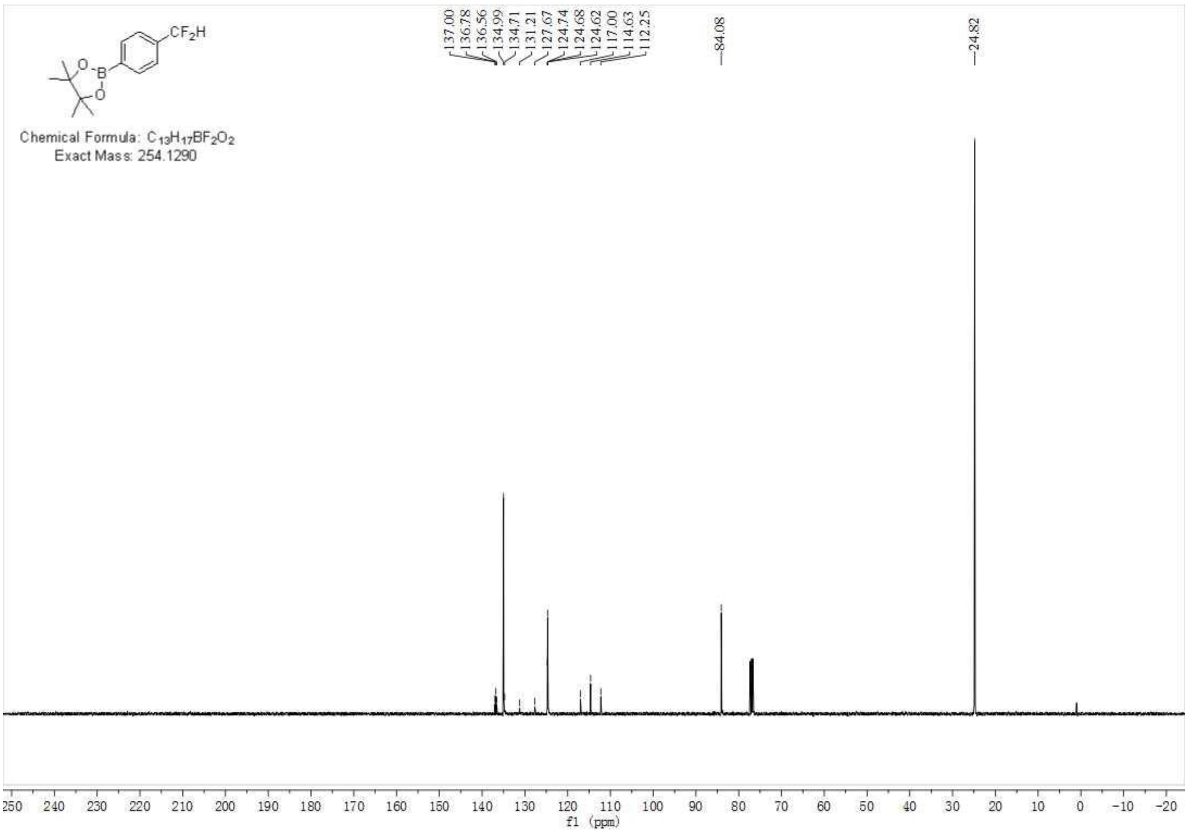

Supplementary Figure 64. **^{13}C-NMR of 2-(4-(difluoromethyl)phenyl)-4,4,5,5-tetramethyl-1,3,2-dioxaborolane (3w)**

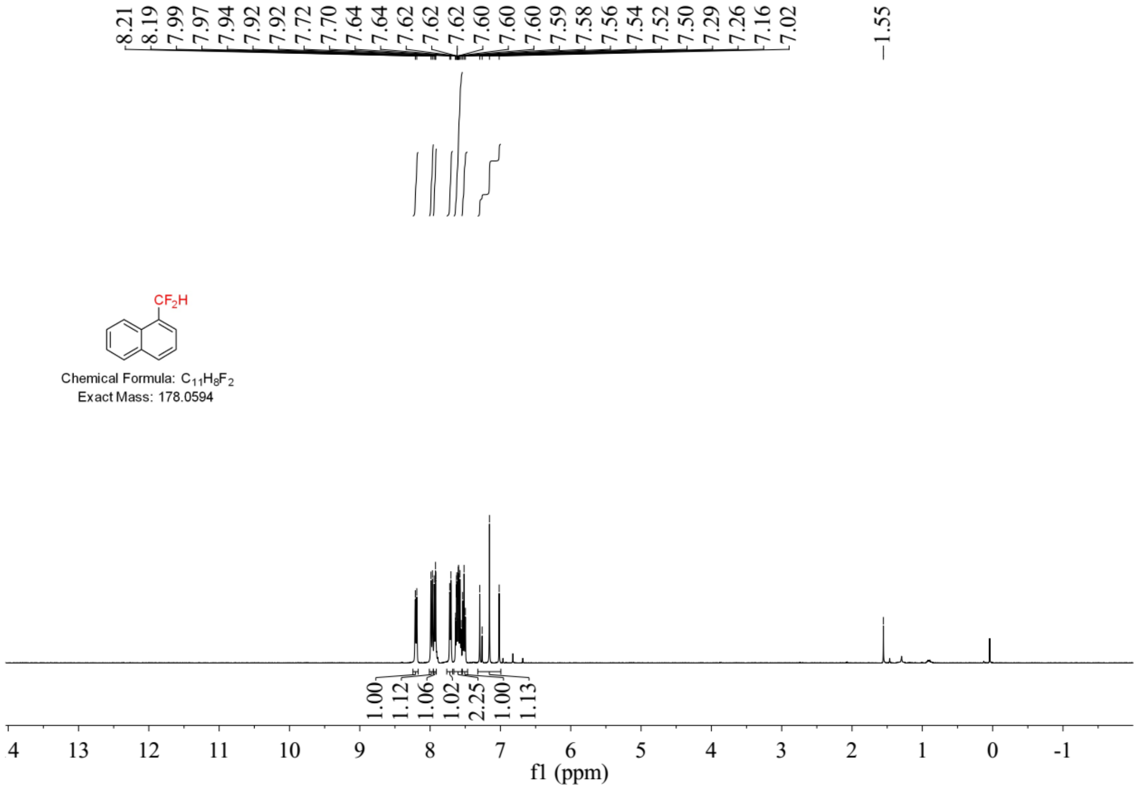

Supplementary Figure 65. ^{1}H-NMR of 1-(difluoromethyl)naphthalene (3x)

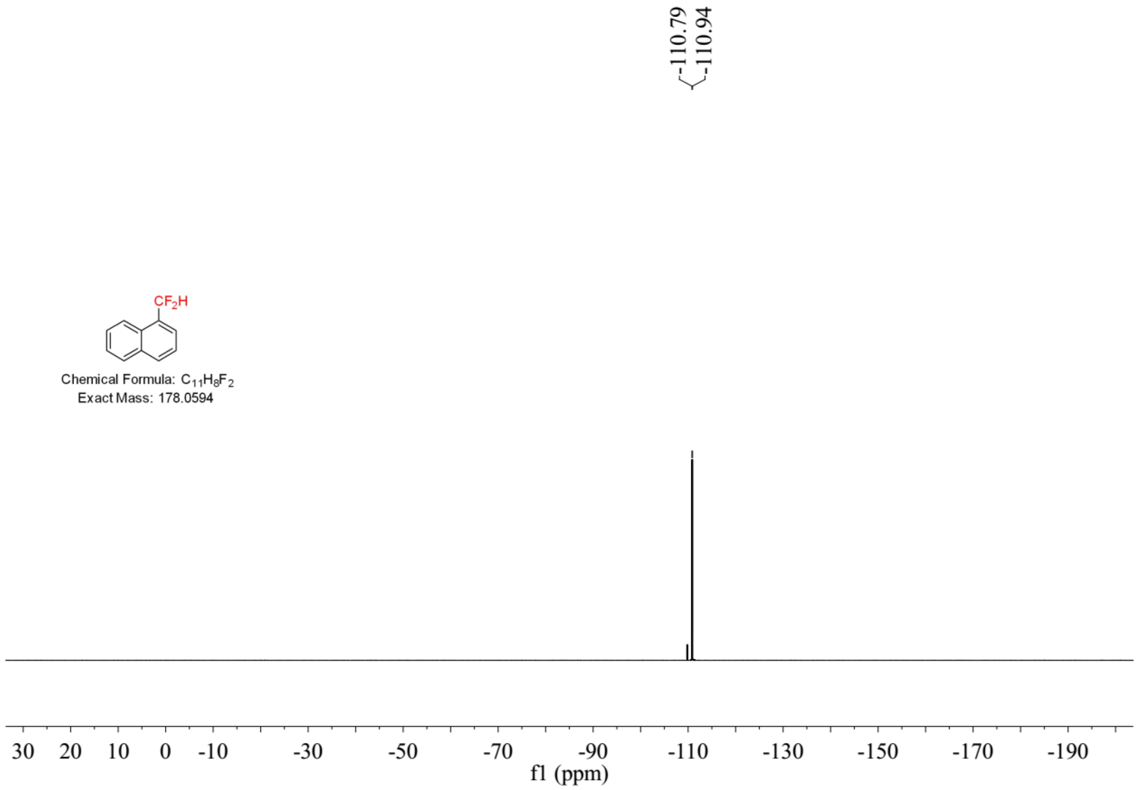

Supplementary Figure 66. ^{19}F-NMR of 1-(difluoromethyl)naphthalene (3x)

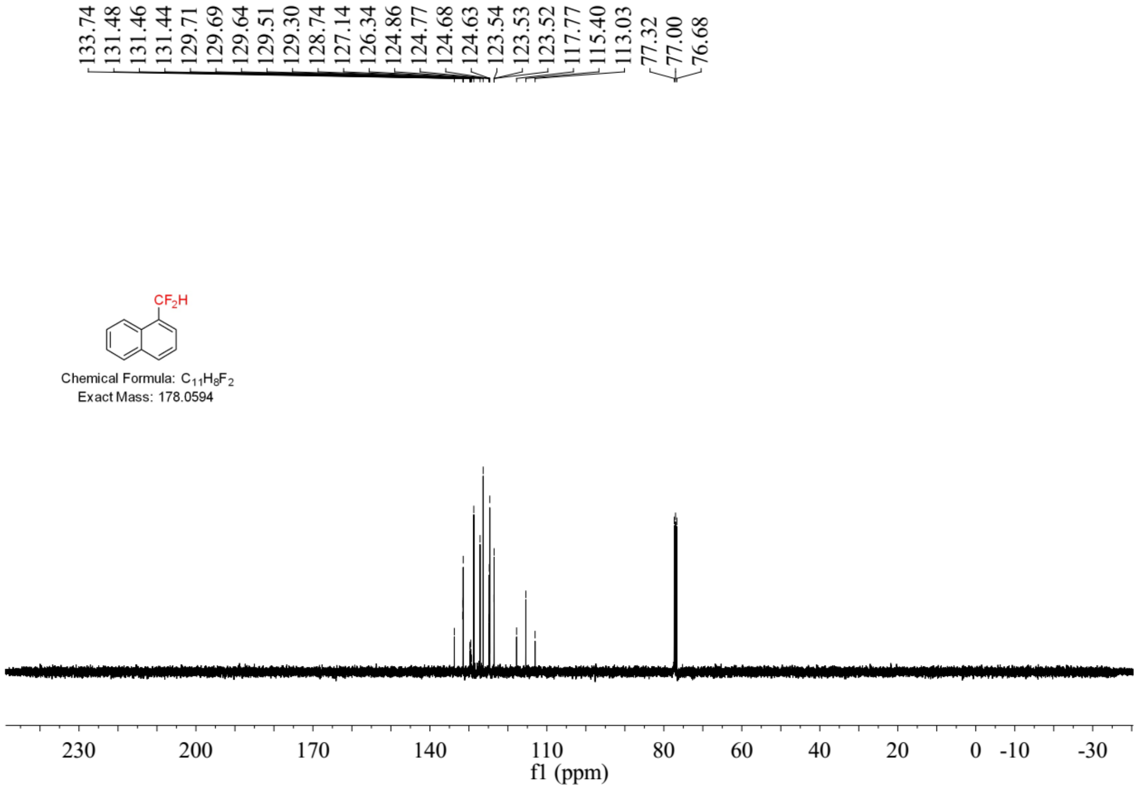

Supplementary Figure 67. ^{13}C-NMR of 1-(difluoromethyl)naphthalene (3x)

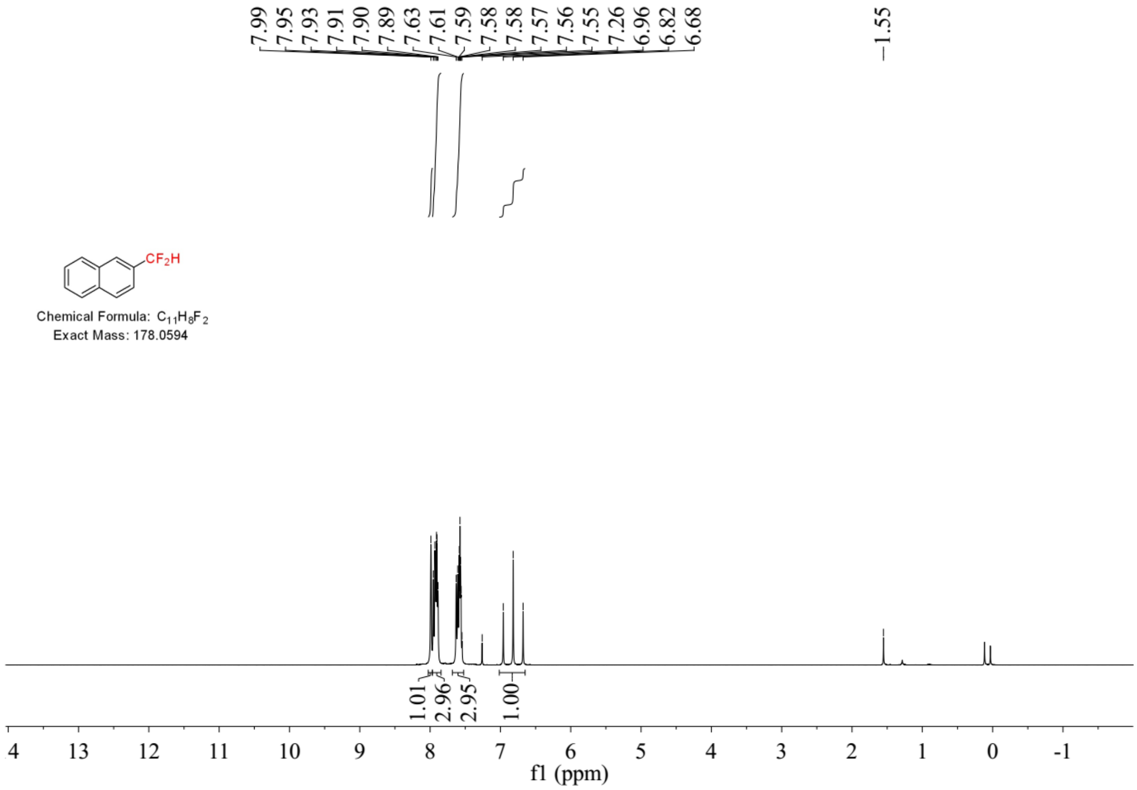

Supplementary Figure 68. ^{1}H-NMR of 2-(difluoromethyl)naphthalene (3y)

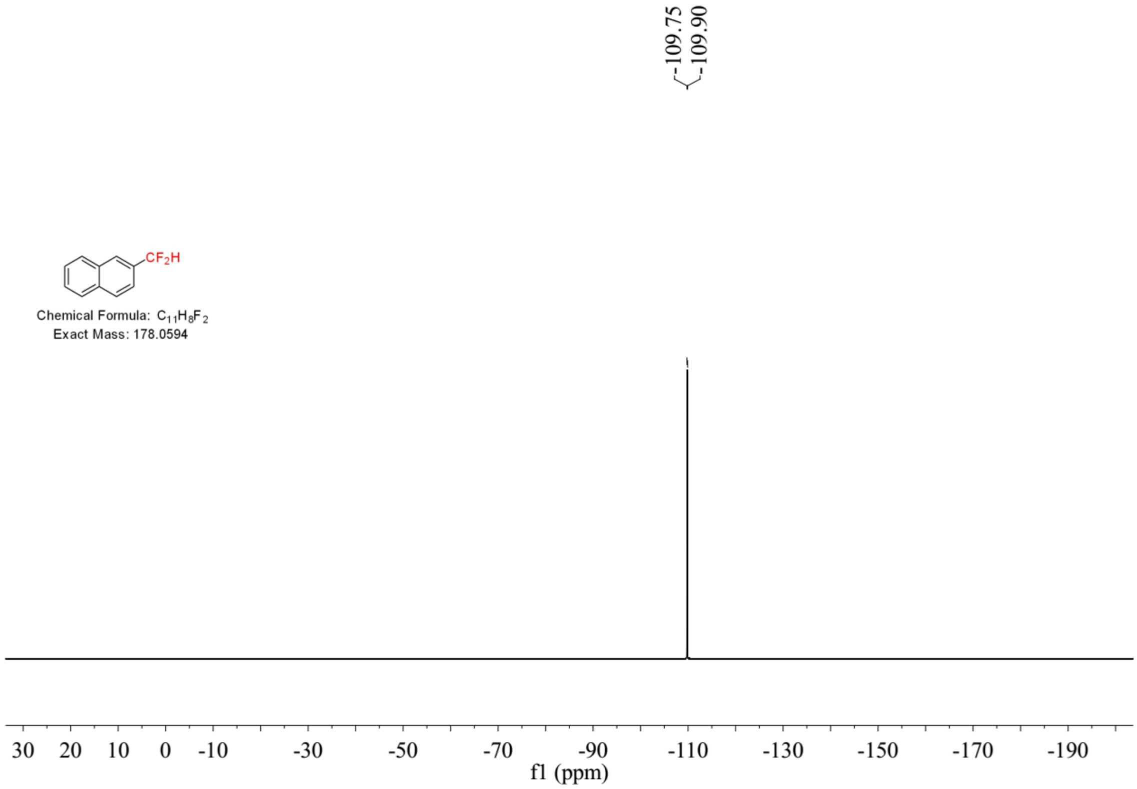

Supplementary Figure 69. ^{19}F-NMR of 2-(difluoromethyl)naphthalene (3y)

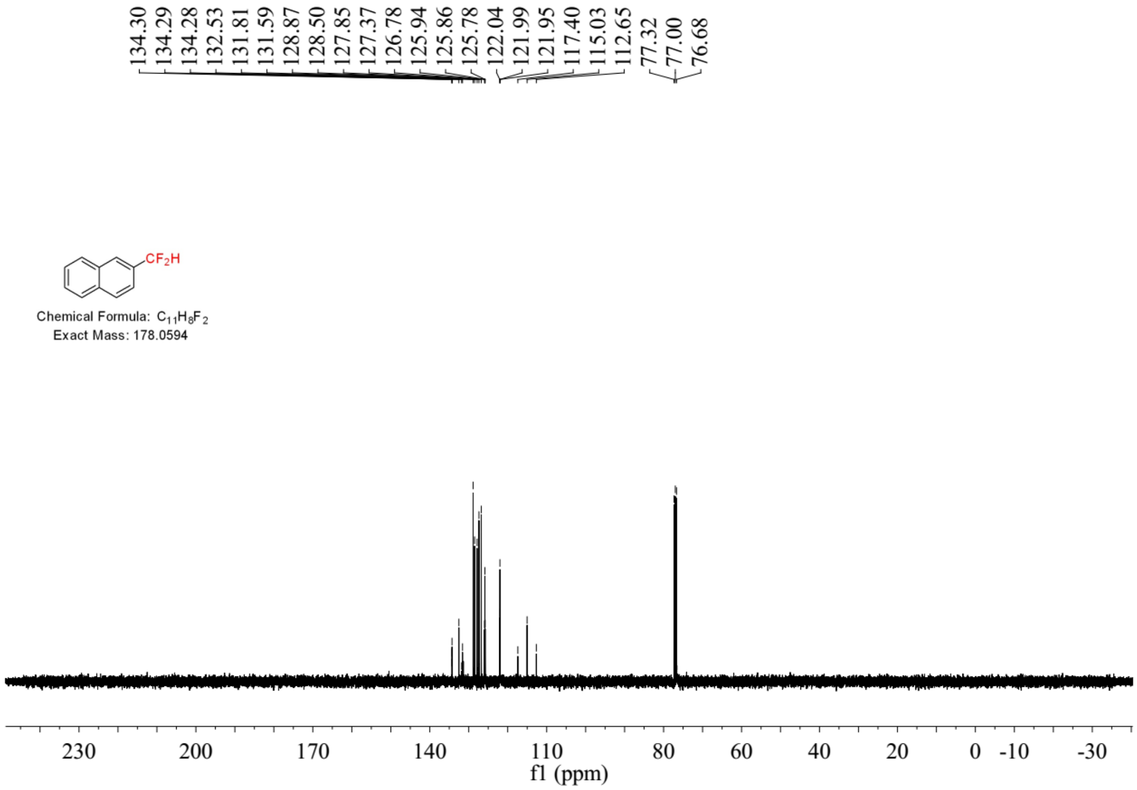

Supplementary Figure 70. ^{13}C-NMR of 2-(difluoromethyl)naphthalene (3y)

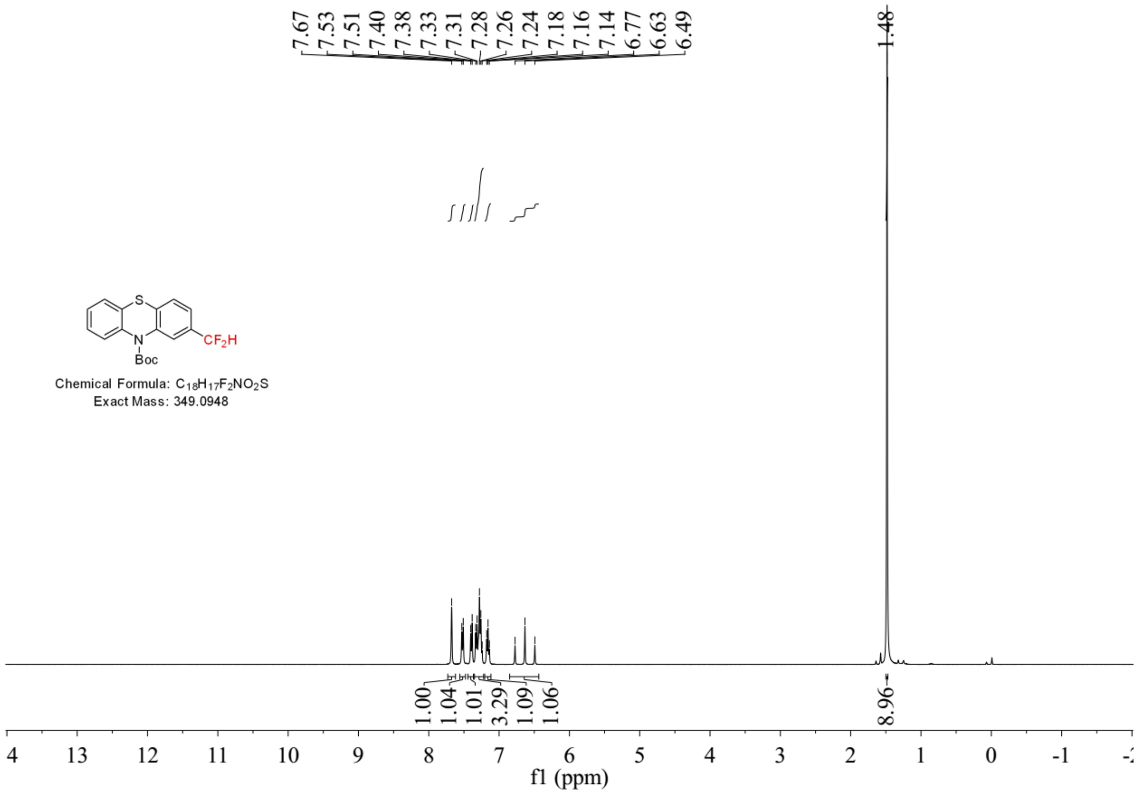

Supplementary Figure 71. ¹H-NMR of *tert*-butyl 2-(difluoromethyl)-10H-phenothiazine-10-carboxylate (5a)

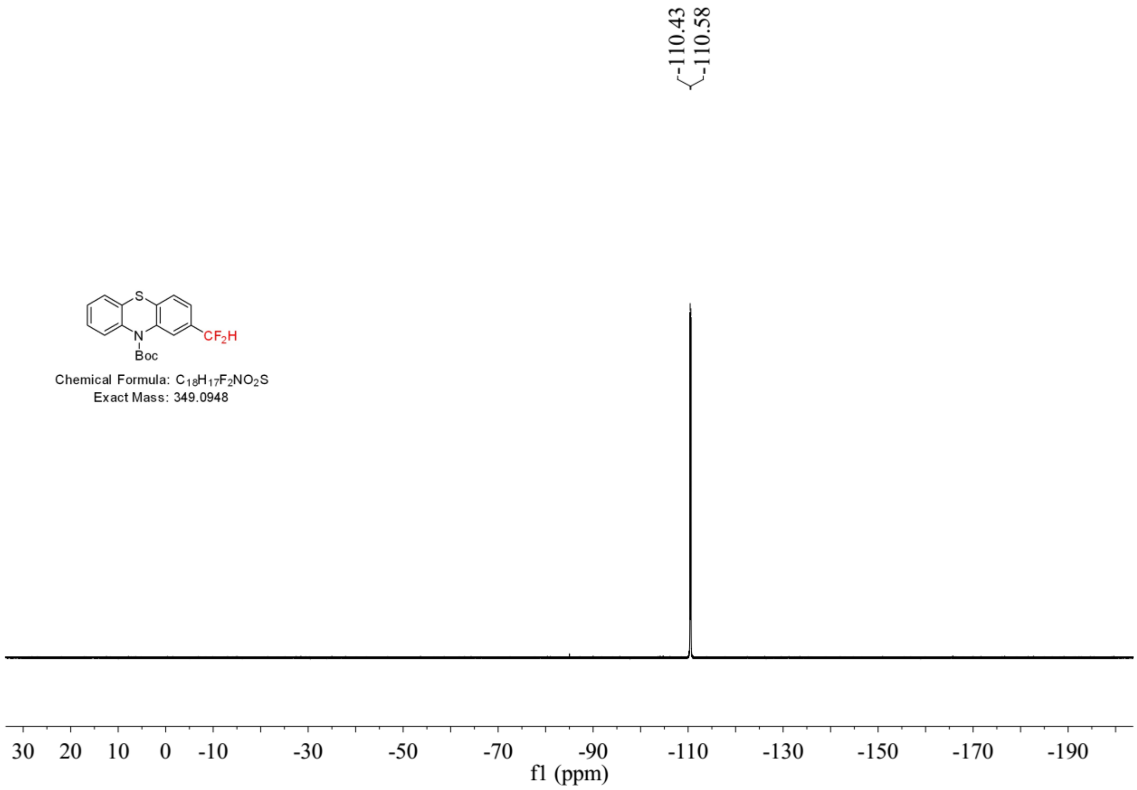

Supplementary Figure 72. ¹⁹F-NMR of *tert*-butyl 2-(difluoromethyl)-10H-phenothiazine-10-carboxylate (5a)

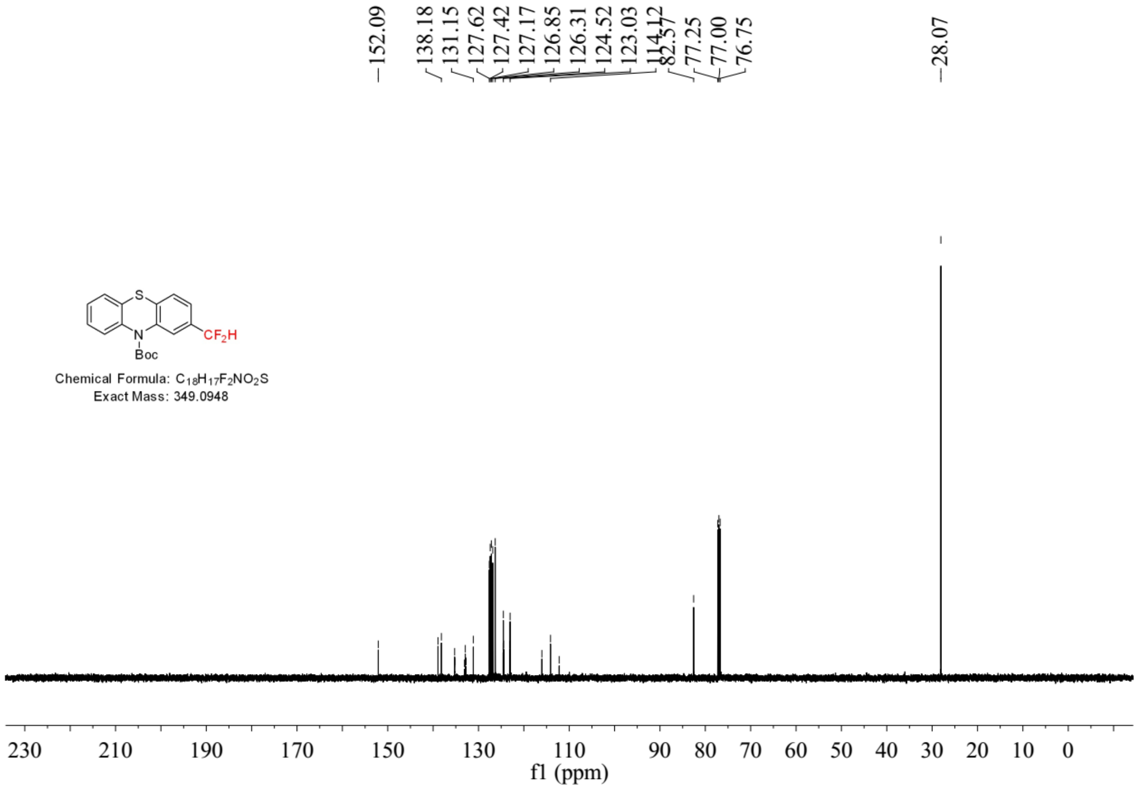

Supplementary Figure 73. ^{13}C-NMR of *tert*-butyl 2-(difluoromethyl)-10H-phenothiazine-10-carboxylate (5a)

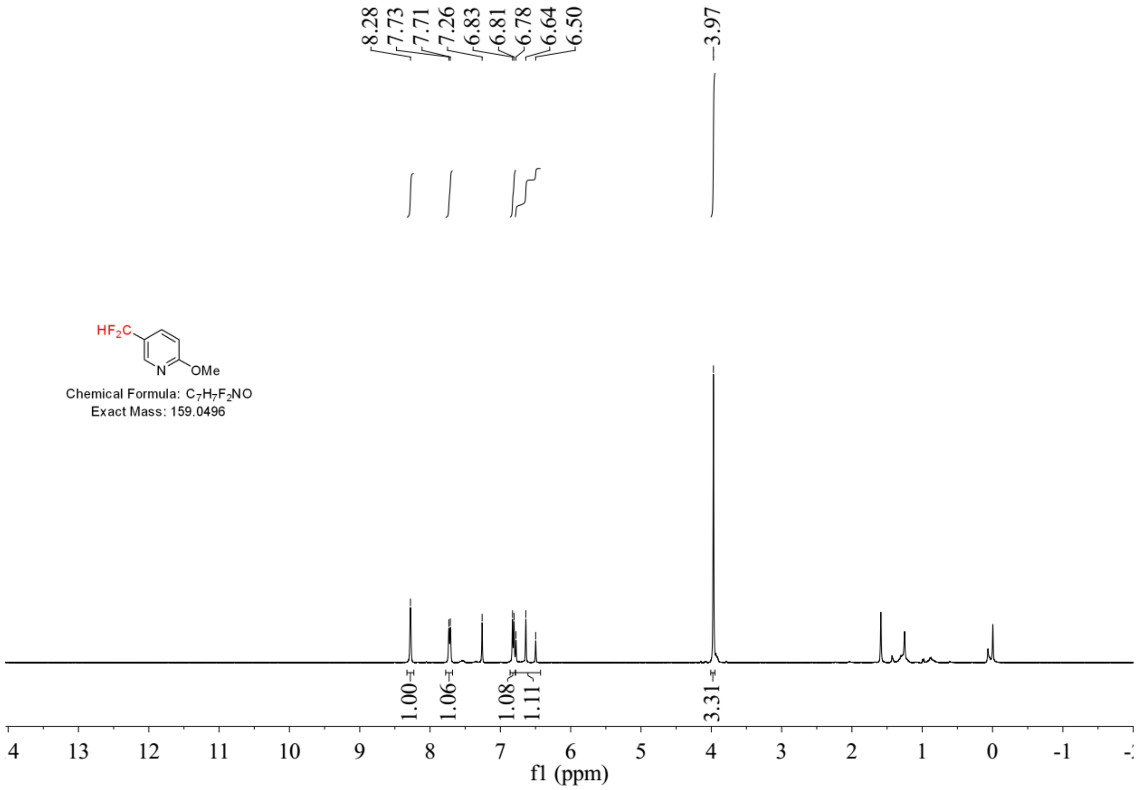

Supplementary Figure 74. ^{1}H-NMR of 5-(difluoromethyl)-2-methoxypyridine (5b)

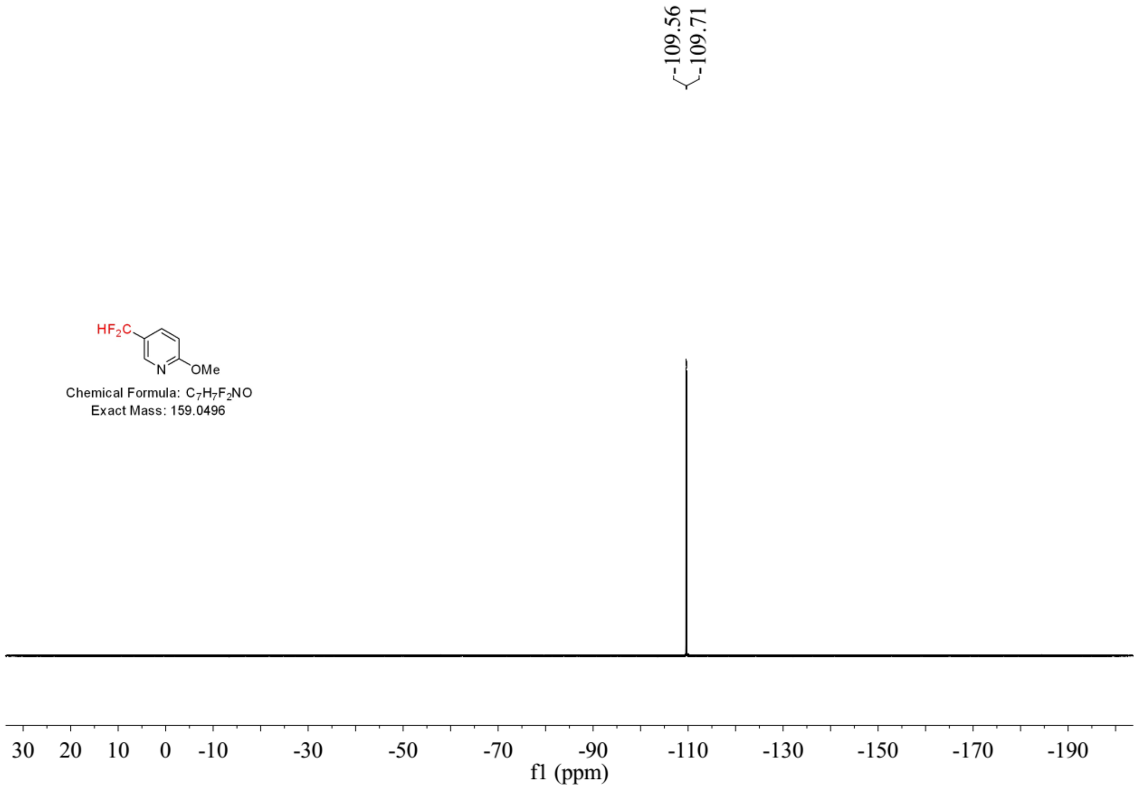

Supplementary Figure 75. ^{19}F-NMR of 5-(difluoromethyl)-2-methoxypyridine (5b)

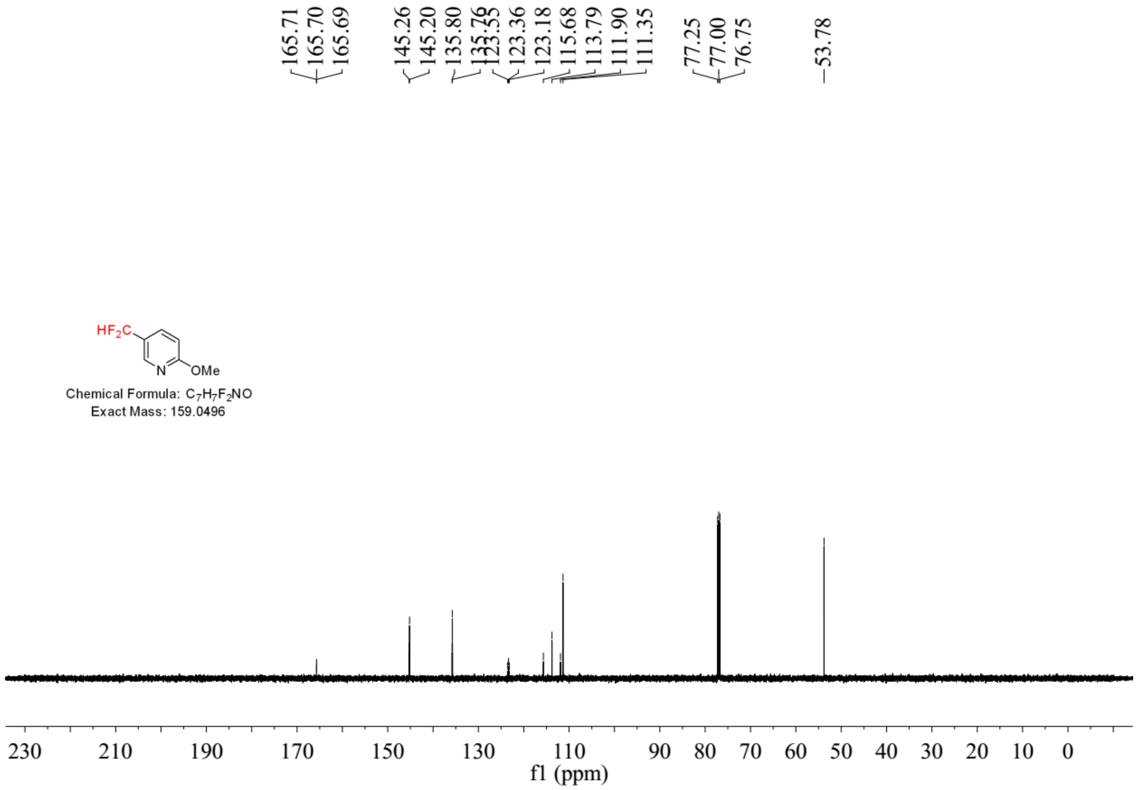

Supplementary Figure 76. ^{13}C-NMR of 5-(difluoromethyl)-2-methoxypyridine (5b)

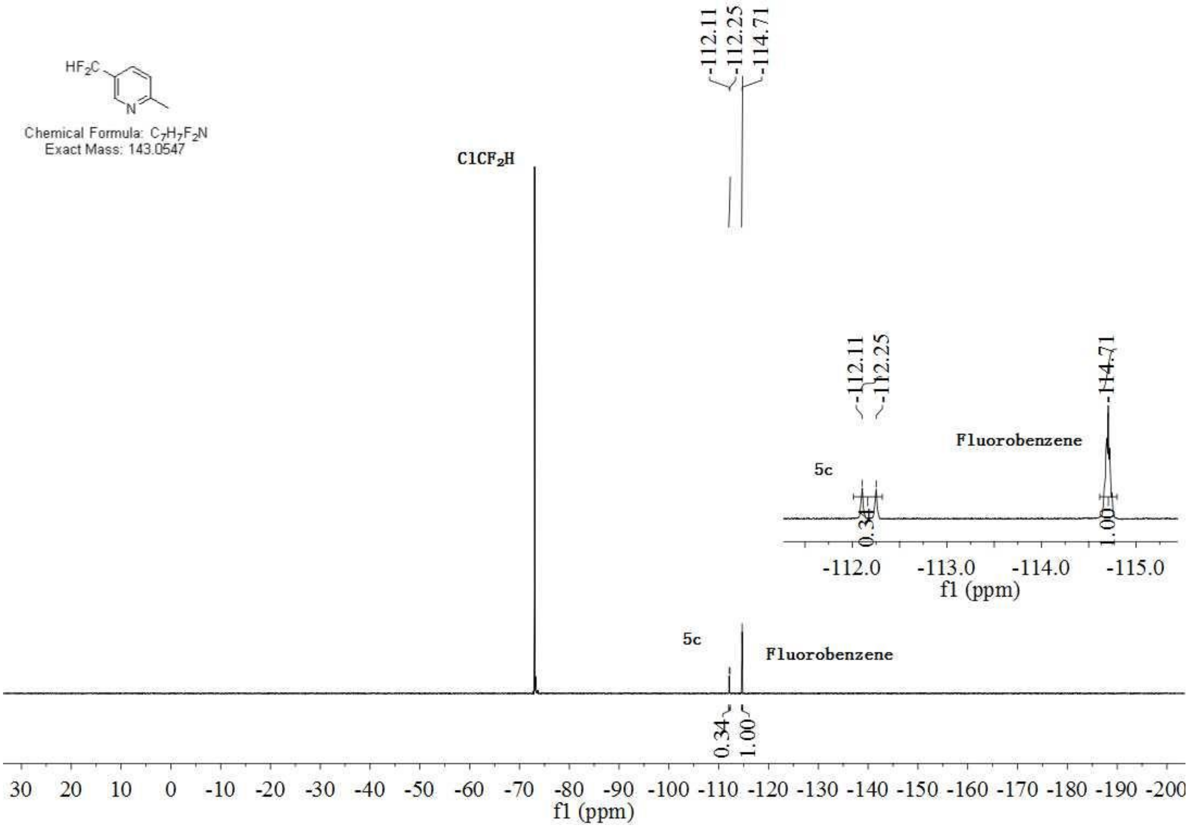

Supplementary Figure 77. Crude ^{19}F NMR of 5-(difluoromethyl)-2-methylpyridine (5c)

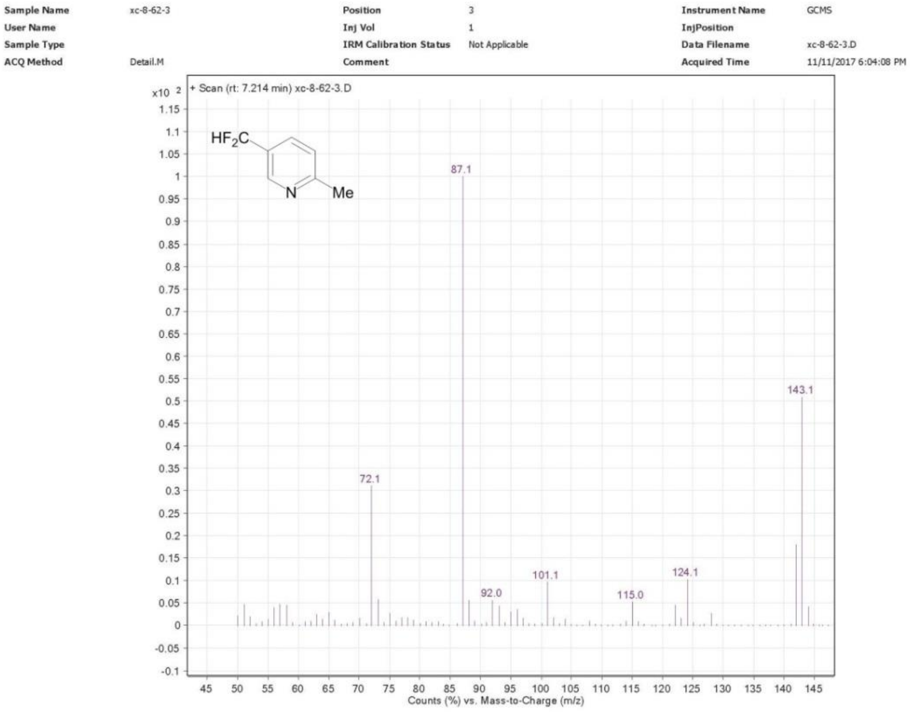

Supplementary Figure 78. MS(EI) of 5-(difluoromethyl)-2-methylpyridine (5c)

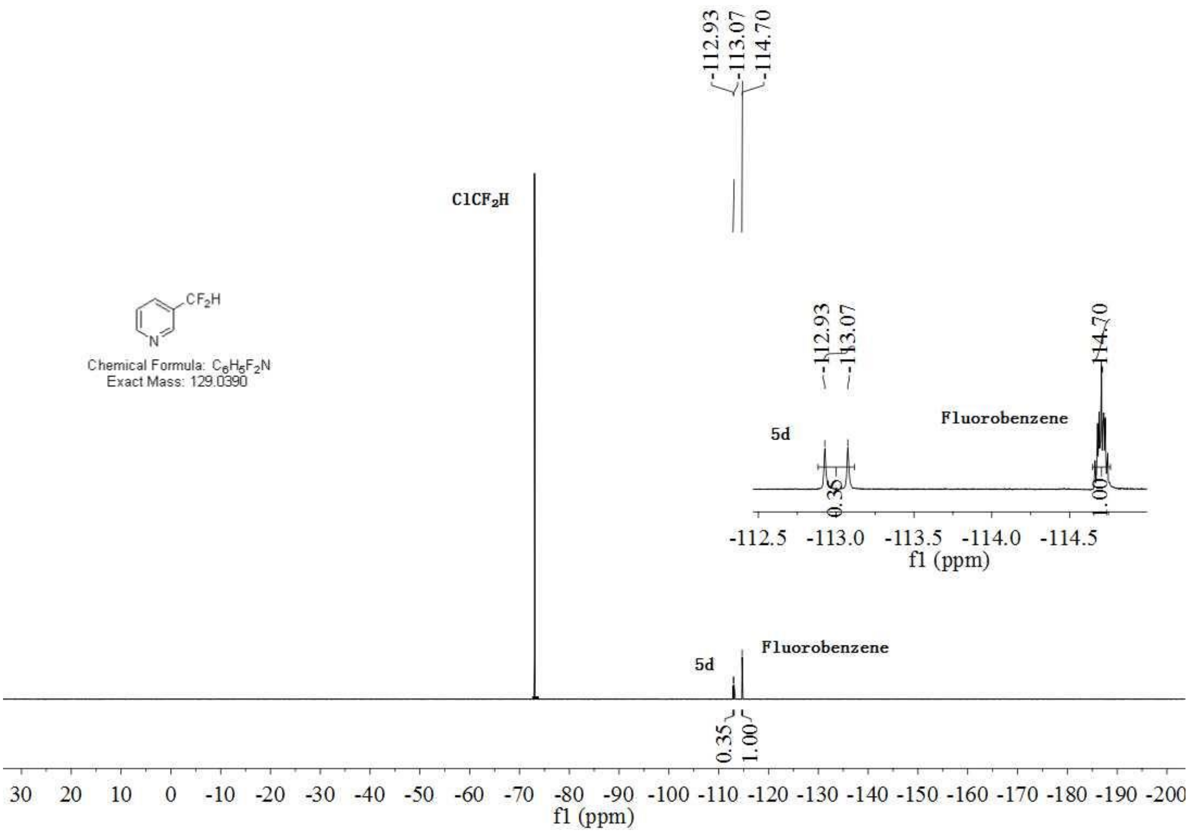

Supplementary Figure 79. Crude ^{19}F NMR of 3-(difluoromethyl)pyridine (5d)

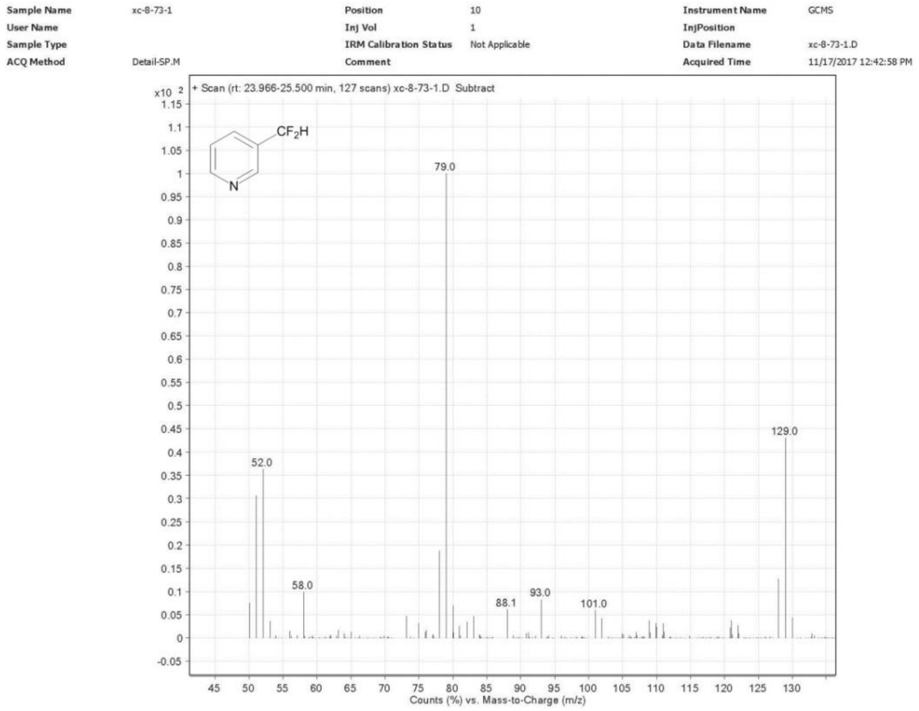

Supplementary Figure 80. MS(EI) of 3-(difluoromethyl)pyridine (5d)

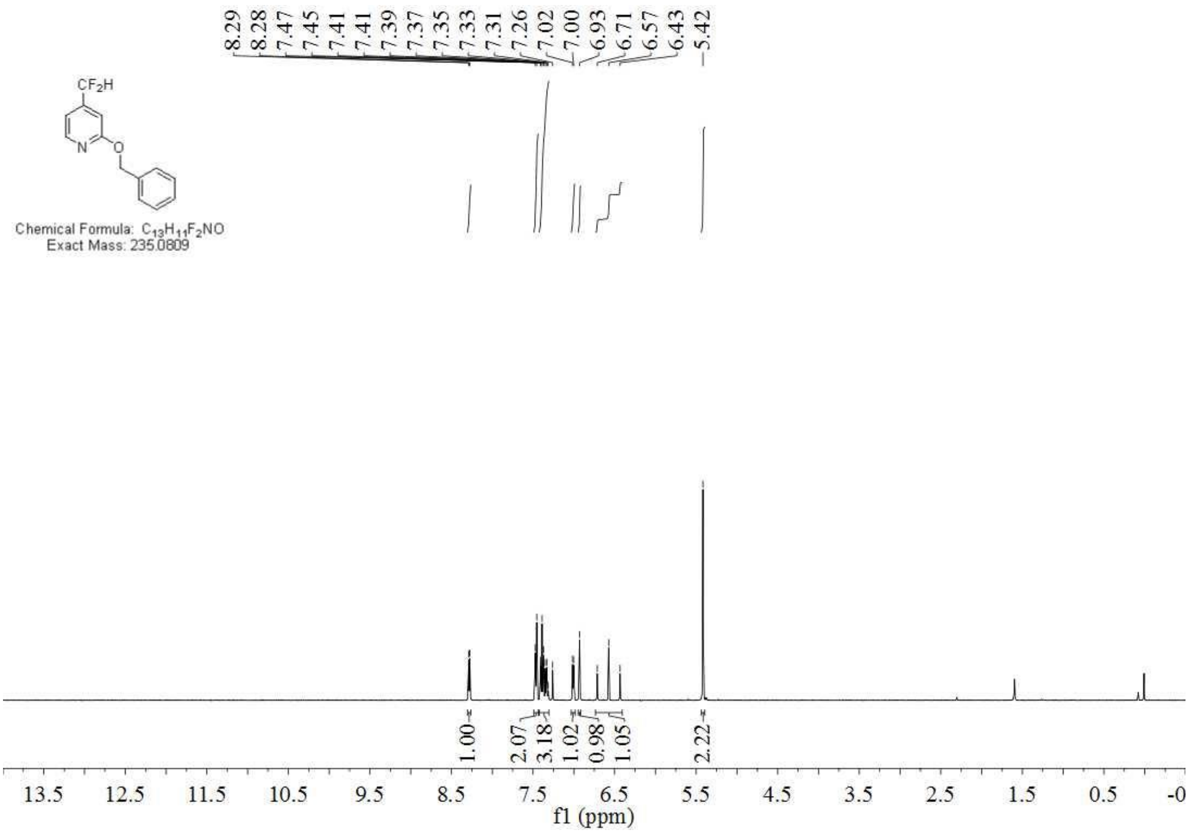

Supplementary Figure 81. ^{1}H-NMR of 2-(benzyloxy)-4-(difluoromethyl)pyridine (5e)

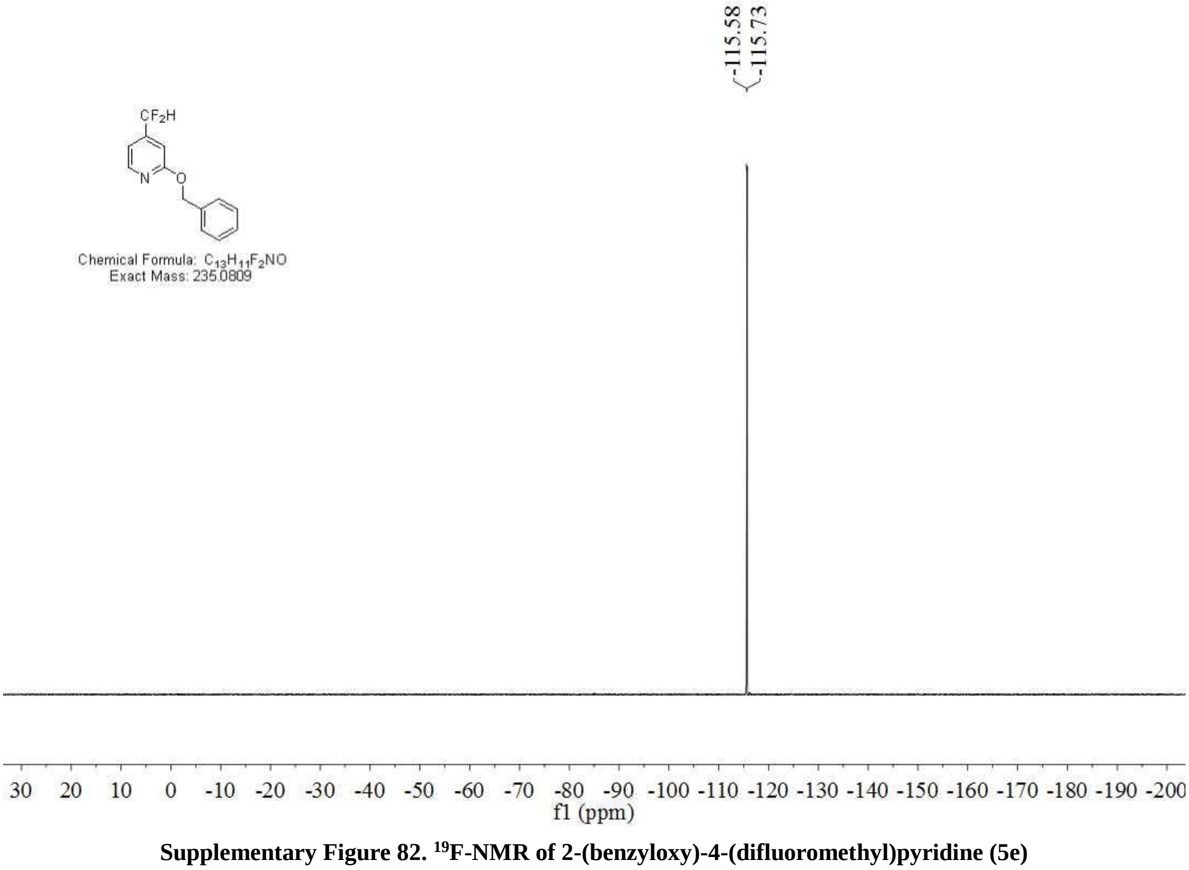

Supplementary Figure 82. ^{19}F-NMR of 2-(benzyloxy)-4-(difluoromethyl)pyridine (5e)

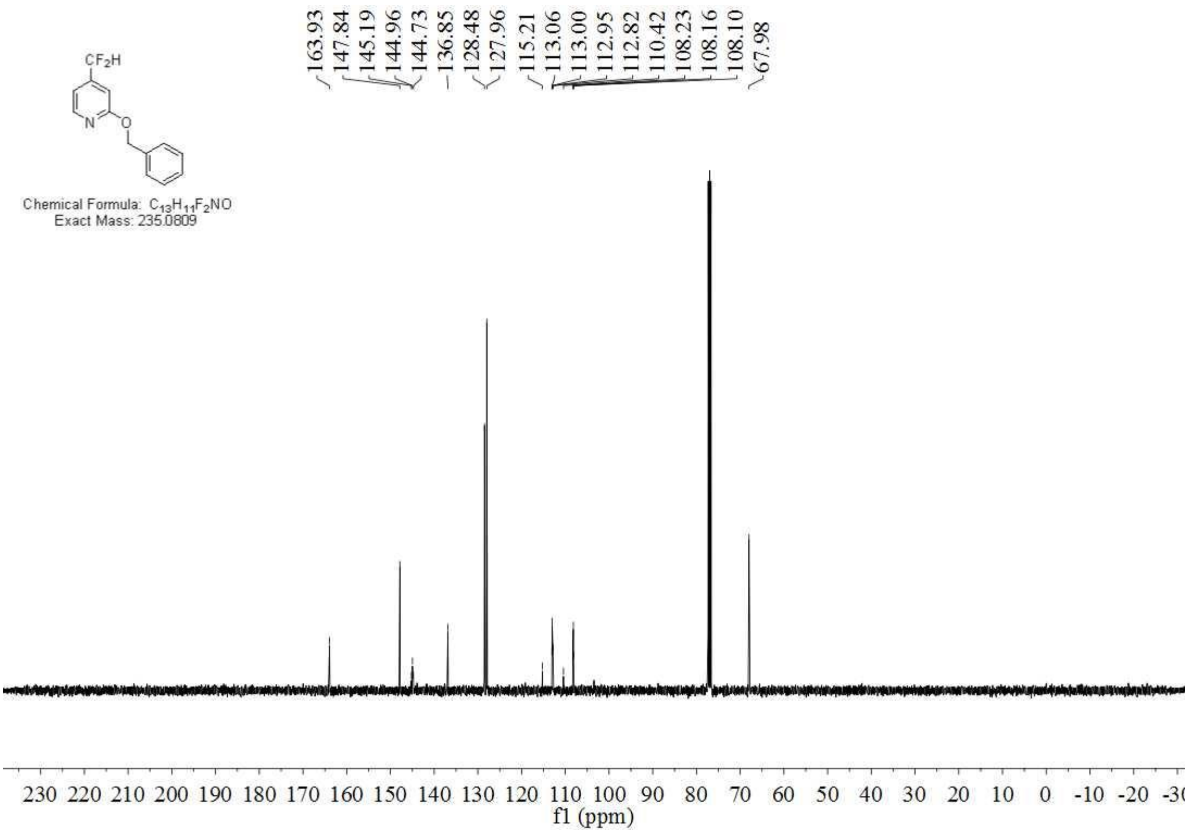

Supplementary Figure 83. ^{13}**C-NMR of 2-(benzyloxy)-4-(difluoromethyl)pyridine (5e)**

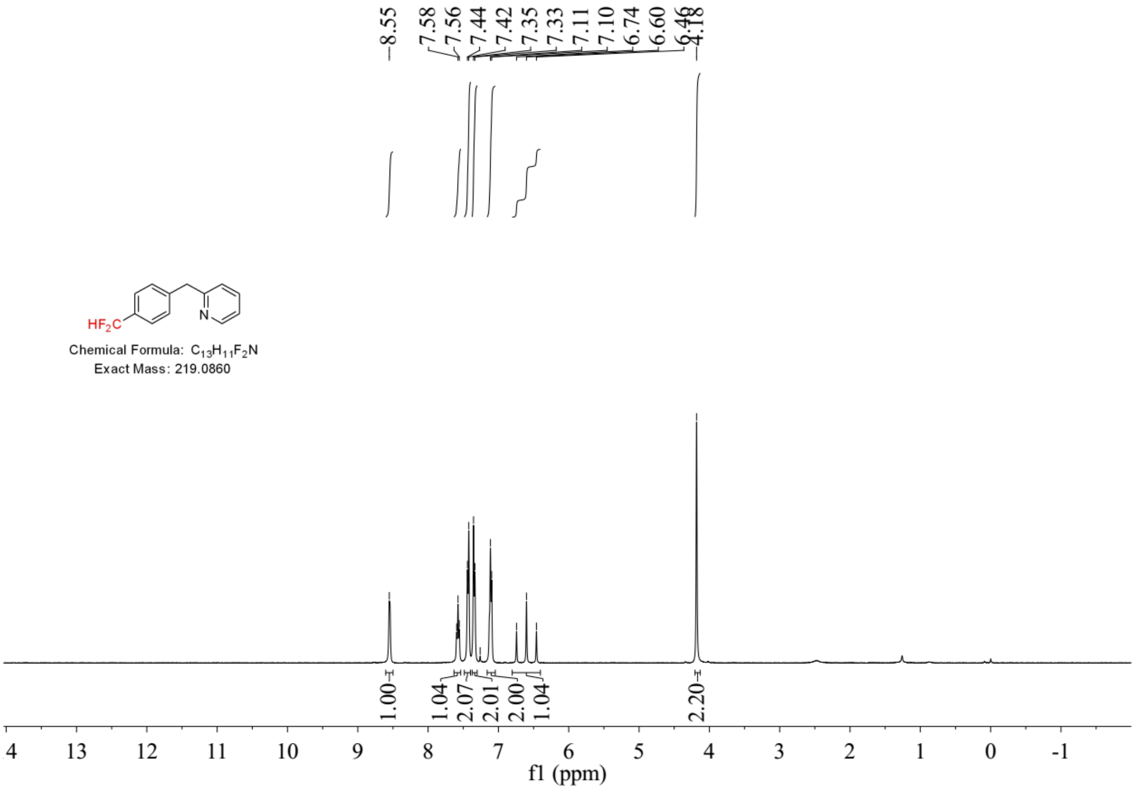

Supplementary Figure 84. ^{1}**H-NMR of 2-(4-(difluoromethyl)benzyl)pyridine (5f)**

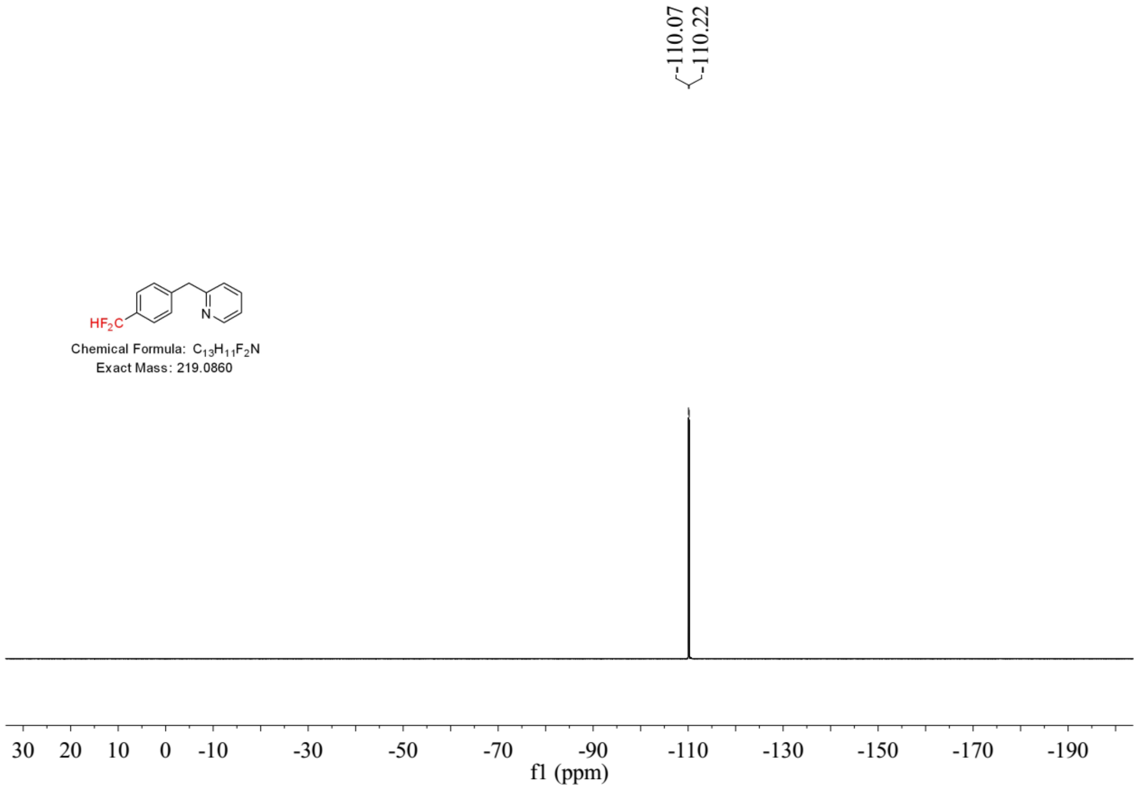

Supplementary Figure 85. ^{19}F-NMR of 2-(4-(difluoromethyl)benzyl)pyridine (5f)

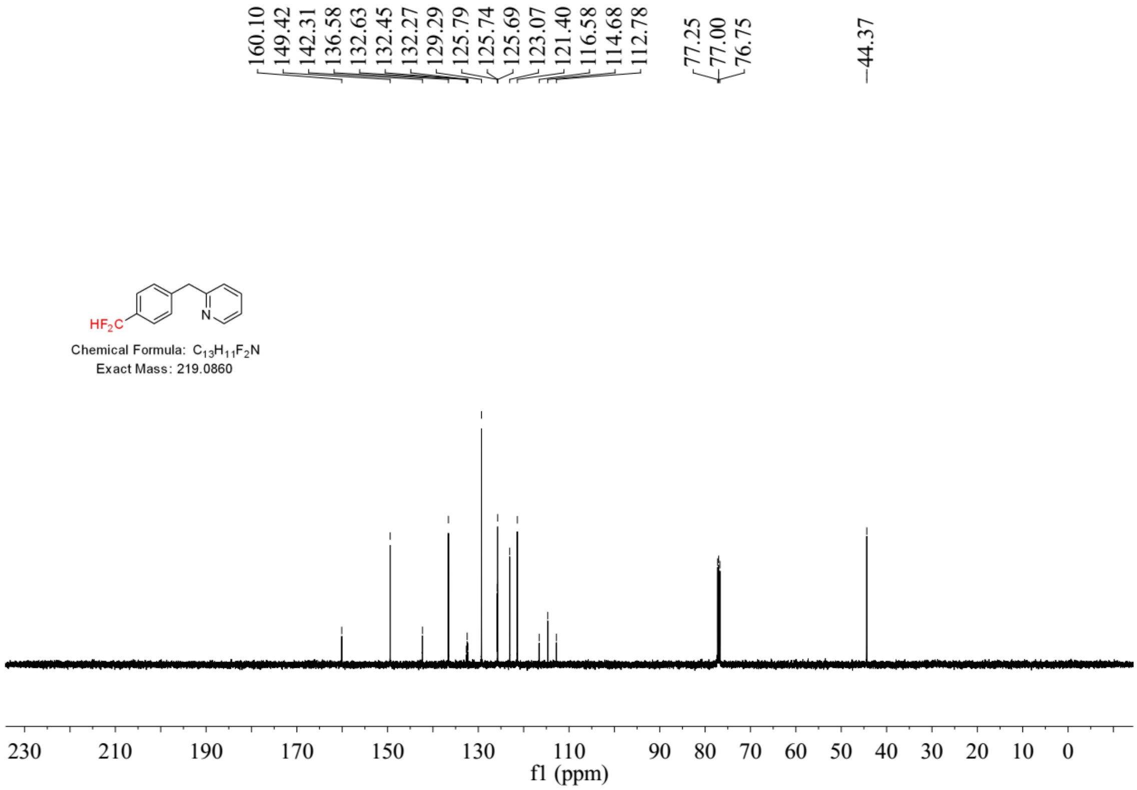

Supplementary Figure 86. ^{13}C-NMR of 2-(4-(difluoromethyl)benzyl)pyridine (5f)

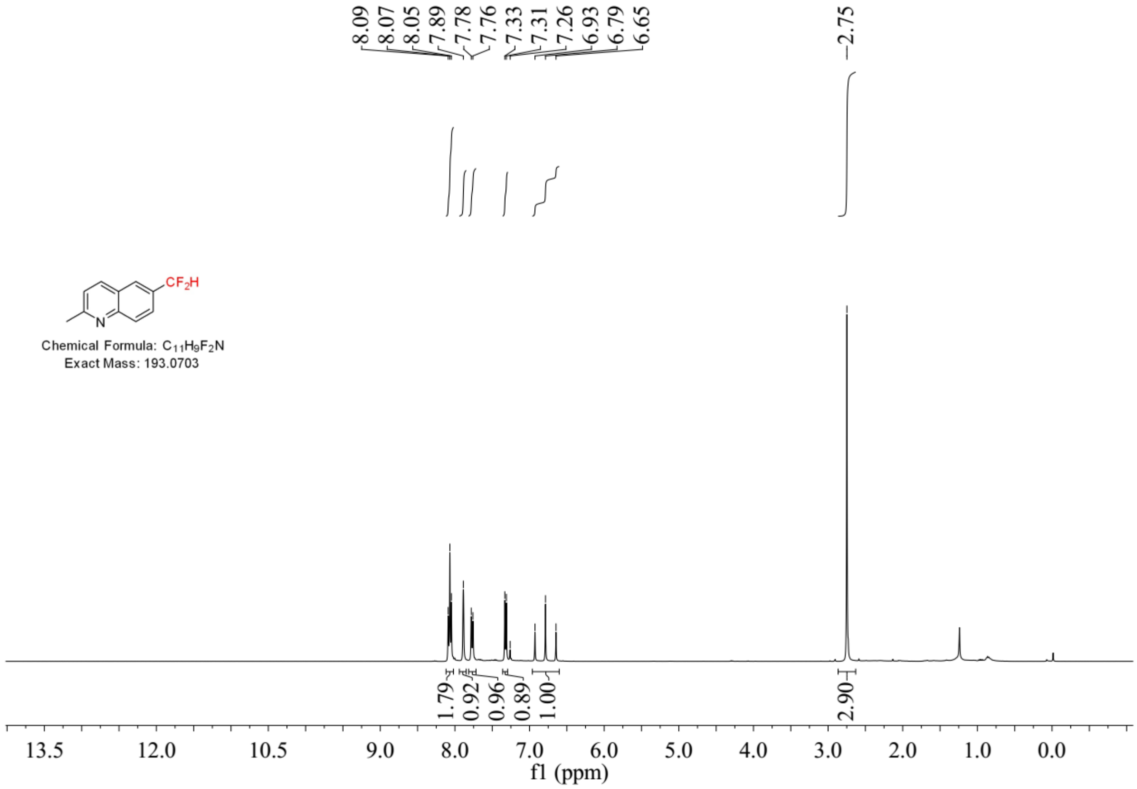

Supplementary Figure 87. ^{1}H-NMR of 6-(difluoromethyl)-2-methylquinoline (5g)

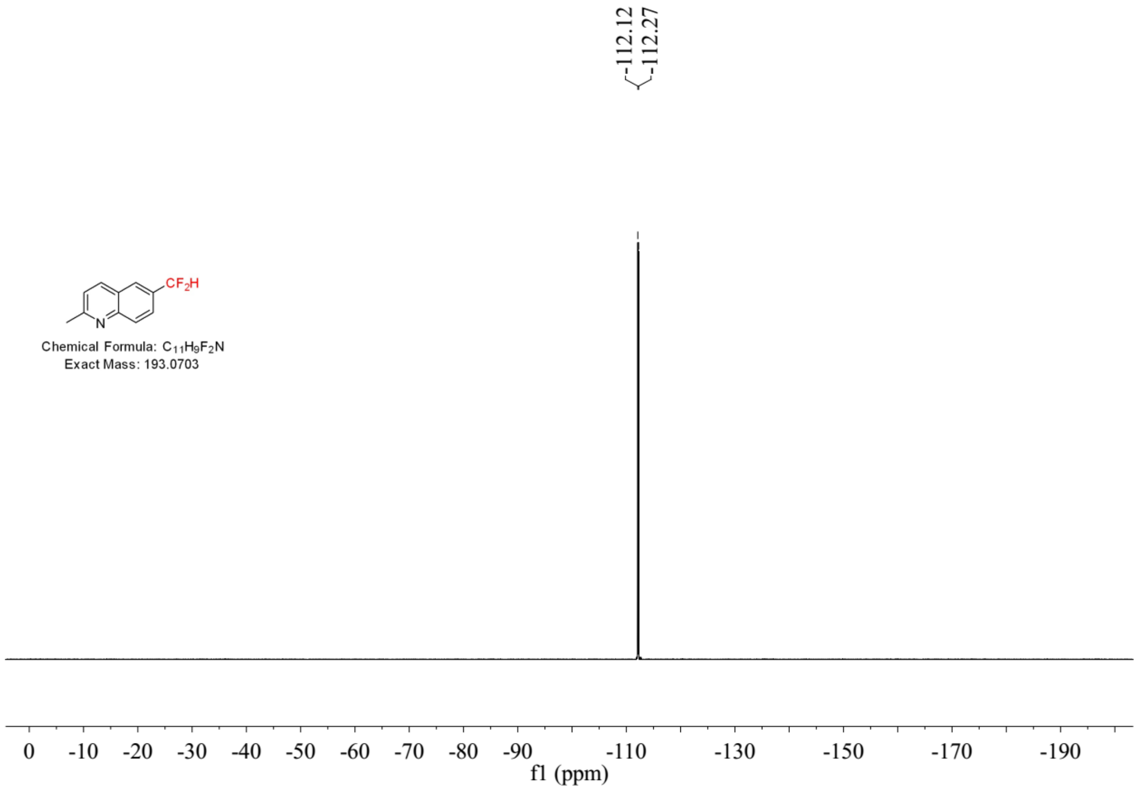

Supplementary Figure 88. ^{19}F-NMR of 6-(difluoromethyl)-2-methylquinoline (5g)

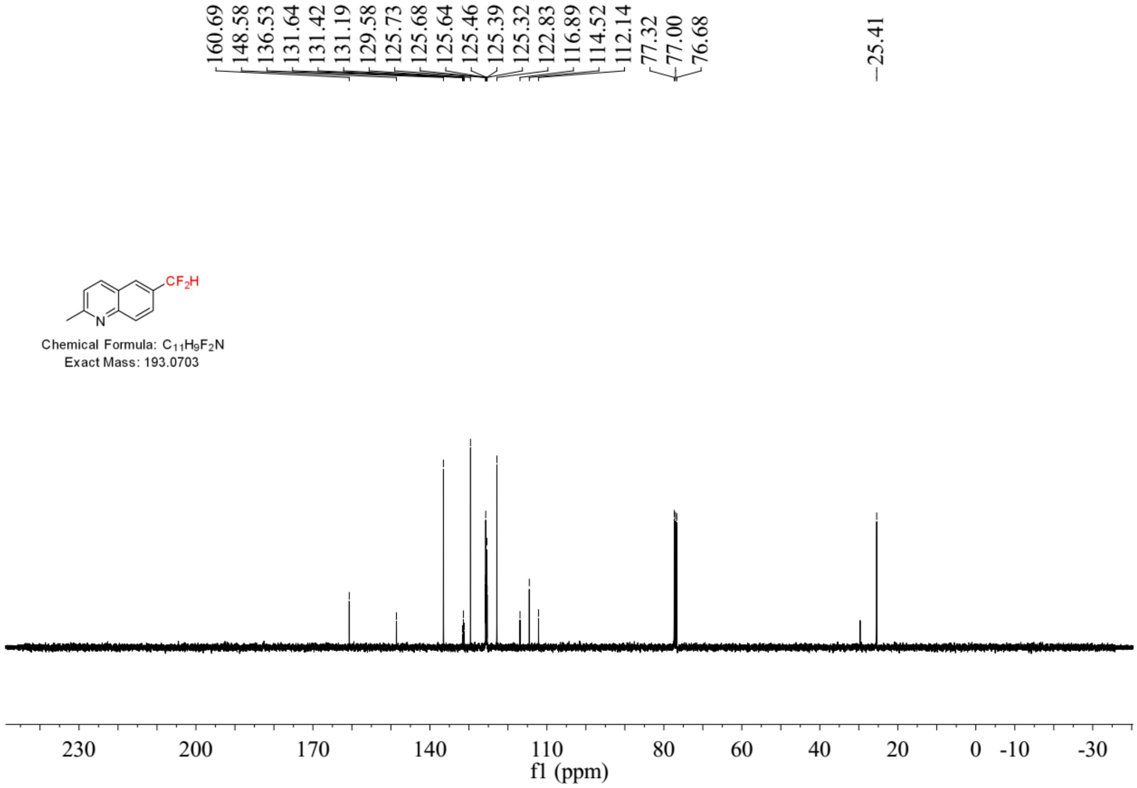

Supplementary Figure 89. ^{13}C-NMR of 6-(difluoromethyl)-2-methylquinoline (5g)

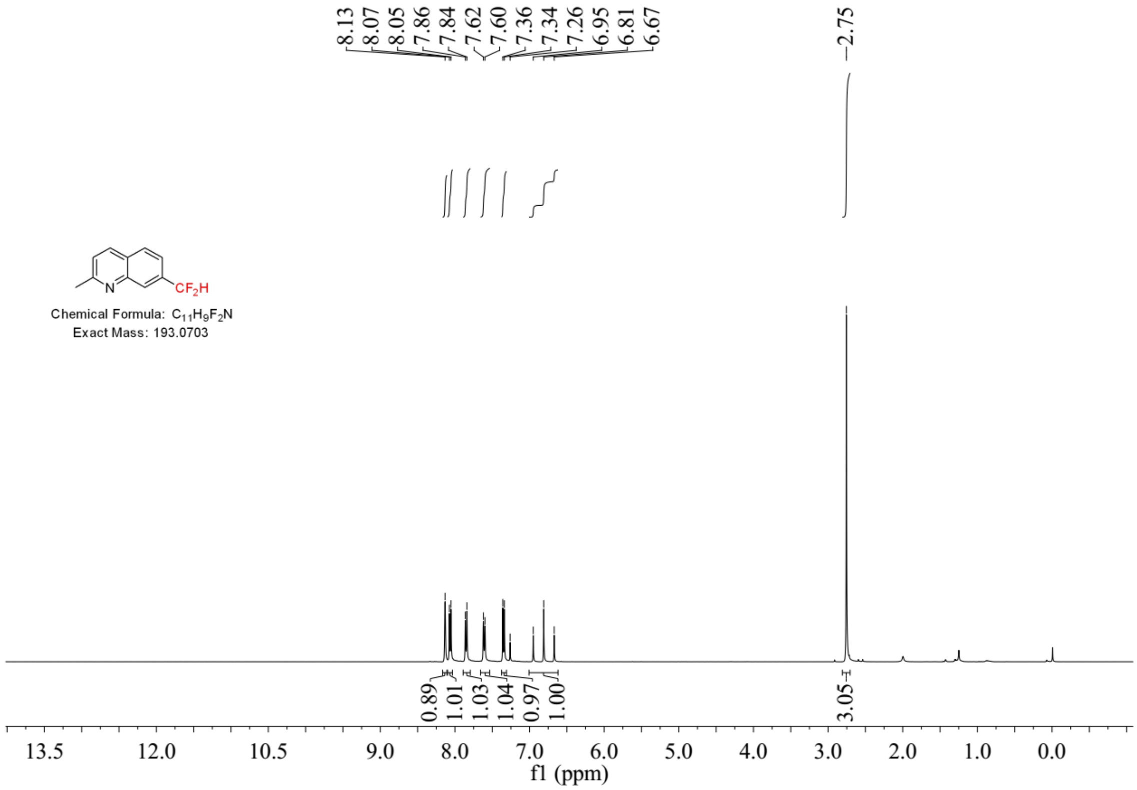

Supplementary Figure 90. ^{1}H-NMR of 7-(difluoromethyl)-2-methylquinoline (5h)

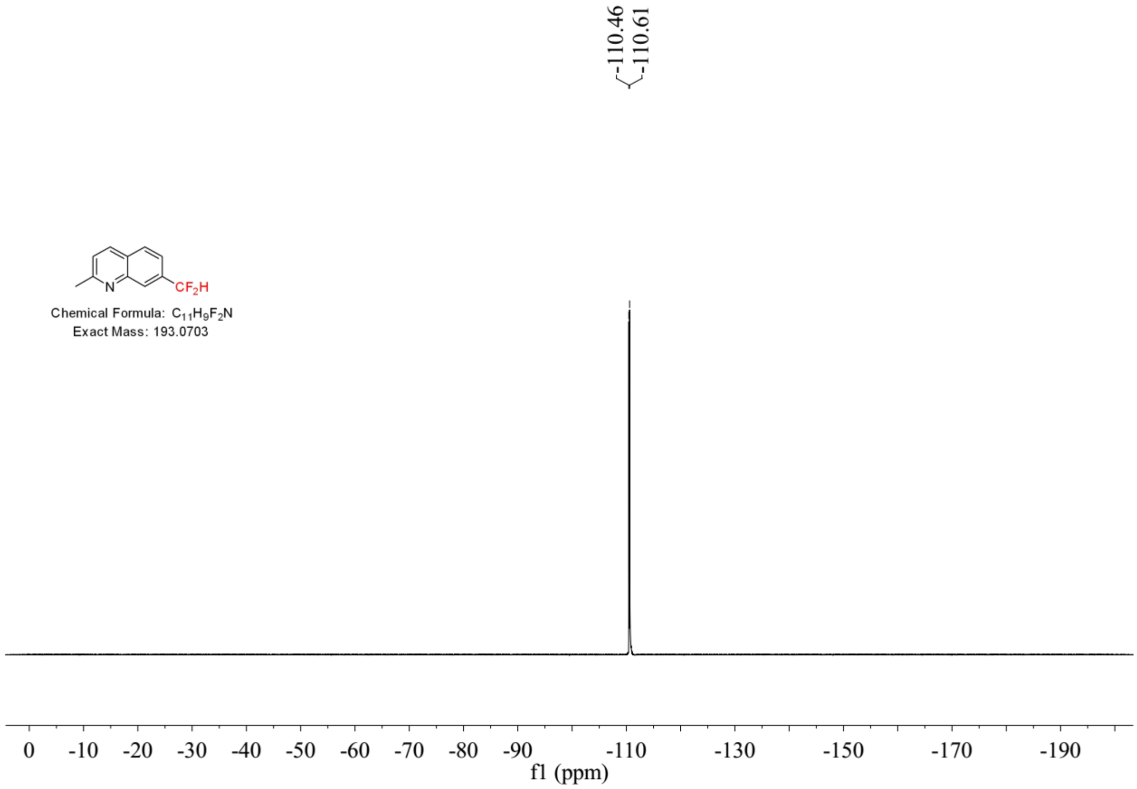

Supplementary Figure 91. **^{19}F-NMR of 7-(difluoromethyl)-2-methylquinoline (5h)**

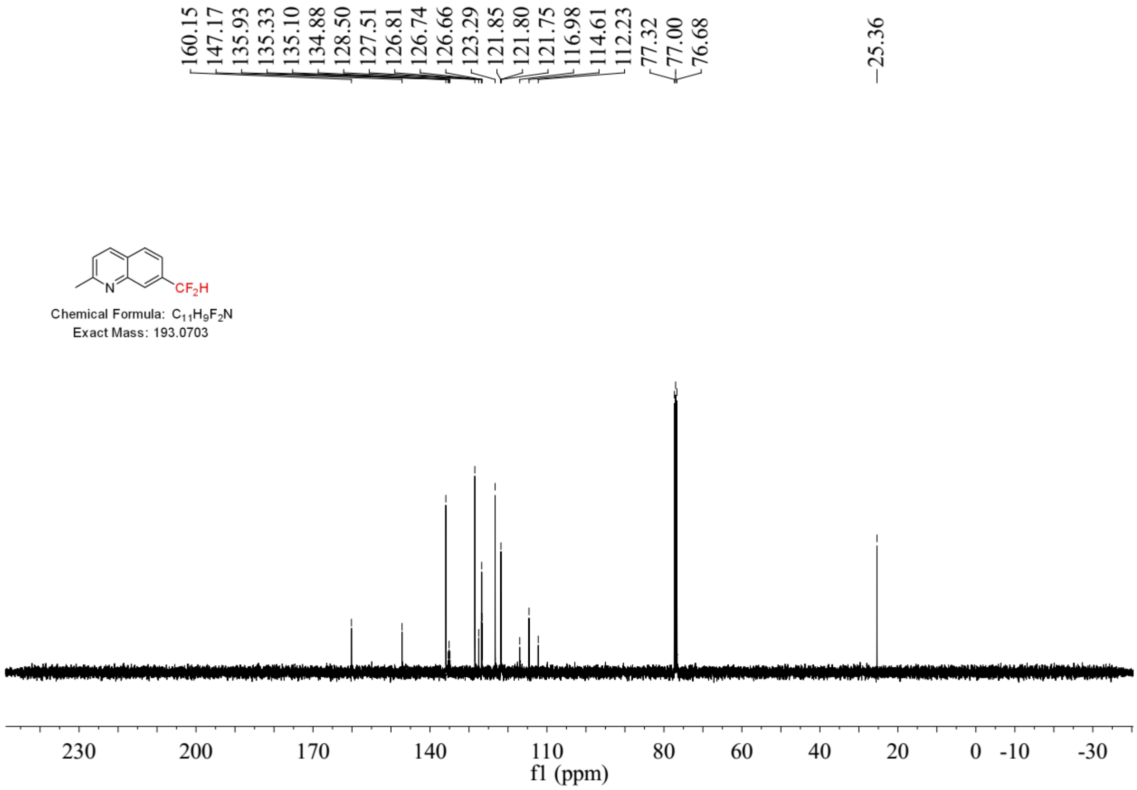

Supplementary Figure 92. **^{13}C-NMR of 7-(difluoromethyl)-2-methylquinoline (5h)**

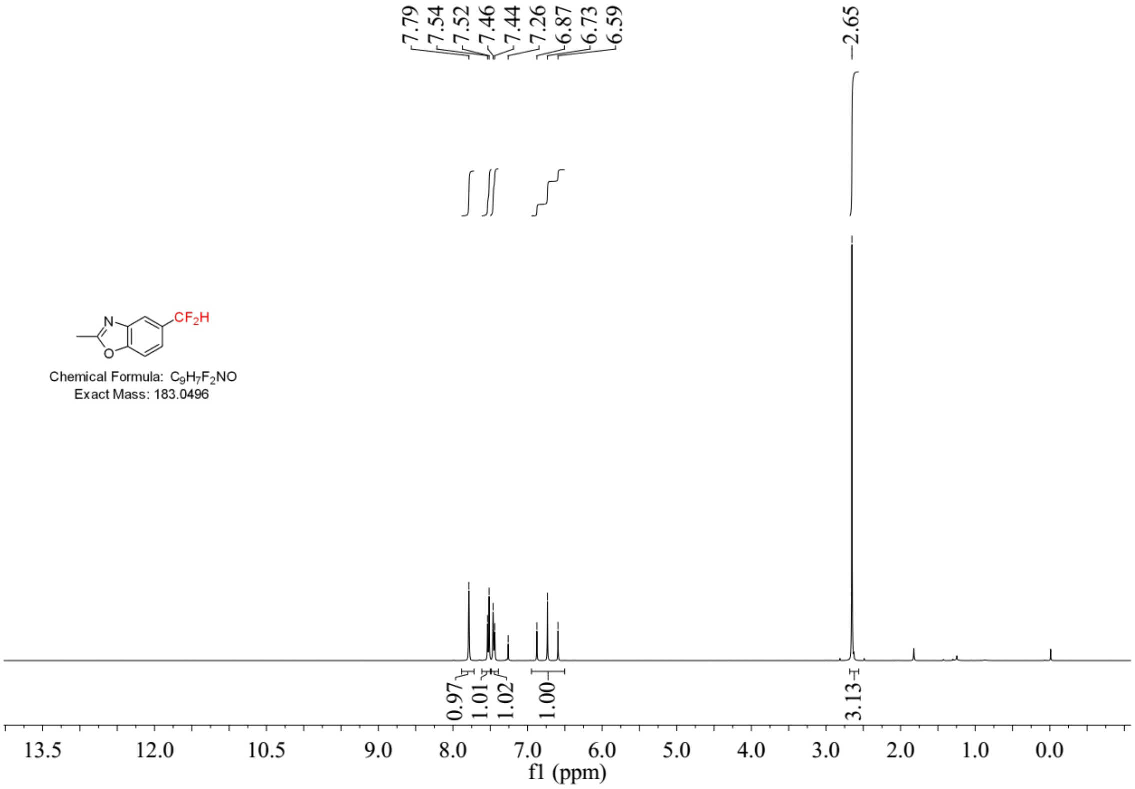

Supplementary Figure 93. ^{1}**H-NMR of 5-(difluoromethyl)-2-methylbenzo[d]oxazole (5i)**

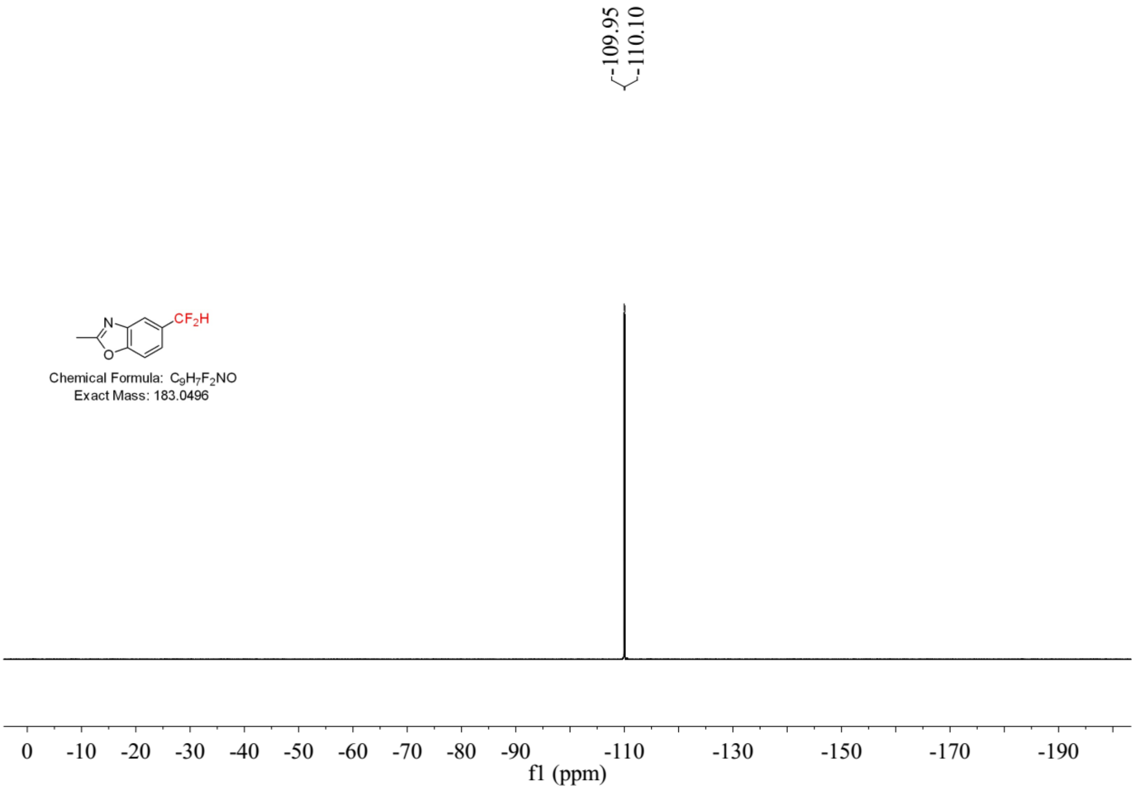

Supplementary Figure 94. ^{19}**F-NMR of 5-(difluoromethyl)-2-methylbenzo[d]oxazole (5i)**

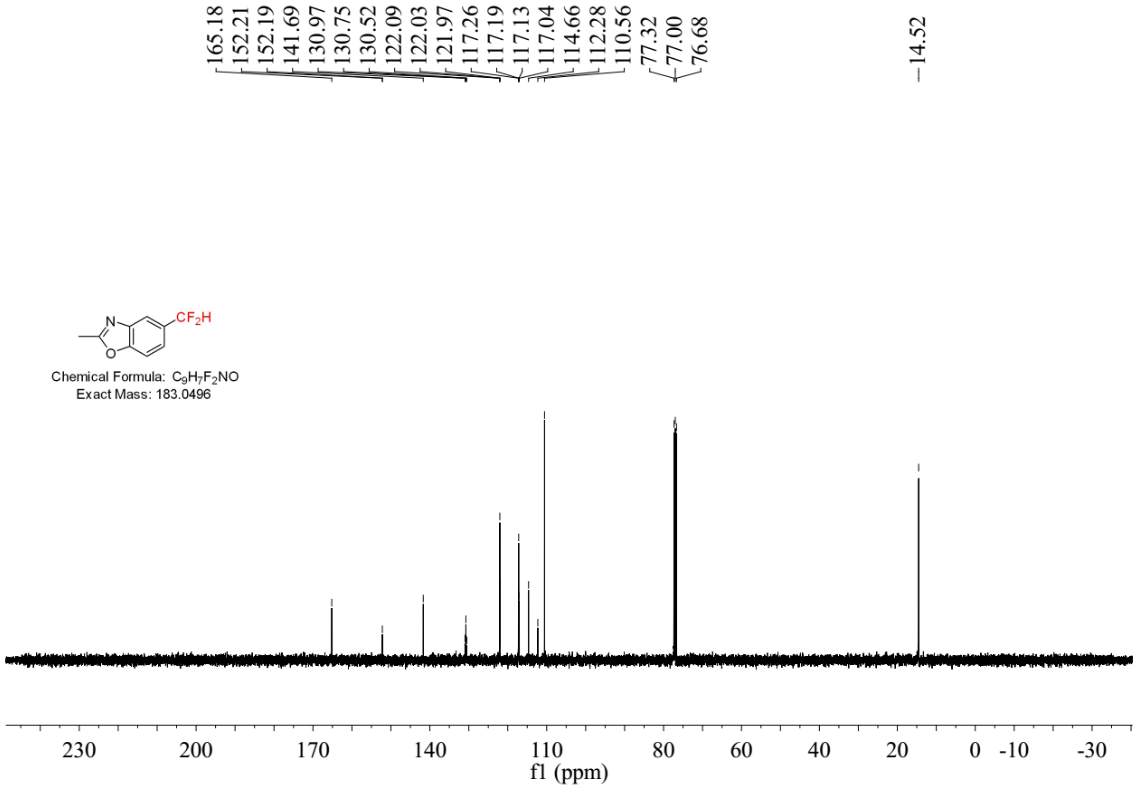

Supplementary Figure 95. ^{13}C-NMR of 5-(difluoromethyl)-2-methylbenzo[d]oxazole (5i)

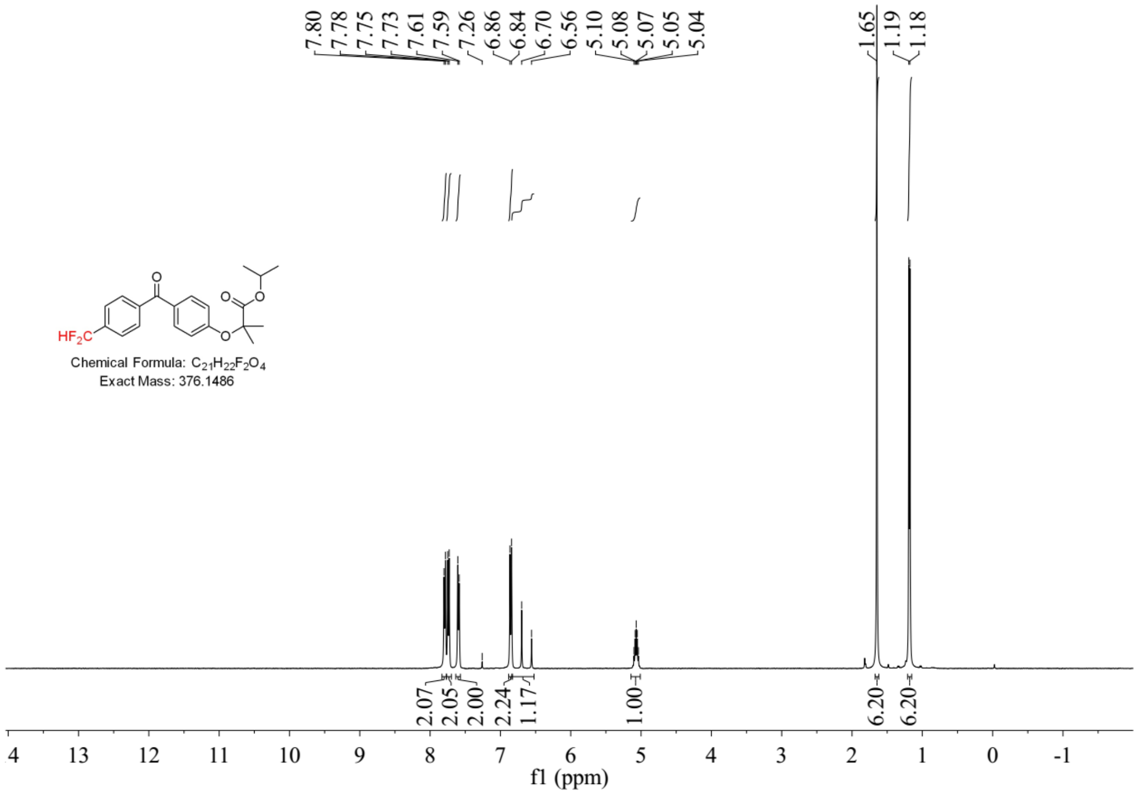

Supplementary Figure 96. ^{1}H-NMR of isopropyl 2-(4-(4-(difluoromethyl)benzoyl)phenoxy)-2-methylpropanoate (7a)

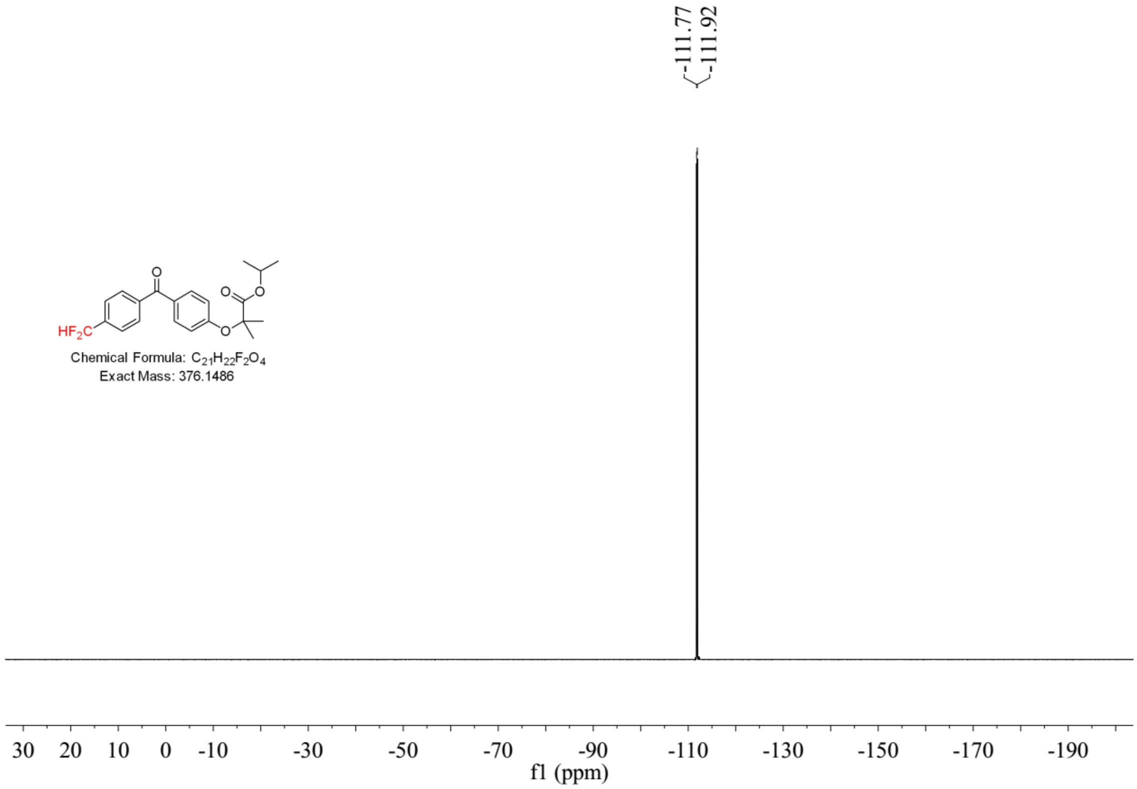

Supplementary Figure 97. ^{19}F-NMR of isopropyl 2-(4-(4-(difluoromethyl)benzoyl)phenoxy)-2-methylpropanoate (7a)

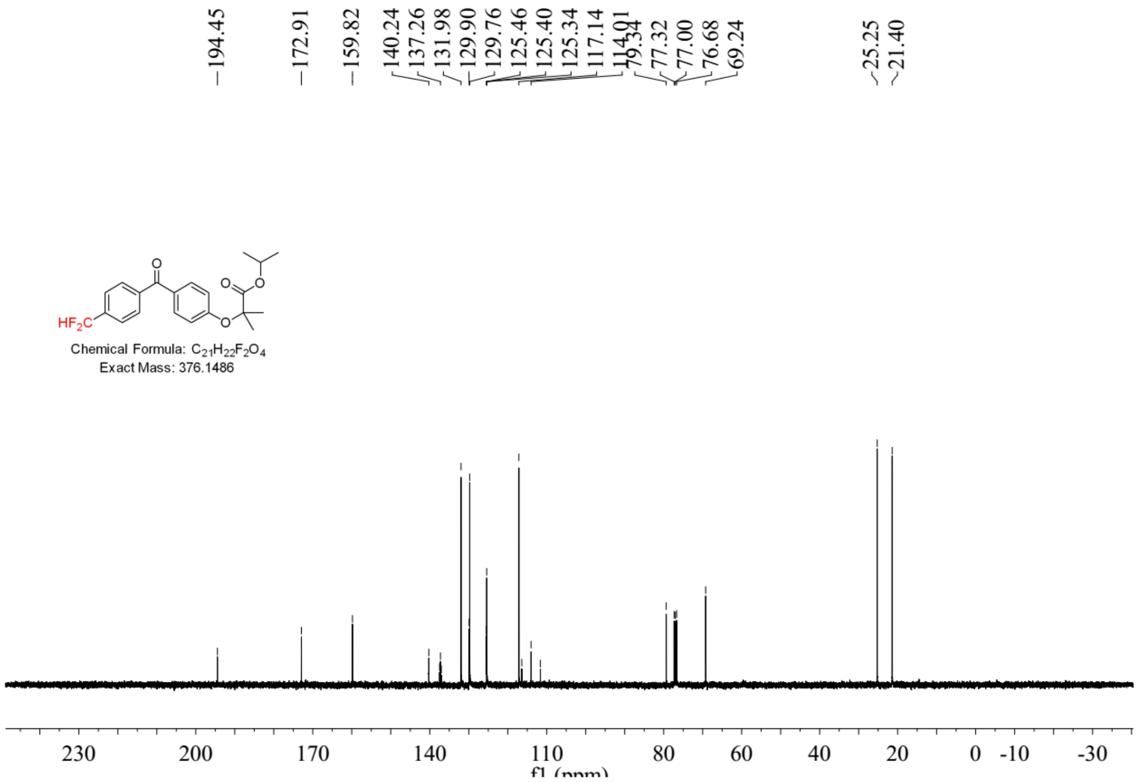

Supplementary Figure 98. ^{13}C-NMR of isopropyl 2-(4-(4-(difluoromethyl)benzoyl)phenoxy)-2-methylpropanoate (7a)

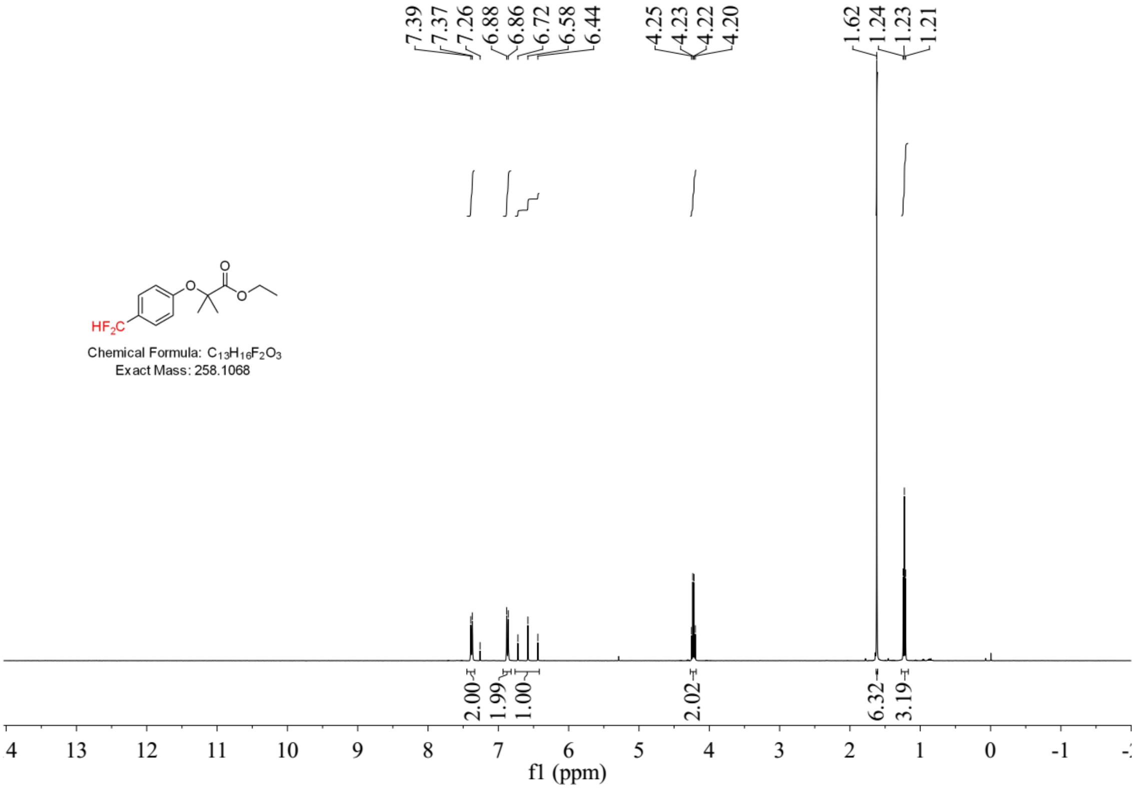

Supplementary Figure 99. ^{1}H-NMR of ethyl 2-(4-(difluoromethyl)phenoxy)-2-methylpropanoate (7b)

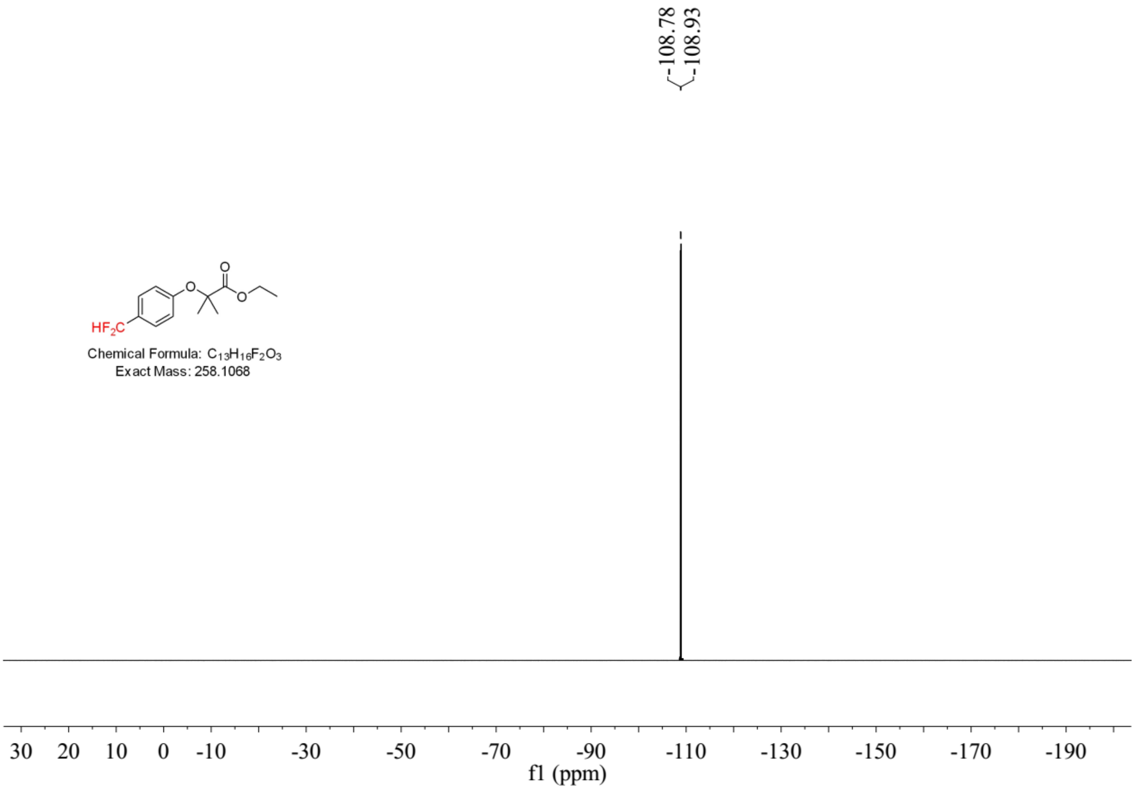

Supplementary Figure 100. ^{19}F-NMR of ethyl 2-(4-(difluoromethyl)phenoxy)-2-methylpropanoate (7b)

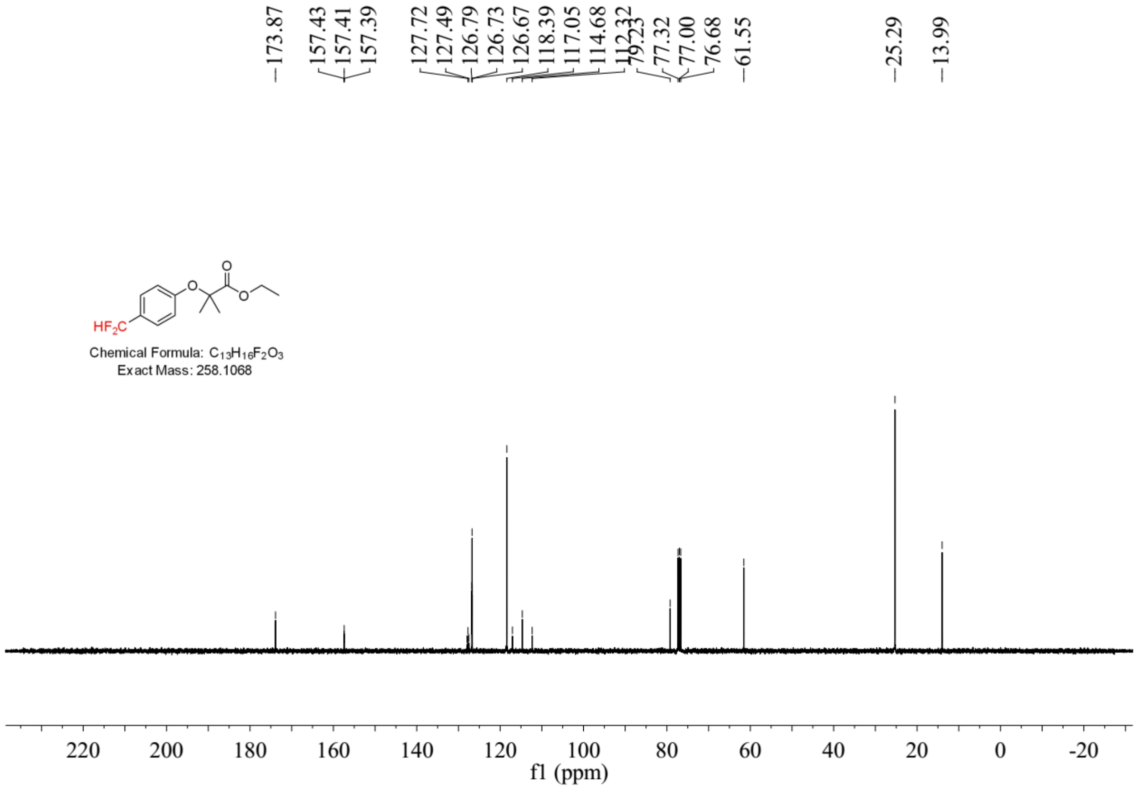

Supplementary Figure 101. ^{13}C-NMR of ethyl 2-(4-(difluoromethyl)phenoxy)-2-methylpropanoate (7b)

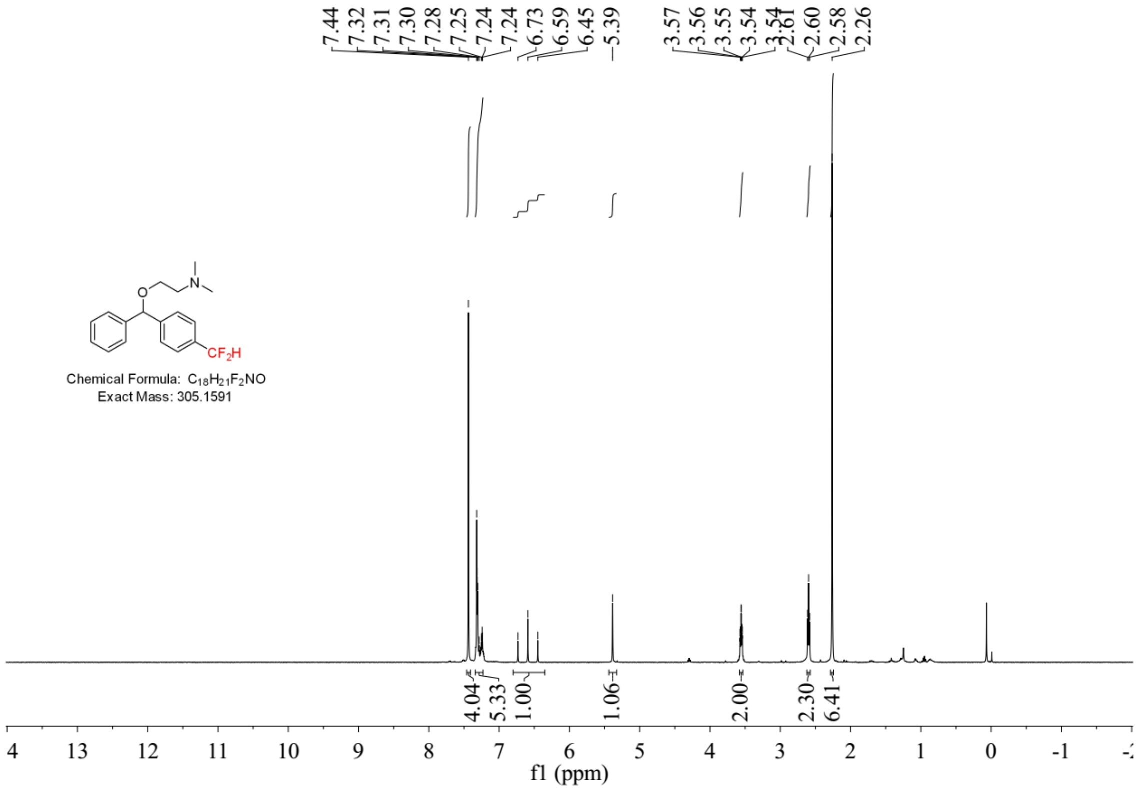

Supplementary Figure 102. ^{1}H-NMR of 2-((4-(difluoromethyl)phenyl)(phenyl)methoxy)-*N*,*N*-dimethylethan-1-amine (7c)

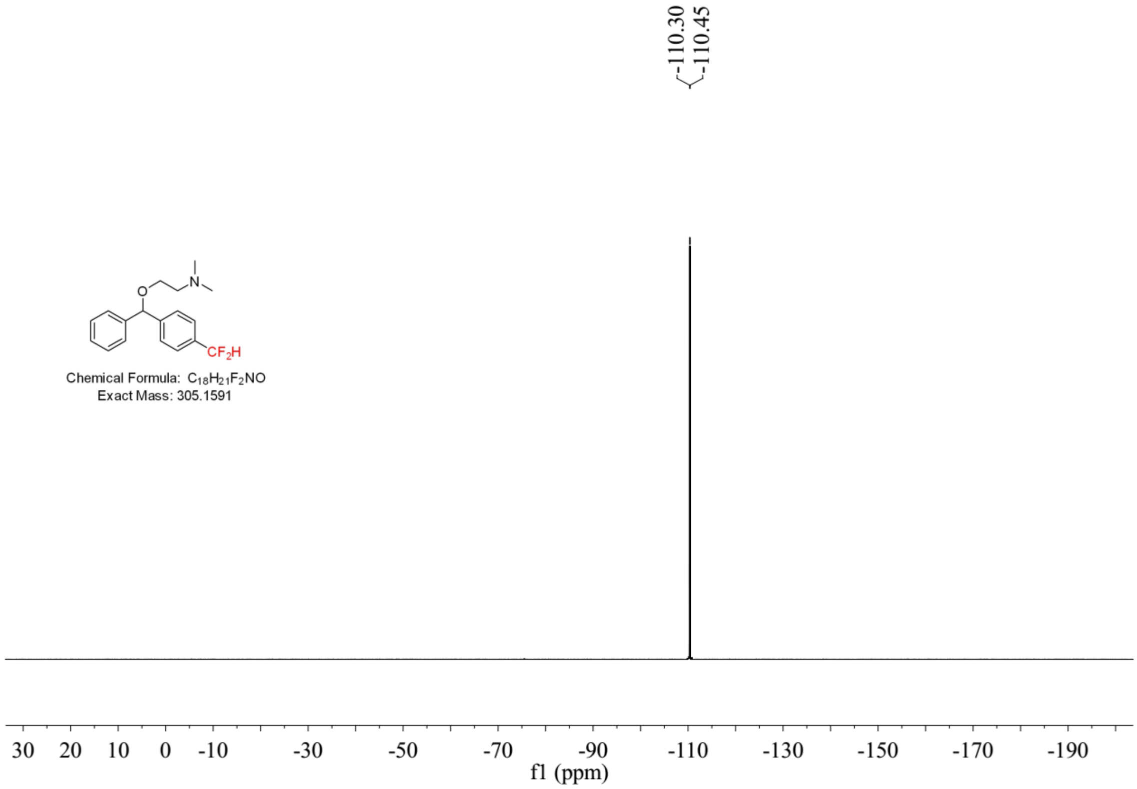

Supplementary Figure 103. ^{19}**F-NMR of 2-((4-(difluoromethyl)phenyl)(phenyl)methoxy)-*N*,*N*-dimethylethan-1-amine (7c)**

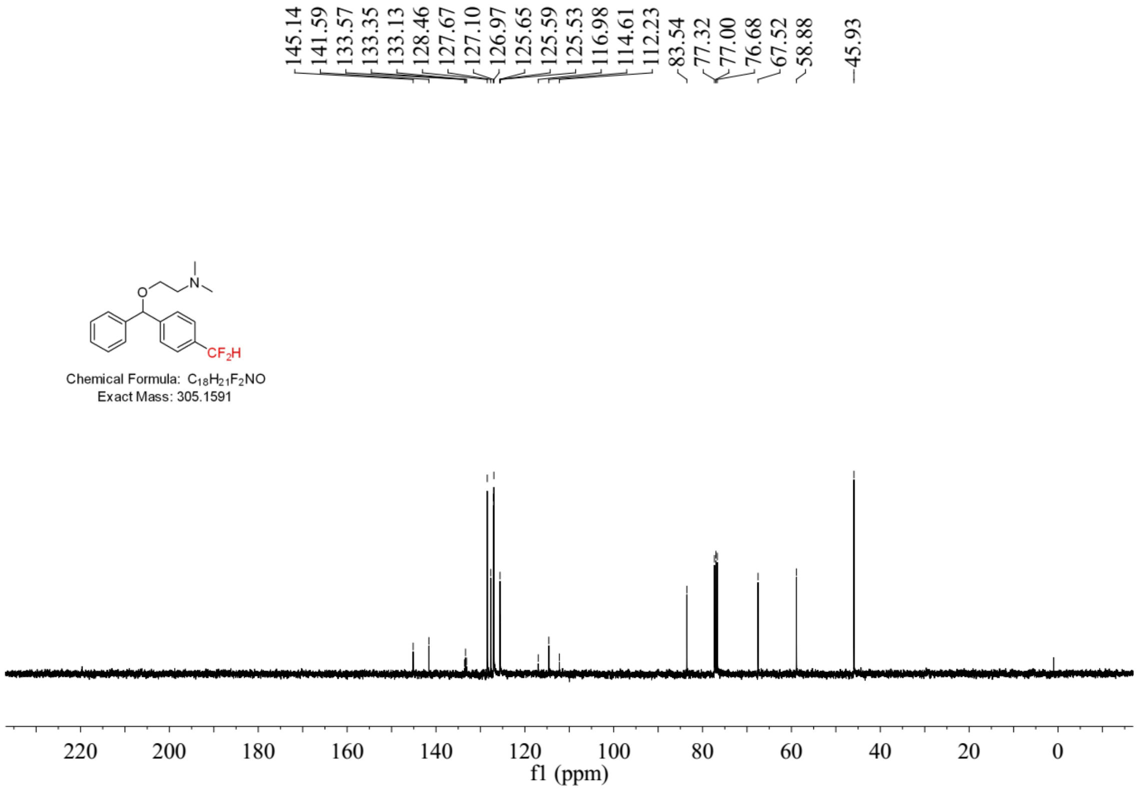

Supplementary Figure 104. ^{13}**C-NMR of 2-((4-(difluoromethyl)phenyl)(phenyl)methoxy)-*N*,*N*-dimethylethan-1-amine (7c)**

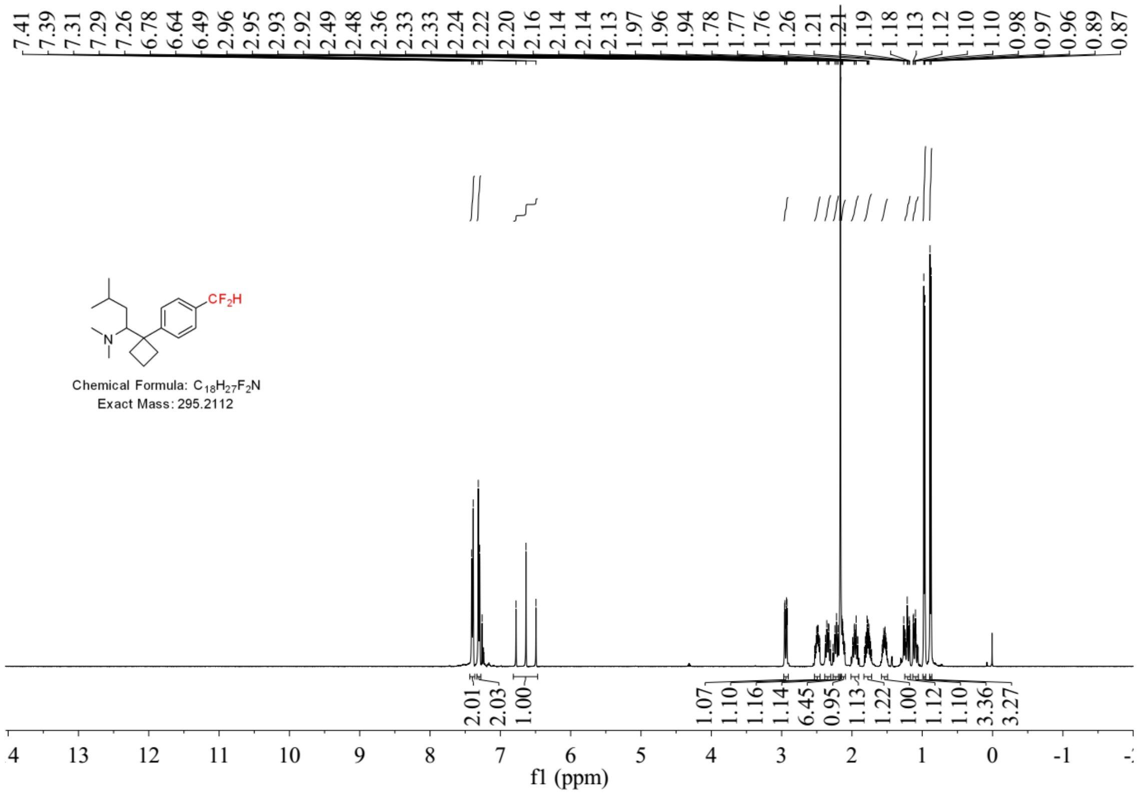

Supplementary Figure 105. ^{1}H-NMR of 1-(1-(4-(difluoromethyl)phenyl)cyclobutyl)-*N*,*N*-3-trimethylbutan-1-amine (7d)

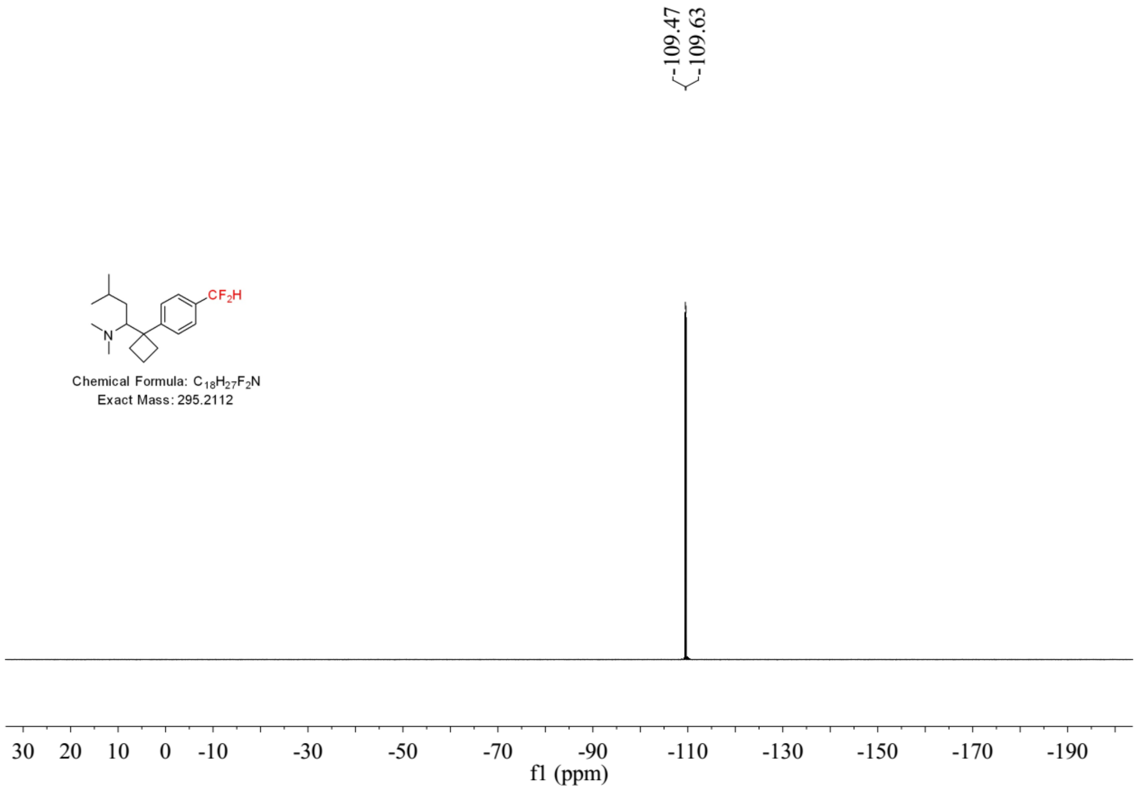

Supplementary Figure 106. ^{19}F-NMR of 1-(1-(4-(difluoromethyl)phenyl)cyclobutyl)-*N*,*N*-3-trimethylbutan-1-amine (7d)

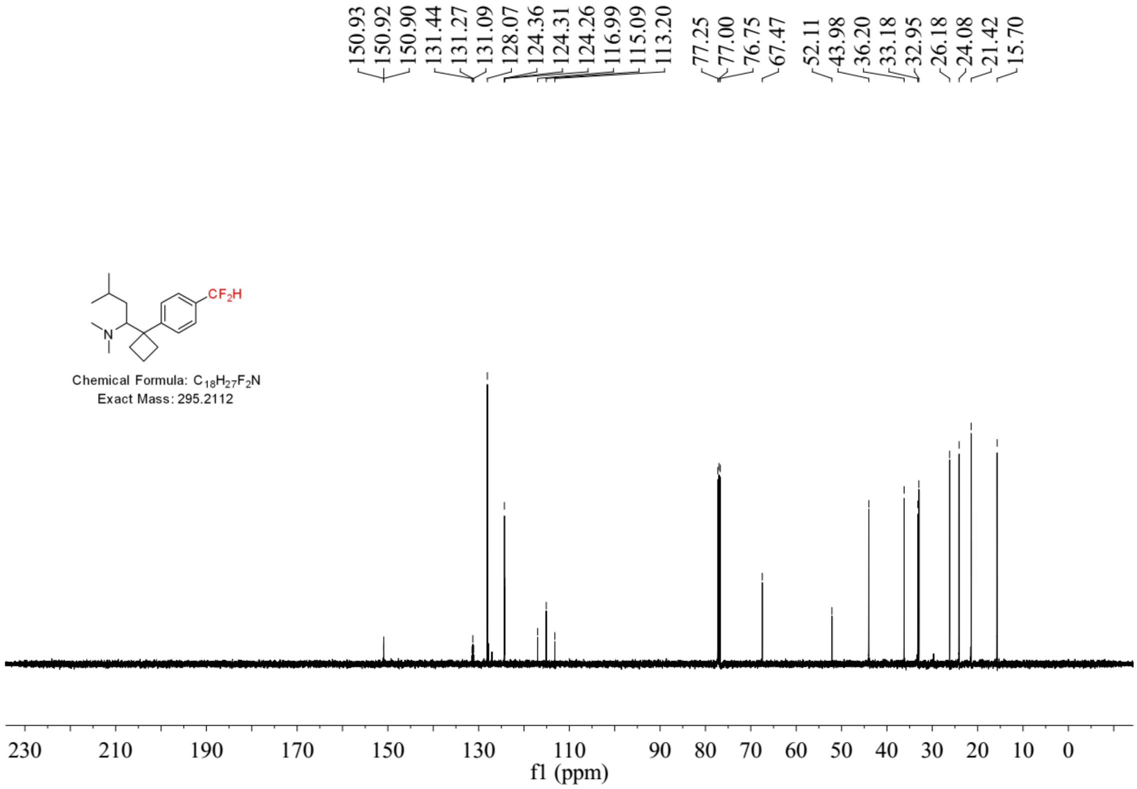

Supplementary Figure 107. ^{13}**C-NMR of 1-(1-(4-(difluoromethyl)phenyl)cyclobutyl)-*N*,*N*-3-trimethylbutan-1-amine (7d)**

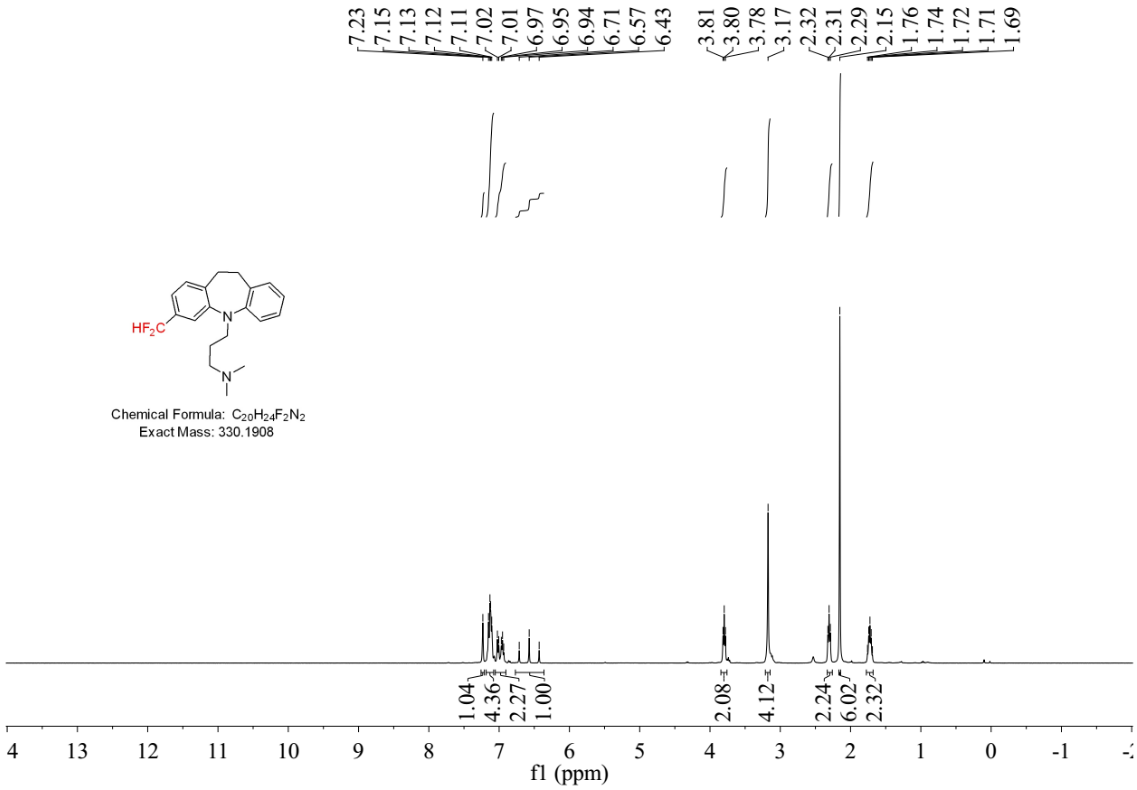

Supplementary Figure 108. ^{1}**H-NMR of product 7e**

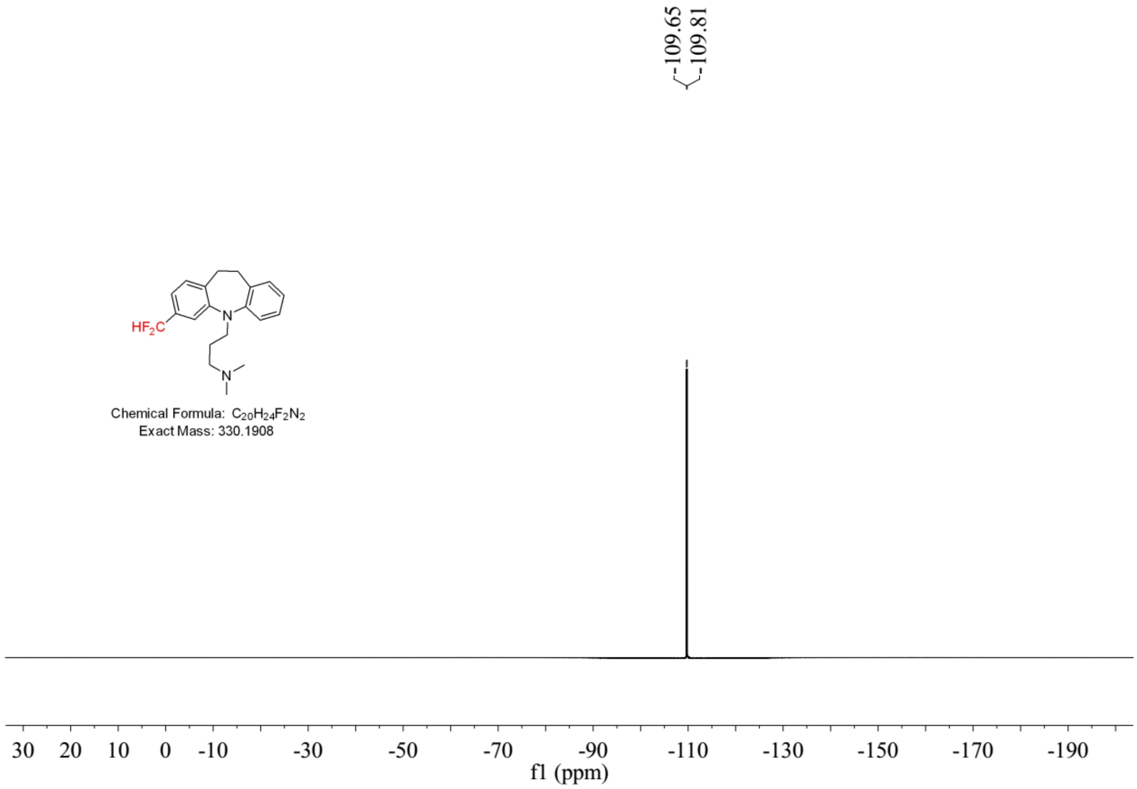

Supplementary Figure 109. ^{19}F-NMR of product 7e

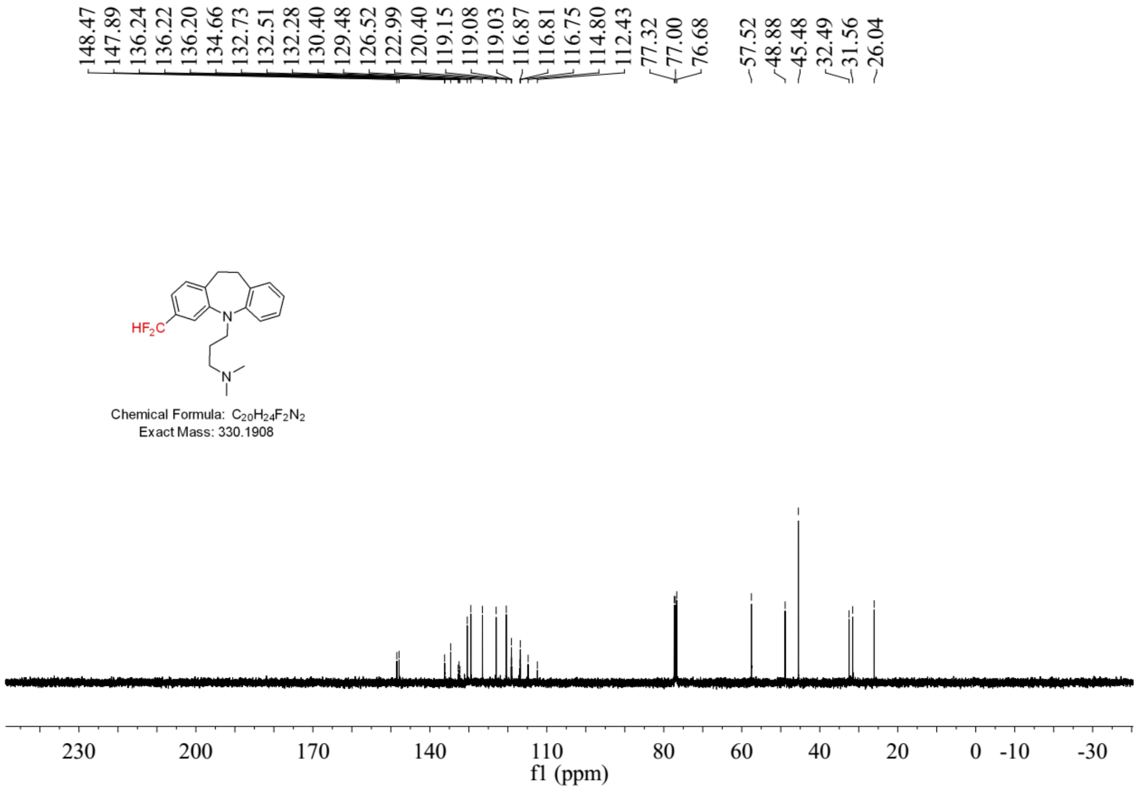

Supplementary Figure 110. ^{13}C-NMR of product 7e

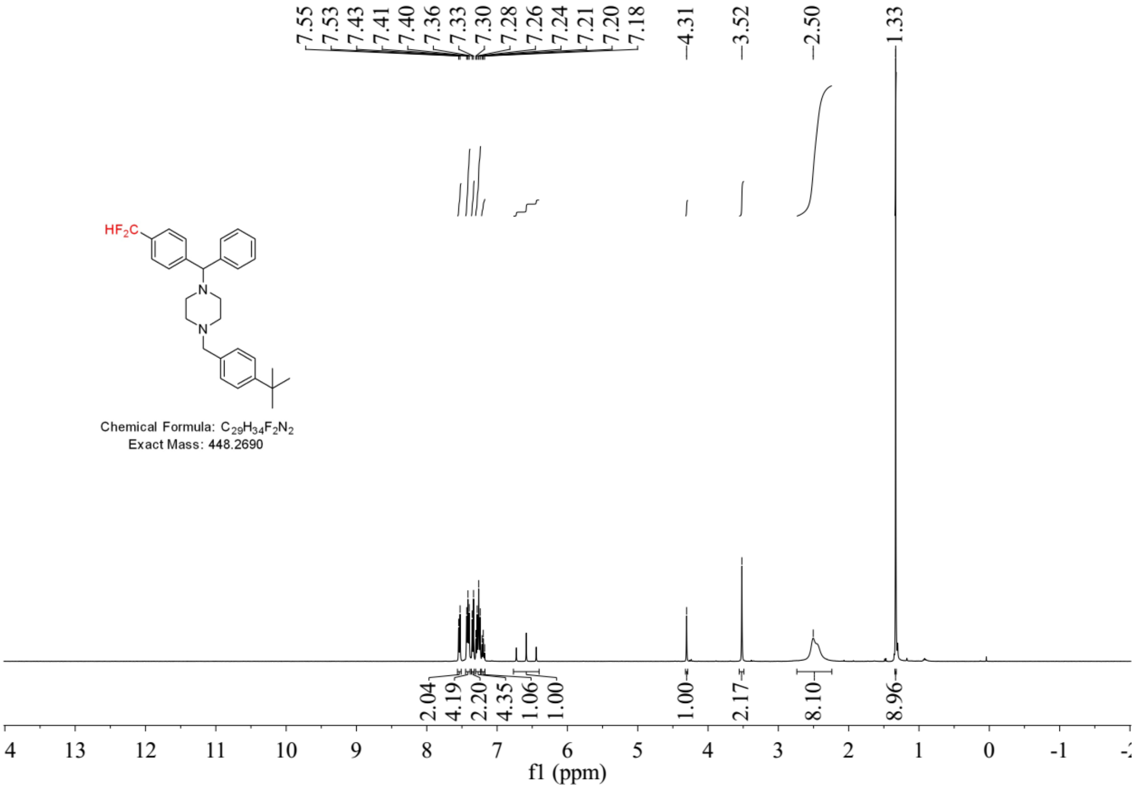

Supplementary Figure 111. **^{1}H-NMR of 1-(4-(*tert*-butyl)benzyl)-4-((4-(difluoromethyl)phenyl)(phenyl)methyl)piperazine (7f)**

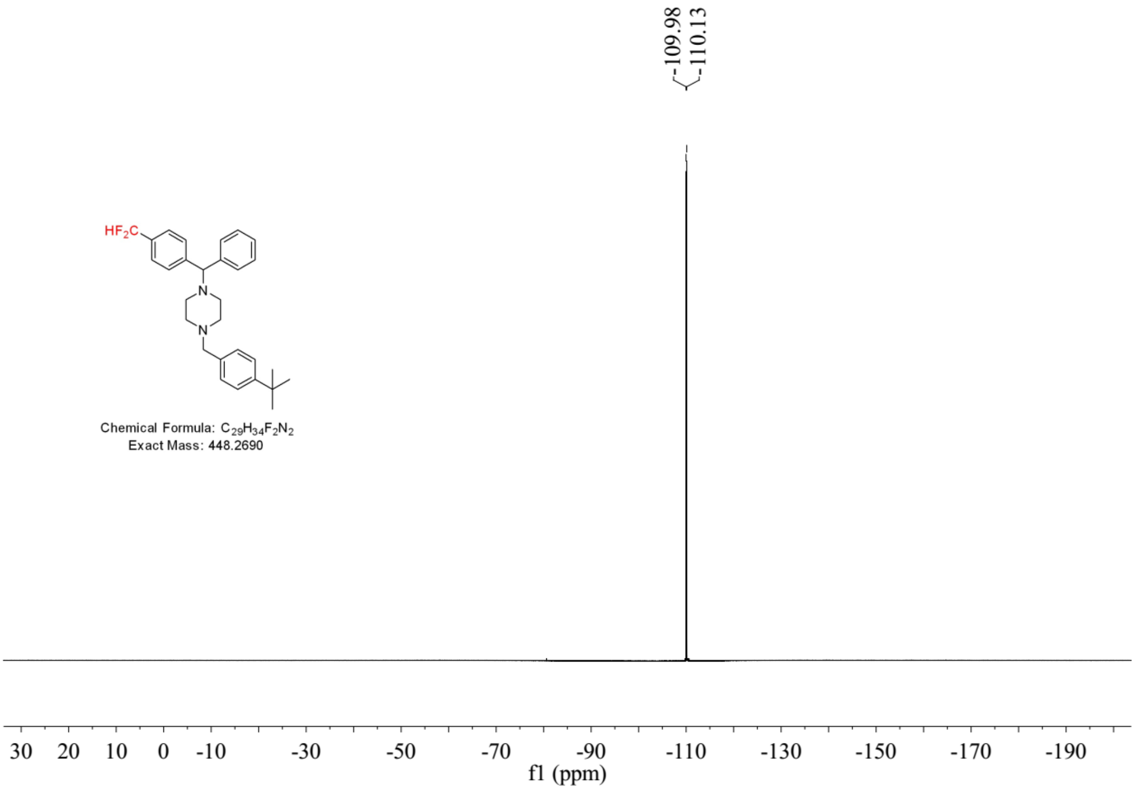

Supplementary Figure 112. **^{19}F-NMR of 1-(4-(*tert*-butyl)benzyl)-4-((4-(difluoromethyl)phenyl)(phenyl)methyl)piperazine (7f)**

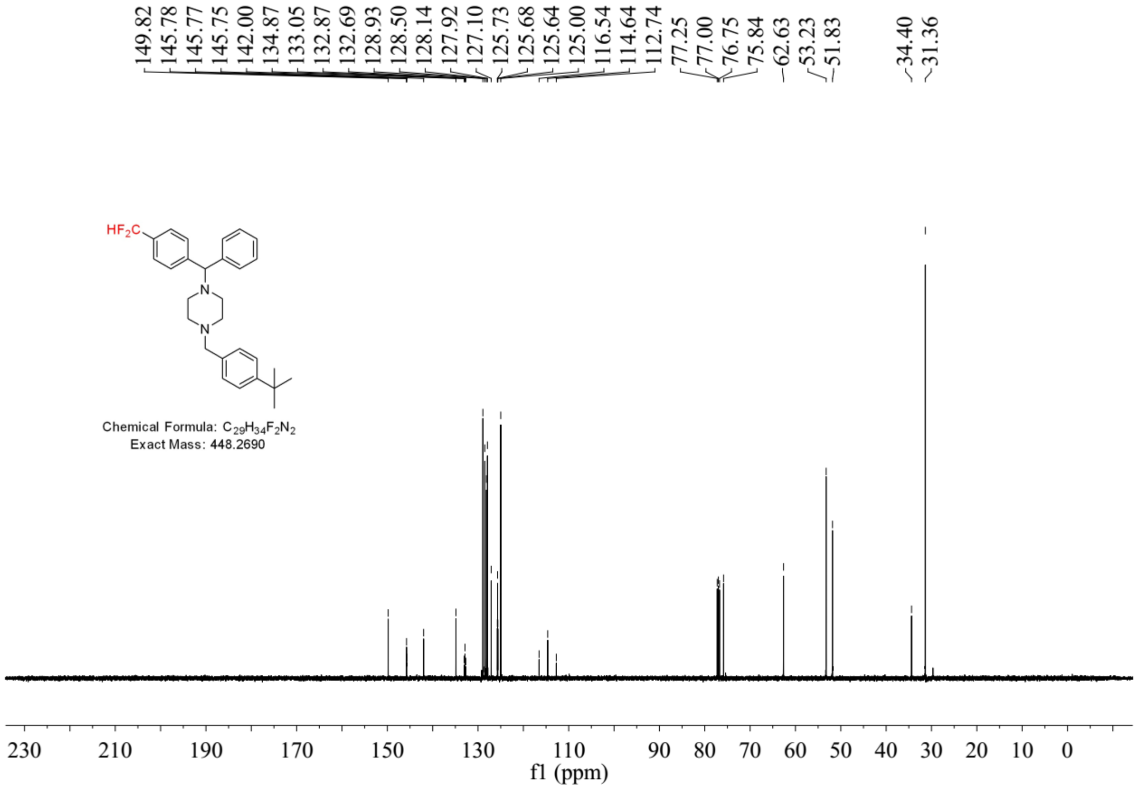

Supplementary Figure 113. ^{13}**C-NMR of 1-(4-(*tert*-butyl)benzyl)-4-((4-(difluoromethyl)phenyl)(phenyl)methyl)piperazine (7f)**

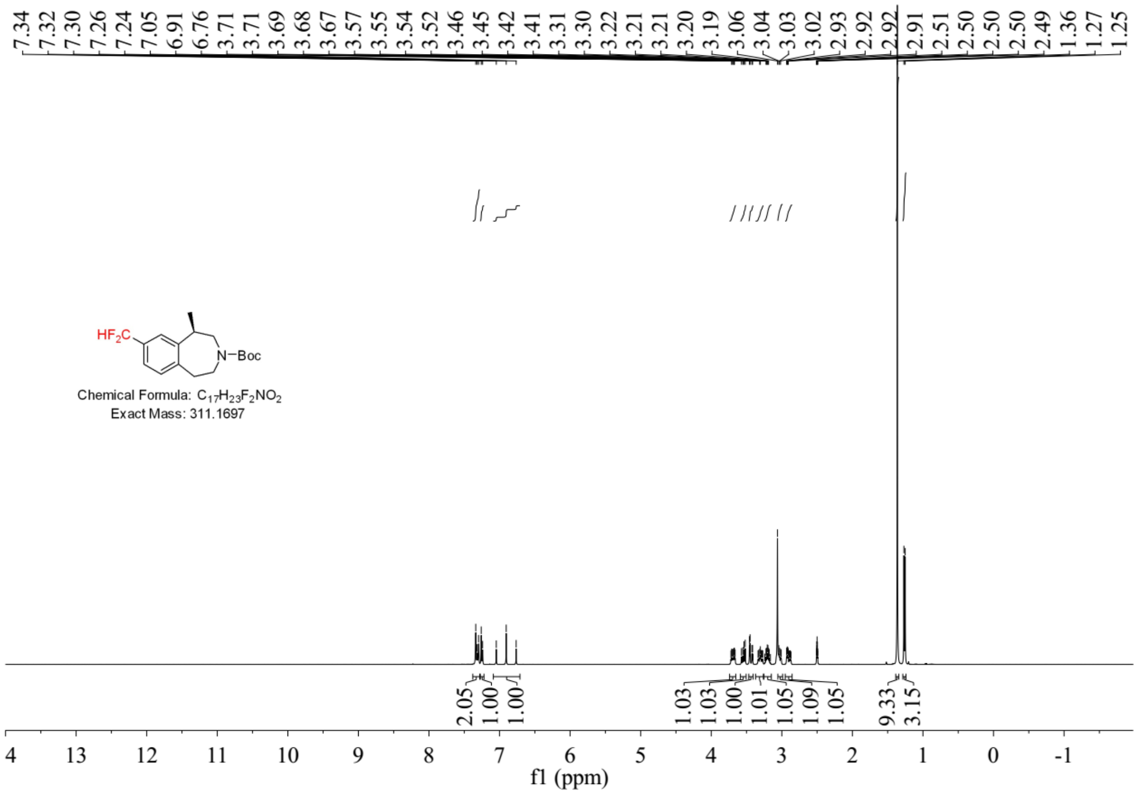

Supplementary Figure 114. ^{1}**H-NMR of product 7g**

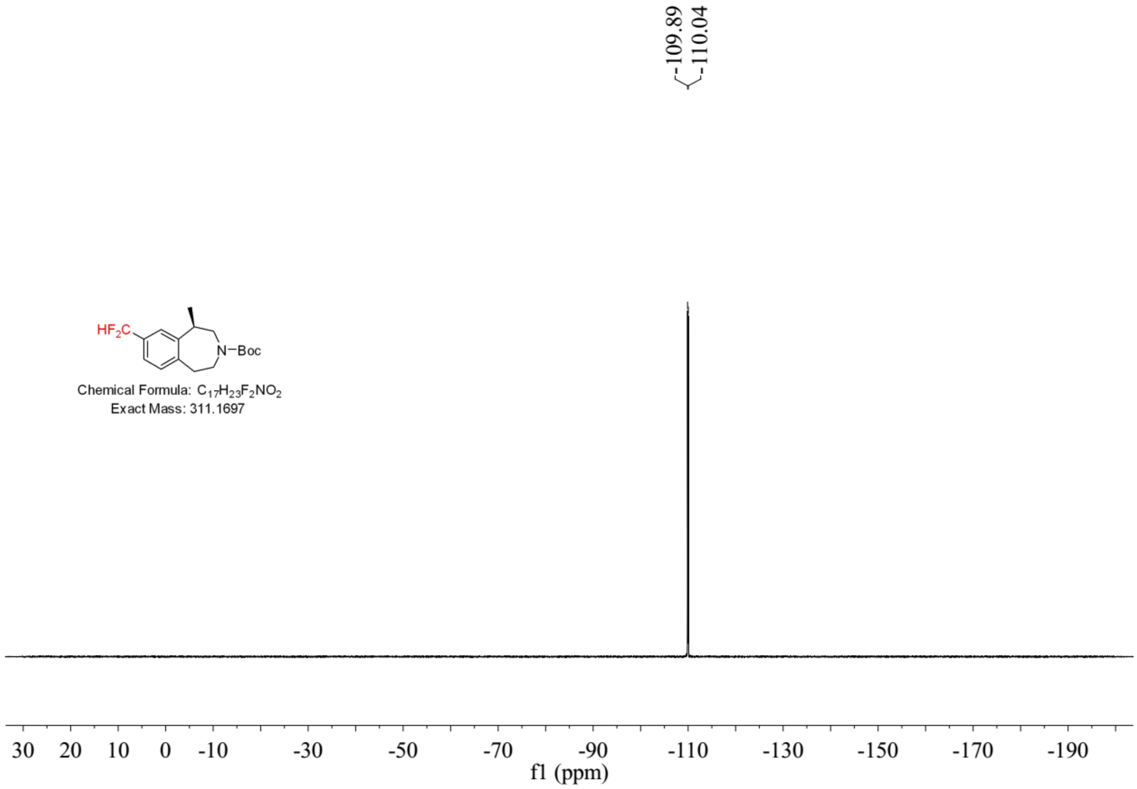

Supplementary Figure 115. ^{19}F-NMR of product 7g

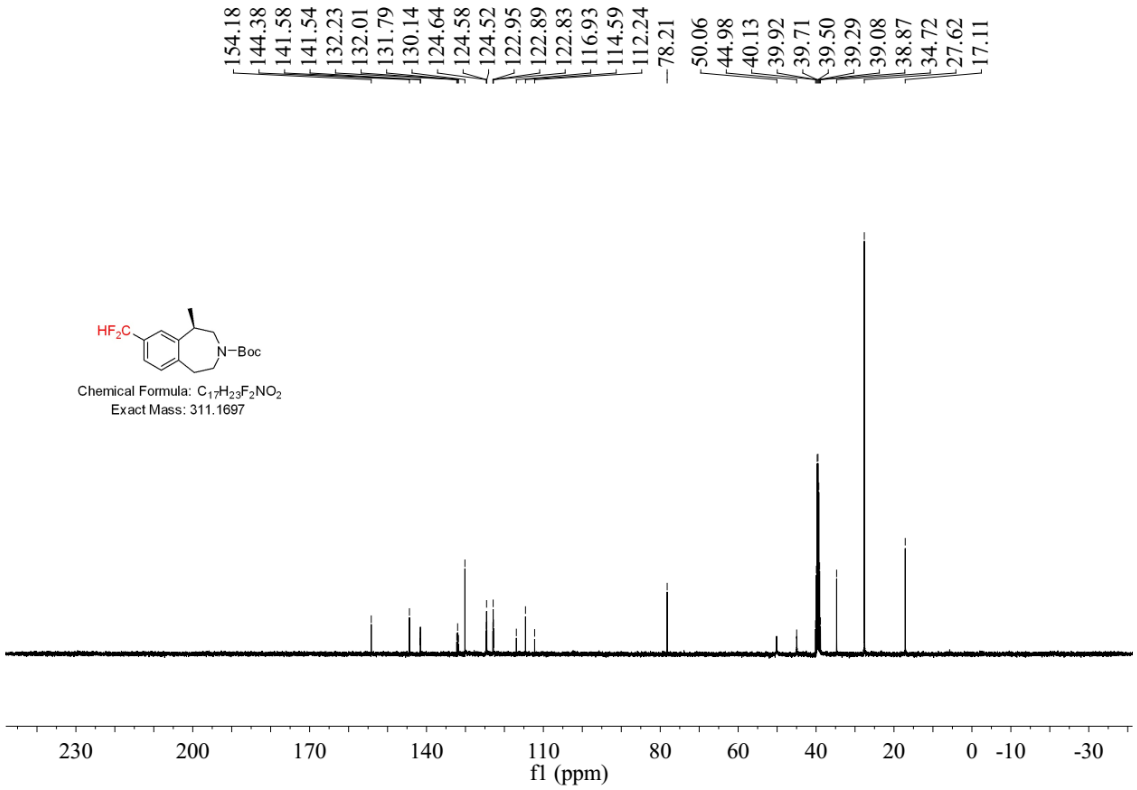

Supplementary Figure 116. ^{13}C-NMR of product 7g

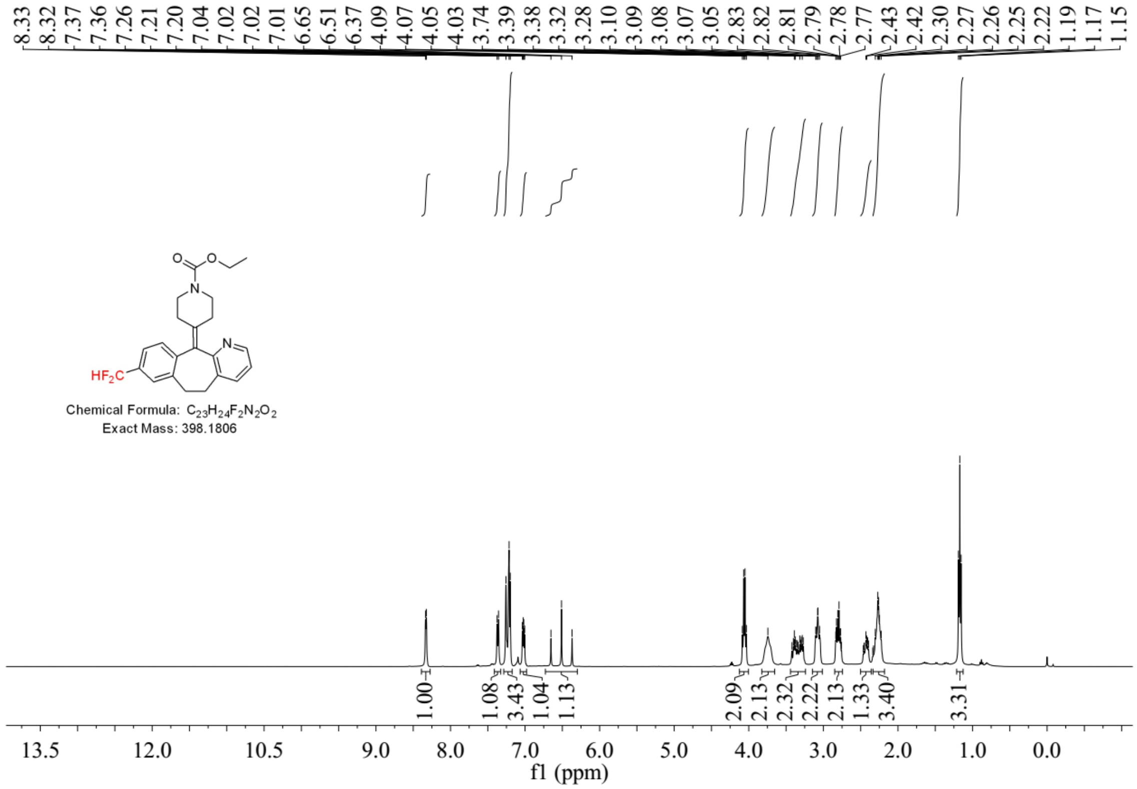

Supplementary Figure 117. ^{1}H-NMR of product 7h

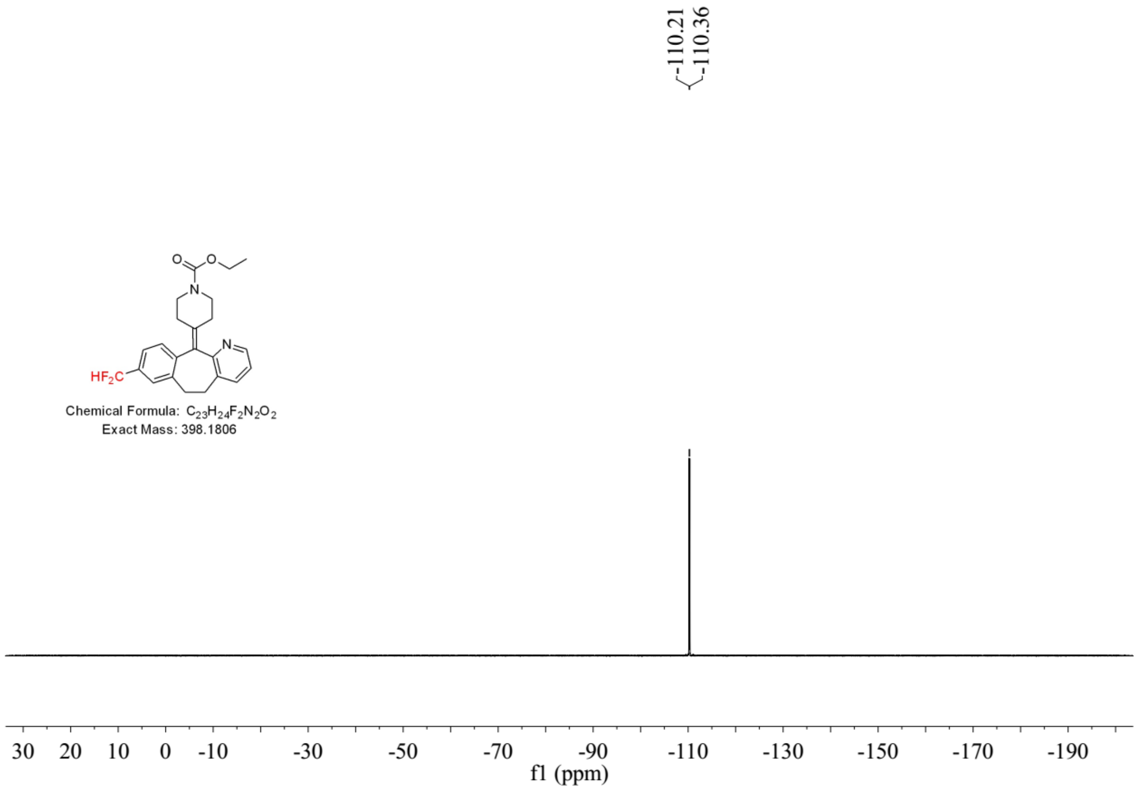

Supplementary Figure 118. ^{19}F-NMR of product 7h

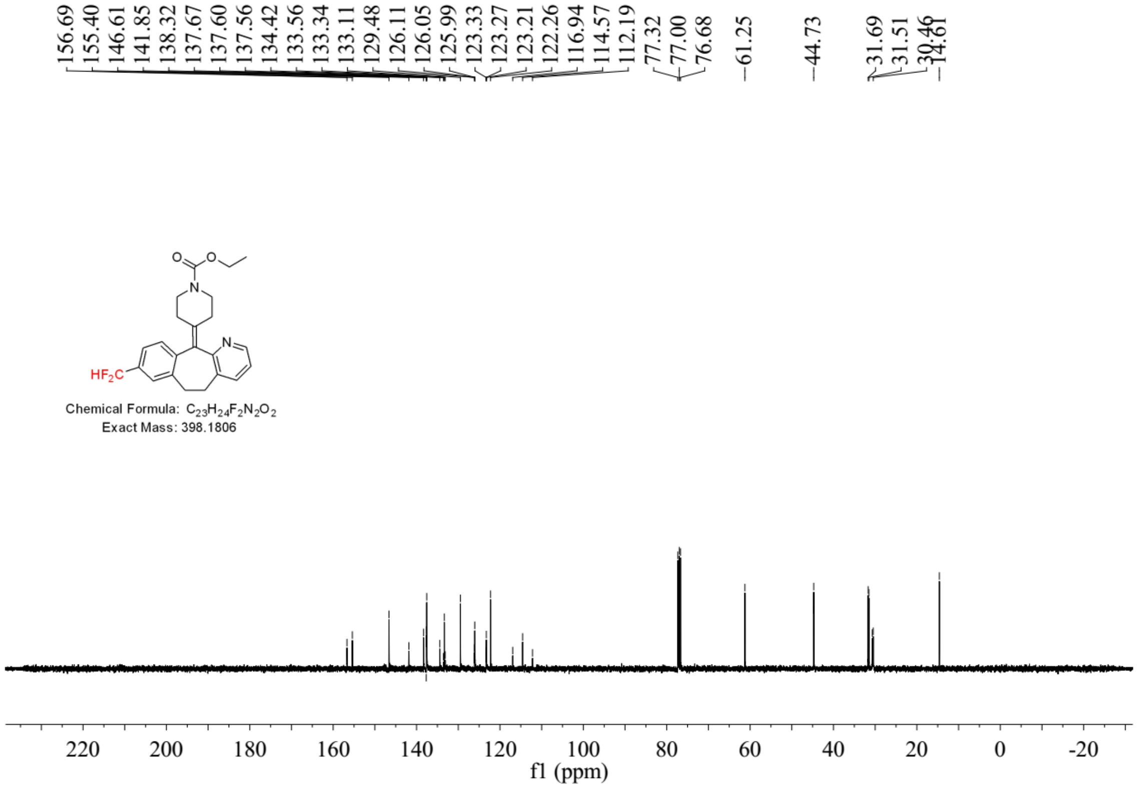

Supplementary Figure 119. ^{13}C-NMR of product 7h

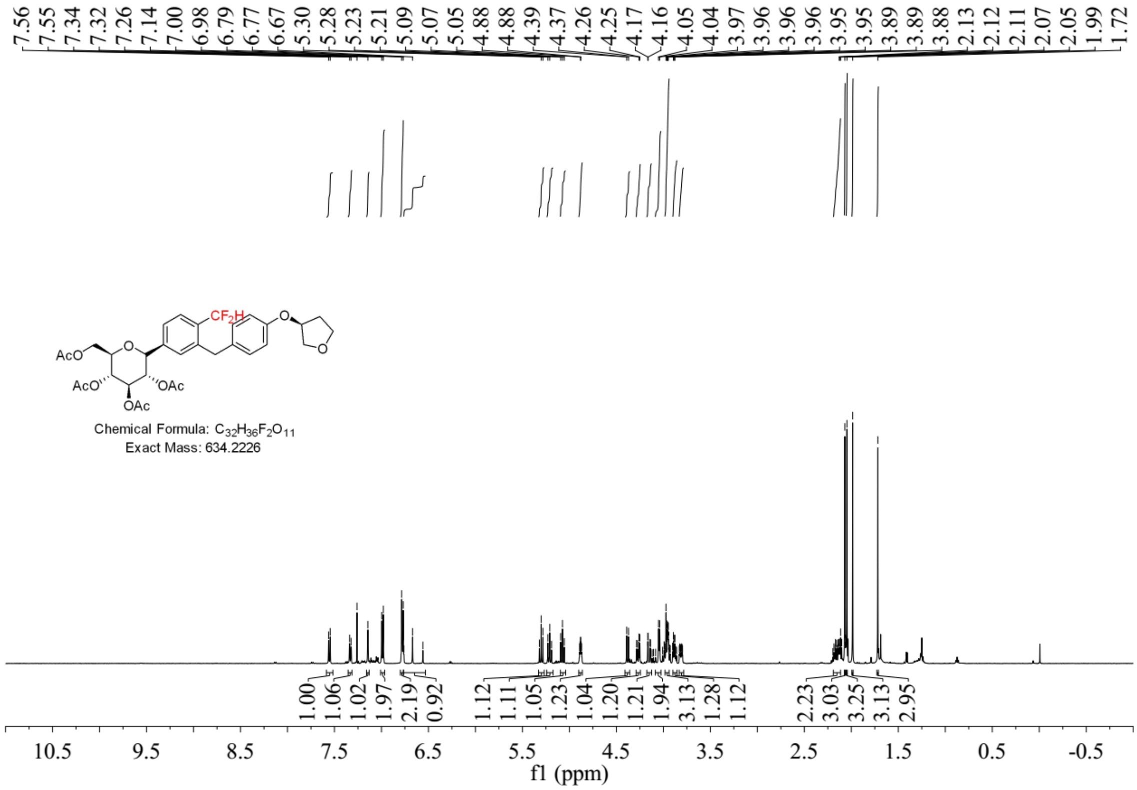

Supplementary Figure 120. ^{1}H-NMR of product 7i

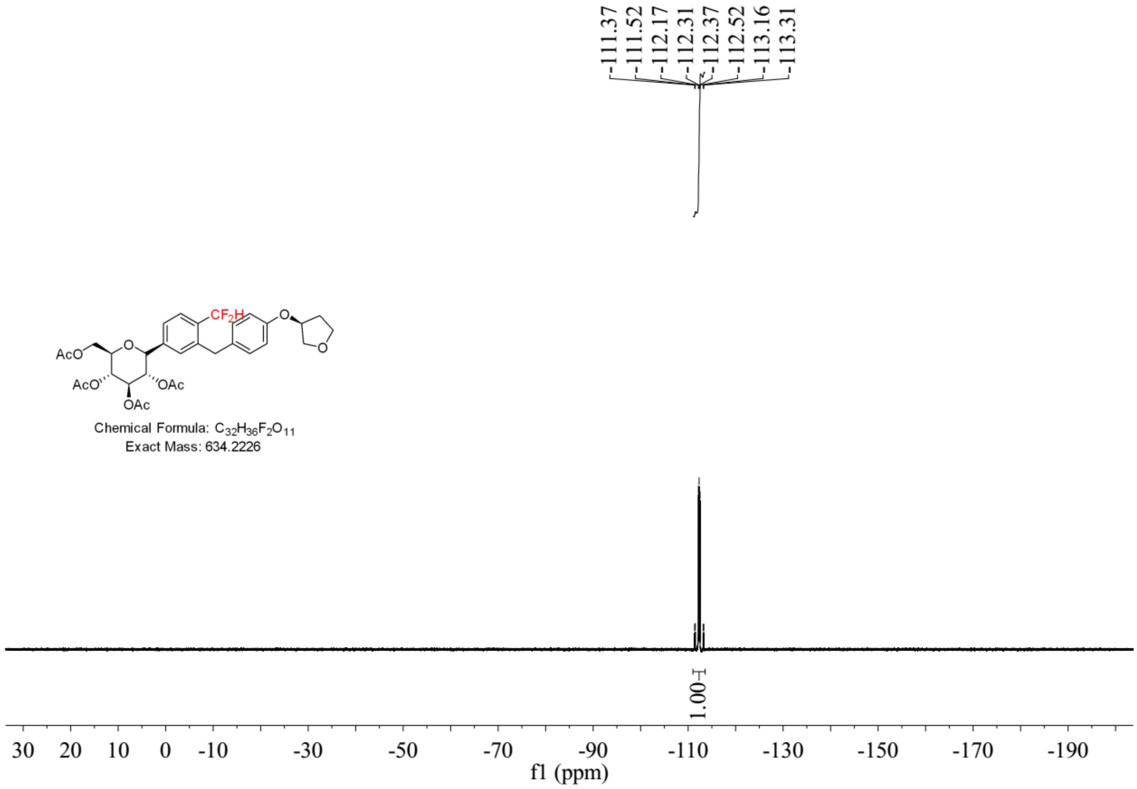

Supplementary Figure 121. ^{19}F-NMR of product 7i

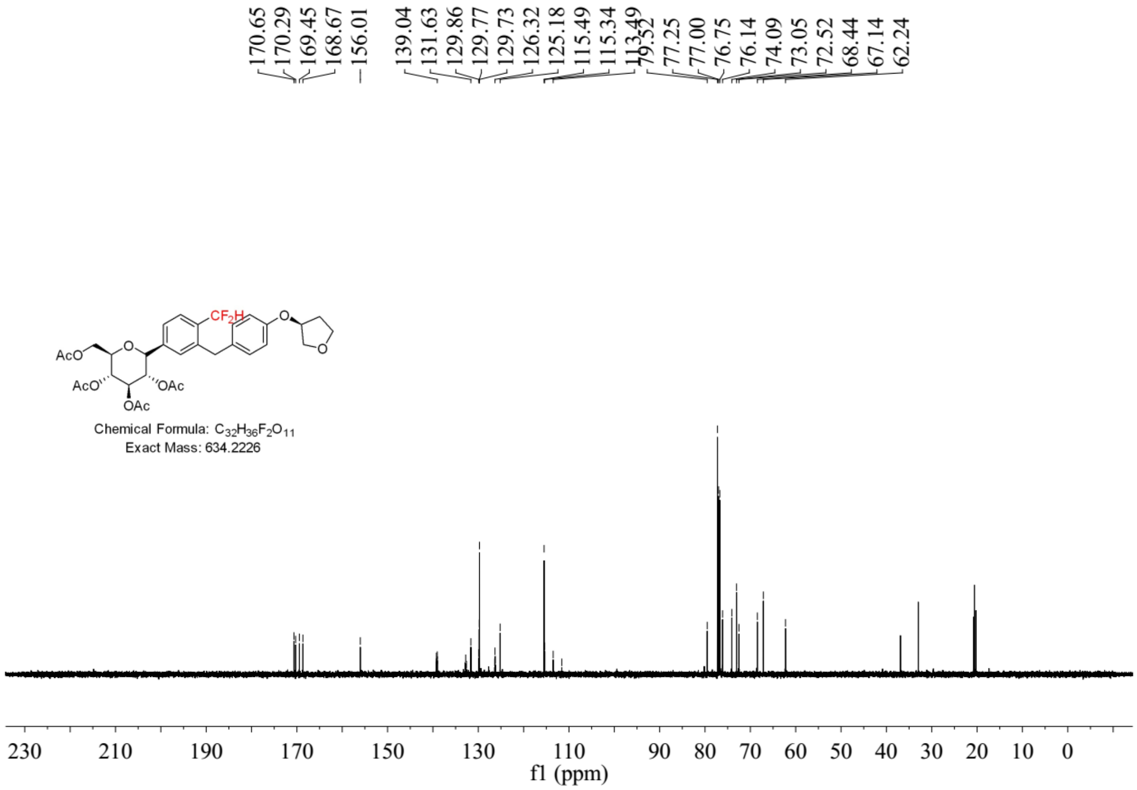

Supplementary Figure 122. ^{13}C-NMR of product 7i

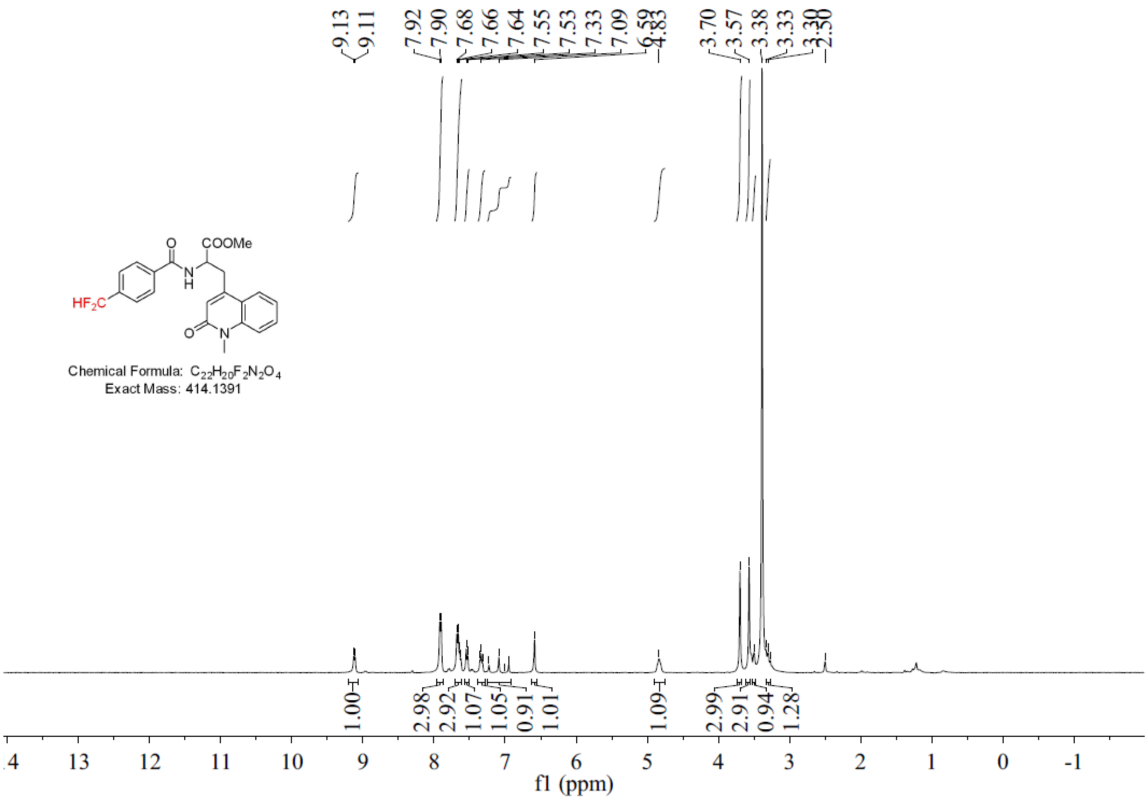

Supplementary Figure 123. ^{1}H-NMR of product 7j

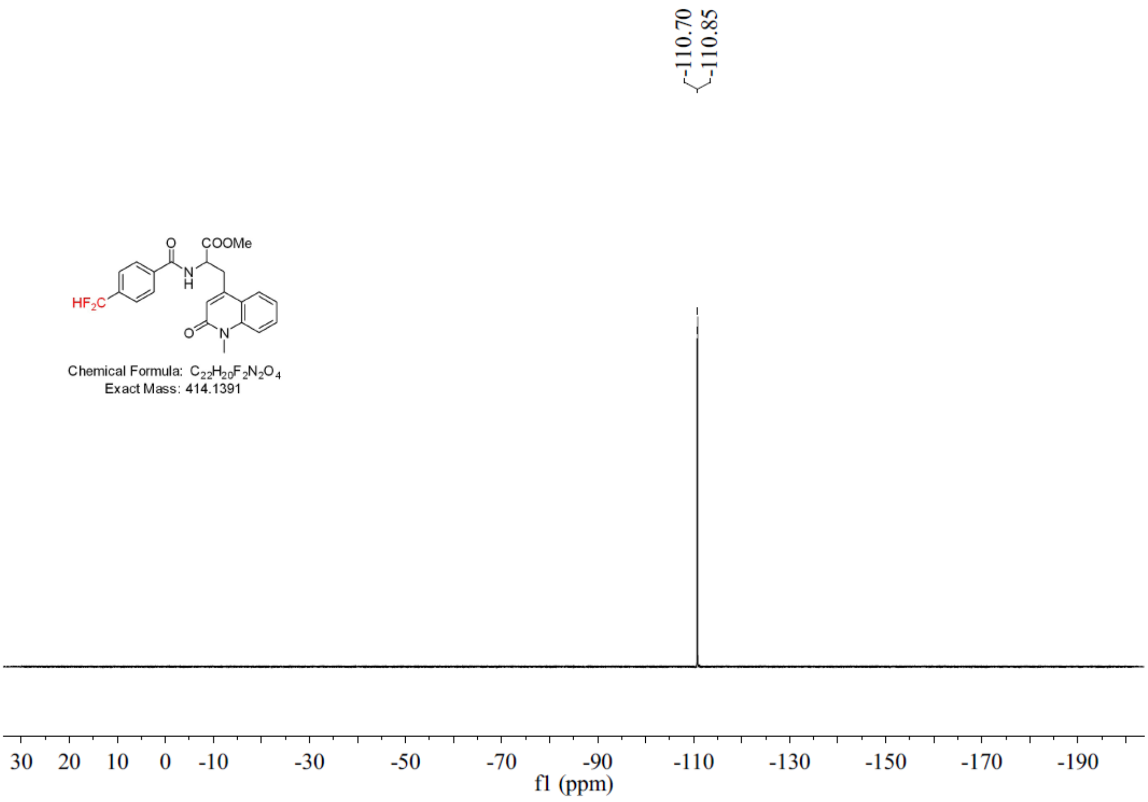

Supplementary Figure 124. ^{19}F-NMR of product 7j

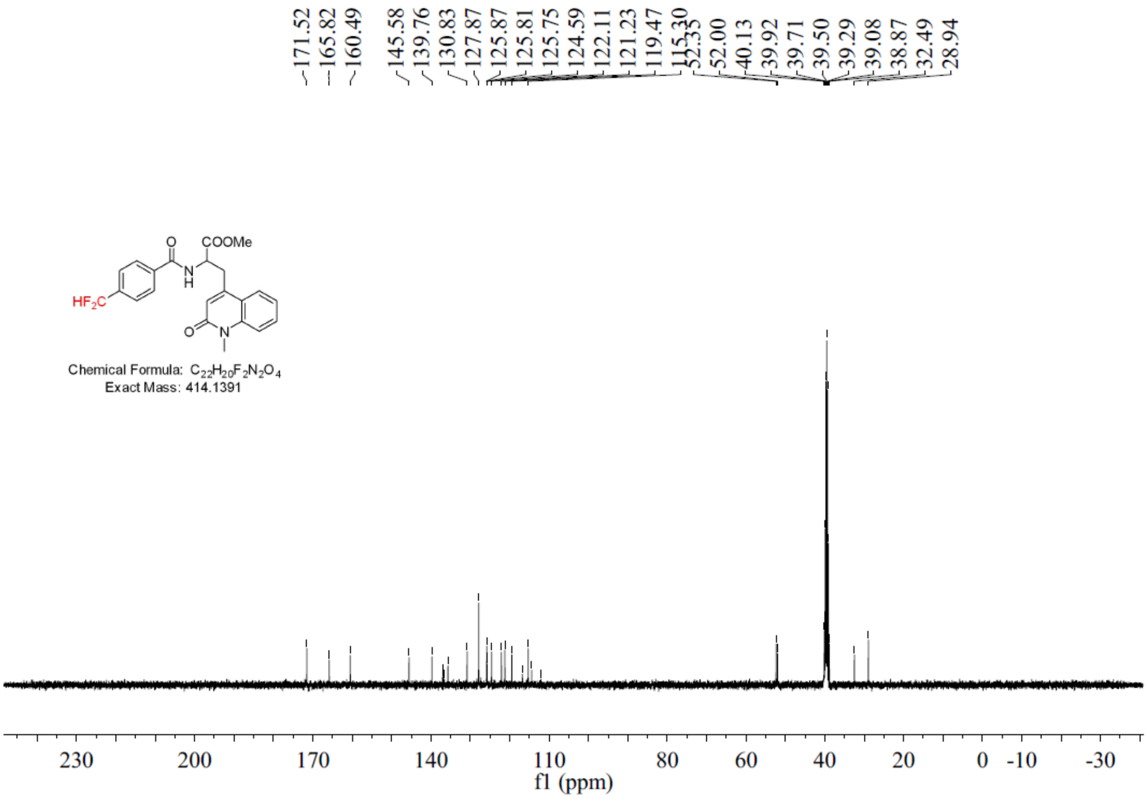

Supplementary Figure 125. ^{13}C-NMR of product 7j

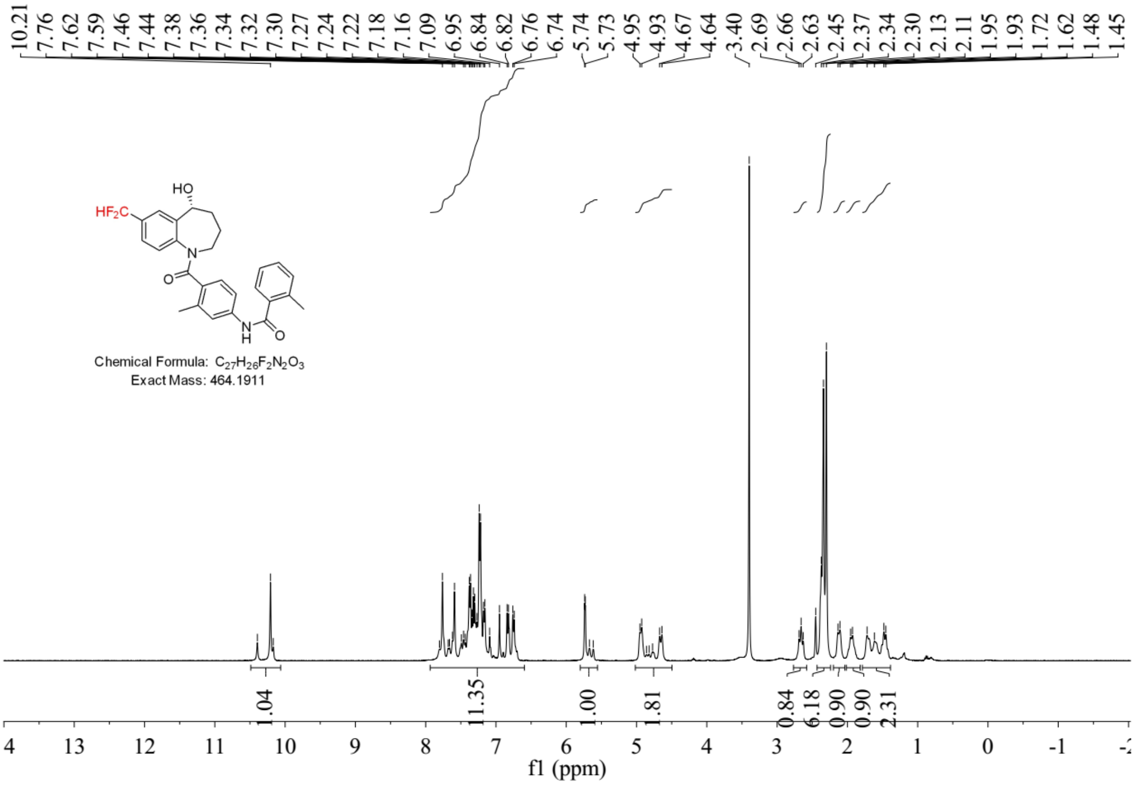

Supplementary Figure 126. ^{1}H-NMR of product 7k

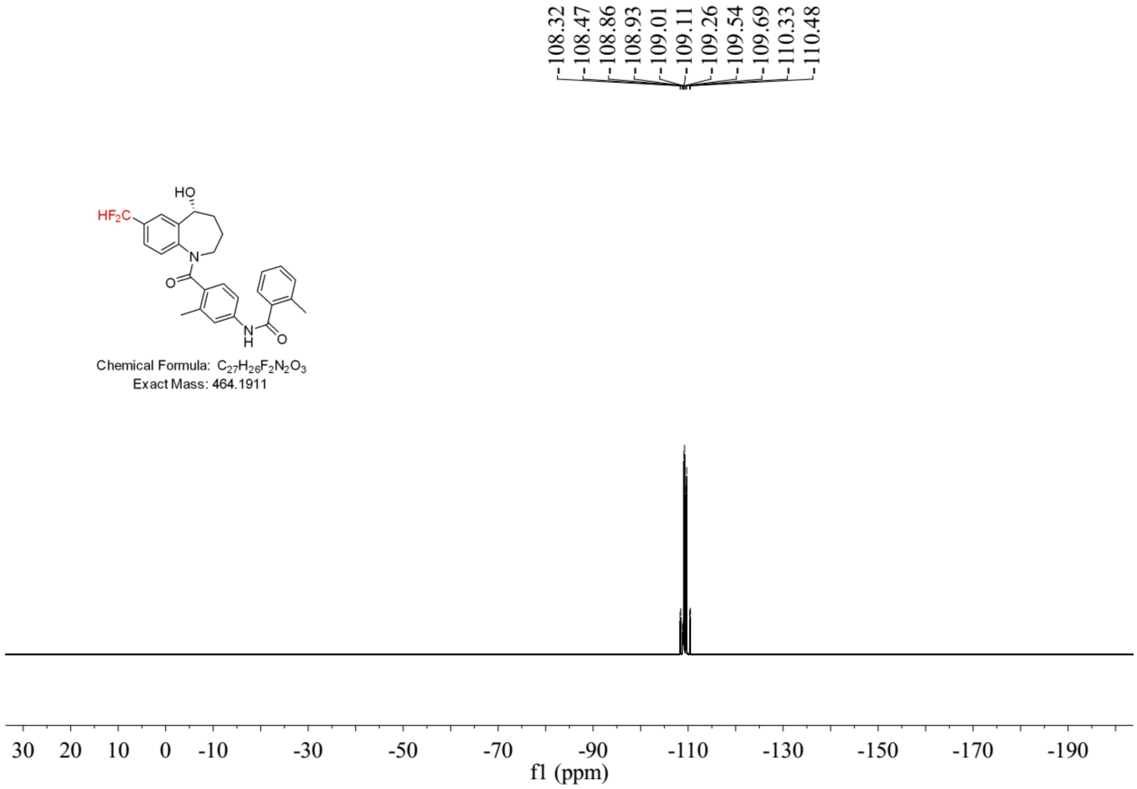

Supplementary Figure 127. ^{19}F-NMR of product 7k

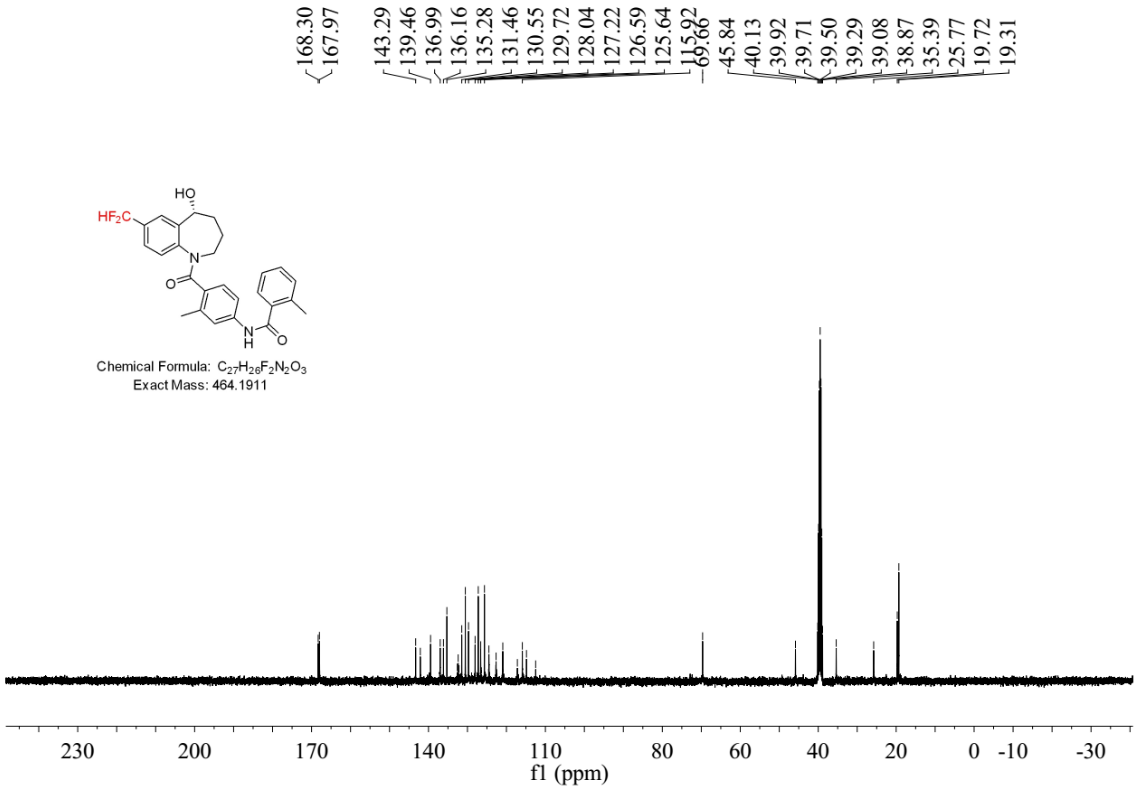

Supplementary Figure 128. ^{13}C-NMR of product 7k

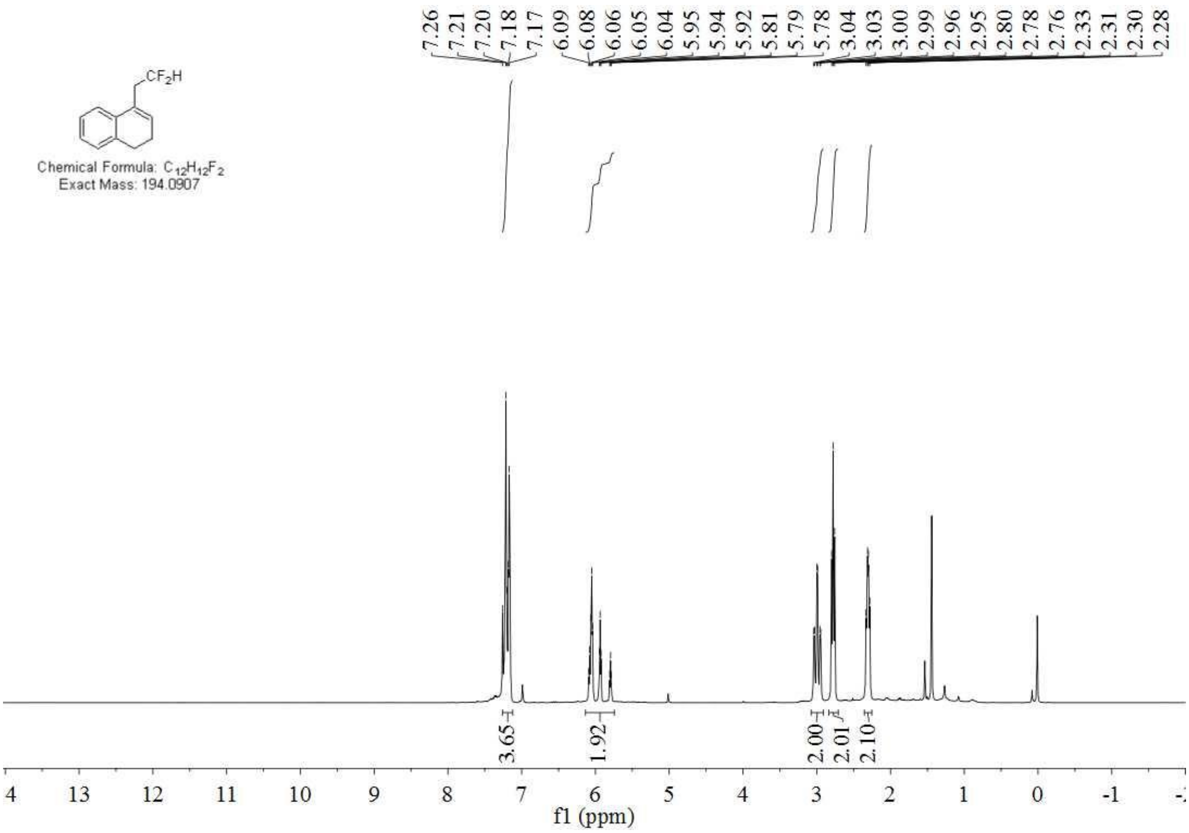

Supplementary Figure 129. **^{1}H-NMR of 4-(2,2-difluoroethyl)-1,2-dihydronaphthalene (9)**

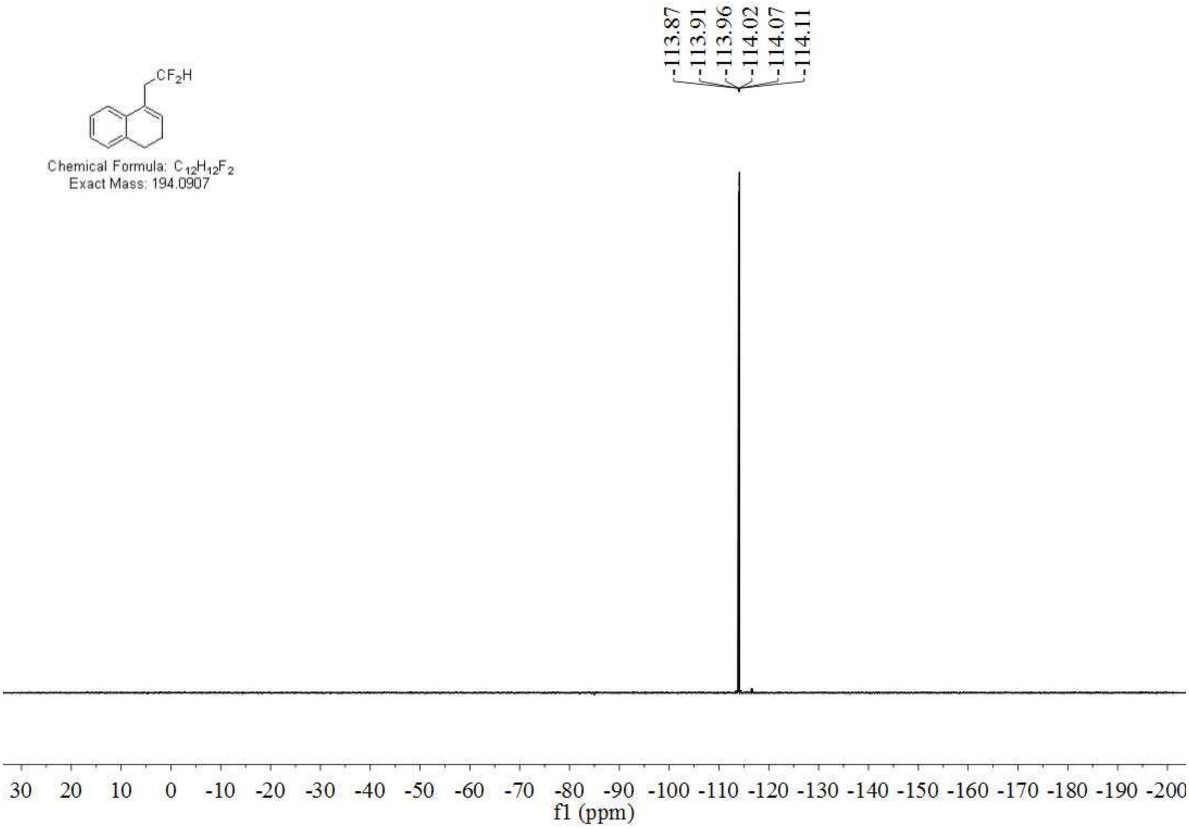

Supplementary Figure 130. **^{19}F-NMR of 4-(2,2-difluoroethyl)-1,2-dihydronaphthalene (9)**

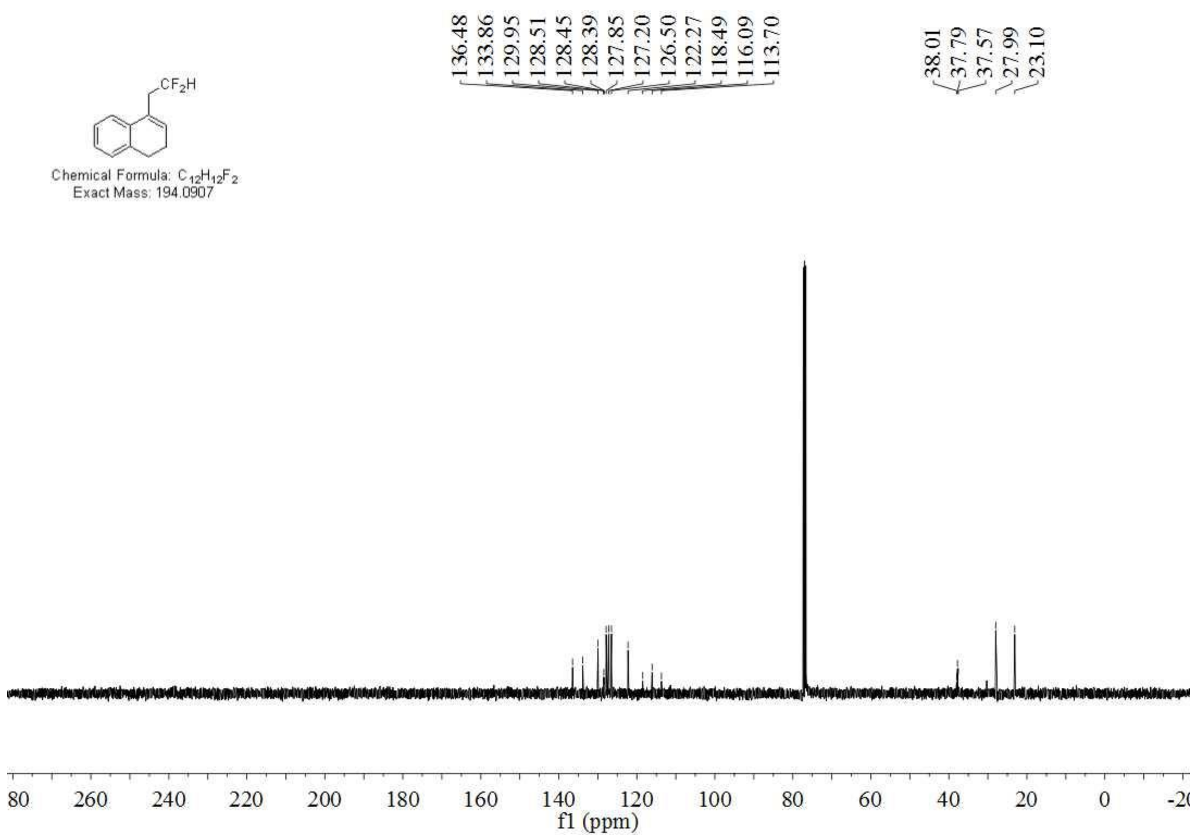

Supplementary Figure 131. ^{13}C-NMR of 4-(2,2-difluoroethyl)-1,2-dihydronaphthalene (9)

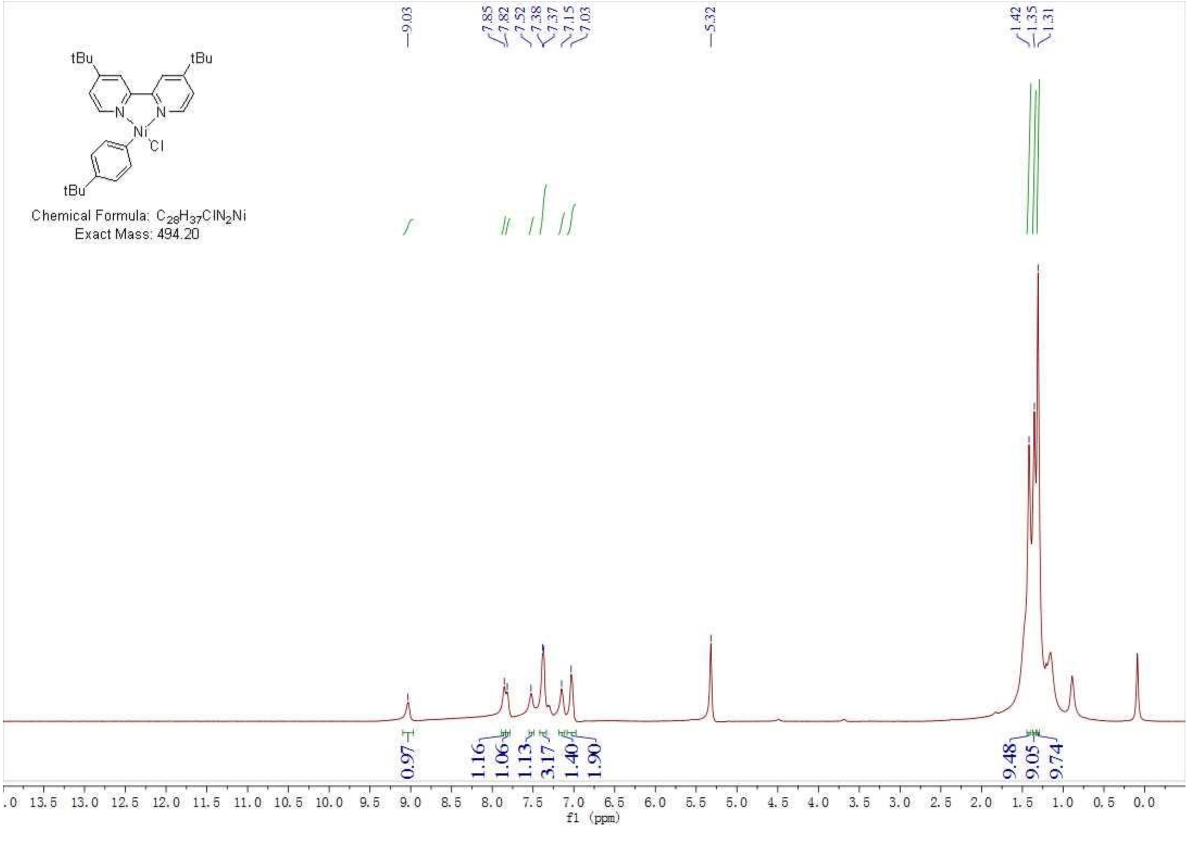

Supplementary Figure 132. ^{1}H-NMR of nickel complex B1

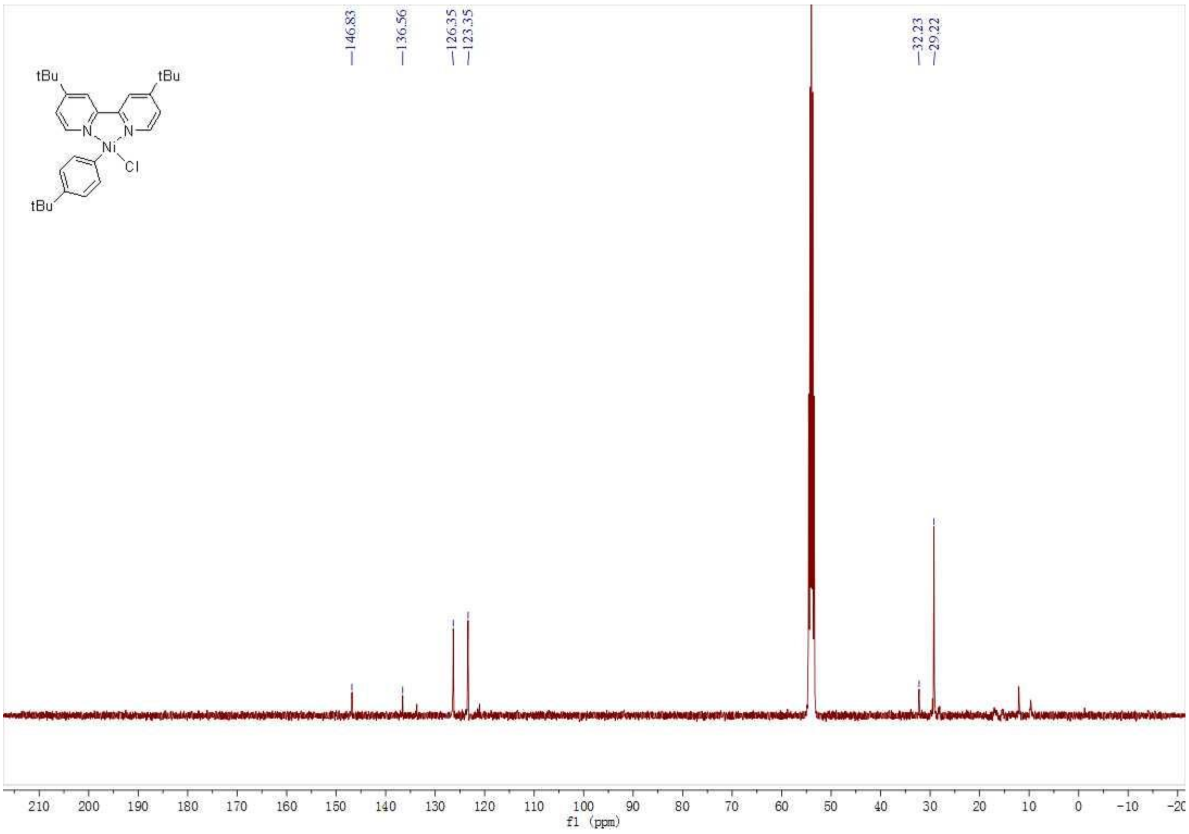

Supplementary Figure 133. ^{13}C-NMR of nickel complex B1

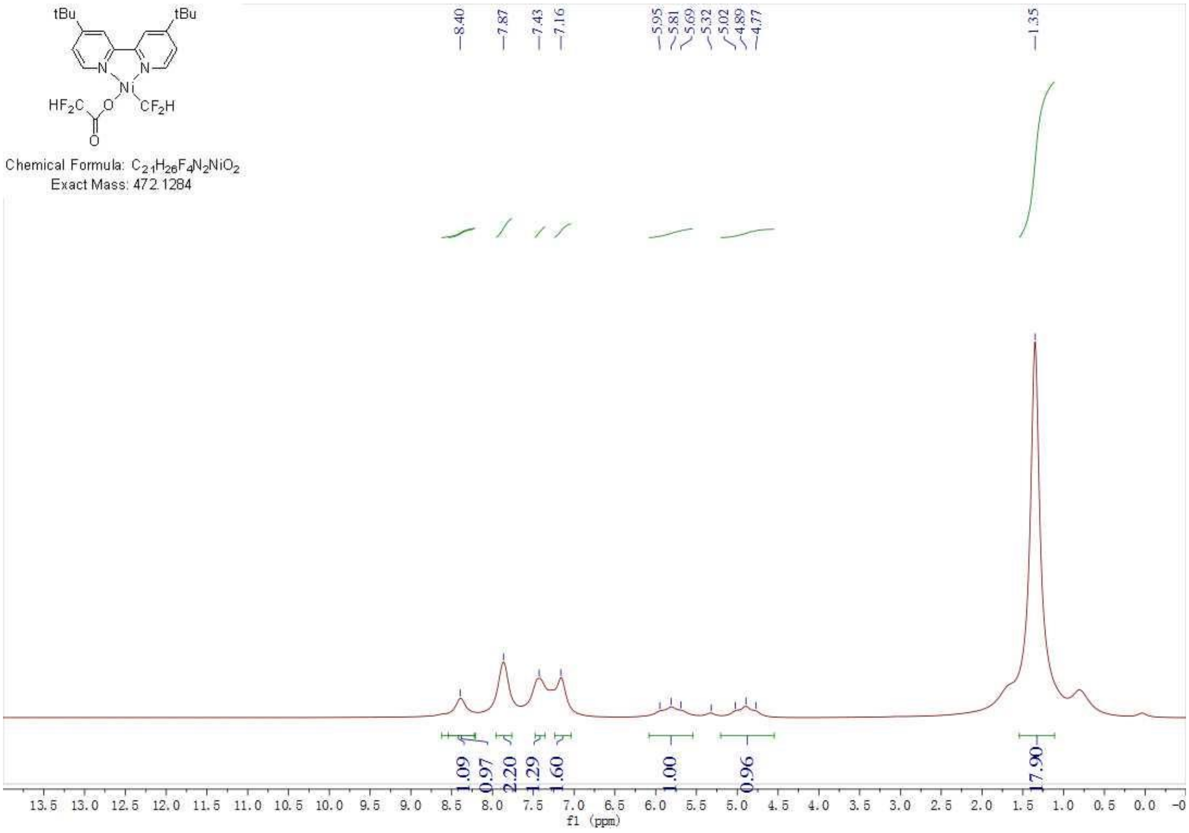

Supplementary Figure 134. ^{1}H-NMR of nickel complx C1

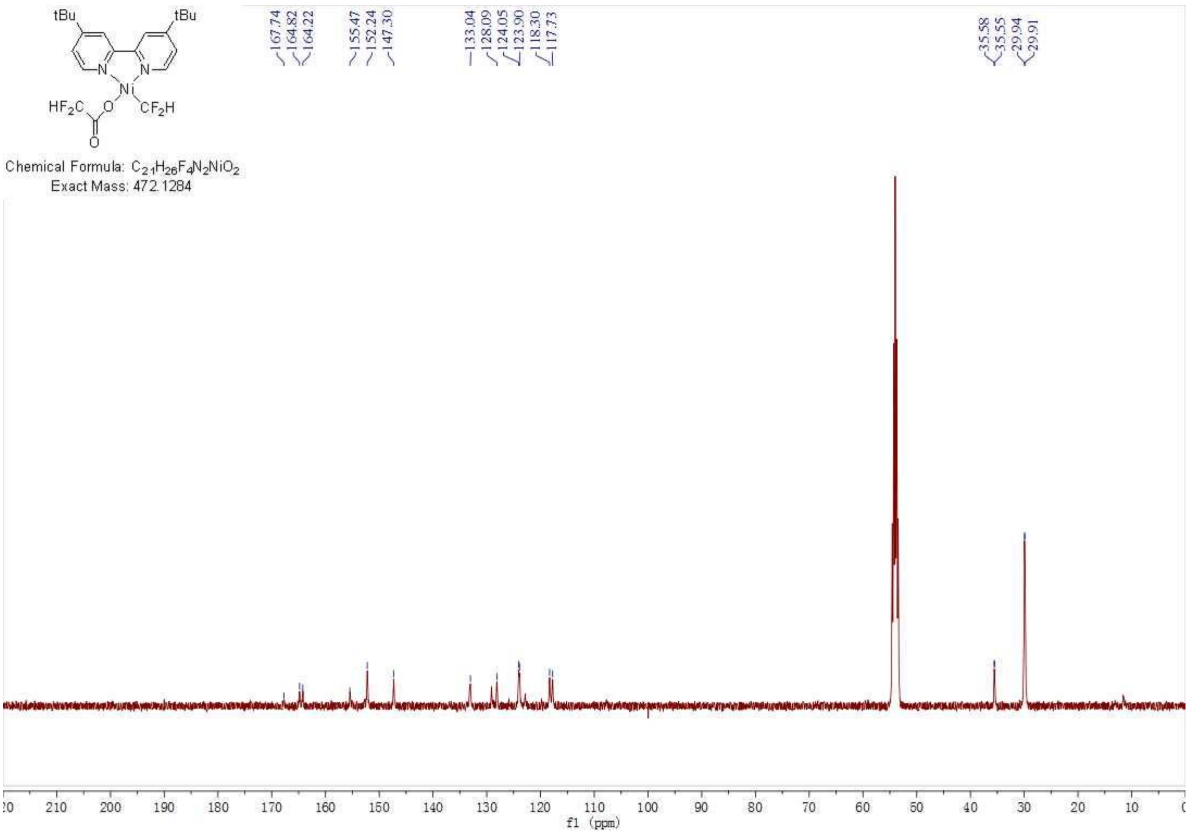

Supplementary Figure 135. ^{13}C-NMR of nickel complx C1

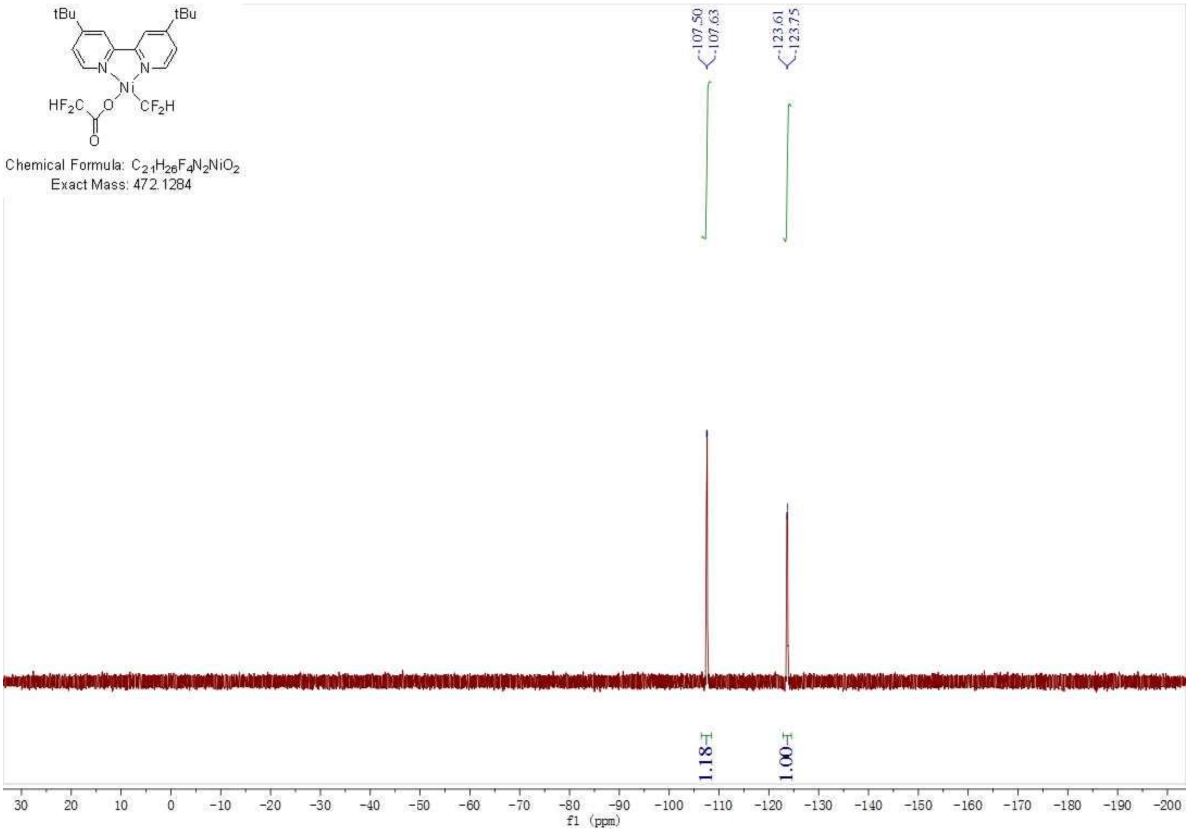

Supplementary Figure 136. ^{19}F-NMR of nickel complx C1

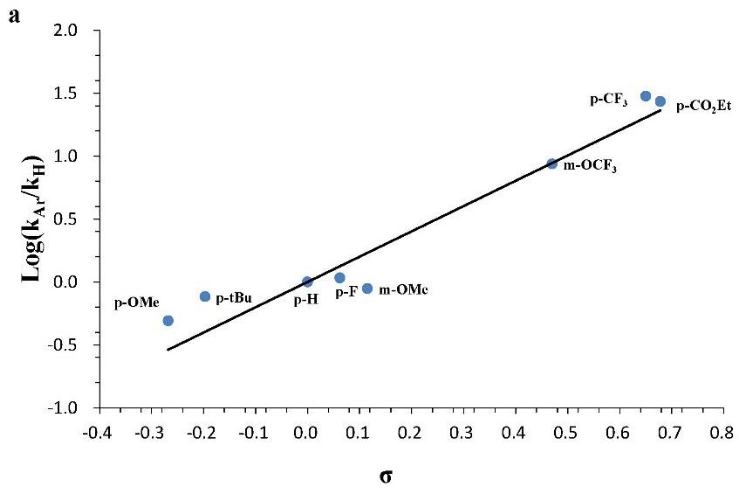

Supplementary Figure 137. Hammett plot of log(k_{rel}) versus σ, log(k_{rel}) = 2.0099σ, R^2 = 0.9328

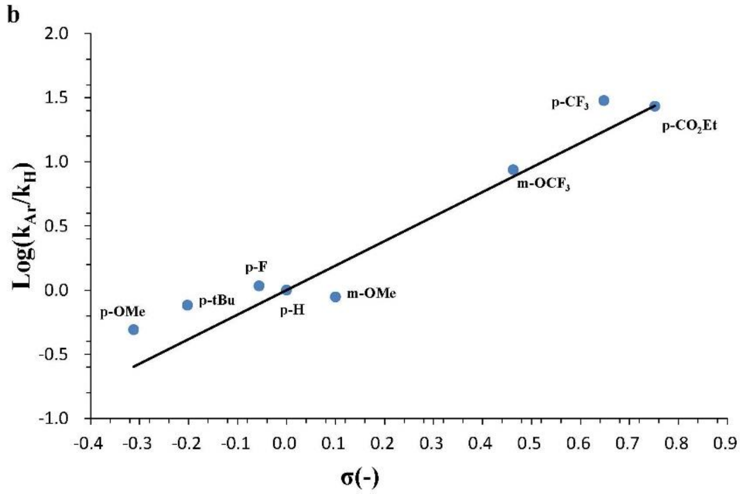

Supplementary Figure 138. Hammett plot of log(k_{rel}) versus σ(-), log(k_{rel}) = 1.9107σ(-), R^2 = 0.922

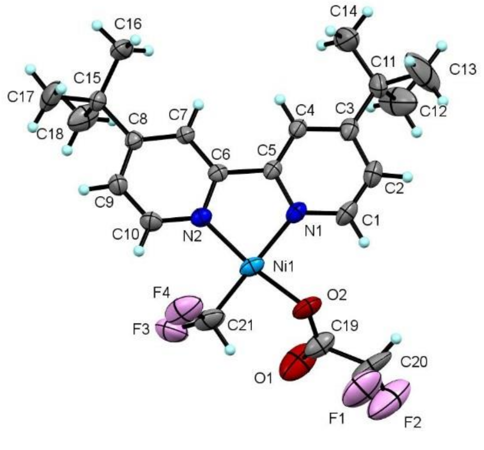

Supplementary Figure 139. X-ray crystal structure of nickel complex **C1**

Supplementary Table 1 Ligand effect on Ni-catalyzed cross-coupling of $ClCF_2H$ with **2a**[a]

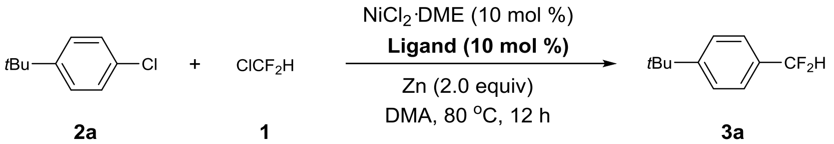

2a **1** **3a**

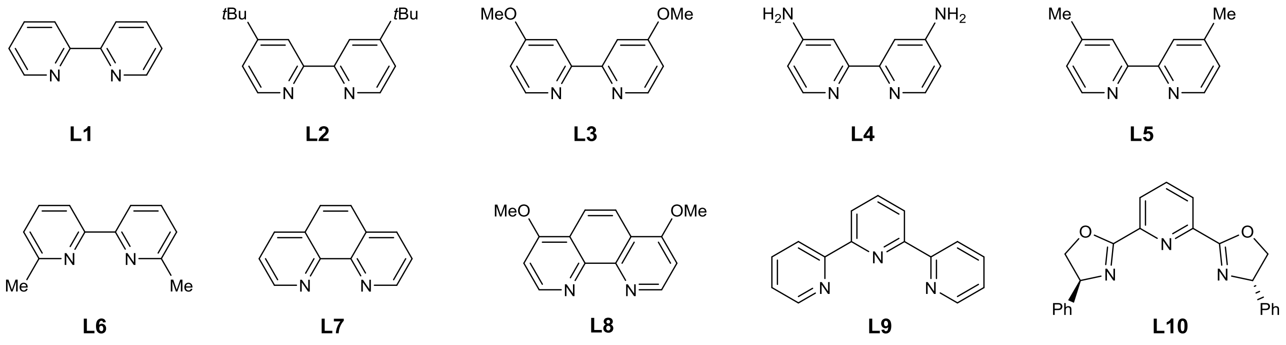

Entry	L	**3a**, yield (%)[b]	CF_2H_2, yield (%)[b]
1	**L1**	2	0
2	**L2**	4	0
3	**L3**	2	0
4	**L4**	2	0
5	**L5**	2	0
6	**L6**	0	0
7	**L7**	0	0
8	**L8**	0	0
9	**L9**	0	0
10	**L10**	0	0

[a]Reaction conditions (unless otherwise specified): **2a** (0.2 mmol, 1.0 equiv), **1** (2.6 M in DMA, 1.3 mmol, 6.5 equiv), DMA (2 mL). [b]Determined by ^{19}F NMR using fluorobenzene as an internal standard.

Supplementary Table 2 Ni-catalyzed cross-coupling of $ClCF_2H$ with **2a** in the presence of $MgCl_2$[a]

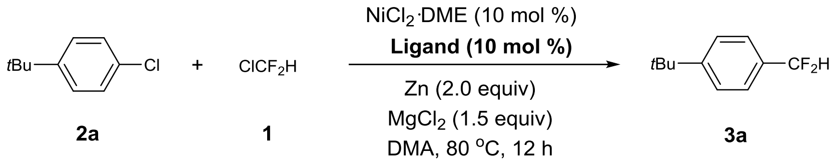

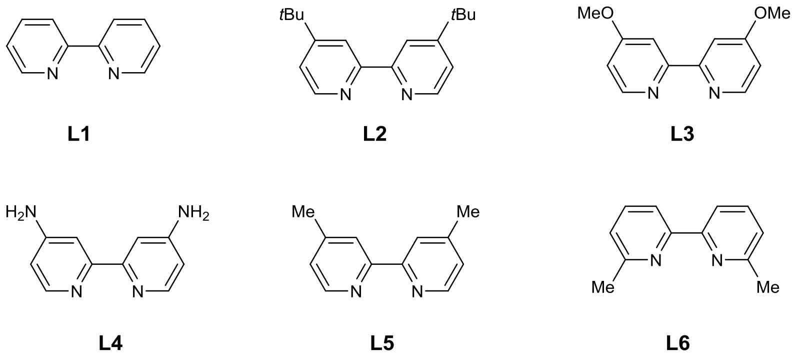

Entry	L	**3a**, yield (%)[b]	CF_2H_2, yield (%)[b]
1	**L1**	16	0
2	**L2**	21	0
3	**L3**	10	0
4	**L4**	33	0
5	**L5**	27	0
6	**L6**	0	0

[a]Reaction conditions (unless otherwise specified): **2a** (0.2 mmol, 1.0 equiv), **1** (2.6 M in DMA, 1.3 mmol, 6.5 equiv), DMA (2 mL). [b]Determined by ^{19}F NMR using fluorobenzene as an internal standard.

Supplementary Table 3 Additive effect on Ni-catalyzed cross-coupling of $ClCF_2H$ with **2a**[a]

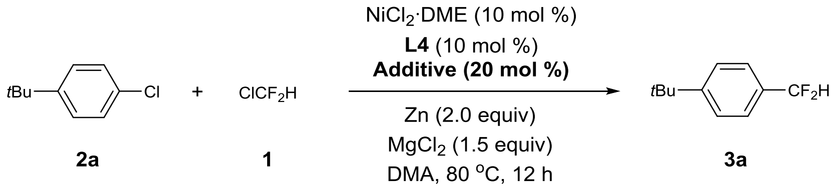

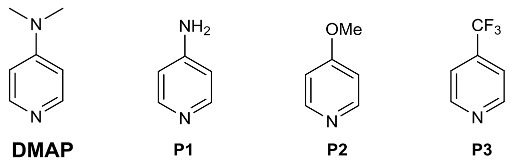

Entry	**Additive**	**3a**, yield (%)[b]	CF_2H_2, yield (%)[b]
1	**--**	33	0
2	**Py**	35	0
3	**DMAP**	46	1
4	**P1**	34	1
5	**P2**	38	1
6	**P3**	31	0
7	**PPh_3**	11	0

[a]Reaction conditions (unless otherwise specified): **2a** (0.2 mmol, 1.0 equiv), **1** (2.6 M in DMA, 1.3 mmol, 6.5 equiv), DMA (2 mL). [b]Determined by ^{19}F NMR using fluorobenzene as an internal standard.

Supplementary Table 4 Optimization of ratio of Zn/$MgCl_2$[a]

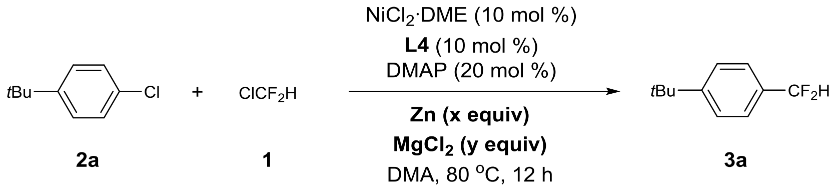

Entry	Zn (x)	$MgCl_2$ (y)	**3a**, yield (%)[b]	CF_2H_2, yield (%)[b]
1	2	1.5	46	1
2	3	2	44	0
3	3	4	55	1

[a]Reaction conditions (unless otherwise specified): **2a** (0.2 mmol, 1.0 equiv), **1** (2.6 M in DMA, 1.3 mmol, 6.5 equiv), DMA (2 mL). [b]Determined by ^{19}F NMR using fluorobenzene as an internal standard.

Supplementary Table 5 Screening of reaction temperature[a]

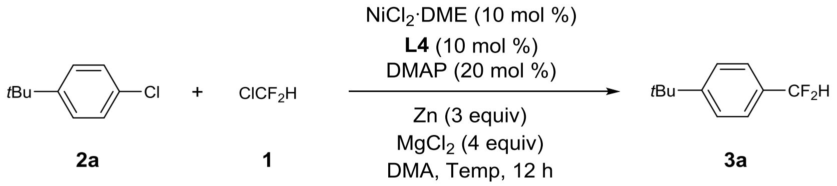

Entry	Temp (°C)	**3a**, yield (%)[b]	CF_2H_2, yield (%)[b]
1	40	33	0
2	60	54	0
3	80	55	1

[a]Reaction conditions (unless otherwise specified): **2a** (0.2 mmol, 1.0 equiv), **1** (2.6 M in DMA, 1.3 mmol, 6.5 equiv), DMA (2 mL). [b]Determined by ^{19}F NMR using fluorobenzene as an internal standard.

Supplementary Table 6 Solvent effect on Ni-catalyzed cross-coupling of $ClCF_2H$ with **2a**[a]

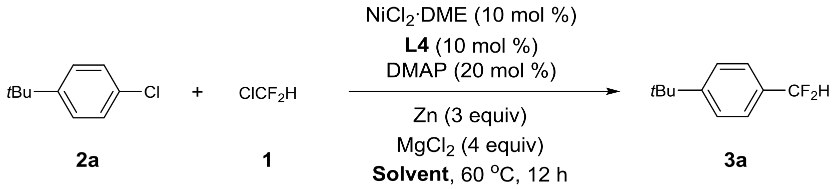

Entry	Solvent	**3a**, yield (%)[b]	CF_2H_2, yield (%)[b]
1	DMA	54	0
2	DMF	43	0
3	DMPU	43	0
4	NMP	22	0
5	dioxane	0	0
6	THF	0	0

[a]Reaction conditions (unless otherwise specified): **2a** (0.2 mmol, 1.0 equiv), **1** (2.6 M in DMA, 1.3 mmol, 6.5 equiv), DMA (2 mL). [b]Determined by ^{19}F NMR using fluorobenzene as an internal standard.

Supplementary Table 7 Screening of nickel sources[a]

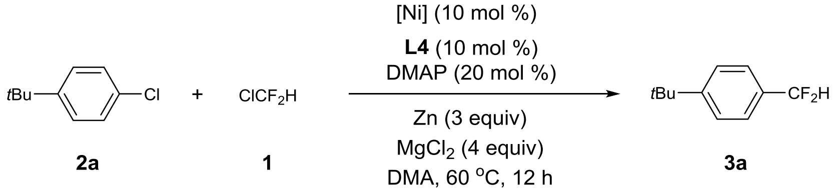

Entry	[Ni]	**3a**, yield (%)[b]	CF_2H_2, yield (%)[b]
1	$Ni(acac)_2$	29	1
2	$Ni(dppf)Cl_2$	8	0
3	$Ni(PPh_3)Br_2$	10	0
4	$Ni(NO_3)_2·6H_2O$	35	1
5	$NiCl_2$	59	1
6	$NiCl_2·DME$	54	1
7	$NiBr_2$	25	1
8	$NiBr_2·DME$	34	1
9	$NiBr_2·diglyme$	29	1
10	NiI_2	50	0

[a]Reaction conditions (unless otherwise specified): **2a** (0.2 mmol, 1.0 equiv), **1** (2.6 M in DMA, 1.3 mmol, 6.5 equiv), DMA (2 mL). [b]Determined by ^{19}F NMR using fluorobenzene as an internal standard.

Supplementary Table 8 Screening of ratio of $NiCl_2$/**L**[a]

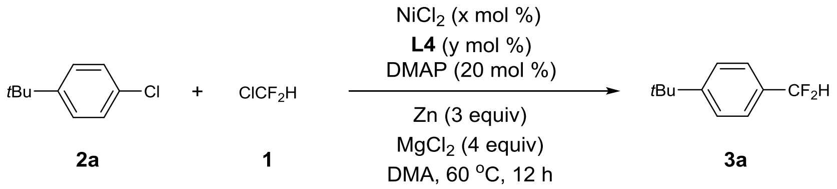

Entry	[Ni] (x) / **L4** (y)	**3a**, yield (%)[b]	CF_2H_2, yield (%)[b]
1	10 / 10	59	1
2	10 / 5	34	1
3	15 / 5	61	2
4	5 / 5	38	0
5	15 / 10	58	1
6	20 / 10	71	2
7	15 / 7.5	60	2

[a]Reaction conditions (unless otherwise specified): **2a** (0.2 mmol, 1.0 equiv), **1** (2.6 M in DMA, 1.3 mmol, 6.5 equiv), DMA (2 mL). [b]Determined by ^{19}F NMR using fluorobenzene as an internal standard.

Supplementary Table 9 The addition of 3 Å molecular sieve (MS) to the reaction[a]

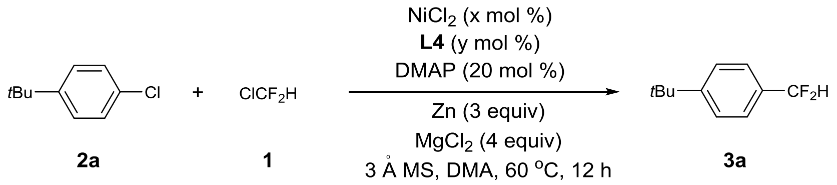

Entry	[Ni] (x) / **L4** (y)	**3a**, yield (%)[b]	CF_2H_2, yield (%)[b]
1	10 / 5	50	0
2[c]	10 / 10	46-76	0
3	15 / 10	79	0
4	15 / 7.5	60	0

[a]Reaction conditions (unless otherwise specified): **2a** (0.2 mmol, 1.0 equiv), **1** (2.6 M in DMA, 1.3 mmol, 6.5 equiv), DMA (2 mL), 3 Å MS (100 mg). [b]Determined by ^{19}F NMR using fluorobenzene as an internal standard. [c]The repeatability of these reaction conditions was poor.

Supplementary Table 10 Other nickel sources[a]

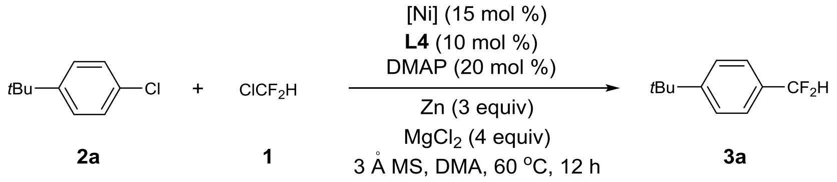

Entry	[Ni]	**3a**, yield (%)[b]	CF_2H_2, yield (%)[b]
1	$NiCl_2$	79	0
2	$NiBr_2$	77	0
3	$NiBr_2$·diglyme	78	0

[a]Reaction conditions (unless otherwise specified): **2a** (0.2 mmol, 1.0 equiv), **1** (2.6 M in DMA, 1.3 mmol, 6.5 equiv), DMA (2 mL), 3 Å MS (100 mg). [b]Determined by ^{19}F NMR using fluorobenzene as an internal standard.

Supplementary Table 11 Screening of loading amount of $MgCl_2$ in the absence of DMAP[a]

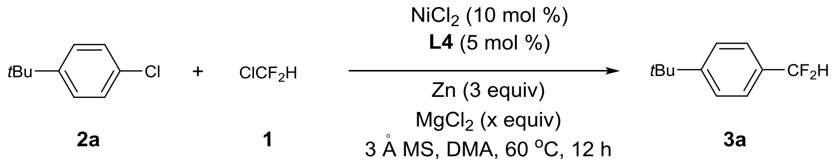

Entry	$MgCl_2$ (x)	**3a**, yield (%)[b]	CF_2H_2, yield (%)[b]
1	1	7	0
2	2	6	0
3	2.5	6	0
4	3	8	0
5	4	9	0
6	5	7	0

[a]Reaction conditions (unless otherwise specified): **2a** (0.2 mmol, 1.0 equiv), **1** (2.6 M in DMA, 1.3 mmol, 6.5 equiv), DMA (2 mL), 3 Å MS (100 mg). [b]Determined by ^{19}F NMR using fluorobenzene as an internal standard.

Supplementary Table 12 Screening of solvents in the absence of DMAP[a]

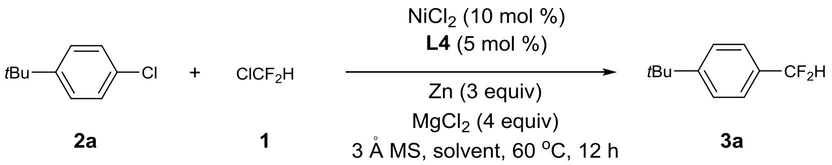

Entry	Solvent	**3a**, yield (%)[b]	CF_2H_2, yield (%)[b]
1	DMA	8	0
2	DMF	27	0
3	DMPU	6	0
4	NMP	18	0
5	dioxane	0	0

[a]Reaction conditions (unless otherwise specified): **2a** (0.2 mmol, 1.0 equiv), **1** (2.6 M in DMA, 1.3 mmol, 6.5 equiv), solvent (2 mL), 3 Å MS (100 mg). [b]Determined by ^{19}F NMR using fluorobenzene as an internal standard.

Supplementary Table 13 Screening of other additives[a]

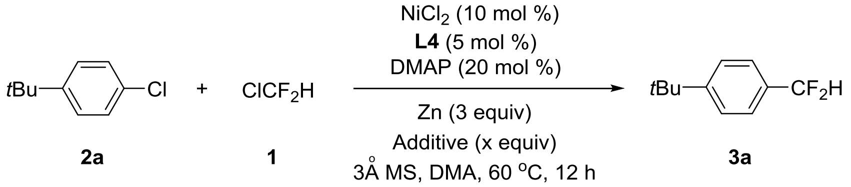

Entry	Additve (x)	**3a**, yield (%)[b]	CF_2H_2, yield (%)[b]
1	$MgCl_2$ (4)	54	0
2	TMSCl (4)	4	0
3	DIBAL-H (0.1)	0	0
4	HCl (0.1)	0	0

[a]Reaction conditions (unless otherwise specified): **2a** (0.2 mmol, 1.0 equiv), **1** (2.6 M in DMA, 1.3 mmol, 6.5 equiv), DMA (2 mL), 3 Å MS (100 mg). [b]Determined by ^{19}F NMR using fluorobenzene as an internal standard.

Supplementary Table 14 Rplacement of Zn with TDAE[a]

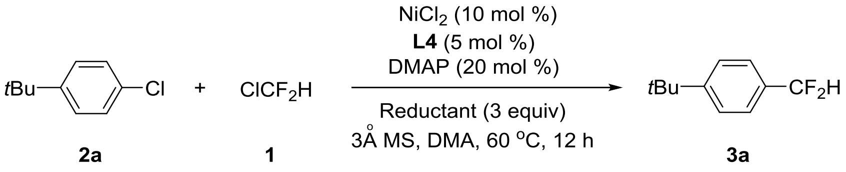

Entry	Reductant	**3a**, yield (%)[b]	CF_2H_2, yield (%)[b]
1	Zn (3.0 equiv) + $MgCl_2$ (4.0 equiv)	54	0
2	TDAE (3.0 equiv)	0	0

[a]Reaction conditions (unless otherwise specified): **2a** (0.2 mmol, 1.0 equiv), **1** (2.6 M in DMA, 1.3 mmol, 6.5 equiv), DMA (2 mL), 3 Å MS (100 mg). [b]Determined by ^{19}F NMR using fluorobenzene as an internal standard.

Supplementary Table 15 Using excess **L4**[a]

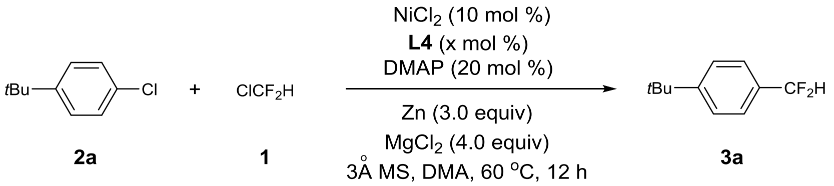

Entry	**L4** (x)	**3a**, yield (%)[b]	CF_2H_2, yield (%)[b]
1	5	54	0
2	15	34	0

[a]Reaction conditions (unless otherwise specified): **2a** (0.2 mmol, 1.0 equiv), **1** (2.6 M in DMA, 1.3 mmol, 6.5 equiv), DMA (2 mL), 3 Å MS (100 mg). [b]Determined by ^{19}F NMR using fluorobenzene as an internal standard.

Supplementary Methods

General Information ^{1}H NMR and ^{13}C NMR spectra were recorded on a Bruker AM400 and AM500 spectrometer and are calibrated using residual undeuterated solvent ($CHCl_3$ at 7.26 ppm ^{1}H NMR, 77.00 ppm ^{13}C NMR). ^{19}F NMR was recorded on a Bruker AM400 spectrometer ($CFCl_3$ as an external standard and low field is positive). Chemical shifts (δ) are reported in ppm, and coupling constants (*J*) are in Hertz (Hz). The following abbreviations were used to explain the multiplicities: s = singlet, d = doublet, t = triplet, q = quartet, m = multiplet, br = broad. NMR yield was determined by ^{19}F NMR using fluorobenzene as an internal standard before working up the reaction.

Materials All reagents were used as received from commercial sources and used without further purification. DMF, DMA, DMPU, NMP were distilled under reduced pressure from CaH_2. 1,4-Dioxane and THF were distilled from sodium and benzophenone immediately before use. Zinc dust was activated according to the literature before use. All (hetero)aryl chlorides and bromides were used from commercial suppliers.

Preparation of $ClCF_2H$ Stock Solution

Anhydrous DMA (100 mL) was added to a Schlenk tube under argon atomosphere (Ar). $ClCF_2H$ gas was then slowly bubbled through the DMA until the total volume of the solution reach the maximum (generally 2 hours). The concentration of the $ClCF_2H$ stock solution was determined by ^{19}F NMR using fluorobenzene as an internal standard (generally 2.0 ~ 2.6 mol/L). This solution could be stored at refrigerator (-4 ^{o}C) for one month without loss of $ClCF_2H$.

General Procedure of Ni-Catalyzed Cross-Coupling of $ClCF_2H$ with Aryl Chloride 2a

To a 25 mL of Schlenk tube were added aryl chloride **2, 4** or **6** (0.2 mmol, 1.0 equiv), $NiCl_2$ (10 mol %), **L4** (5 mol %), zinc dust (3.0 equiv), $MgCl_2$ (4.0 equiv), 3Å MS (100 mg) and DMAP (20 mol %). The mixture was evacuated and backfilled with argon for three times, DMA (2 mL) and $ClCF_2H$ **1** (2.6 M in DMA, 1.3 mmol, 6.5 equiv) were then added. The Schlenk tube was screw capped and put into a preheated oil bath (60 ^{o}C). After stirring for 20 h, the reaction mixture was cooled to room temperature and diluted with ethyl acetate (2 mL). The yield was determined by ^{19}F NMR using fluorobenzene as an internal standard before working up. Then the reaction mixture was filtered with a pad of cellite. The filtrate was washed with brine, extracted with EtOAc for three times. Then the organic layer was dried over Na_2SO_4 and concentrated. The residue was purified with silica gel chromatography to give product **3**, **5** or **7**. Isolated yields are based on the average of two runs under identical conditions.

Characterization Data for Difluoromethylated (Hetero)Arenes 3, 5 and 7

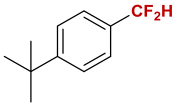

1-(*tert*-Butyl)-4-(difluoromethyl)benzene (3a). [known compound[1]]: The reaction carried out with $NiCl_2$ (15 mol %) and **L4** (10 mol %). The product **3a** (24.0 mg, 65% yield, 79% yield determined by ^{19}F NMR) as an oil was purified with silica gel chromatography (Hexane). ^1H NMR (400 MHz, $CDCl_3$) δ 7.47 (m, 4H), 6.63 (t, J = 56.6 Hz, 1H), 1.35 (s, 9H). ^{19}F NMR (376 MHz, $CDCl_3$) δ -109.9 (d, J = 56.4 Hz, 2F). ^{13}C NMR (101 MHz, $CDCl_3$) δ 154.0, 131.5 (t, J = 22.2 Hz), 125.6, 125.3 (t, J = 6.0 Hz), 114.9 (t, J = 238.9 Hz), 34.8, 31.2.

3 mmol-Scale Synthesis by Using 2.0 equiv of $ClCF_2H$: To a 25 mL of Schlenk tube were added $NiCl_2$ (15 mol %), **L4** (10 mol %), zinc dust (3.0 equiv), $MgCl_2$ (4.0 equiv), 3Å MS (700 mg) and DMAP (20 mol %). The mixture was evacuated and backfilled with argon for three times, aryl chloride **2a** (3 mmol), DMA (8 mL) and $ClCF_2H$ **1** (2.6 M in DMA, 6 mmol, 2.0 equiv) were then added. The Schlenk tube was screw capped and put into a preheated oil bath (60 °C). After stirring for 20 h, the reaction mixture was cooled to room temperature and diluted with ethyl acetate (10 mL). The yield (83%) was determined by ^{19}F NMR using fluorobenzene as an internal standard.

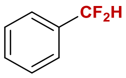

(Difluoromethyl)benzene (3b) [known compound[2]]: Due to the low boiling point of the product **3b**, the yield (78%) was determined by ^{19}F NMR using fluorobenzene as an internal standard. The product was characterized by ^{19}F NMR and GC-MS analysis. ^{19}F NMR (376 MHz) δ -110.4 (d, J = 56.4 Hz, 2F). GC-MS (EI): *m/z* (%) 128 (M^+).

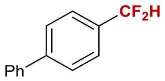

4-(Difluoromethyl)-1,1'-biphenyl (3c). [known compound[1]]: The product **3c** (for arylchloride: 36.7 mg, 90% yield; for arylbromide: 32.6 mg, 80%) as a white soild (m.p. 74 ~ 77 ℃) was purified with silica gel chromatography (Hexane). ^1H NMR (400 MHz, $CDCl_3$) δ 7.69 (d, J = 8.3 Hz, 2H), 7.61 (m, 4H), 7.48 (m, 2H), 7.40 (t, J = 7.3 Hz, 1H), 6.71 (t, J = 56.4 Hz, 1H). ^{19}F NMR (376 MHz, $CDCl_3$) δ

-110.3 (d, J = 56.4 Hz, 2F). ^{13}C NMR (126 MHz, $CDCl_3$) δ 143.7 (t, J = 2.0 Hz), 140.2, 133.2 (t, J = 22.1 Hz), 128.9, 127.9, 127.4, 127.2, 126.0 (t, J = 6.0 Hz), 114.7 (t, J = 238.8 Hz).

10-Gram-Scale Synthesis: To a 350 mL of reaction vessel were added aryl chloride **2c** (11.3 g, 60 mmol, 1.0 equiv), $NiCl_2$ (10 mol %), **L4** (5 mol %), zinc dust (3.0 equiv), $MgCl_2$ (4.0 equiv), 3 Å MS (15 g) and DMAP (20 mol %) under Ar. DMA (100 mL) and $ClCF_2H$ **1** (2.6 M in DMA, 360 mmol, 6 equiv) were added subsequently. The vessel was screw capped and put into a preheated oil bath (60 ^{o}C). After stirring for 36 h, the reaction mixture was cooled to room temperature and diluted with ethyl acetate (100 mL). The yield was determined by ^{19}F NMR using fluorobenzene as an internal standard before working up. Then the reaction mixture was filtered with a pad of cellite. The filtrate was extracted with petroleum ether (200 mL ×3 times) and washed with brine. The organic layer was evaporated. The residue was purified with silica gel chromatography (Hexane) to give product **3c** (9.8 g, 80%) as a white solid.

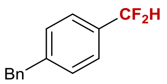

1-Benzyl-4-(difluoromethyl)benzene (3d). The product **3d** (32.7 mg, 75% yield) as a colorless oil was purified with silica gel chromatography (Hexane). ^{1}H NMR (400 MHz, $CDCl_3$) δ 7.44 (d, J = 7.9 Hz, 2H), 7.36 – 7.16 (m, 7H), 6.62 (t, J = 56.6 Hz, 1H), 4.03 (s, 2H). ^{19}F NMR (376 MHz, $CDCl_3$) δ -109.9 (d, J = 56.4 Hz, 2F). ^{13}C NMR (101 MHz, $CDCl_3$) δ 144.0 (t, J = 1.7 Hz), 140.3, 132.3 (t, J = 22.7 Hz), 129.2, 128.9, 128.6, 126.3, 125.1 (t, J = 6.0 Hz), 114.8 (t, J = 238.9 Hz), 41.7. MS (EI): *m/z* (%) 218 (M^+). HRMS: Calculated for $C_{14}H_{12}F_2$: 218.0907; Found: 218.0902.

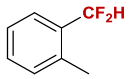

1-(Difluoromethyl)-2-methylbenzene (3e). [known compound[3]]: The reaction was carried out with $NiCl_2$ (15 mol%) and **L4** (10 mol%). Due to the low boiling point of the product **3e**, the yield (37%) was determined by ^{19}F NMR using fluorobenzene as an internal standard. The product was characterized by ^{19}F NMR and GC-MS analysis. ^{19}F NMR (376 MHz) δ -113.6 (d, J = 56.4 Hz, 2F). GC-MS (EI): *m/z* (%) 142 (M^+).

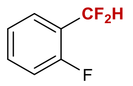

1-(Difluoromethyl)-2-fluorobenzene (3f). [known compound[4]]: The reaction was carried out with $NiCl_2$ (15 mol%) and **L4** (10 mol%). Due to the low boiling point of the product **3f**, the yield (43%) was determined by ^{19}F NMR using trifluorotoluene as an internal standard. The product was characterized by ^{19}F NMR and GC-MS analysis. ^{19}F NMR (376 MHz) δ -114.23 (d, *J* = 54.4 Hz, 2F). GC-MS (EI): *m/z* (%) 145.0 ($[M-H]^+$).

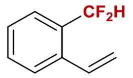

1-(Difluoromethyl)-2-vinylbenzene (3g). The reaction was carried out with $NiCl_2$ (15 mol%) and **L4** (10 mol%). The product **3g** (15.1 mg, 49% yield) as a colorless oil was purified with silica gel chromatography (Hexane). ^{1}H NMR (400 MHz, $CDCl_3$) δ 7.60 – 7.53 (m, 2H), 7.46 (t, *J* = 7.6 Hz, 1H), 7.37 (t, *J* = 7.6 Hz, 1H), 7.05 (dd, *J* = 17.3, 11.1 Hz, 1H), 6.82 (t, *J* = 55.3 Hz, 1H), 5.72 (d, *J* = 17.3 Hz, 1H), 5.45 (d, *J* = 11.0 Hz, 1H). ^{19}F NMR (376 MHz, $CDCl_3$) δ -110.92 (d, *J* = 55.3 Hz, 2F). ^{13}C NMR (101 MHz, $CDCl_3$) δ 136.75 (t, *J* = 4.4 Hz), 132.64 , 130.88 , 130.74 (t, *J* = 1.7 Hz), 127.73 , 126.60 , 125.80 (t, *J* = 7.4 Hz), 118.27 , 114.02 (t, *J* = 238.0 Hz). MS (EI): *m/z* (%) 154 (M^+), 63(100). HRMS: Calculated for $C_9H_8F_2$: 154.0594; Found: 154.0595.

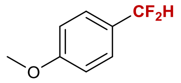

1-(Difluoromethyl)-4-methoxybenzene (3h). [known compound[1]]: The reaction was carried out with $NiBr_2$ (20 mol%) and **L4** (10 mol%). Due to the low boiling point of the product **3h**, the yield (41%) was determined by ^{19}F NMR using fluorobenzene as an internal standard. The product was characterized by ^{19}F NMR and GC-MS analysis. ^{19}F NMR (376 MHz) δ -108.0 (d, *J* = 56.4 Hz, 2F). GC-MS (EI): *m/z* (%) 158 (M^+).

3 mmol-Scale Synthesis by Using 2.0 equiv of $ClCF_2H$: To a 25 mL of Schlenk tube were added $NiBr_2$ (15 mol %), **L4** (10 mol %), zinc dust (3.0 equiv), $MgCl_2$ (4.0 equiv), 3Å MS (700 mg) and DMAP (20 mol %). The mixture was evacuated and backfilled with argon for three times, aryl chloride **2h** (3 mmol), DMA (8 mL) and $ClCF_2H$ **1** (2.6 M in DMA, 6 mmol, 2.0 equiv) were then added. The Schlenk tube was screw capped and put into a preheated oil bath (60 °C). After stirring

for 20 h, the reaction mixture was cooled to room temperature and diluted with ethyl acetate (10 mL). The yield (75%) was determined by ^{19}F NMR using fluorobenzene as an internal standard.

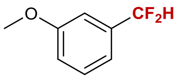

1-(Difluoromethyl)-3-methoxybenzene (3i). [known compound[2]]: The reaction was carried out with $NiCl_2$ (15 mol%) and **L4** (10 mol%). Due to the low boiling point of the product **3i**, the yield (for arylchloride: 70% yield; for arylbromide: 65% yield) was determined by ^{19}F NMR using fluorobenzene as an internal standard. The product was characterized by ^{19}F NMR and GC-MS analysis. ^{19}F NMR (376 MHz) δ -110.5 (d, *J* = 56.4 Hz, 2F). GC-MS (EI): *m/z* (%) 158 (M^+).

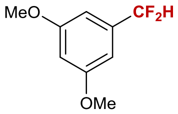

1-(Difluoromethyl)-3,5-dimethoxybenzene (3j). [known compound[1]]: The product **3j** (23.7 mg, 63% yield) as a colorless oil was purified with silica gel chromatography (Hexane / EtOAc = 100:1). ^{1}H NMR (400 MHz, $CDCl_3$) δ 6.76 – 6.38 (m, 4H), 3.82 (s, 6H). ^{19}F NMR (376 MHz, $CDCl_3$) δ -110.9 (d, *J* = 56.4 Hz, 2F). ^{13}C NMR (126 MHz, $CDCl_3$) δ 161.0, 136.3 (t, *J* = 22.7 Hz), 114.46 (t, *J* = 240.4 Hz), 103.4 (t, *J* = 6.3 Hz), 102.7 (t, *J* = 1.7 Hz), 55.5.

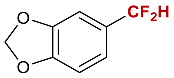

5-(Difluoromethyl)benzo[*d*][1,3]dioxole (3k). [known compound[1]]: The product **3k** (19.0 mg, 55% yield, 60% yield determined by ^{19}F NMR) as a colorless oil was purified with silica gel chromatography (Hexane / EtOAc = 100:1). ^{1}H NMR (400 MHz, $CDCl_3$) 6.98 (m, 2H), 6.85 (d, *J* = 7.8 Hz, 1H), 6.55 (t, *J* = 56.6 Hz, 1H), 6.02 (d, *J* = 1.6 Hz, 2H). ^{19}F NMR (376 MHz, $CDCl_3$) δ -108.0 (d, *J* = 56.4 Hz, 2F). ^{13}C NMR (101 MHz, $CDCl_3$) δ 149.5 (t, *J* = 1.9 Hz), 148.0, 128.3 (t, *J* = 22.7 Hz), 120.1 (t, *J* = 7.2 Hz), 114.6 (t, *J* = 239.4 Hz), 108.2, 105.8 (t, *J* = 5.5 Hz), 101.6.

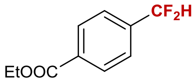

Ethyl 4-(difluoromethyl)benzoate (3l). [known compound[1]]: The reaction was carried out with $NiCl_2$ (15 mol%) and **L4** (10 mol%). The product **3l** (for arylchloride: 27.2 mg, 68% yield; for arybormide: 23.2 mg, 58% yield) as a colorless oil was purified with silica gel chromatography (Hexane / EtOAc = 50:1). ^{1}H NMR (400 MHz, $CDCl_3$) δ 8.13 (d, J = 7.9 Hz, 2H), 7.58 (d, J = 7.9 Hz, 2H), 6.69 (t, J = 56.1 Hz, 1H), 4.40 (q, J = 7.1 Hz, 2H), 1.41 (t, J = 7.1 Hz, 3H). ^{19}F NMR (376 MHz, $CDCl_3$) δ -112.2 (d, J = 56.4 Hz, 2F). ^{13}C NMR (126 MHz, $CDCl_3$) δ 165.7, 138.3 (t, J = 22.7 Hz), 132.7 (t, J = 1.9 Hz), 129.9, 125.6 (t, J = 6.0 Hz), 114.0 (t, J = 240.0 Hz), 61.3, 14.2.

3 mmol-Scale Synthesis by Using 2.0 equiv of $ClCF_2H$: To a 25 mL of Schlenk tube were added $NiCl_2$ (15 mol %), **L4** (10 mol %), zinc dust (3.0 equiv), $MgCl_2$ (4.0 equiv), 3Å MS (700 mg) and DMAP (20 mol %). The mixture was evacuated and backfilled with argon for three times, aryl chloride **2l** (3 mmol), DMA (8 mL) and $ClCF_2H$ **1** (2.6 M in DMA, 6 mmol, 2.0 equiv) were then added. The Schlenk tube was screw capped and put into a preheated oil bath (60 °C). After stirring for 20 h, the reaction mixture was cooled to room temperature and diluted with ethyl acetate (10 mL). The reaction mixture was filtered with a pad of cellite. The filtrate was washed with brine, extracted with EtOAc for three times. The combined organic layers were dried over Na_2SO_4 and concentrated. The residue was purified with silica gel chromatography to give product **3l** (300 mg, 50% yield) as a colorless oil.

10-Gram Scale Synthesis: To a 350 mL of reaction vessel were added aryl chloride **2l** (11.1 g, 60 mmol, 1.0 equiv), $NiCl_2$ (10 mol %), **L4** (5 mol %), zinc dust (3.0 equiv), $MgCl_2$ (4.0 equiv), 3 Å MS (10 g) and DMAP (20 mol %) under Ar. DMA (150 mL) and $ClCF_2H$ **1** (2.6 M in DMA, 240 mmol, 4 equiv) were added subsequently. The vessel was screw capped and put into a preheated oil bath (60 °C). After stirring for 36 h, the reaction mixture was cooled to room temperature and diluted with ethyl acetate (100 mL). The yield was determined by ^{19}F NMR using fluorobenzene as an internal standard before working up. Then the reaction mixture was filtered with a pad of cellite. The filtrate was extracted with ethyl acetate (200 mL×3 times) and washed with brine. The organic layer was evaporated. The residue was purified with silica gel chromatography (Hexane/EtOAc = 40/1) to give product **3l** (8.8 g, 74%) as a colorless oil.

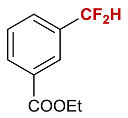

Ethyl 3-(difluoromethyl)benzoate (3m). [known compound[1]]: The reaction was carried out with $NiCl_2$ (15 mol%) and **L4** (10 mol%). The product **3m** (28.8 mg, 72% yield) as a colorless oil was purified with silica gel chromatography (Hexane / EtOAc = 50:1). ^{1}H NMR (400 MHz, $CDCl_3$) δ 8.17 (d, J = 11.8 Hz, 2H), 7.71 (d, J = 7.7 Hz, 1H), 7.54 (t, J = 7.7 Hz, 1H), 6.69 (t, J = 56.2 Hz, 1H), 4.40 (q, J = 7.1 Hz, 2H), 1.41 (t, J = 7.1 Hz, 3H). ^{19}F NMR (376 MHz, $CDCl_3$) δ -111.2 (d, J = 56.4 Hz, 2F). ^{13}C NMR (101 MHz, $CDCl_3$) δ 165.7, 134.7 (t, J = 23.2 Hz), 131.8 (t, J = 1.6 Hz), 131.1, 129.7 (t, J = 5.7 Hz), 128.9, 126.9 (t, J = 6.2 Hz), 114.1 (t, J = 240.5 Hz), 61.4, 14.3.

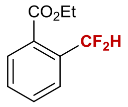

Ethyl 2-(difluoromethyl)benzoate (3n). [known compound[5]]: The reaction was carried out with $NiCl_2$ (15 mol%) and **L4** (10 mol%). The product **3n** (23.6 mg, 59% yield) as a colorless oil was purified with silica gel chromatography (Hexane / EtOAc = 50:1). ^{1}H NMR (400 MHz, $CDCl_3$) δ 8.04 (d, J = 7.8 Hz, 1H), 7.81 (d, J = 7.7 Hz, 1H), δ 7.63 (t, J = 7.6 Hz, 1H), δ 7.54 (t, J = 55.6 Hz, 1H), 7.53 (t, J = 7.5 Hz, 1H), 4.40 (q, J = 7.1 Hz, 2H), 1.41 (t, J = 7.1 Hz, 3H). ^{19}F NMR (376 MHz, $CDCl_3$) δ -113.77 (d, J = 55.6 Hz). ^{13}C NMR (126 MHz, $CDCl_3$) δ 165.95, 135.19 (t, J = 22.2 Hz), 132.53 , 130.78 , 130.29 (t, J = 1.9 Hz), 129.08 (t, J = 5.2 Hz), 125.85 (t, J = 8.1 Hz), 111.97 (t, J = 237.6 Hz), 61.57 , 14.11 .

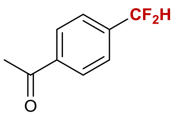

1-(4-(Difluoromethyl)phenyl)ethan-1-one (3o). [known compound[1]]: The reaction was carried out with $NiCl_2$ (15 mol%) and **L4** (10 mol%). The product **3o** (22.1 mg, 65% yield) as a colorless oil was purified with silica gel chromatography (Hexane / EtOAc = 50:1). ^{1}H NMR (400 MHz, $CDCl_3$) δ 8.04 (d, J = 7.9 Hz, 2H), 7.61 (d, J = 7.9 Hz, 2H), 6.69 (t, J = 56.1 Hz, 1H), 2.63 (s, 3H).^{19}F NMR (376 MHz, $CDCl_3$) δ -112.4 (d, J = 56.4 Hz, 2F). ^{13}C NMR (126 MHz, $CDCl_3$) δ 197.3, 138.85 (t, J = 1.8 Hz), 138.5 (t, J = 22.1 Hz), 128.6, 125.9(t, J = 6.0 Hz), 113.93 (t, J = 240.0 Hz), 26.7.

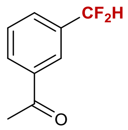

1-(3-(Difluoromethyl)phenyl)ethan-1-one (3p). [known compound[1]]: The product **3p** (24.5 mg, 72% yield) as a colorless oil was purified with silica gel chromatography (Hexane / EtOAc = 50:1). ^{1}H NMR (400 MHz, $CDCl_3$) δ 8.07 (m, 2H), 7.71 (d, *J* = 7.7 Hz, 1H), 7.57 (t, *J* = 7.7 Hz, 1H), 6.70 (t, *J* = 56.2 Hz, 1H), 2.63 (s, 3H). ^{19}F NMR (376 MHz, $CDCl_3$) δ -111.2 (d, *J* = 56.4 Hz, 2F). ^{13}C NMR (101 MHz, $CDCl_3$) δ 197.1, 137.4, 134.9 (t, *J* = 22.7 Hz), 130.4 (t, *J* = 1.7 Hz), 129.9 (t, *J* = 5.7 Hz), 129.1, 125.5 (t, *J* = 6.2 Hz), 114.1 (t, *J* = 240.4 Hz), 26.6.

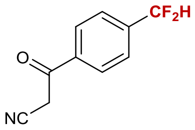

3-(4-(Difluoromethyl)phenyl)-3-oxopropanenitrile (3q). The reaction was carried out with $NiCl_2$ (15 mol%) and **L4** (10 mol%). The product **3q** (13.7 mg, 35% yield) as a orange oil was purified with silica gel chromatography (Hexane / EtOAc = 4:1). ^{1}H NMR (400 MHz, $CDCl_3$) δ 8.01 (d, *J* = 8.0 Hz, 2H), 7.68 (d, *J* = 8.0 Hz, 2H), 6.71 (t, *J* = 55.9 Hz, 1H), 4.12 (s, 2H). ^{19}F NMR (376 MHz, $CDCl_3$) δ -113.1(d, *J* = 56.4 Hz, 2F). ^{13}C NMR (126 MHz, $CDCl_3$) δ 186.6, 140.0 (t, *J* = 22.7 Hz), 135.9 (t, *J* = 1.8 Hz), 128.8, 126.4 (t, *J* = 6.0 Hz), 113.5 (t, *J* = 241.3 Hz), 113.4, 29.6. MS (EI): *m/z* (%) 195 (M^+). HRMS: Calculated for $C_{10}H_7NOF_2$: 195.0496; Found: 195.0499.

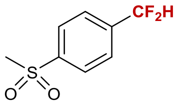

1-(Difluoromethyl)-4-(methylsulfonyl)benzene (3r). The reaction was carried out with $NiCl_2$ (15 mol%) and **L4** (10 mol%). The product **3r** (20.6 mg, 50% yield) as a white soild (m.p. 49 ~ 52 ℃) was purified with silica gel chromatography (Hexane / EtOAc = 5:1). ^{1}H NMR (400 MHz, $CDCl_3$) δ 8.03 (d, *J* = 7.7 Hz, 2H), 7.72 (d, *J* = 7.7 Hz, 2H), 6.72 (t, *J* = 55.8 Hz, 1H), 3.06 (s, 3H). ^{19}F NMR (376 MHz, $CDCl_3$) δ -112.9 (d, *J* = 56.4 Hz, 2F). ^{13}C NMR (126 MHz, $CDCl_3$) δ 142.7 (t, *J* = 1.9 Hz), 139.4 (t, *J* = 22.7 Hz), 127.9, 126.7 (t, *J* = 6.0 Hz), 113.3 (t, *J* = 241.3 Hz), 44.3. MS (EI): *m/z* (%) 206 (M^+). HRMS: Calculated for $C_8H_8SO_2F_2$: 206.0213; Found: 206.0214.

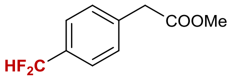

Methyl 2-(4-(difluoromethyl)phenyl)acetate (3s). The product **3s** (32.0 mg, 80% yield) as a colorless oil was purified with silica gel chromatography (Hexane / EtOAc = 50:1). ^{1}H NMR (400 MHz, $CDCl_3$) δ 7.47 (d, *J* = 7.8 Hz, 2H), 7.37 (d, *J* = 7.8 Hz, 2H), 6.63 (t, *J* = 56.5 Hz, 1H), 3.70 (s, 3H), 3.67 (s, 2H). ^{19}F NMR (376 MHz, $CDCl_3$) δ -110.2 (d, *J* = 56.4 Hz, 2F). ^{13}C NMR (101 MHz, $CDCl_3$) δ 171.4, 136.7 (t, *J* = 2.0 Hz), 133.3 (t, *J* = 22.7Hz), 129.6 125.8 (t, *J* = 6.0 Hz), 114.6 (t, *J* = 239.9 Hz), 52.1, 40.9. MS (EI): *m/z* (%) 200 (M^+). HRMS: Calculated for $C_{10}H_{10}O_2F_2$: 200.0649; Found: 200.0654.

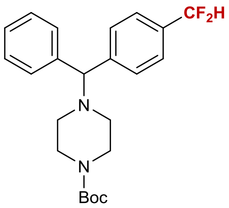

***tert*-Butyl 4-((4-(difluoromethyl)phenyl)(phenyl)methyl)piperazine-1-carboxylate (3t).** The product **3t** (69.1 mg, 86% yield) as a white soild (m.p. 120 ~ 123 ℃) was purified with silica gel chromatography (Hexane / EtOAc = 10:1). ^{1}H NMR (400 MHz, $CDCl_3$) δ 7.51 (d, *J* = 7.8 Hz, 2H), 7.39 (m, 4H), 7.27 (t, *J* = 7.5 Hz, 2H), 7.18 (m, 1H), 6.56 (t, *J* = 56.5 Hz, 1H), 4.26 (s, 1H), 3.42 (s, 4H), 2.32 (s, 4H), 1.42 (s, 9H). ^{19}F NMR (376 MHz, $CDCl_3$) δ -110.3 (d, *J* = 56.4 Hz, 2F). ^{13}C NMR (101 MHz, $CDCl_3$) δ 154.7, 145.4 (t, *J* = 1.9 Hz), 141.6, 133.1 (t, *J* = 22.7 Hz), 128.7, 128.1, 127.9, 127.3, 125.8 (t, *J* = 6.0 Hz), 114.6 (t, *J* = 239.9 Hz), 79.5, 75.7, 51.7, 43.9, 28.4. MS (DART): m/z (%) 403.2 ($[M+H]^+$). HRMS calcd. for $C_{23}H_{28}O_2F_2N_2$ ($[M+H]^+$): 403.2192; Found: 403.2185.

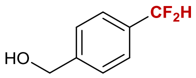

(4-(Difluoromethyl)phenyl)methanol (3u). [known compound[1]]: The reaction was carried out with $NiCl_2$ (15 mol%) and **L4** (10 mol%). The product **3u** (20.5 mg, 65% yield; 76% yield determined by ^{19}F NMR) as a colorless oil was purified with silica gel chromatography (Hexane / EtOAc = 10:1). ^{1}H NMR (400 MHz, $CDCl_3$) δ 7.50 (d, *J* = 8.1 Hz, 2H), 7.44 (d, *J* = 8.1 Hz, 2H), 6.64 (t, *J* = 56.5 Hz, 1H), 4.73 (s, 2H), 2.03 (s, 1H). ^{19}F NMR (376 MHz, $CDCl_3$) δ -110.4 (d, *J* = 56.4 Hz, 2F). ^{13}C NMR (101 MHz, $CDCl_3$) δ 143.5, 133.6 (t, *J* = 22.2 Hz), 126.9, 125.8 (t, *J* = 6.0 Hz), 114.6 (t, *J* = 239.4 Hz), 64.6.

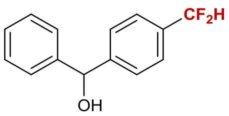

(4-(Difluoromethyl)phenyl)(phenyl)methanol (3v). The product **3v** (33.7 mg, 72% yield) as a colorless oil was purified with silica gel chromatography (Hexane / EtOAc = 10:1). ^{1}H NMR (400 MHz, $CDCl_3$) δ 7.46 (s, 4H), 7.34 (m, 4H), 7.27 (m, 1H), 6.61 (t, *J* = 56.5 Hz, 1H), 5.84 (s, 1H), 2.43 (m, 1H). ^{19}F NMR (376 MHz, $CDCl_3$) δ -110.4 (dd, *J* = 56.4, 3.8 Hz, 2F). ^{13}C NMR (101 MHz, $CDCl_3$) δ 146.4, 143.3, 133.5 (t, *J* = 22.7 Hz), 128.6, 127.9, 126.7, 126.6, 125.7 (t, *J* = 6.0 Hz), 114.6 (t, *J* = 239.4 Hz), 75.8. MS (EI): *m/z* (%) 234 (M^+). HRMS: Calculated for $C_{14}H_{12}OF_2$: 234.0856; Found: 234.0854.

10-Gram Scale Synthesis: To a 350 mL of reaction vessel were added aryl chloride **2v** (10 g, 46 mmol for reaction with 6 equiv of $ClCF_2H$ or 10.9 g, 50 mmol for reaction with 2 equiv of $ClCF_2H$), $NiCl_2$ (10 mol %), **L4** (5 mol %), zinc dust (3.0 equiv), $MgCl_2$ (4.0 equiv), 3 Å MS (12 g) and DMAP (20 mol %) under Ar. DMA (150 mL) and $ClCF_2H$ **1** (2.6 M in DMA, 278 mmol, 6 equiv or 100 mmol, 2 equiv) were added subsequently. The vessel was screw capped and put into a preheated oil bath (60 °C). After stirring for 36 h, the reaction mixture was cooled to room temperature and diluted with ethyl acetate (100 mL). The yield was determined by ^{19}F NMR using fluorobenzene as an internal standard before working up. Then the solvent was pumnped off. The residue was purified with silica gel chromatography (Hexane/EtOAc = 10/1) to give product **3v** (9.7 g, 90% yield for reaction with 6 equiv of $ClCF_2H$ or 9.6 g, 82% yield for reaction with 2 equiv of $ClCF_2H$) as a colorless oil.

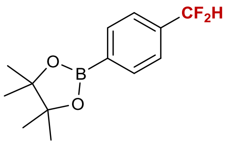

2-(4-(Difluoromethyl)phenyl)-4,4,5,5-tetramethyl-1,3,2-dioxaborolane (3w). The reaction was carried out with $NiCl_2$ (20 mol%), **L4** (10 mol%). The product **3w** (30.5 mg, 60% yield, 78% yield determined by ^{19}F NMR) as a colorless oil was purified with silica gel chromatography (Hexane / EtOAc = 100:1). ^{1}H NMR (400 MHz, $CDCl_3$) δ 7.89 (d, J = 7.7 Hz, 2H), 7.50 (d, J = 7.7 Hz, 2H), 6.65 (t, J = 56.4 Hz, 1H), 1.36 (s, J = 1.8 Hz, 13H). ^{19}F NMR (282 MHz, $CDCl_3$) δ-111.51 (d, *J* = 56.4 Hz. 2F).. ^{13}C NMR (101 MHz, $CDCl_3$) δ 136.78 (t, *J* = 22.2 Hz), 134.99, 124.68 (t, *J* = 6.0 Hz),

114.63 (t, J = 238.9 Hz), 84.08 , 24.82. MS (EI): m/z (%) 254 (M^+), 239 (100). HRMS: Calculated for $C_{13}H_{16}{}^{10}BO_2F_2$ (M-H^+): 252.1248; Found: 252.1247.

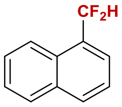

1-(Difluoromethyl)naphthalene (3x). [known compound[1]]: The product **3x** (24.2 mg, 68% yield) as a colorless oil was purified with silica gel chromatography (Hexane). ^{1}H NMR (400 MHz, $CDCl_3$) δ 8.20 (d, J = 8.2 Hz, 1H), 7.98 (d, J = 8.2 Hz, 1H), 7.93 (m, 1H), 7.71 (d, J = 7.1 Hz, 1H), 7.61 (m, 2H), 7.52 (t, J = 7.7 Hz, 1H), 7.16 (t, J = 54.0 Hz, 1H). ^{19}F NMR (376 MHz, $CDCl_3$) δ -110.9 (d, J = 56.4 Hz, 2F). ^{13}C NMR (101 MHz, $CDCl_3$) δ 133.7, 131.5 (t, J = 1.7 Hz), 129.7, 129.5 (t, J = 20.7 Hz), 128.7, 127.1, 126.3, 124.8 (t, J = 9.1 Hz), 124.6, 123.5 (t, J = 1.2 Hz), 115.0 (t, J = 239.4 Hz).

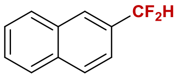

2-(Difluoromethyl)naphthalene (3y). [known compound[1]]: The product **3y** (25.0 mg, 70% yield) as a colorless oil was purified with silica gel chromatography (Hexane). ^{1}H NMR (400 MHz, $CDCl_3$) δ 7.99 (s, 1H), 7.92 (m, 3H), 7.58 (m, 3H), 6.82 (t, J = 56.4 Hz, 1H). ^{19}F NMR (376 MHz, $CDCl_3$) δ -109.8 (d, J = 56.4 Hz, 2F). ^{13}C NMR (101 MHz, $CDCl_3$) δ 134.3 (t, J = 1.3 Hz), 132.5, 131.6 (t, J = 22.2 Hz), 128.9, 128.5, 127.9, 127.4, 126.8, 125.9 (t, J = 7.5 Hz), 122.0 (t, J = 4.8 Hz), 115.0 (t, J = 239.9 Hz).

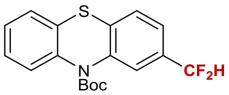

***tert*-Butyl 2-(difluoromethyl)-10*H*-phenothiazine-10-carboxylate (5a).** [known compound[6]]: The reaction was carried out with $NiCl_2$ (15 mol%) and **L4** (10 mol%). The product **5a** (41.9 mg, 60% yield) as a white soild was purified with silica gel chromatography (Hexane / EtOAc = 20:1). ^{1}H NMR (400 MHz, $CDCl_3$) δ 7.67 (s, 1H), 7.52 (d, J = 8.0 Hz, 1H), 7.39 (d, J = 8.0 Hz, 1H), 7.29 (m, 3H), 7.16 (t, J = 7.5 Hz, 1H), 6.63 (t, J = 56.4 Hz, 1H), 1.48 (s, 9H). ^{19}F NMR (376 MHz, $CDCl_3$) δ -110.5 (d, J = 56.4 Hz, 2F). ^{13}C NMR (126 MHz, $CDCl_3$) δ 152.1, 138.9, 138.2, 135.3 (t, J = 2.1 Hz),

132.9 (t, J = 22.7 Hz), 131.2, 127.6, 127.4, 127.2, 126.9, 126.3, 124.5 (t, J = 6.2 Hz), 123.0 (t, J = 6.0 Hz), 114.1 (t, J = 239.4 Hz), 82.6, 28.1.

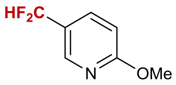

5-(Difluoromethyl)-2-methoxypyridine (5b). The reaction was carried out with $NiCl_2$ (15 mol%) and **L4** (10 mol%). The product **5b** (18.4 mg, 58% yield, 65% determined by ^{19}F NMR) as a colorless oil was purified with silica gel chromatography (Hexane / EtOAc = 20:1). ^{1}H NMR (400 MHz, $CDCl_3$) δ 8.28 (s, 1H), 7.72 (d, J = 8.4 Hz, 1H), 6.82 (d, J = 8.5 Hz, 1H), 6.64 (t, J = 56.0 Hz, 1H), 3.97 (s, 3H). ^{19}F NMR (376 MHz, $CDCl_3$) δ -109.6 (d, J = 56.4 Hz, 2F). ^{13}C NMR (126 MHz, $CDCl_3$) δ 165.7 (t, J = 1.6 Hz), 145.2 (t, J = 7.5 Hz), 135.8 (t, J = 4.5 Hz), 123.4 (J = 23.2 Hz), 113.8 (t, J = 238.4 Hz), 111.4, 53.8. MS (EI): *m/z* (%) 159 (M^+). HRMS: Calculated for $C_7H_7ONF_2$: 159.0496; Found: 159.0497.

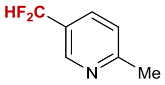

5-(Difluoromethyl)-2-methylpyridine (5c). The reaction was carried out with $NiCl_2$ (15 mol%) and **L4** (10 mol%). Due to the low boiling point of the product **5c**, the yield (34%) was determined by ^{19}F NMR using fluorobenzene as an internal standard. The product was characterized by ^{19}F NMR and GC-MS analysis. ^{19}F NMR (376 MHz) δ -112.18 (d, J = 55.6 Hz, 2F). GC-MS (EI): *m/z* (%) 143.1 (M^+).

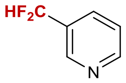

3-(Difluoromethyl)pyridine (5d). [known compound[7]]: The reaction was carried out with $NiCl_2$ (15 mol%) and **L4** (10 mol%). Due to the low boiling point of the product **5d**, the yield (35%) was determined by ^{19}F NMR using fluorobenzene as an internal standard. The product was characterized by ^{19}F NMR and GC-MS analysis. ^{19}F NMR (376 MHz) δ -113.00 (d, J = 55.4 Hz, 2F). GC-MS (EI): *m/z* (%) 129.0 (M^+).

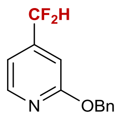

2-(Benzyloxy)-4-(difluoromethyl)pyridine (5e). The reaction was carried out with $NiCl_2$ (15 mol%) and **L4** (10 mol%). The product **5e** (23.5 mg, 50% yield) as a colorless oil was purified with silica gel chromatography (Hexane / EtOAc = 40:1). ^{1}H NMR (400 MHz, $CDCl_3$) δ 8.28 (d, *J* = 5.3 Hz, 1H), 7.46 (d, *J* = 7.1 Hz, 2H), 7.42 – 7.31 (m, 3H), 7.01 (d, *J* = 5.2 Hz, 1H), 6.93 (s, 1H), 6.57 (t, *J* = 55.8 Hz, 1H), 5.42 (s, 2H). ^{19}F NMR (376 MHz, $CDCl_3$) δ -115.65 (d, *J* = 55.7 Hz, 2F). ^{13}C NMR (101 MHz, $CDCl_3$) δ 163.93, 147.84, 144.96 (t, *J* = 23.2 Hz), 136.85, 128.48, 127.96, 113.00 (t, *J* = 5.3 Hz), 112.82 (t, *J* = 240.8 Hz), 108.16 (t, *J* = 6.8 Hz), 67.98. MS (EI): *m/z* (%) 235 (M^+), 91 (100) HRMS: Calculated for $C_{13}H_{11}F_2NO$: 235.0809; Found: 235.0807.

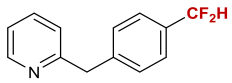

2-(4-(Difluoromethyl)benzyl)pyridine (5f). The reaction was carried out with $NiCl_2$ (15 mol%) and **L4** (10 mol%). The product **5f** (32.7 mg, 75% yield) as a colorless oil was purified with silica gel chromatography (Hexane / EtOAc = 4:1). ^{1}H NMR (400 MHz, $CDCl_3$) δ 8.55 (s, 1H), 7.58 (t, *J* = 7.4 Hz, 1H), 7.43 (d, *J* = 7.6 Hz, 2H), 7.34 (d, *J* = 7.6 Hz, 2H), 7.11 (m, 2H), 6.60 (t, *J* = 56.5 Hz, 1H), 4.18 (s, 2H). ^{19}F NMR (376 MHz, $CDCl_3$) δ -110.1 (d, *J* = 56.4 Hz, 2F). ^{13}C NMR (126 MHz, $CDCl_3$) δ 160.1, 149.4, 142.3(t, *J* = 2.5 Hz), 136.6, 132.5 (t, *J* = 22.7 Hz), 129.3, 125.7 (t, *J* = 6.0 Hz), 123.1, 121.4, 114.7 (t, *J* = 239.4 Hz), 44.4. MS (EI): *m/z* (%) 218 (M^+). HRMS: Calculated for $C_{13}H_{10}NF_2$: 218.0781; Found: 218.0782.

3 mmol-Scale Synthesis by Using 2.0 equiv of $ClCF_2H$: To a 25 mL of Schlenk tube were added $NiCl_2$ (15 mol %), **L4** (10 mol %), zinc dust (3.0 equiv), $MgCl_2$ (4.0 equiv), 3Å MS (700 mg) and DMAP (20 mol %). The mixture was evacuated and backfilled with argon for three times, aryl chloride **4f** (3 mmol), DMA (8 mL) and $ClCF_2H$ **1** (2.6 M in DMA, 6 mmol, 2.0 equiv) were then added. The Schlenk tube was screw capped and put into a preheated oil bath (60 °C). After stirring for 20 h, the reaction mixture was cooled to room temperature and diluted with ethyl acetate (10 mL). The reaction mixture was filtered with a pad of cellite. The filtrate was washed with brine, extracted with EtOAc for three times. The combined organic layers were dried over Na_2SO_4 and concentrated.

The residue was purified with silica gel chromatography to give product **5f** (395 mg, 60% yield) as a colorless oil.

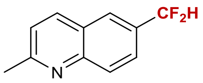

6-(Difluoromethyl)-2-methylquinoline (5g). [known compound[6]]: The reaction was carried out with $NiCl_2$ (15 mol%) and **L4** (10 mol%). The product **5g** (27.0 mg, 70% yield) as a white soild was purified with silica gel chromatography (Hexane / EtOAc = 10:1). ^{1}H NMR (400 MHz, $CDCl_3$) δ 8.07 (t, *J* = 8.5 Hz, 8H), 7.89 (s, 4H), 7.77 (d, *J* = 8.7 Hz, 4H), 7.30 (t, *J* = 14.2 Hz, 5H), 6.79 (t, *J* = 56.0 Hz,), 2.75 (s, 12H). ^{19}F NMR (376 MHz, $CDCl_3$) δ -112.2 (d, *J* = 56.4 Hz, 2F). ^{13}C NMR (101 MHz, $CDCl_3$) δ 160.7, 148.6, 136.5, 131.4 (t, *J* = 22.7 Hz), 129.6, 125.68 (t, *J* = 4.5 Hz), 125.69, 125.4 (t, *J* = 7.1 Hz), 122.8, 114.5 (t, *J* = 239.9 Hz), 25.4.

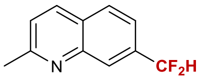

7-(Difluoromethyl)-2-methylquinoline (5h). The reaction was carried out with $NiCl_2$ (15 mol%) and **L4** (10 mol%). The product **5h** (21.2 mg, 55% yield) as a colorless oil was purified with silica gel chromatography (Hexane / EtOAc = 10:1). ^{1}H NMR (400 MHz, $CDCl_3$) δ 8.13 (s, 1H), 8.06 (d, *J* = 8.4 Hz, 1H), 7.85 (d, *J* = 8.4 Hz, 1H), 7.61 (d, *J* = 8.3 Hz, 1H), 7.35 (d, *J* = 8.4 Hz, 1H), 6.81 (t, *J* = 56.2 Hz, 1H), 2.75 (s, 3H). ^{19}F NMR (376 MHz, $CDCl_3$) δ -110.5 (d, *J* = 56.4 Hz, 2F). ^{13}C NMR (101 MHz, $CDCl_3$) δ 160.2, 147.2, 135.9, 135.1 (t, *J* = 22.7 Hz), 128.5, 127.5, 126.7 (t, *J* = 7.4 Hz), 123.3, 121.8 (t, *J* = 4.8 Hz), 114.6 (t, *J* = 239.9 Hz), 25.4. MS (EI): *m/z* (%) 193 (M^+). HRMS: Calculated for $C_{11}H_9OF_2$: 193.0703; Found: 193.0710.

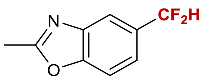

5-(Difluoromethyl)-2-methylbenzo[*d*]oxazole (5i). The reaction was carried out with $NiCl_2$ (15 mol%) and **L4** (10 mol%). The product **5i** (22.7 mg, 62% yield) as a colorless oil was purified with silica gel chromatography (Hexane / EtOAc = 10:1). ^{1}H NMR (400 MHz, $CDCl_3$) δ 7.79 (s, 1H), 7.53 (d, *J* = 8.4 Hz, 1H), 7.45 (d, *J* = 8.3 Hz, 1H), 6.73 (t, *J* = 56.5 Hz, 1H), 2.65 (s, 3H). ^{19}F NMR (376 MHz, $CDCl_3$) δ -110.0 (d, *J* = 56.4 Hz, 2F). ^{13}C NMR (101 MHz, $CDCl_3$) δ 165.2, 152.2 (t, *J* = 1.7 Hz), 141.7), 130.8 (t, *J* = 22.7 Hz), 122.0 (t, *J* = 5.9 Hz), 117.2 (t, *J* = 6.6 Hz), 114.7 (t, *J* = 240.4

Hz), 110.6, 14.5. MS (EI): *m/z* (%) 183 (M^+). HRMS: Calculated for $C_9H_7ONF_2$: 183.0496; Found: 183.0497.

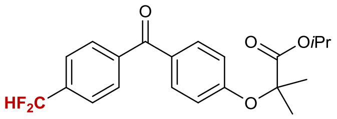

Isopropyl 2-(4-(4-(difluoromethyl)benzoyl)phenoxy)-2-methylpropanoate (7a). [known compound[6]]: The reaction was carried out with $NiBr_2$ (20 mol%) and **L4** (10 mol%) in the absence of 3Å MS. The product **7a** (51.1 mg, 68% yield) as a white soild was purified with silica gel chromatography (Hexane / EtOAc = 10:1). ^{1}H NMR (400 MHz, $CDCl_3$) δ 7.79 (d, *J* = 7.9 Hz, 2H), 7.74 (d, *J* = 8.7 Hz, 2H), 7.60 (d, *J* = 7.9 Hz, 2H), 6.85 (d, *J* = 8.8 Hz, 2H), 6.70 (d, *J* = 56.0 Hz, 1H), 5.07 (hept, *J* = 6.0 Hz, 1H), 1.65 (s, 6H), 1.18 (d, *J* = 6.0 Hz, 6H). ^{19}F NMR (376 MHz, $CDCl_3$) δ -111.8 (d, *J* = 56.4 Hz, 2F). ^{13}C NMR (101 MHz, $CDCl_3$) δ 194.5, 172.9, 159.8, 140.2 (t, *J* = 2.0 Hz), 137.3 (t, *J* = 22.7 Hz), 132.0, 129.9, 129.8, 125.4 (t, *J* = 6.1 Hz), 117.1, 114.0 (t, *J* = 240.4 Hz), 79.3, 69.2, 25.3, 21.4.

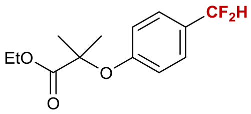

Ethyl 2-(4-(difluoromethyl)phenoxy)-2-methylpropanoate (7b). [known compound[6]]: The product **7b** (37.2 mg, 72% yield) as a colorless oil was purified with silica gel chromatography (Hexane / EtOAc = 10:1). ^{1}H NMR (400 MHz, $CDCl_3$) δ 7.38 (d, *J* = 8.8 Hz, 2H), 6.87 (d, *J* = 8.8 Hz, 2H), 6.58 (t, *J* = 56.0 Hz, 1H), 4.23 (q, *J* = 7.1 Hz, 2H), 1.62 (s, 6H), 1.23 (t, *J* = 7.1 Hz, 3H). ^{19}F NMR (376 MHz, $CDCl_3$) δ -108.9 (d, *J* = 56.4 Hz, 2F). ^{13}C NMR (101 MHz, $CDCl_3$) δ 173.8, 157.4 (t, *J* = 1.8 Hz), 127.7 (t, *J* = 23.2 Hz), 126.7 (t, *J* = 6.0 Hz), 118.4, 114.7 (t, *J* = 238.9 Hz), 79.2, 61.6, 25.3, 14.0.

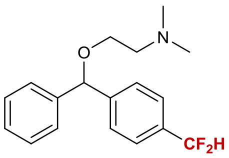

2-((4-(Difluoromethyl)phenyl)(phenyl)methoxy)-*N*,*N*-dimethylethan-1-amine (7c). The product **7c** (44.0 mg, 72% yield) as a colorless oil was purified with silica gel chromatography

(Dichloromethane / EtOAc / Et_3N = 1:5:1%). ^{1}H NMR (400 MHz, $CDCl_3$) δ 7.44 (s, 4H), 7.34 – 7.23 (m, 5H), 6.59 (t, *J* = 56.5 Hz, 1H), 5.39 (s, 1H), 3.56 (td, *J* = 5.9, 1.8 Hz, 2H), 2.60 (t, *J* = 5.9 Hz, 2H), 2.26 (s, 6H). ^{19}F NMR (376 MHz, $CDCl_3$) δ -110.4 (d, *J* = 56.4 Hz, 2F). ^{13}C NMR (101 MHz, $CDCl_3$) δ 145.1, 141.6, 133.4 (t, *J* = 22.2 Hz), 128.5, 127.7, 127.1, 127.0, 125.59 (t, *J* = 6.0 Hz), 114.6 (t, *J* = 239.9 Hz), 83.5, 67.5, 58.9, 45.9. MS (DART): m/z (%) 306.2 ([M+H]$^+$). HRMS calcd. for $C_{18}H_{22}OF_2N$ ([M+H]$^+$): 306.1664; Found: 306.1661.

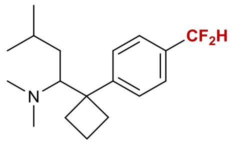

1-(1-(4-(Difluoromethyl)phenyl)cyclobutyl)-*N*,*N*-3-trimethylbutan-1-amine (7d). The product **7d** (40.0 mg, 67% yield) as a colorless oil was purified with silica gel chromatography (Dichloromethane / EtOAc / Et_3N = 1:5:1%). ^{1}H NMR (400 MHz, $CDCl_3$) δ 7.40 (d, *J* = 8.1 Hz, 2H), 7.30 (d, *J* = 8.1 Hz, 2H), 6.64 (t, *J* = 56.7 Hz, 1H), 2.94 (dd, *J* = 10.7, 2.6 Hz, 1H), 2.49 (m, 1H), 2.34 (m, 1H), 2.22 (m, 1H), 2.16 (s, 6H), 2.12 (m, 1H), 1.96 (m, 1H), 1.77 (m, 1H), 1.54 (m, 1H), 1.21 (m, 1H), 1.11 (m, 1H), 0.97 (d, *J* = 8.0 Hz, 3H), 0.88 (d, *J* = 8.0 Hz, 3H). ^{19}F NMR (376 MHz, $CDCl_3$) δ -109.6 (d, *J* = 60.2 Hz, 2F). ^{13}C NMR (126 MHz, $CDCl_3$) δ 150.9 (t, *J* = 1.9 Hz), 131.3 (t, *J* = 22.1 Hz), 128.1, 124.31 (t, *J* = 6.0 Hz), 115.1 (t, *J* = 238.8 Hz), 67.5, 52.1, 44.0, 36.2, 33.2, 32.9, 26.2, 24.1, 21.4, 15.7. MS (DART): m/z (%) 296.2 ([M+H]$^+$). HRMS calcd. for $C_{18}H_{28}F_2N$ ([M+H]$^+$): 296.2184; Found: 296.2181.

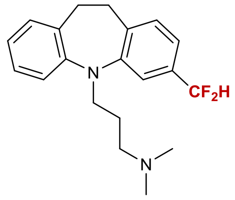

3-(3-(Difluoromethyl)-10,11-dihydro-5*H*-dibenzo[*b*,*f*]azepin-5-yl)-*N*,*N*-dimethylpropan-1-amine (7e). The reaction was carried out with $NiCl_2$ (15 mol%) and **L4** (10 mol%). The product **7e** (44.2 mg, 67% yield) as a colorless oil was purified with silica gel chromatography (Dichloromethane / EtOAc / NEt_3= 1:5:2%). ^{1}H NMR (400 MHz, $CDCl_3$) δ 7.23 (s, 1H), 7.13 (m, 4H), 6.98 (m, 2H), 6.57 (t, *J* = 56.7 Hz, 1H), 3.80 (t, *J* = 6.8 Hz, 2H), 3.17 (s, 4H), 2.31 (t, *J* = 7.1 Hz, 2H), 2.15 (s, 6H), 1.72 (m, 2H). ^{19}F NMR (376 MHz, $CDCl_3$) δ -109.7 (d, *J* = 60.2 Hz, 2F). ^{13}C NMR (101 MHz,

$CDCl_3$) δ 148.5, 147.9, 136.2 (t, J = 2.1 Hz), 134.7, 132.5 (t, J = 22.7 Hz), 130.4, 129.5, 126.5, 123.0, 120.4, 119.1 (t, J = 6.1 Hz), 116.8 (t, J = 5.7 Hz), 114.8 (t, J = 239.4 Hz), 57.5), 48.9), 45.5, 32.5, 31.6, 26.0. MS (DART): m/z (%) 331.2 ($[M+H]^+$). HRMS calcd. for $C_{20}H_{25}F_2N_2$ ($[M+H]^+$): 331.1980; Found: 331.1975.

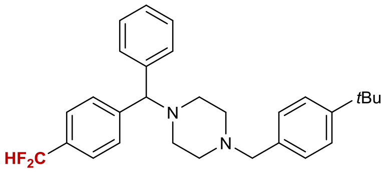

1-(4-(*tert*-Butyl)benzyl)-4-((4-(difluoromethyl)phenyl)(phenyl)methyl)piperazine (7f). The product **7f** (67.3 mg, 75% yield) as a white soild (m.p. 80 ~ 83 ℃) was purified with silica gel chromatography (Hexane / EtOAc = 1:3). ^{1}H NMR (400 MHz, $CDCl_3$) δ 7.54 (d, J = 8.2 Hz, 2H), 7.41 (m, 4H), 7.35 (d, J = 8.2 Hz, 2H), 7.27 (m, 4H), 7.20 (m, 1H), 6.58 (t, J = 56.6 Hz, 1H), 4.31 (s, 1H), 3.52 (s, 2H), 2.50 (s, 8H), 1.33 (s, 9H). ^{19}F NMR (376 MHz, $CDCl_3$) δ -110.1 (d, J = 56.4 Hz, 2F). ^{13}C NMR (126 MHz, $CDCl_3$) δ 149.8, 145.8 (t, J = 1.9 Hz), 142.0, 134.9, 132.9 (t, J = 22.7 Hz), 128.9, 128.5, 128.1, 127.9, 127.1, 125.7 (t, J = 6.0 Hz), 125.0, 114.6 (t, J = 239.4 Hz), 75.8, 62.6, 53.2, 51.8, 34.4, 31.4. MS (DART): m/z (%) 449.3 ($[M+H]^+$). HRMS calcd. for $C_{29}H_{35}F_2N_2$ ($[M+H]^+$): 449.2763; Found: 449.2761.

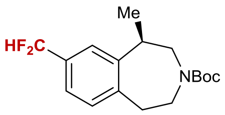

***tert*-Butyl(*R*)-8-(difluoromethyl)-1-methyl-1,2,4,5-tetrahydro-3*H*-benzo[*d*]azepine-3-carboxylate (7g).** The reaction was carried out with $NiCl_2$ (15 mol%) and **L4** (10 mol%). The product **7g** (52.3 mg, 84% yield) as a colorless oil was purified with silica gel chromatography (Hexane / EtOAc = 6:1). ^{1}H NMR (400 MHz, D_6-DMSO, 80 °C) δ 7.34 (s, 1H), 7.31 (d, J = 7.8 Hz, 1H), 7.25 (d, J = 7.8 Hz, 1H), 6.91 (t, J = 56.2 Hz, 1H), 3.69 (m, 1H), 3.54 (dd, J = 13.7, 7.3 Hz, 1H), 3.44 (dd, J = 14.0, 3.8 Hz, 1H), 3.30 (m, 1H), 3.20 (m, 1H), 3.03 (m, 1H), 2.90 (m, 1H), 1.36 (s, 9H), 1.26 (d, J = 7.2 Hz, 3H). ^{19}F NMR (376 MHz, D_6-DMSO, 80 °C) δ -109.9 (d, J = 56.4 Hz, 2F). ^{13}C NMR (101 MHz, D_6-DMSO, 80 °C) δ 154.2, 144.4, 141.6 (t, J = 2.0 Hz), 132.0 (t, J = 22.2 Hz), 130.1, 124.6 (t, J =

6.0 Hz), 122.9 (t, J = 6.0 Hz), 114.6 (t, J = 236.8 Hz), 78.2, 50.1, 45.0, 34.7, 27.6, 17.1. MS (EI): m/z (%) 311 (M^+). HRMS: Calculated for $C_{17}H_{23}NO_2F_2$: 311.1697; Found: 311.1703.

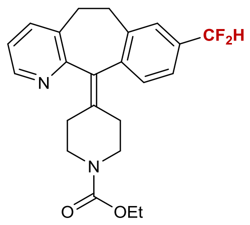

Ethyl4-(8-(difluoromethyl)-5,6-dihydro-11*H*-benzo[5,6]cyclohepta[1,2-*b*]pyridin-11-ylidene)piperidine-1-carboxylate (7h). [known compound[6]]: The product **7h** (65.3 mg, 82% yield) as a white soild was purified with silica gel chromatography (Hexane / EtOAc = 1:1). ^{1}H NMR (400 MHz, $CDCl_3$) δ 8.33 (d, J = 4.0 Hz, 1H), 7.37 (d, J = 7.4 Hz, 1H), 7.22 (m, 3H), 7.02 (dd, J = 7.6, 4.8 Hz, 1H), 6.51 (t, J = 56.0 Hz, 1H), 4.06 (q, J = 7.1 Hz, 2H), 3.74 (s, 2H), 3.34 (m, 2H), 3.08 (m, 2H), 2.79 (m, 2H), 2.43 (m, 1H), 2.27 (m, 3H), 1.17 (t, J = 7.1 Hz, 3H). ^{19}F NMR (376 MHz, $CDCl_3$) δ -110.3 (d, J = 56.4 Hz, 2F). ^{13}C NMR (101 MHz, $CDCl_3$) δ 156.7, 155.4, 146.6, 141.9, 138.3, 137.60, 137.56, 134.4, 133.3 (t, J = 22.7 Hz), 129.5, 126.1 (t, J = 5.8 Hz), 123.3 (t, J = 6.0 Hz), 122.3, 114.6 (t, J = 239.9 Hz), 61.3, 44.7, 31.7, 31.5, 30.7, 30.5, 14.6.

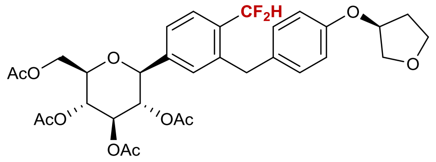

(2*R*, 3*R*, 4*R*, 5*S*, 6*S*)-2-(Acetoxymethyl)-6-(4-(difluoromethyl)-3-(4-(((*S*)-tetrahydrofuran-3-yl)oxy)benzyl)phenyl)tetrahydro-2*H*-pyran-3,4,5-triyl triacetate (7i). The reaction was carried out with $NiCl_2$ (20 mol%) and **L4** (10 mol%). The product **7i** (25% yield determined by ^{19}F NMR) as a white soild (125 ~ 128 ℃) was purified through the reverse phase preparative HPLC (MeOH/H_2O = 50:50 (v/v); Column: Nova-Pak HR C18 (19 mm × 300 mm, 6 μm); Flow rate of the Mobile phase: 18 mL/min; Wavelength: 230 nm; Temperature: 25 °C). ^{1}H NMR (500 MHz, $CDCl_3$) δ 7.55 (d, J = 8.1 Hz, 1H), 7.33 (d, J = 7.4 Hz, 1H), 7.14 (s, 1H), 6.99 (d, J = 8.6 Hz, 2H), 6.78 (d, J = 8.6 Hz, 2H), 6.66 (t, J = 55.0 Hz, 1H), 5.30 (t, J = 9.4 Hz, 1H), 5.21 (t, J = 9.7 Hz, 1H), 5.07 (t, J = 9.7 Hz, 1H), 4.88 (m, 1H), 4.38 (d, J = 9.8 Hz, 1H), 4.27 (m, 1H), 4.15 (m, 1H), 4.06 (d, J = 6.0 Hz, 2H), 3.96 (m, 3H), 3.88 (m,

1H), 3.81 (m, 1H), 2.15 (m, 2H), 2.07 (s, 3H), 2.05 (s, 3H), 1.99 (s, 3H), 1.72 (s, 3H). ^{19}F NMR (376 MHz, $CDCl_3$) δ -111.8 (dd, *J* = 300.8, 56.4 Hz, 1F), δ -112.8 (dd, *J* = 300.8, 56.4 Hz, 1F). ^{13}C NMR (126 MHz, $CDCl_3$) δ 170.7, 170.3, 169.5, 168.7, 156.0, 139.2 (t, *J* = 4.4 Hz), 139.0 (t, *J* = 2.5 Hz), 132.8 (t, *J* = 20.2 Hz), 131.6, 129.8, 129.7, 126.3 (t, *J* = 7.4 Hz), 125.2, 115.5, 113.5 (t, *J* = 239.4 Hz), 79.5, 77.3, 76.1, 74.1, 73.1, 72.5, 68.4, 67.1, 62.2. 36.9, 32.9, 20.7, 20.6, 20.3. MS (DART): m/z (%) 635.2 ([M+H]$^+$). HRMS calcd. for $C_{32}H_{37}O_{11}F_2$ ([M+H]$^+$): 635.2298; Found: 635.2289.

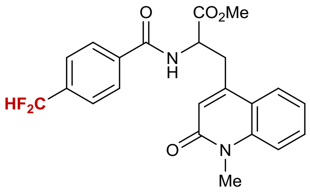

Methyl 2-(4-(difluoromethyl)benzamido)-3-(1-methyl-2-oxo-1,2-dihydroquinolin-4-yl)propanoate (7j). The reaction was carried out with $NiBr_2$ (20 mol%), **L4** (10 mol%) and without 3 Å MS. The product **7j** (63.0 mg, 76% yield) as a white soild (175 ~ 178 ℃) was purified with silica gel chromatography (Hexane / EtOAc = 1:2). ^{1}H NMR (400 MHz, *D*$_6$-DMSO) δ 9.12 (d, *J* = 7.4 Hz, 1H), 7.91 (d, *J* = 7.2 Hz, 3H), 7.65 (m, 3H), 7.54 (d, *J* = 7.5 Hz, 1H), 7.33 (t, *J* = 6.9 Hz, 1H), 7.09 (t, *J* = 56.1 Hz, 1H), 6.59 (s, 1H), 4.83 (s, 1H), 3.70 (s, 3H), 3.57 (s, 3H), 3.50 (m, 1H), 3.30 (sm, 1H). ^{19}F NMR (376 MHz, *D*$_6$-DMSO) δ -110.8 (d, *J* = 56.4 Hz, 2F). ^{13}C NMR (101 MHz, *D*$_6$-DMSO) δ 171.5, 165.8, 160.5, 145.6, 139.8, 136.8 (t, *J* = 22.2 Hz), 135.7, 130.8, 127.9, 127.3, 125.8 (t, *J* = 6.0 Hz), 124.6, 122.1, 121.2, 119.5, 115.3, 114.4 (t, *J* = 237.9 Hz), 52.4, 52.0, 32.5, 28.9. MS (DART): m/z (%) 415.1 ([M+H]$^+$). HRMS calcd. for $C_{22}H_{21}O_4F_2N_2$ ([M+H]$^+$): 415.1464; Found: 415.1461.

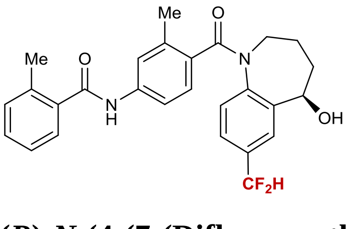

(*R*)-*N*-(4-(7-(Difluoromethyl)-5-hydroxy-2,3,4,5-tetrahydro-1*H*-benzo[*b*]azepine-1-carbonyl)phenyl)-2-methylbenzamide (7k). The product **7k** (85.4 mg, 92% yield) as a white soild (m.p. 60 ~ 63 ℃) was purified with silica gel chromatography (Hexane / EtOAc = 1:2). ^{1}H NMR (400 MHz, *D*$_6$-DMSO) rotameric mixture: δ 10.39, 10.21 and 10.17 (s, 1H, rotamer), 7.94 – 6.60 (m, 11H), 5.69 (m, 1H), 4.81 (m, 2H), 2.66 (t, *J* = 11.8 Hz, 1H), 2.34 (m, 6H), 2.12 (d, *J* = 11.3 Hz, 1H), 1.94 (m,

1H), 1.57 (m, 2H). ^{19}F NMR (376 MHz, D_6-DMSO) δ -109.4 (m, 2F). ^{13}C NMR (101 MHz, D_6-DMSO) δ 168.3, 168.0, 143.3, 142.1, 139.5, 137.0, 136.2, 135.3, 132.4 (t, J = 22.7 Hz), 131.5, 130.6, 129.7, 128.0, 127.2, 126.6, 125.6, 124.5, 122.7, 120.9, 115.9 (t, J = 236.3 Hz), 112.5, 69.7, 45.8, 35.4, 25.8, 19.7, 19.3. MS (DART): m/z (%) 465.2 ($[M+H]^+$). HRMS calcd. for $C_{27}H_{27}O_3F_2N_2$ ($[M+H]^+$): 465.1984; Found: 465.1980.

3 mmol-Scale Synthesis by Using 2.0 equiv of $ClCF_2H$: To a 25 mL of Schlenk tube were added $NiCl_2$ (10 mol %), **L4** (5 mol %), zinc dust (3.0 equiv), $MgCl_2$ (4.0 equiv), 3Å MS (700 mg) and DMAP (20 mol %). The mixture was evacuated and backfilled with argon for three times, aryl chloride **6k** (3 mmol), DMA (8 mL) and $ClCF_2H$ **1** (2.6 M in DMA, 6 mmol, 2.0 equiv) were then added. The Schlenk tube was screw capped and put into a preheated oil bath (60 °C). After stirring for 20 h, the reaction mixture was cooled to room temperature and diluted with ethyl acetate (10 mL). Tthe reaction mixture was filtered with a pad of cellite. The filtrate was washed with brine, extracted with EtOAc for three times. The combined organic layers were dried over Na_2SO_4 and concentrated. The residue was purified with silica gel chromatography to give product **7k** (1.25 g, 90%) as a solid.

10-Gram Scale Synthesis: To a 350 mL of reaction vessel were added aryl chloride **6k** (11.2 g, 25 mmol, 1.0 equiv), $NiCl_2$ (10 mol %), **L4** (5 mol %), zinc dust (3.0 equiv), $MgCl_2$ (4.0 equiv), 3 Å MS (6 g) and DMAP (20 mol %) under Ar. DMA (150 mL) and $ClCF_2H$ **1** (2.6 M in DMA, 150 mmol, 6 equiv) were added subsequently. The vessel was screw capped and put into a preheated oil bath (60 °C). After stirring for 36 h, the reaction mixture was cooled to room temperature and diluted with ethyl acetate (100 mL). The yield was determined by ^{19}F NMR using fluorobenzene as an internal standard before working up. Then the solvent was pumnped off. The residue was purified with silica gel chromatography (Hexane/EtOAc = 2/1) to give product **7k** (10.5 g, 91%) as a solid.

Reaction of $ClCF_2H$ with Zn

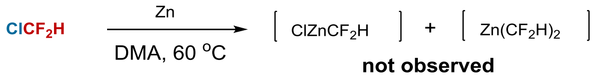

Procedure: Acid-washed, fresh zinc dust (0.3 mmol, 1.0 equiv) was added to a 25 mL of Schlenk tube, which was then evacuated and backfilled with argon (3 times). $ClCF_2H$ (2.6 M in DMA, 0.3 mmol, 1.0 equiv) and DMA (2.0 mL) were added subsequently. The Schlenk tube was screw capped and put into a preheated oil bath (60 °C). After stirring for 3 h, the reaction mixture was cooled to

room temperature. ^{19}F NMR showed no difluoromethylzinc species were formed and only $ClCF_2H$ were observed.

Reaction of $ClCF_2H$ under Standard Reaction Conditions

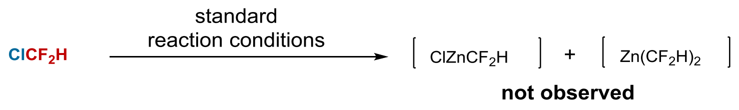

Procedure: To a 25 mL of Schlenk tube were added $NiCl_2$ (10 mol %), **L4** (5 mol %), zinc dust (3.0 equiv), $MgCl_2$ (4.0 equiv), 3 Å MS (100 mg) and DMAP (20 mol %). The mixture was evacuated and backfilled with argon for three times, DMA (1.5 mL) and $ClCF_2H$ **1** (2.6 M in DMA, 0.3 mmol, 1.0 equiv) were added subsequently. The Schlenk tube was screw capped and put into a preheated oil bath (60 °C). After the reaction was stirred for 6 h, ^{19}F NMR showed no difluoromethylzinc species were formed and only $ClCF_2H$ were observed.

Reaction of Difluoromethylzinc Reagents with 2c

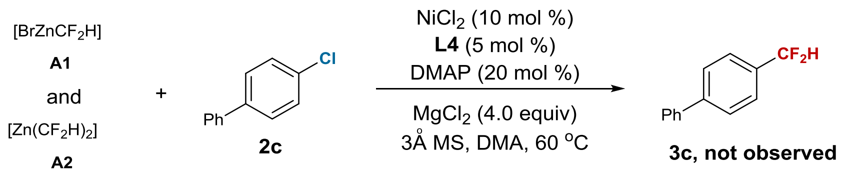

Procedure: Acid-washed, fresh zinc dust (0.6 mmol, 2.0 equiv) was added to a 25 mL of Schlenk tube, which was then evacuated and backfilled with argon (3 times). $BrCF_2H$ (5.2 M in DMA, 0.3 mmol, 1.0 equiv) and DMA (1.5 mL) were added subsequently. The Schlenk tube was screw capped and put into a preheated oil bath (60 °C). After stirring for 8 h, the reaction mixture was cooled to room temperature. ^{19}F NMR showed both mono- and bis-difluoromethylzinc reagents **A1** and **A2** were formed (**A1**/**A2** = 1:3) and their chemical shifts were in accordance with the literature.[8] **A1**: ^{19}F NMR (376 MHz, $CDCl_3$) δ -126.7 (d, J = 45.1 Hz); **A2**: ^{19}F NMR (376 MHz, $CDCl_3$) δ -127.8 (d, J = 41.4 Hz).

To a 25 mL of Schlenk tube were added **2c** (1.0 equiv), $NiCl_2$ (10 mol %), **L4** (5 mol %), $MgCl_2$ (4.0 equiv), 3 Å MS (100 mg) and DMAP (20 mol %). The mixture was evacuated and backfilled with argon for three times, then DMA (1.0 mL) was added subsequently. The difluoromethylzinc reagents **A1** and **A2** were added dropwise to the reaction mixture with stirring. Then the Schlenk

tube was screw capped and put into a preheated oil bath (60 °C). After stirring for 6 h, the reaction mixture was cooled to room temperature and worked up with ethyl acetate. ^{19}F NMR showed no desired product **3c** was formed.

Preparation of Nickel Complex B1

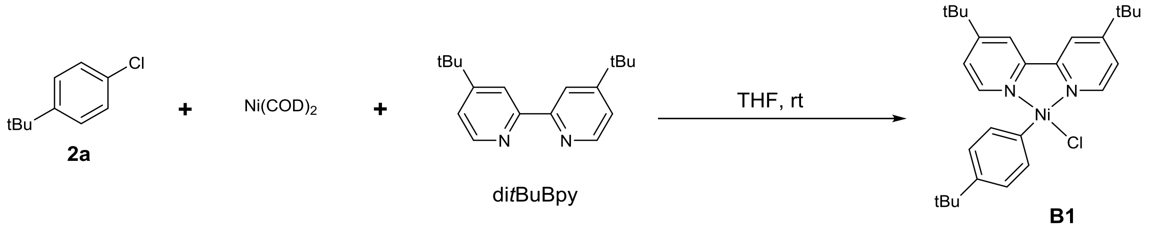

Procedure: The preparation of nickel complex (**B1**) is a modification of the literature method.[9] To a Schlenk tube was charged with Ni(COD)$_2$ (550 mg, 2.0 mmol), 4,4′-di-*tert*-butyl-2,2′- pyridine (537 mg, 2.0 mmol) and degassed THF (5 mL) under Ar. The resulting deep purple solution was left to stir for 1 hour at ambient temperature. Aryl chloride **2a** (8.6 mL, 51.5 mmol) was then added and the reaction mixture was stirred for 20 min. The resulting dark red solution was triturated with pentane and the precipitate was collected on a frit, rinsed with pentane and dried under vacuum to give nickel complex (**B1**) as a light red powder (705 mg, 71% yield). The nickel complex was used in stoichiometric experiments without further purification. **NOTE:** Complex **B1** is unstable in solution, which is prone to decomposition (**B1** slowly deterorated during its recording by ^{13}C NMR).

^{1}H NMR (400 MHz, CH_2Cl_2-d_2) δ 9.03 (s, 1H), 7.85 (s, 1H), 7.82 (s, 1H), 7.52 (s, 1H), 7.42 – 7.34 (m, 3H), 7.15 (s, 1H), 7.03 (s, 2H), 1.42 (s, 9H), 1.35 (s, 9H), 1.31 (s, 10H). ^{13}C NMR (101 MHz, CH_2Cl_2-d_2) δ 146.83, 136.56, 126.35, 123.35, 32.23, 29.22.

SAESI-MS Analysis of Nickel Complex B1

SAESI-MS spectra were recorded on a Finnigan TSQ (Thermo Finnigan, Quantum Access TM) triple-quadrupole mass spectrometer equipped with a home-made SAESI ion source in positive mode. The basic SAESI conditions were: vacuum, 2.8×10^{-6} torr; spray voltage, 3000 V; capillary temperature, 300°C; sheath gas pressure of two sprayers, 10 arb. units; the collision energy of CID, 10~30 eV. Data acquisition and analysis were done with the Xcalibur (version 2.0, Thermoquest Finnigan) software package.

Procedure: The chemical solution of **B1** in CH_2Cl_2 was injected by a 500-μL air-tight syringe with a speed at 5 μL/min to SAESI-MS. The assisted solvent of methanol was injected by another 500-μL air-tight syringe with a speed at 10 μL/min to SAESI-MS. (**B1** deterorated during its recording by SAESI-MS).

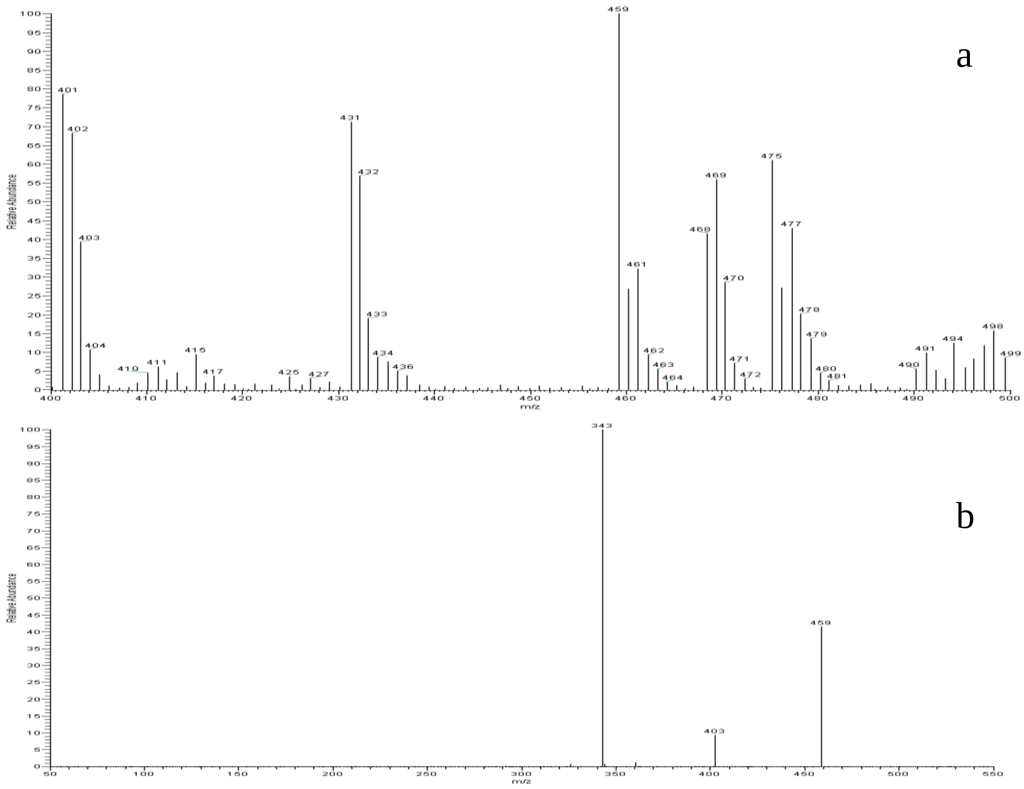

Supplementary Figure 140. a SAESI-MS spectrum of nickel complex **B1**, showing the complex ion $[M-Cl]^+$ at *m/z* 459. **b** The SAESI-MS/MS spectra of the ion at *m/z* 459.

Reaction of Nickel Complex B1 with $ClCF_2H$

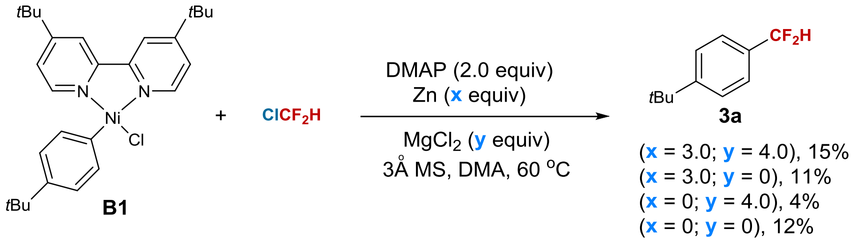

Procedure: To a 25 mL of Schlenk tube were added nickel complex **B1** (0.2 mmol, 1.0 equiv), Zn dust (3.0 equiv or 0 equiv), $MgCl_2$ (4.0 equiv or 0 equiv), 3 Å MS (100 mg) and DMAP (0.4 mmol, 2 equiv). The mixture was then evacuated and backfilled with Ar (3 times). DMA (2 mL) and $ClCF_2H$ (2.6 M in DMA, 1.3 mmol, 6.5 equiv) were then added. The Schlenk tube was screw capped and put into a preheated oil bath (60 °C). After stirring for 8 h, the reaction mixture was cooled to room

temperature, diluted with ethyl acetate. The yield was determined by ^{19}F NMR using fluorobenzene as an internal standard. (The yields are based on the average of two runs under identical conditions).

Nickel complex B1 or NiCl2/di*t*BuBpy Catalyzed Cross-Coupling of Aryl Chloride 2a with ClCF2H

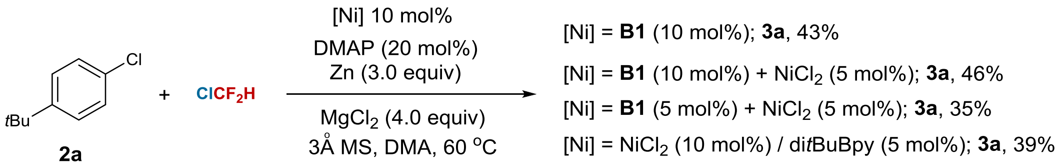

Procedure: To a 25 mL of Schlenk tube were added nickel complex **B1** (0.02 mmol, 10 mol %) or [**B1** (10 mol%) + $NiCl_2$ (5 mol%)] or [**B1** (5 mol%) + $NiCl_2$ (5 mol%)] or [Ni] ($NiCl_2$, 10 mol%; di*t*BuBpy, 5 mol%), Zn dust (3.0 equiv), $MgCl_2$ (4.0 equiv), 3Å MS (100 mg) and DMAP (0.04 mmol, 20 mol %). The mixture was then evacuated and backfilled with Ar (3 times). **2a** (0.2 mmol, 1 equiv), DMA (2 mL) and $ClCF_2H$ (2.6 M in DMA, 1.3 mmol, 6.5 equiv) were then added. The Schlenk tube was screw capped and put into a preheated oil bath (60 °C). After stirring for 20 h, the reaction mixture was cooled to room temperature, diluted with ethyl acetate. The yield was determined by ^{19}F NMR using fluorobenzene as an internal standard.

Preparation of Difluoromethyl Nickel Complex C1

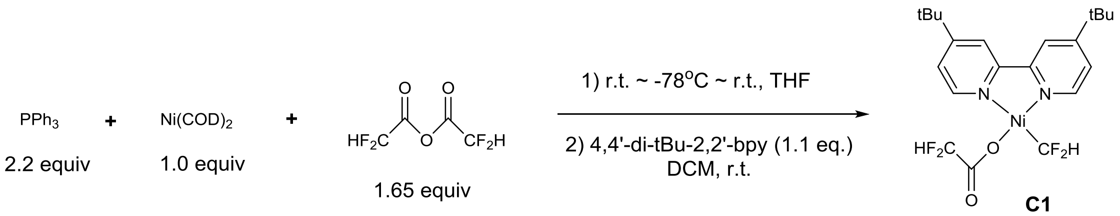

Procedure: The preparation of nickel complex **C1** is a modification of the literature method.[10, 11] To Schlenk tube was added triphenylphosphine (2.30 g, 8.8 mmol) and $Ni(COD)_2$ (1.1 g, 4 mmol) under Ar. Degassed THF (150 mL) was then added, and the resulting dark red mixture was stirred at 25 ℃ for 15 min. The solution was then cooled to −78 ℃, and difluoroacetic anhydride (0.82 mL, 6.6 mmol) was added dropwise. The solution was slowly warmed to room temperature over ~20 min, and volatiles were then removed under reduced pressure. Diethyl ether (100 mL) was added to the residue, and the product precipitated in the form of orange solid. The product was collected via

fltration, washed with several portions of diethyl ether, and dried under vacuum to afford an orange solid. A Schlenk flask was charged with the orange solid (1.46 g, 2 mmol), sealed with a septum and evacuated and backfilled with Ar (3 times). Dry dichloromethane (75 mL) was added, and the resulting dark orange solution was stirred for 5 min at room temperature. The volatiles were removed under reduced pressure, and pentane (30 mL) was added to triturate the residue. The resulting solids were collected, washed with a 10:1 solution of pentane: diethyl ether (3 x 30 mL), and dried under reduced pressure to afford nickel complex **C1** as a yellow solid (856.7 mg, 90%).

The ^{1}H and ^{13}C NMR spectra of **C1** were recorded at –30 ℃ to slow the fluxional processes associated with this complex.

^{1}H NMR (400 MHz, Methylene Chloride-d_2, -30°C) δ 8.40 (br, 1H), 7.87 (br, 2H), 7.43 (br, 1H), 7.16 (br, 2H), 6.08 – 5.54 (m, 1H), 5.20 – 4.55 (m, 1H), 1.35 (br, 18H). ^{13}C NMR (101 MHz, Methylene Chloride-d_2, -30 ^{o}C) δ 167.74, 164.82, 164.22, 155.47, 152.24, 147.30, 133.04, 128.09, 124.05, 123.90, 118.30, 117.73, 35.58, 35.55, 29.94, 29.91. ^{19}F NMR (376 MHz, Methylene Chloride-d_2) δ -107.57 (d, J = 49.1 Hz, 2F), -123.68 (d, J = 53.1 Hz, 2F). IR (cm^{-1}): 2964.9, 1674.8, 1617.7, 1414.4.

SAESI-MS Analysis of Nickel Complex C1

Procedure: The chemical solution of **C1** in CH_2Cl_2 was injected by a 500-μL air-tight syringe with a speed at 5 μL/min to SAESI-MS. The assisted solvent of methanol was injected by another 500-μL air-tight syringe with a speed at 10 μL/min to SAESI-MS.

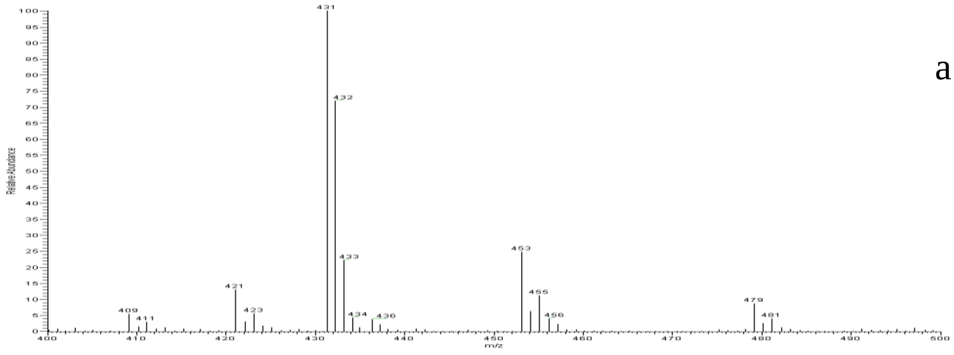

a

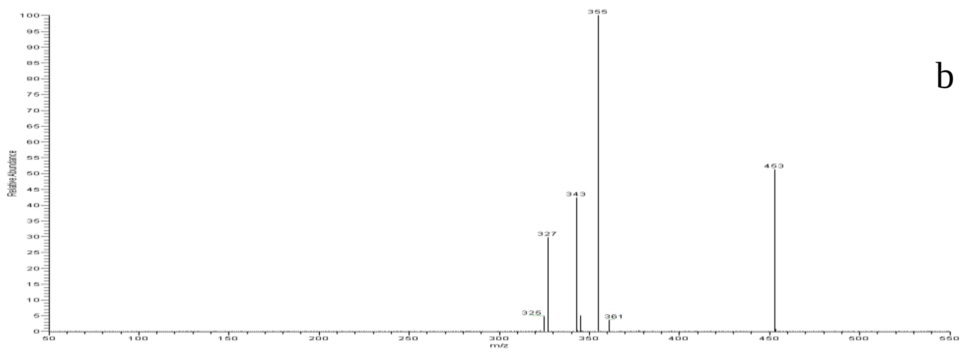

Supplementary Figure 141. a SAESI-MS spectrum of nickel complex **C1**, showing the complex ion $[M\text{-}F]^+$ at *m/z* 453. **b** The SAESI-MS/MS spectra of the ion at *m/z* 453.

Reaction of Nickel Complex C1 with Aryl Chloride 2a

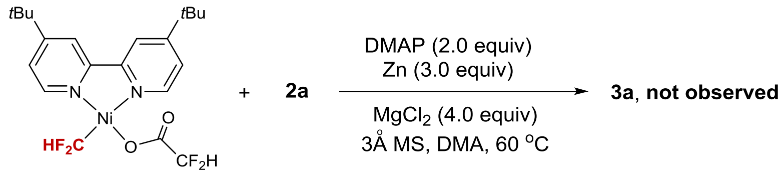

Procedure: To a 25 mL of Schlenk tube were added nickel complex **C1** (0.2 mmol, 1.0 equiv), Zn dust (3.0 equiv), $MgCl_2$ (4.0 equiv)， 3Å MS (100 mg) and DMAP (0.4 mmol, 2 equiv). The mixture was then evacuated and backfilled with Ar (3 times). DMA (2 mL) and aryl chloride **2a** (0.2 mol, 1.0 equiv) were then added. The Schlenk tube was screw capped and put into a preheated oil bath (60 ^{o}C). After stirring for 20 h, the reaction mixture was cooled to room temperature, diluted with ethyl acetate. The yield was determined by ^{19}F NMR using fluorobenzene as an internal standard. But no **3a** was observed.

Supplementary Table 16 Radical inhibition experiments

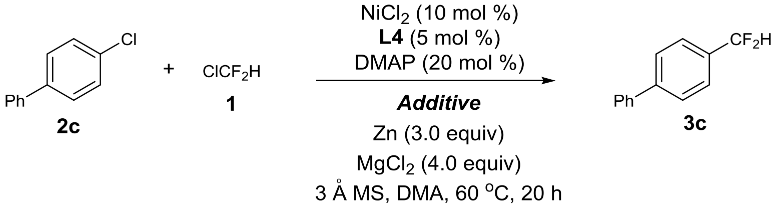

Additive	**3c**, yield (%)
TEMPO (20 mol %)	27
TEMPO (1.0 equiv)	ND
1,4-dinitrobenzene (20 mol %)	ND
1,4-dinitrobenzene (1.0 equiv)	ND

Procedure: To a 25 mL of Schlenk tube were added 4-chlorobiphenyl **2c** (0.2 mmol, 1.0 equiv), $NiCl_2$ (10 mol %), **L4** (5 mol %), zinc dust (3.0 equiv), $MgCl_2$ (4.0 equiv), 3 Å MS (100 mg), DMAP (20 mol%) and addtive (20 ~ 100 mol%). The mixture was evacuated and backfilled with argon for three times, then DMA (2 mL) and $ClCF_2H$ **1** (2.6 M in DMA, 1.3 mmol, 6.5 equiv) were added subsequently. The Schlenk tube was screw capped and put into a preheated oil bath (60 °C). After stirring for 20 h, the reaction mixture was cooled to room temperature and worked up with ethyl acetate. The yield was determined by ^{19}F NMR using fluorobenzene as an internal standard.

Radical Clock Experiments

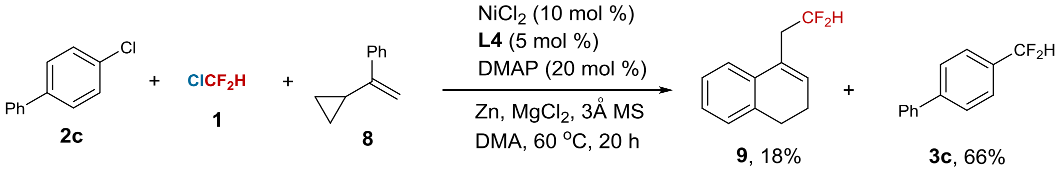

yields were determined by ^{19}F NMR

Procedure: To a 25 mL of Schlenk tube were added **2c** (0.2 mmol, 1.0 equiv), $NiCl_2$ (10 mol %), **L4** (5 mol %), Zn dust (3.0 equiv), $MgCl_2$ (4.0 equiv), 3Å MS (100 mg) and DMAP (20 mol %). The mixture was then evacuated and backfilled with Ar (3 times). Compound **8** (0.4 mmol, 2.0 equiv), DMA (2 mL) and $ClCF_2H$ (2.6 M in DMA, 1.3 mmol, 6.5 equiv) were added subsequently. The Schlenk tube was screw capped and put into a preheated oil bath (60 °C). After stirring for 20 h, the reaction mixture was cooled to room temperature, diluted with ethyl acetate, and filtered through a pad of celite. The filtrate was concentrated, and the residue was washed with 10 mL of water,

extracted with EtOAc for three times. The organic layer was washed with brine, dried over Na_2SO_4 and concentrated. The residue was purified by silica gel (Petroleum ether) to give product **9** (18% yield determined by ^{19}F NMR). Compound **9**: ^{1}H NMR (400 MHz, $CDCl_3$) δ 7.26 – 7.12 (m, 4H), 6.13 – 5.74 (m, 2H), 2.99 (td, J = 16.5, 4.2 Hz, 2H), 2.78 (t, J = 8.0 Hz, 2H), 2.35 – 2.25 (m, 2H). ^{19}F NMR (376 MHz, $CDCl_3$) δ -113.99 (dt, J = 56.7, 16.5 Hz, 2H). ^{13}C NMR (101 MHz, $CDCl_3$) δ 136.48, 133.86, 129.95 , 128.45 (t, J = 6.4 Hz), 127.85 , 127.20 , 126.50 , 122.27 , 116.09 (t, J = 240.8 Hz), 37.79 (t, J = 22.5 Hz), 27.99 , 23.10. (EI): m/z (%) 194 (M^+), 129 (100). HRMS: Calculated for $C_{12}H_{12}F_2$: 194.0907; Found: 194.0904.

Reaction of 2c and $ClCF_2H$ with Compound 8 in the Presence of TEMPO

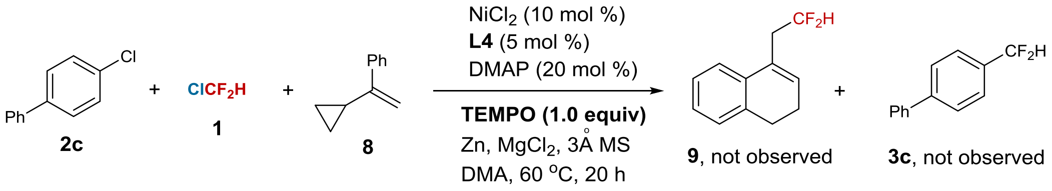

Procedure: To a 25 mL of Schlenk tube were added $NiCl_2$ (10 mol %), **L4** (5 mol %), Zn dust (3.0 equiv), $MgCl_2$ (4.0 equiv), 3Å MS (100 mg), DMAP (20 mol %) and TEMPO (1.0 equiv). The mixture was then evacuated and backfilled with Ar (3 times). Compound **8** (0.4 mmol, 2.0 equiv), **2c** (0.2 mmol, 1.0 equiv), DMA (2 mL) and $ClCF_2H$ (2.6 M in DMA, 1.3 mmol, 6.5 equiv) were then added. The Schlenk tube was screw capped and put into a preheated oil bath (60 °C). After stirring for 20 h, the reaction mixture was cooled to room temperature, diluted with ethyl acetate. The yield was determined by ^{19}F NMR using fluorobenzene as an internal standard. ^{19}F NMR showed both **3c** and compound **9** were not formed.

Reaction of Nickel Complex B1 with $ClCF_2H$ and 8

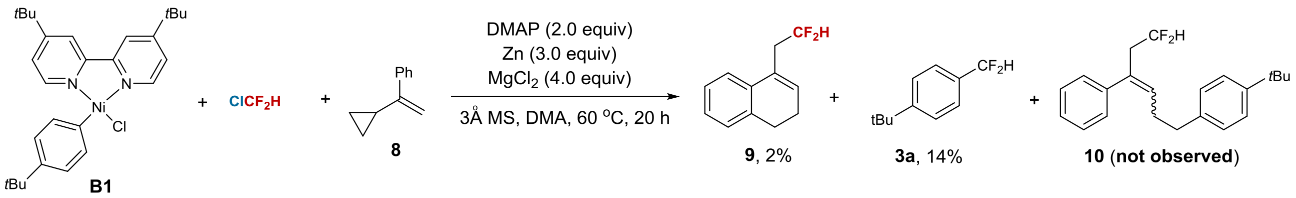

Procedure: To a 25 mL of Schlenk tube were added nickel complex **B1** (0.2 mmol, 1 equiv), DMAP (2 equiv), Zn dust (3 equiv), $MgCl_2$ (4 equiv) and 3Å MS (100 mg) under Ar. Compound **8** (0.4 mmol, 2 equiv), DMA (2 mL) and $ClCF_2H$ (2.6 M in DMA, 1.3 mmol, 6.5 equiv) were then added. The Schlenk tube was screw capped and put into a preheated oil bath (60 °C). After stirring for 20 h, the reaction mixture was cooled to room temperature, diluted with ethyl acetate. The yields were determined by ^{19}F NMR using fluorobenzene as an internal standard.

Reaction of Nickel Complex C1 with Aryl Chloride 2a and Compound 8

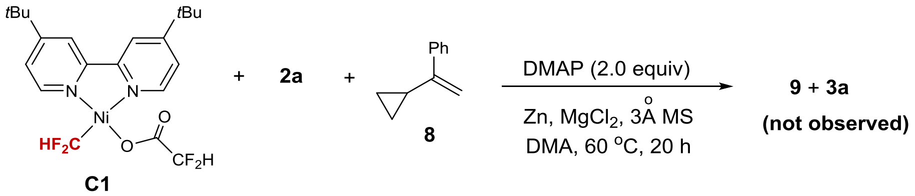

Procedure: To a 25 mL of Schlenk tube were added nickel complex **C1** (0.2 mmol, 1 equiv), DMAP (2 equiv), Zn dust (3 equiv), $MgCl_2$ (4 equiv) and 3Å MS (100 mg) under Ar. Compound **8** (0.4 mmol, 2 equiv), **2a** (0.2mmol, 2 equiv) and DMA (2 mL) were then added. The Schlenk tube was screw capped and put into a preheated oil bath (60 °C). After stirring for 20 h, the reaction mixture was cooled to room temperature, diluted with ethyl acetate. The yield was determined by ^{19}F NMR using fluorobenzene as an internal standard. But no compounds **9** and **3a** were formed.

Thus, this result demonstrates that the formation of compound **9** from **C1** through Ni-concerted insertion mechanism can be ruled out (Supplementary Figure 142b). Furthermore, the possibility of formation of compound **9** from aryldifluoromethyl nickel complex Ar-[Ni]-CF_2H generated *in situ* between **B1** and $ClCF_2H$ is also unlikely (Supplementary Figure 142c), as no ring-opening product **10** was observed by treatment of **B1** with $ClCF_2H$ and **8**. If compound **9** can be generated by nickel complex Ar-[Ni]-CF_2H, compound **10** should be produced by insertion of Ar-[Ni]-CF_2H into double

bond of **8**, followed by reductive elimination. Therefore, these results suggest that the formation of **9** via a radical pathway is reasonable and a difluoromethyl radical species may be involved in the current catalytic cycle.

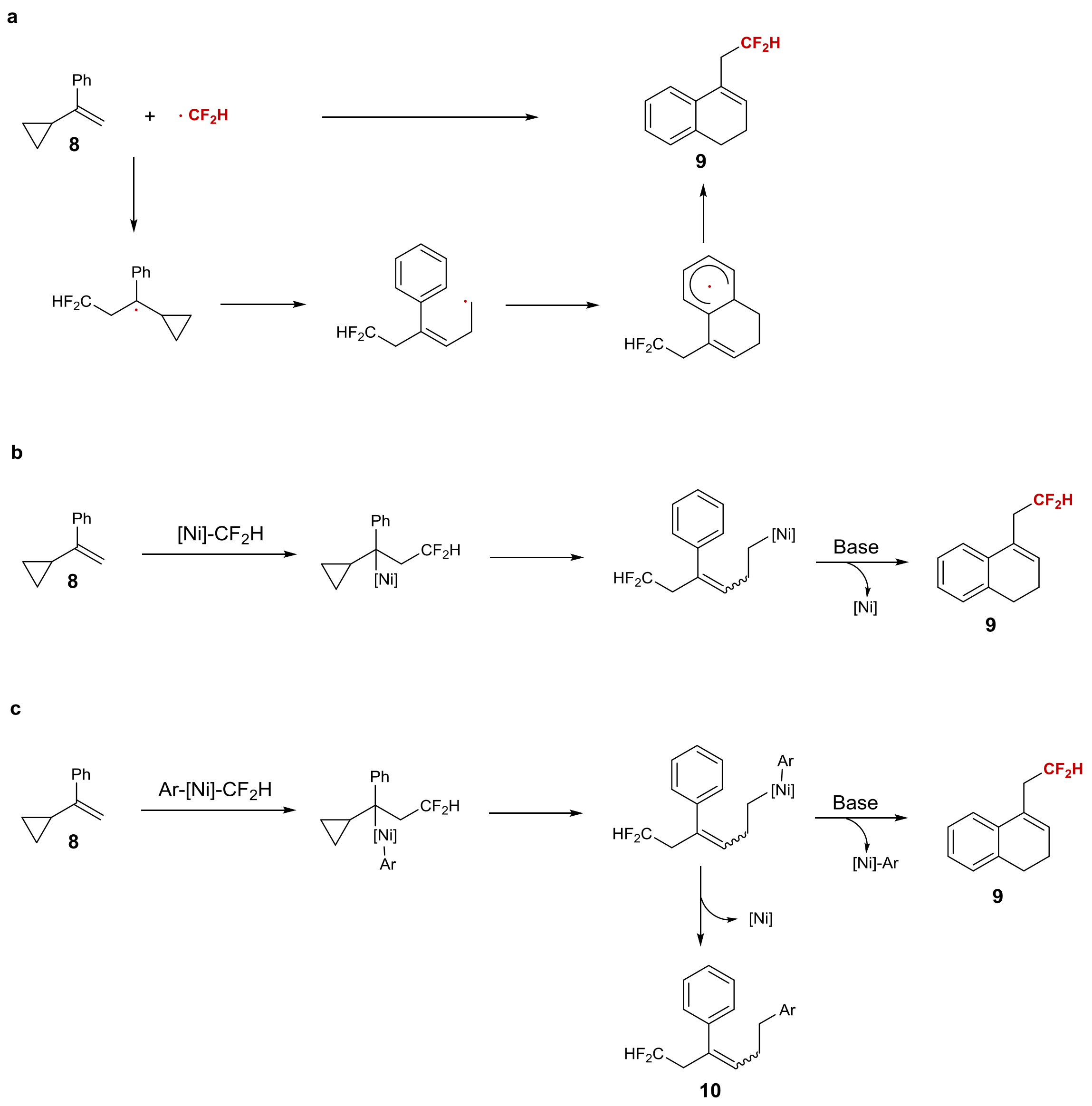

Supplementary Figure 142. Possible pathways for the formation of compound **9**. **a** Radical clock pathway. **b** Ni-concerted insertion mechanism for the formation of compound **9**. **c** Nickel complex Ar-[Ni]-CF_2H involved formation of compounds **9** and **10**.

The Role of DMAP

Preparation of Nickle complex D1

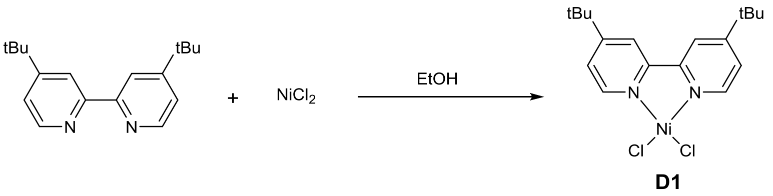

Procedure: Complex **D1** was prepared according to the literature.[12] To a solution of $NiCl_2$ (129.6 mg, 1.0 mmol) in ethanol (13 mL) was added a solution of 4,4′-di*t*Bu-bpy (271 mg, 1.0 mmol) in ethanol (7 mL). The reaction mixture was refluxed with stirring for 10 h. The yellow solution slowly became green. The solution was filtrated and the filtrate was evaporated in vacuo to give a crude product **D1** (358 mg, 90% yield). The product was recrystallized from methanol.

Preparation of Nickel Complex D2

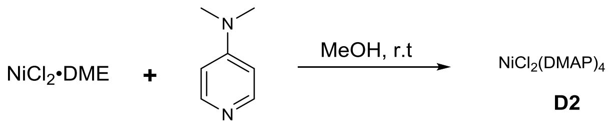

Procedure: Complex **D2** was prepared according to the literature.[12] To a stirring solution of DMAP (293 mg, 2.4 mmol, 8.0 equiv) in MeOH (5 mL) was added dropwise a solution of $NiCl_2\cdot$DME (66 mg, 0.3 mmol, 1.0 equiv) in MeOH (5 mL). After the reaction mixture was stirred at room temperature for another 6 h, the solution was filtrated. The filtrate was evaporated in vacuo to give a blue solid **D2**, which was recrystallized from methanol.

Nickel Complex D1 Catalyzed Cross-Coupling of 2a with $ClCF_2H$

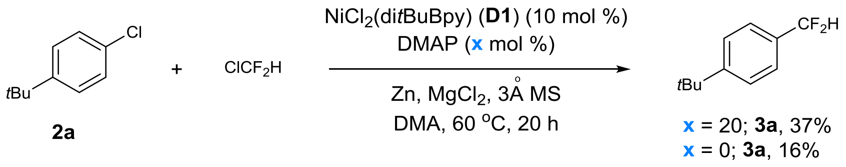

Procedure: To a 25 mL of Schlenk tube were added nickel complex **D1** (0.02 mmol, 10 mol %), Zn dust (3.0 equiv), $MgCl_2$ (4.0 equiv), 3Å MS (100 mg) and DMAP (20 mol % or 0 mol %). The mixture was then evacuated and backfilled with Ar (3 times). Aryl chloride **2a** (0.2 mmol, 1 equiv),

DMA (2 mL) and $ClCF_2H$ (2.6 M in DMA, 1.3 mmol, 6.5 equiv) were then added. The Schlenk tube was screw capped and put into a preheated oil bath (60 °C). After stirring for 20 h, the reaction mixture was cooled to room temperature, diluted with ethyl acetate. The yield was determined by ^{19}F NMR using fluorobenzene as an internal standard.

Nickel Complex D2 Catalyzed Cross-Coupling of 2a with $ClCF_2H$

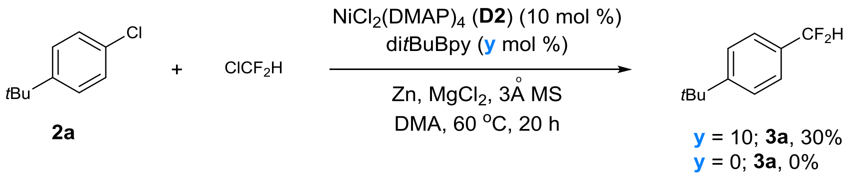

Procedure: To a 25 mL of Schlenk tube were added nickel complex **D2** (0.02 mmol, 10 mol %), 4,4′-di-*t*Bu-2,2′-bpy (10 mol % or 0 mol %), Zn dust (3.0 equiv), $MgCl_2$ (4.0 equiv) and 3Å MS (100 mg). The mixture was then evacuated and backfilled with Ar (3 times). **2a** (0.2 mmol, 1 equiv), DMA (2 mL) and $ClCF_2H$ (2.6 M in DMA, 1.3 mmol, 6.5 equiv) were then added. The Schlenk tube was screw capped and put into a preheated oil bath (60 °C). After stirring for 20 h, the reaction mixture was cooled to room temperature, diluted with ethyl acetate. The yield was determined by ^{19}F NMR using fluorobenzene as an internal standard.

Reaction of B_2 with $ClCF_2H$

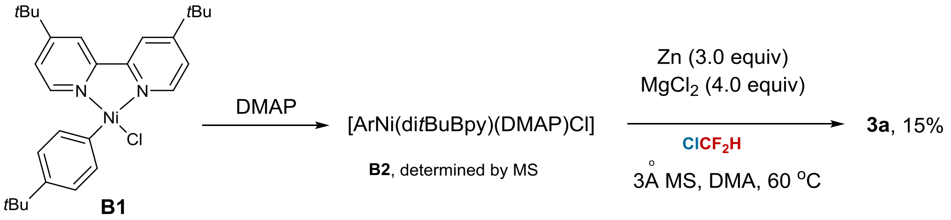

Procedure: To a Schlenk tube were added nickel complex **B1** (0.2 mol, 1.0 equiv), DMAP (0.2 mol equiv) and DMA (2 mL). The resulting mixture was then stirred at room temperature for 20 min. Mass spectrometric analysis of the reaction showed the complex ion $[M-Cl]^+$ at *m/z* 581, which is corresponding to a DMAP coordinated nickel complex **B2** (Supplementary Figure 143). The resulting solution was added to the mixture of Zn dust (3.0 equiv), $MgCl_2$ (4.0 equiv) and 3Å MS (100 mg). Subsequently, $ClCF_2H$ (2.6 M in DMA, 1.3 mmol, 6.5 equiv) was added. The Schlenk

tube was screw capped and put into a preheated oil bath (60 ^{o}C). After stirring for 8 h, the reaction mixture was cooled to room temperature, diluted with ethyl acetate. The yield was determined by ^{19}F NMR using fluorobenzene as an internal standard.

SAESI-MS Analysis of Nickel Complex B2

Procedure: The chemical solution of **B2** in CH_2Cl_2 was injected by a 500-μL air-tight syringe with a speed at 5 μL/min to SAESI-MS. The assisted solvent of methanol was injected by another 500-μL air-tight syringe with a speed at 10 μL/min to SAESI-MS.

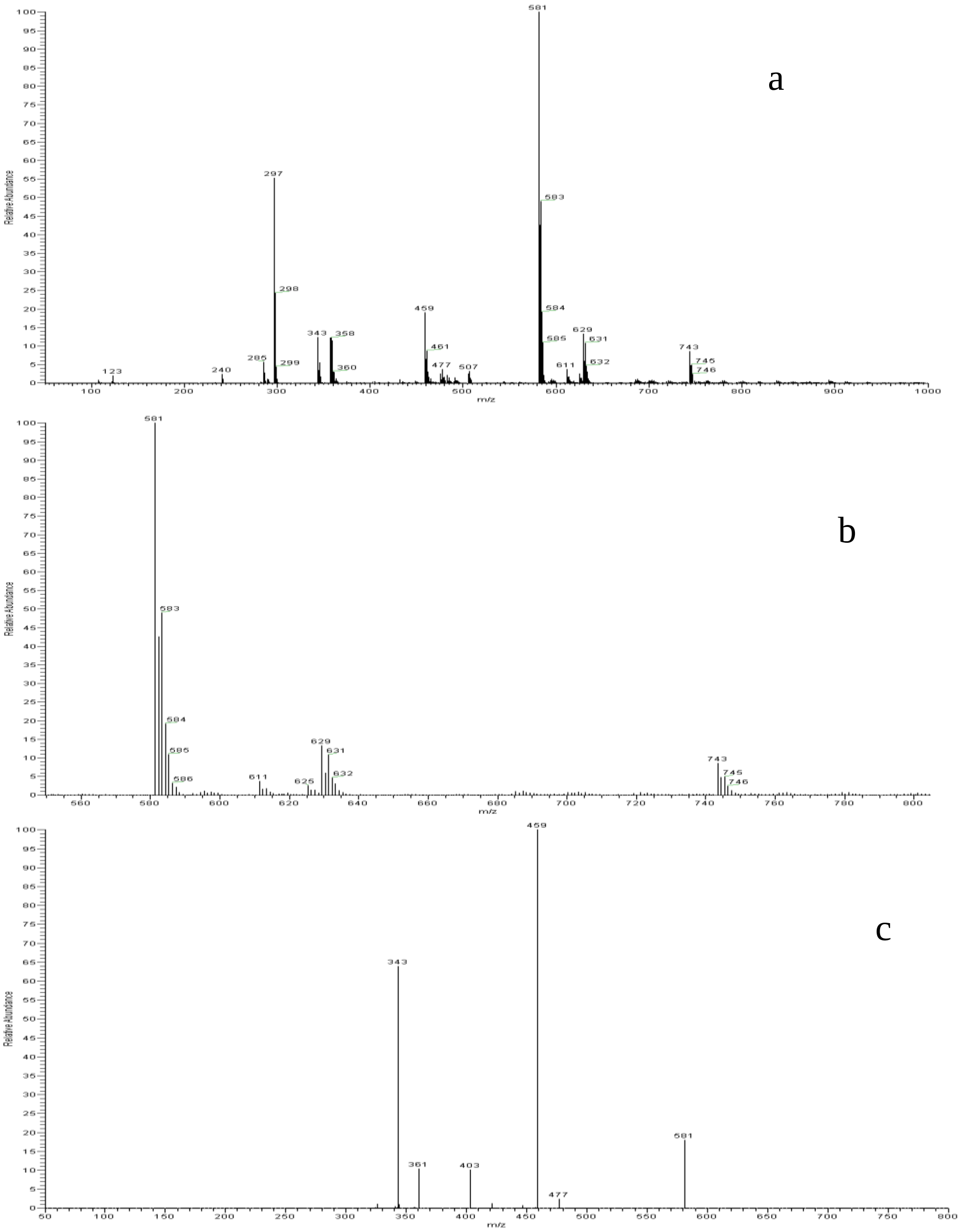

Supplementary Figure 143. **a** SAESI-MS spectrum of nickel complex **B2**, showing the complex ion [M-Cl]$^{+}$ at *m/z* 581. **b** The expanded ESI-MS spectrum of **B2**. **c** The SAESI-MS/MS spectra of the ion at *m/z* 581.

Reaction of Nickel Complex B1 with $ClCF_2H$ in the Absence of DMAP

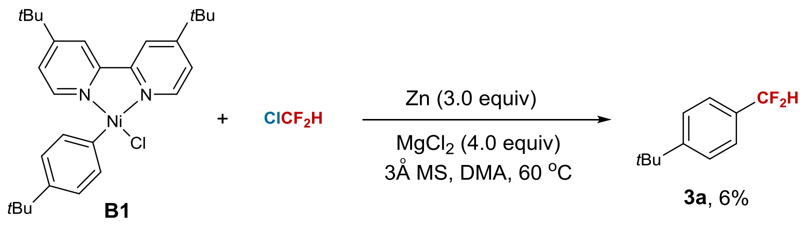

Procedure: To a 25 mL of Schlenk tube were added nickel complex **B1** (0.2 mmol, 1.0 equiv), Zn dust (3.0 equiv), $MgCl_2$ (4.0 equiv), and 3Å MS (100 mg). The mixture was then evacuated and backfilled with Ar (3 times). DMA (2 mL) and $ClCF_2H$ (2.6 M in DMA, 1.3 mmol, 6.5 equiv) were then added. The Schlenk tube was screw capped and put into a preheated oil bath (60 °C). After stirring for 8 h, the reaction mixture was cooled to room temperature, diluted with ethyl acetate. The yield was determined by ^{19}F NMR using fluorobenzene as an internal standard.

Hammett-Type Analysis of the Reaction

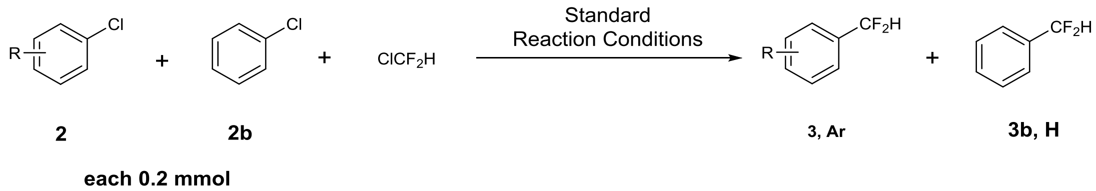

Procedure of Competition Reactions for Hammett Plot: To a 25 mL of Schlenk tube were added $NiCl_2$ (15 mol %), **L4** (10 mol %), zinc dust (3.0 equiv), $MgCl_2$ (4.0 equiv), 3Å MS (100 mg) and DMAP (20 mol %). The mixture was evacuated and backfilled with argon for three times. Chlorobenzene **2b** (0.2 mmol, 1 equiv), corresponding aryl chloride **2** (0.2 mmol, 1 equiv)**,** DMA (2 mL) and $ClCF_2H$ (2.6 M in DMA, 1.3 mmol, 6.5 equiv) were then added. The Schlenk tube was screw capped and put into a preheated oil bath (60 °C). After stirring for 20 h, the reaction mixture was cooled to room temperature and diluted with ethyl acetate. The yield of **3b** and corresponding **3** were determined by ^{19}F NMR using fluorobenzene as an internal standard. Linear regression was performed with 21 data point sets (3 runs for each substrate and (0, 0)). The Hammett ρ value was obtained in Excel with LINEST function.

Supplementary Table 17 Hammett-type analysis of nickel-catalyzed cross-coupling of aryl chlorides with $ClCF_2H^a$

R	^{19}F-NMR Yield(%)^a		3b/3	k_{Ar}/k_H	$\log(k_{Ar}/k_H)$	Average	$\sigma^{ref\ 13}$	$\sigma(-)^{ref\ 13}$
	**3b, H**	**3, Ar**						
p-tBu	55	41	1.341	0.7455	-0.1276	-0.1174	-0.197	-0.202
p-tBu	78	69	1.130	0.8846	-0.0532			
p-tBu	46	31	1.484	0.6739	-0.1714			
p-OMe	73	34	2.147	0.4658	-0.3318	-0.3078	-0.268	-0.312
p-OMe	70	33	2.121	0.4714	-0.3266			
p-OMe	81	44	1.841	0.5432	-0.2650			
m-OMe	78	70	1.114	0.8974	-0.0470	-0.0532	0.115	0.100
m-OMe	76	71	1.070	0.9342	-0.0296			
m-OMe	46	38	1.211	0.8261	-0.0830			
p-COOEt	1	28	0.036	28.00	1.4472	1.4334	0.678	0.752
p-COOEt	2	46	0.043	23.00	1.3617			
p-COOEt	1	31	0.032	31.00	1.4914			
p-F	34	36	0.944	1.0588	0.0248	0.0327	0.062	-0.056
p-F	32	34	0.941	1.0625	0.0263			
p-F	35	39	0.897	1.1143	0.0470			
p-CF_3	1	30	0.033	30.00	1.4771	1.4765	0.650	0.648
p-CF_3	2	56	0.036	28.00	1.4472			
p-CF_3	1	32	0.031	32.00	1.5051			
m-OCF_3	7	63	0.111	9.0000	0.9542	0.9376	0.470	0.463
m-OCF_3	6	52	0.115	8.6667	0.9379			
m-OCF_3	6	50	0.120	8.3333	0.9208			
4-H			1	1	0	0.0000	0.000	0.000

^{a}Yields were determined by ^{19}F NMR

On the basis of these proposed mechanisms, a cage difluoromethyl $\cdot CF_2H$ is involved in the catalytic cycle. In the Supplementary Figure 144b,[14] the $\cdot CF_2H$ diffuses to the solution to combine with [Ar-Ni^{II}-Cl] (**B**) to produce the key intermediate **F**. The generation of a cage $\cdot CF_2H$ may be the explanation for the observation of higher yield of difluoromethylated arene **3c** comparing with the yield of ring-expanded product **9** in the reaction of aryl chloride **2c** and $ClCF_2H$ with vinyl cyclopropane **8** (Figure 4e). Because the cage $\cdot CF_2H$ in Supplementary Figure 144a preferentially combines with [Ar-Ni^{II}-Cl] (**B**) to give difluoromethylated arene after reductive elimination of **F**. On

the other hand, some $\cdot CF_2H$ radical diffuses to the solution to produce compound **9**, which is an unfavorable path way, as a result, lower yield of **9** was obtained.

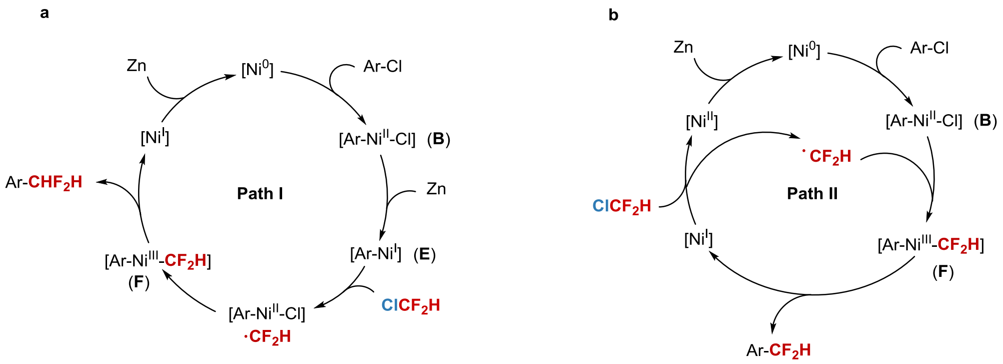

Supplementary Figure 144. Proposed reaction mechanisms. **a** Proposed mechanism via a radical-cage-rebound process. **b** Proposed mechanism via a radical chain process.

Supplementary References

(1) Feng, Z., Min, Q. Q. & Zhang, X. Access to difluoromethylated arenes by Pd-Catalyzed reaction of arylboronic acids with bromodifluoroacetate. *Org. Lett.* **18**, 44-47 (2016).

(2) Mizuta, S. *et al.* Catalytic decarboxylative fluorination for the synthesis of tri- and difluoromethyl arenes. *Org. Lett.* **15**, 2648-2651 (2013).

(3) Matheis, C., Jouvin, K. & Goossen, L. J. Sandmeyer difluoromethylation of (hetero-)arenediazonium salts. *Org. Lett.* **16**, 5984-5987 (2014).

(4) Li X, *et al.* Copper-mediated aerobic (phenylsulfonyl)difluoromethylation of arylboronic acids with difluoromethyl phenyl sulfone. *Chem. Commun.* **52**, 3657-3660 (2016).

(5) Serizawa H, Ishii K, Aikawa K, Mikami K. Copper-Catalyzed Difluoromethylation of Aryl Iodides with (Difluoromethyl)zinc Reagent. *Org. Lett.* **18**, 3686-3689 (2016).

(6) Feng Z., Min Q. Q., Fu X. P., An L., Zhang X. Chlorodifluoromethane-triggered formation of difluoromethylated arenes catalysed by palladium. *Nat Chem* **9**, 918-923 (2017).

(7) Haas A, Spitzer M, Lieb M. Synthese seitenkettenfluorierter aromatischer Verbindungen und deren chemische Reaktivit ä t. *Chem. Ber.* **121**, 1329-1340 (1988).

(8) Burton, D. J. & Hartgraves, G. A. The preparation of HCF_2CdX and HCF_2ZnX via direct insertion into the carbon halogen bond of CF_2HY (Y=Br, I). *J. Fluorine Chem.* **128**, 1198-1215 (2007).

(9) Shields, B. J. & Doyle, A. G. Direct C(sp3)-H cross coupling enabled by catalytic generation of chlorine radicals. *J. Am. Chem. Soc.* **138**, 12719-12722 (2016).

(10) Maleckis, A. & Sanford, M. S. Synthesis of fluoroalkyl palladium and nickel complexes via decarbonylation of acylmetal species. *Organometallics* **33**, 3831-3839 (2014).

(11) Bour, J. R., Camasso, N. M. & Sanford, M. S. Oxidation of Ni(II) to Ni(IV) with aryl electrophiles enables Ni-mediated aryl-CF_3 coupling. *J. Am. Chem. Soc.* **137**, 8034-8037 (2015).

(12) Xiao, Y. L., Min, Q. Q., Xu, C., Wang, R. W. & Zhang, X. Nickel-catalyzed difluoroalkylation of (hetero)arylborons with unactivated 1-bromo-1,1-difluoroalkanes. *Angew. Chem. Int. Ed.* **55**, 5837-5841 (2016).

(13) Kuang-Chih, T. A study on the linear free energy relationships for amines I. A relatively complete set of new σ- values for anilines. *Acta Chim. Sinica* **32**, 107-121,136,137 (1966).

(14) Biswas, S. & Weix, D. J. Mechanism and selectivity in nickel-catalyzed cross-electrophile coupling of aryl halides with alkyl halides. *J. Am. Chem. Soc.* **135**, 16192-16197 (2013).
