# Supplementary material for: Difluoromethylation of (hetero)aryl chlorides with chlorodifluoromethane catalyzed by nickel
Source: Nat Commun. 2018 Mar 21;9:1170. doi: 10.1038/s41467-018-03532-1 (PMC5862906; doi:10.1038/s41467-018-03532-1)
Supplement: Supplementary file 2 — Description of Additional Supplementary Files(PDF 165 kb) [file 41467_2018_3532_MOESM2_ESM.pdf]

Description of Additional Supplementary Files

File Name: Supplementary Data 1
Description: X-ray crystal structure of nickel complex C1.
