# Supplementary material for: Difluoromethylation of (hetero)aryl chlorides with chlorodifluoromethane catalyzed by nickel
Source: Nat Commun. 2018 Mar 21;9:1170. doi: 10.1038/s41467-018-03532-1 (PMC5862906; doi:10.1038/s41467-018-03532-1)
Supplement: Supplementary file 5 — Description for the revised Supplementary Information(PDF 147 kb) [file 41467_2018_3532_MOESM5_ESM.pdf]

Description for the revised Supplementary Information

Date:	March 8, 2018
Manuscript Number:	DOI:10.1038/s41467-018-03532-1
Corresponding Author Name:	Xingang Zhang

R1: The "DMAP (2.0 equiv)" in the Scheme for nickel complex **B1** or $NiCl_2$/ditBuBpy Catalyzed Cross-Coupling of Aryl Chloride **2a** with $ClCF_2H$ in page S103 has been changed into "DMAP (20 mol%)", see following

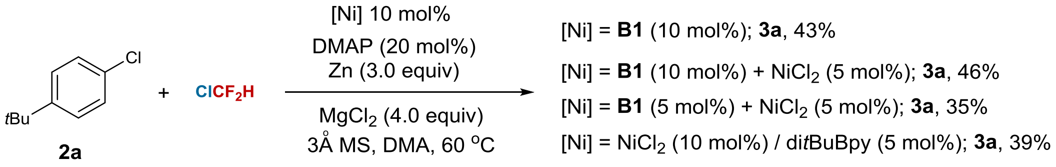

R2: The "**9**, 18%" and "**3c**, 66%" in the Scheme for Reaction of **2c** and $ClCF_2H$ with compound **8** in the presence of TEMPO in page S107 has been changed into "**9**, not observed" and "**3c**, not observed", see following

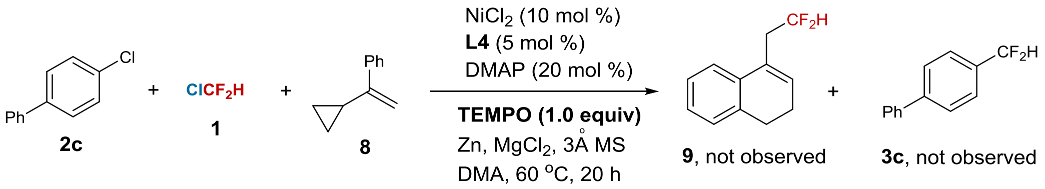

R3: "Figure S3" in page S111 has been changed into "Supplementary Figure 143".

R4: "Supplementary Figure 142b" in page S114 has been changed into "Supplementary Figure 144b".

R5: "Supplementary Figure 142a" in page S114 has been changed into "Supplementary Figure 144a".
